# Linear-Scaling Systematic Molecular Fragmentation Approach for Perturbation Theory and Coupled-Cluster Methods

**DOI:** 10.1021/acs.jctc.2c00587

**Published:** 2022-08-16

**Authors:** Uğur Bozkaya, Betül Ermiş

**Affiliations:** Department of Chemistry, Hacettepe University, Ankara 06800, Turkey

## Abstract

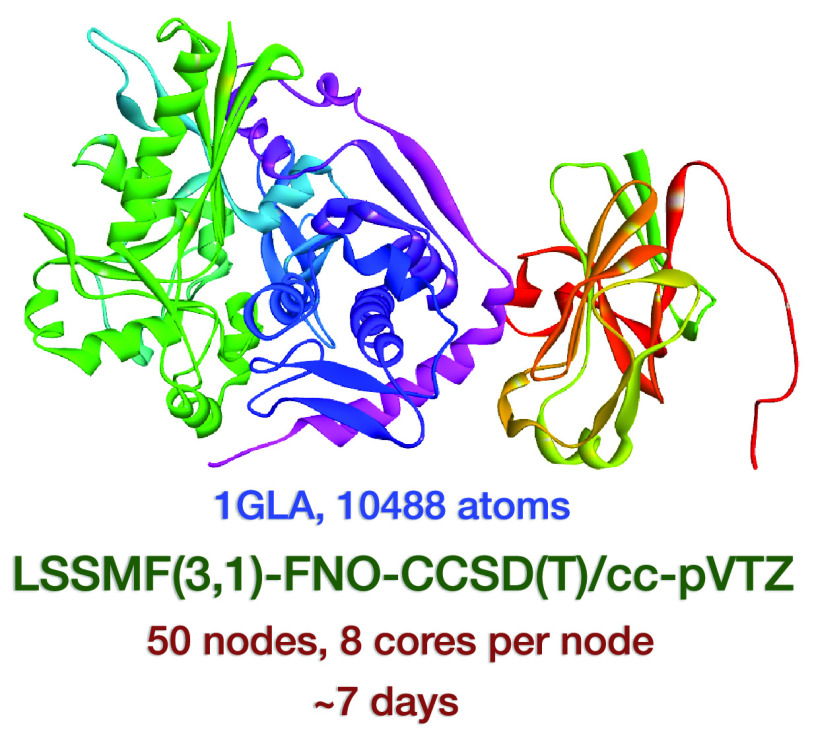

The coupled-cluster (CC) singles and doubles with perturbative triples [CCSD(T)] method is frequently referred to as the “gold standard” of modern computational chemistry. However, the high computational cost of CCSD(T) [*O*(*N*^7^)], where *N* is the number of basis functions, limits its applications to small-sized chemical systems. To address this problem, efficient implementations of linear-scaling coupled-cluster methods, which employ the systematic molecular fragmentation (SMF) approach, are reported. In this study, we aim to do the following: (1) To achieve exact linear scaling and to obtain a pure *ab initio* approach, we revise the handling of nonbonded interactions in the SMF approach, denoted by LSSMF. (2) A new fragmentation algorithm, which yields smaller-sized fragments, that better fits high-level CC methods is introduced. (3) A modified nonbonded fragmentation scheme is proposed to enhance the existent algorithm. Performances of the LSSMF-CC approaches, such as LSSMF-CCSD(T), are compared with their canonical versions for a set of alkane molecules, C_*n*_H_2*n*+2_ (*n* = 6–10), which includes 142 molecules. Our results demonstrate that the LSSMF approach introduces negligible errors compared with the canonical methods; mean absolute errors (MAEs) are between 0.20 and 0.59 kcal mol^–1^ for LSSMF(3,1)-CCSD(T). For a larger alkanes set (L12), C_*n*_H_2*n*+2_ (*n* = 50–70), the performance of LSSMF for the second-order perturbation theory (MP2) is investigated. For the L12 set, various bonded and nonbonded levels are considered. Our results demonstrate that the combination of bonded level 6 with nonbonded level 2, LSSMF(6,2), provides very accurate results for the MP2 method with a MAE value of 0.32 kcal mol^–1^. The LSSMF(6,2) approach yields more than a 26-fold reduction in errors compared with LSSMF(3,1). Hence, we obtain substantial improvements over the original SMF approach. To illustrate the efficiency and applicability of the LSSMF-CCSD(T) approach, we consider an alkane molecule with 10,004 atoms. For this molecule, the LSSMF(3,1)-CCSD(T)/cc-pVTZ energy computation, on a Linux cluster with 100 nodes, 4 cores, and 5 GB of memory provided to each node, is performed just in ∼24 h. As a second test, we consider a biomolecular complex (PDB code: 1GLA), which includes 10,488 atoms, to assess the efficiency of the LSSMF approach. The LSSMF(3,1)-FNO–CCSD(T)/cc-pVTZ energy computation is completed in ∼7 days for the biomolecular complex. Hence, our results demonstrate that the LSSMF-CC approaches are very efficient. Overall, we conclude the following: (1) The LSSMF(*m*, *n*)-CCSD(T) methods can be reliably used for large-scale chemical systems, where the canonical methods are not computationally affordable. (2) The accuracy of bonded level 3 is not satisfactory for large chemical systems. (3) For high-accuracy studies, bonded level 5 (or higher) and nonbonded level 2 should be employed.

## 1 Introduction

It has been demonstrated that coupled-cluster (CC) methods are accurate for the prediction of molecular properties.^1−5^ The coupled-cluster singles and doubles (CCSD) method^6^ provides quite accurate results for most molecular systems at equilibrium geometries, but nevertheless, a triple excitations correction is required to obtain high accuracy.^7−13^ The coupled-cluster singles and doubles with perturbative triples [CCSD(T)] method^10,11,14^ provides excellent results for a broad range of chemical systems near equilibrium geometries.^12,15−24^ Therefore, the CCSD(T) method generally referred to as the “gold standard” of computational chemistry. However, the high computational cost of CCSD(T) [*O*(*N*^7^)], where *N* is the number of basis functions, limits its applications to small-sized chemical systems.

There have been many attempts at development of reduced cost electron correlation methods.^25−35^ Some of these studies take advantages of the locality of molecular orbitals (MO), which is based on the idea that dynamic correlation is a short-range phenomenon. The introduction of a “correlation domain” concept, by Pulay and co-workers,^25,26^ stimulated local correlation approaches. There are a few variants of local CC methods, such as projected atomic orbitals-based local CC methods (PAO-LCC)^28,29^ and local pair natural orbitals (LPNOs).^32−34^ Other attempts are the cluster-in-molecule (CIM) approach^36−40^ and the divide–expand–consolidate (DEC) approach.^41,42^

Alternative and more effective approaches, compared to LCC methods, to tackle the molecular size dependence problems of electronic structure theories are the molecular fragmentation approaches (MFA). Various molecular fragmentation approaches have been suggested to overcome the steep scaling problem of electronic structure methods.^43−46^ In molecular fragmentation approaches, a molecular system is broken up into small molecular units, and energies of the fragments are combined to approximate the energy of the entire system. Although, the logic behind all fragmentation approaches is similar, the formation of fragments, as well as the combination of the fragment energies, differ significantly from method to method. Molecular fragmentation methods include the molecular tailoring approach (MTA),^47−49^ fragment molecular orbital theory (FMO),^50−52^ molecular fractionation with conjugate caps (MFCC),^53,54^ systematic molecular fragmentation (by annihilation) [SMF(A)],^44,55−64^ combined fragmentation method (CFM),^60,65^ generalized energy-based fragmentation (GEBF),^66^ kernel energy method (KEM),^67,68^ molecules-in-molecules (MIM) approach,^69^ many-overlapping-body expansion (MOBE),^70^ and generalized many-body expansion (GMBE).^71^

In terms of accuracy and general applicability, the SMF approach appears to be very attractive. The SMF energy is a sum of two components: bonded and nonbonded. We may also call them as covalent and noncovalent terms. The number of bonded fragments scales linearly [*O*(*n*)], where *n* is the number of groups, while the number of nonbonded fragments scales quadratically [*O*(*n*^2^)]. To reduce the high cost of nonbonded fragments, Collins introduced a cutoff distance (*R*_*cut*_), such as 2 Å.^61^ If the distance between monomers of a nonbonded fragment is smaller than *R*_*cut*_, then it is treated with electronic structure methods, otherwise, with a simple perturbation theory approach. For branched molecules, Collins’ algorithm yields large-sized fragments compared to the chain-like linear alkanes case, which is another difficulty.^55,56^ This situation especially becomes problematic for high-level CC approaches, such as CCSD(T), where the computational cost increases steeply with the molecular size.

In this research, to achieve exact linear scaling and to obtain a pure *ab initio* approach, we completely neglect all long-range nonbonded contributions since they already approach zero. Further, we introduce a new fragmentation algorithm for the branched molecules, which yields smaller-sized fragments; hence, the new algorithm better fits high-level CC methods. The new linear-scaling SMF algorithm, denoted by LSSMF, has been coded in C++ language by the present authors and added to the MacroQC(72) software. The LSSMF approach is integrated with the Dfocc module.^24,73−82^ Hence, all methods available in the Dfocc module can be used with the LSSMF approach. The newly proposed LSSMF-CC approaches, such as LSSMF-CCSD and LSSMF-CCSD(T) as well as LSSMF-MP2, are applied to a series of alkane molecules to demonstrate their efficiencies and accuracies.

## 2 Systematic Molecular Fragmentation (SMF)

The SMF approach starts with the molecule *M* divided into different “groups”. Groups are sets of atoms defined by the SMF algorithm. The basic ideas involved in the method can be illustrated for the simplest case involving a chain-like molecule containing *N* groups connected by single bonds:
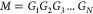
1The target is to derive an accurate value for the total electronic energy:
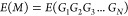
2

The energy of the molecule *M* is determined by summation of the fragment (*F*_*n*_), which is defined in terms of combinations of groups and energies. The sizes of the fragments depend on the “level” of SMF, and the fragments can overlap with each other since a group can be involved in multiple fragments.^59^ Hence, additional fragments with negative coefficients are generated to cancel the effects of multiple counting.

The bonded energy is
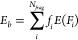
3where *f*_*i*_ is the integer coefficient associated with the fragment *F*_*i*_.

For a model system of a chain containing five groups, the SMF fragmentation scheme can be expressed as
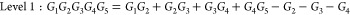
4
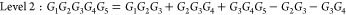
5
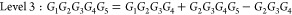
6

Thus, the fragment sizes increase with the level used. However, the number of fragments grows linearly with the size of the system. The authors have noted that the different levels used in SMF are related to some older concepts used in the field of theoretical thermochemistry. For example, level 1 reactions are known as “isodesmic reactions”,^83^ level 2 as homodesmotic reactions,^84^ and level 3 as hyperhomodesmotic^85^ reactions.

Since the bonded energy only includes nearby interactions, one should consider the nonbonded interactions between more distant groups. The nonbonded interactions may be evaluated by the following equation:
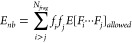
7The “allowed” nonbonded interactions are the interactions that are not already included in *E*_*b*_.^44,60^

### 2.1 New Linear-Scaling SMF (LSSMF) Approach

To illustrate the difference of our LSSMF approach from the previous SMF/SMFA approach(es), let us consider an open-chain alkane molecule. For a chain-like *C*_*n*_*H*_2*n*+2_ molecule with the SMF scheme (at level 3), the bonded fragments are just butane and propane fragments when hydrogen caps are added. The nonbonded fragments are just methane dimers with different molecular distances. The number of bonded and nonbonded fragments are given as follows:
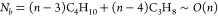
8
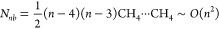
9The number of bonded fragments scales linearly with the number of carbons, while the number of nonbonded fragments (NB) scales quadratically. However, one may consider only short-range NB fragments, and their number also scales linearly with the system size.
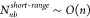
10Hence, we introduce a nonbonded cutoff tolerance, Δ_*nb*_. If the distance between the closest atoms of two groups is larger than Δ_*nb*_, then this nonbonded fragment is disregarded. We denote this algorithm by *distance-based elimination* (DBE). An alternative approach is using the ratio of distance to covalent radii (DCRR) as follows:^86^
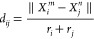
11where *X*_*i*_^*m*^ denotes the Cartesian position of the atom in the fragment *m*, and *r*_*i*_ denotes the covalent radius of the atom. Atomic covalent radii are obtained from Cordero et al.^87^

In this study, we consider different bonded and nonbonded fragmentation levels. Hence, we introduce the LSSMF(*m*, *n*) notation, where *m* and *n* indicate the bonded and nonbonded levels, respectively.

### 2.2 LSSMF Fragmentation Algorithm

Before presenting our fragmentation algorithm, let us define the notation: *i*, *j*, *k*, *l*, ... for atoms; *a*, *b*, *c*, *d*, ... for groups; and μ, ν, λ, σ, ... for fragments.

1. Define the level of SMF and tolerances for single, double, and triple bonds as well as the NB cutoff: Δ_*sb*_, Δ_*db*_, Δ_*tb*_, and Δ_*nb*_.
2. Read molecular info: Cartesian coordinates (*X*, *Y*, and *Z*), number of atoms (*N*_*atom*_), atomic masses, and atomic covalent radii (*r*_*i*_).
3. Compute interatomic distances: *R*_*ij*_.
4. Compute bond order matrix: *B*_*ij*_.
  - If *R*_*ij*_ < *r*_*i*_ + *r*_*j*_ + Δ_*sb*_, then *B*_*ij*_ = 1.
  - If *R*_*ij*_ < *r*_*i*_ + *r*_*j*_ – Δ_*db*_, then *B*_*ij*_ = 2.
  - If *R*_*ij*_ < *r*_*i*_ + *r*_*j*_ – Δ_*tb*_, then *B*_*ij*_ = 3.
  - Else *B*_*ij*_ = 0.
5. Catch the first nonhydrogen atom. The first such atom is assigned to group 1 (in fact, group 0 in C++).
6. Assign the remaining non-H atoms. Starting the first non-H atom, make a loop over atom pairs *i*, *j*. If *B*_*ij*_ > 1, then assign *j* to the group of the *i*th atom, *G*_*i*_. Otherwise, assign it to the next group, *G*_*i*+1_.
7. Catch double/triple bonded non-H atoms in different groups and merge them.
8. Assign the hydrogen atoms to each group according to values of *B*_*ij*_.
9. Form the group connectivity matrix: *L*_*ab*_. If two groups are connected to each other, then *L*_*ab*_ = 1; otherwise, it is equal to zero. Further, determine the bonded atoms of two connected groups: *LA*_*ab*_.
10. Determine the number of caps per group.
11. Form bonded and nonbonded domains for each group. For group *G*_*i*_, the bonded domain is the list of groups *G*_*j*_ (*j* ≠ *i*) that are connected to *G*_*i*_. Similarly, the nonbonded domain is the list of groups *G*_*j*_ (*j* ≠ *i*) that are not connected to *G*_*i*_, within the nonbonded cutoff tolerance.
12. Form lists of groups and bonded and nonbonded fragments according to the SMF level. Details of bonded and nonbonded fragment algorithms are provided in Sections 2.4 and 2.5, respectively.
13. Add embedded charges to groups and fragments in the case of polar molecules.^88^
14. Write MacroQC input files for groups and bonded and nonbonded fragments.

### 2.3 Capping Hydrogens

In each final fragment, bonds that are connecting groups in the fragment to other groups that are not in the fragment are “missing”. These missing bonds are replaced by bonds to hydrogen atoms.^55^ The total number of hydrogen atoms added to fragments with a sign of +1 is exactly equal to the number added to fragments with a sign of −1. The position of each H atom is taken to lie along the missing bond vector at a distance which is proportional to the expected ratio of bond lengths. That is,
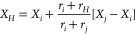
12where *X*_*i*_ denotes the Cartesian position of the atom in the fragment, and *X*_*j*_ denotes the Cartesian position of the atom that is not available in the fragment.

### 2.4 New Fragmentation Algorithm for Branched Molecules

**Figure 1:**
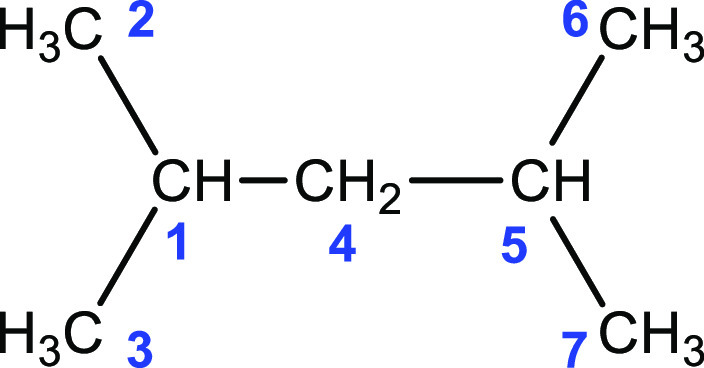
2,4-Dimethylpentane (24DMP) molecule.

Our fragmentation algorithm is identical to the one suggested by Deev and Collins^55^ for unbranched chain-like molecules. However, in the case of branched molecules, we propose a new algorithm. In order to illustrate the difference between two algorithms, let us consider the 2,4-dimethylpentane (24DMP) molecule (Figure 1) for which the fragmentation result at level 3 was reported by Deev and Collins.^55^

In the 24DMP molecule, each carbon atom defines a group, with a total of seven groups. Fragmentation suggested by Deev and Collins yields to the following bonded fragments at level 3:^55^
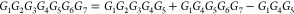
13where *G*_1_*G*_2_*G*_3_*G*_4_*G*_5_*G*_6_*G*_7_ represents the whole molecule. In this case, fragments *G*_1_*G*_2_*G*_3_*G*_4_*G*_5_ and *G*_1_*G*_4_*G*_5_*G*_6_*G*_7_ are formed from the combination of the five groups. However, in the case of an open chain analog, the fragments form from the combination of four groups. Hence, Deev and Collins’ algorithm yields fragments at different sizes for open chain and branched molecules. In the latter case, it yields much larger fragments, which may be a problem for high-level CC methods, where the computational cost increases steeply. Therefore, one of the authors (U.B.) suggests a new fragmentation algorithm for branched molecules, in which smaller-sized fragments form as in the case of open chain molecules. Our algorithm yields the following bonded fragments for the 24DMP molecule at level 3:
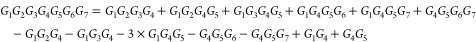
14In the fragmentation in eq 14, fragments formed by the combination of four groups are called the main fragments. The remaining fragments are considered for chemical balance. Hence, we may call them *neutralizing fragments* or *renormalization terms*, reminiscent of the many-body perturbation theory. The logic of the proposed fragmentation approach is to form all possible bonded fragments combining four different groups as in the case of open-chain alkane molecules. Our algorithm produces 6*F*_4_ + 5*F*_3_ + 2*F*_2_, where *F*_*i*_ denotes a fragment formed from *i* different groups, whereas Deev and Collins’s algorithm produces 2*F*_5_ + *F*_3_. Hence, our algorithm yields lower size fragments, while Deev and Collins’ algorithm yields a smaller number of fragments. For high-level CC computations with large basis sets, the size of a fragment is more important than the number of additional small fragments. Moreover, a group can be as small as CH_4_ and H_2_O but can be as large as benzene and naphthalene. Hence, in the case of large groups, such as benzene and naphthalene, decreasing the size of the fragment from *F*_5_ to *F*_4_ is still very important to reduce the cost even though small basis sets are employed. Therefore, our algorithm is more efficient in terms of computational cost and better fits high-level CC methods, such as CCSD(T).

Our new bonded fragmentation algorithm:

1. Let us assume that we are employing bonded level *m* fragmentation. Then, the sizes of our main fragments will be *m* + 1, which means they will include *m* + 1 groups.
2. To form bonded fragments, we need to loop over groups. At the bonded level *m*, one may loop over *m* + 1 groups, which would be a *O*(*n*^*m*+1^) loop, where *n* is the number of groups. However, with the concept of the bonded domain, we can reduce the cost dramatically. Hence, we just run a single loop over groups. For each group (*G*_*i*_), we get bonded domain *BD*_*i*_, which includes *G*_*j*_ groups (*j* ≠ *i*). Then, for each *G*_*j*_ group, we get bonded domain *BD*_*j*_. Finally, we form the union of bonded domains (UBD). Please note that each bonded domain may include just a few groups, in the case of alkanes as many as four. For example, UBD may include a maximum of 16 groups at level 3. Since some groups may be present simultaneously in different BDs, the actual size of UBD is much smaller.
3. Once we form UBD, we loop over the elements of UBD and form all possible main bonded fragments of size *m* + 1. The groups of the fragments which are formed should be connected in the original molecule.
4. Repetitive groups, in the form of the largest possible fragments, in the main bonded fragments are added to the list of bonded fragments with appropriate negative coefficients. For example, at level 3, our main fragments include four groups (*F*_4_). Hence, we first search for repeating three groups (*F*_3_) that are connected in the bonded list. If we find any *F*_3_, then we add them to the list with appropriate negative coefficients. Then, we repeat this procedure for *F*_2_ fragments and finally for groups. The mentioned negative coefficients are obtained following the chemical balance rules.

### 2.5 Modified Nonbonded Algorithms

In the original SMF approach, Collins and co-workers present a simple and effective way to consider nonbonded interactions.^44,55−63^ In Collins and co-workers’ NB approach, only two-body interactions are considered, which may be called the NB level 1 fragmentation. For example, for the linear C_7_H_16_ molecule, the bonded and NB fragments can be written as follows:

Bonded fragments:
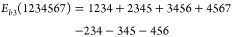
15where each *G*_*i*_ group is represented by the *i* symbol, for example, 234 = *G*_2_*G*_3_*G*_4_.

NB fragments:
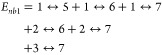
16where *i* ↔ *j* denotes the NB interaction between the *i*th and *j*th groups.

However, for accurate treatment of the NB interactions, one needs to consider larger contributions than two-body interactions. In a 2009 study, Addicoat and Collins reported an improved algorithm for the NB interactions using a level–level approach in addition to a three-body expansion method.^86^

Even though Addicoat and Collins^86^ level–level approach is an improvement over two-body expansion, it includes a limited number of higher terms. Furthermore, Addicoat and Collins^86^ three-body expansion approach yields a very large number of fragments. Hence, we propose a modified NB algorithm, which includes all three-body terms, while lower level interactions come from the renormalization terms. Our modified algorithm is obtained employing our NB cutoff approaches to Addicoat and Collins’ three-body expansion method. For example, for the *n*-heptane NB fragmentation scheme yields
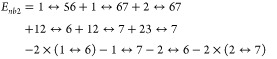
17In our algorithm, we first build all three-body terms, then we investigate all subunits and cancel the repeating terms.

Our nonbonded fragmentation algorithm is as follows::

1. The distant groups that are not present together in the bonded fragments are considered in nonbonded fragments.
2. At the nonbonded level 1, we form “dimers” of the capped groups (*G*_*i*_ ↔ *G*_*j*_) that are not simultaneously present in bonded fragments. Of course, the distance between these two groups should be within the nonbonded cutoff limits. Otherwise, they will be disregarded.
3. In nonbonded level 2, we form three-body complexes (3BCs). Each 3BC is formed between a group and a fragment of two groups (*G*_*i*_ ↔ *F*_2_). The *F*_2_ fragments can be obtained by bonded level 1 fragmentation. Of course, the *G*_*i*_ group and the *F*_2_ fragment should not be simultaneously present in bonded fragments. Next, we inspect these 3BCs for repeating “dimers” of groups. If we find any repeating “dimer”, then we add them (dimers of capped groups) to the list with appropriate negative coefficients. The mentioned negative coefficients are again obtained following the chemical balance rules. As in the case of nonbonded level 1, we consider nonbonded fragments within the nonbonded cutoff limits.

Even though our nonbonded level 2 (NB2) appears to be identical to the three-body expansion approach of Addicoat and Collins,^86^ our algorithm yields a dramatically reduced number of fragments compared to Addicoat and Collins because of employed nonbonded cutoff limits. For example, for the C_70_H_142_ molecule, our algorithm yields 1540 NB fragments, while it would be ∼4 times larger in the case of the three-body expansion approach of Addicoat and Collins.

## 3 Results and Discussion

Results from the HF, MP2, CCSD, CCSD(T), LSSMF(3,1)-HF, LSSMF(3,1)-MP2, LSSMF(3,1)-CCSD, and LSSMF(3,1)-CCSD(T) methods were obtained for a set of alkanes, C_*n*_H_2*n*+2_ (*n* = 6–10), for comparison of the absolute energies. Further, for a larger system, C_*n*_H_2*n*+2_ (*n* = 50–70), results from MP2 and LSSMF-MP2 are compared for the total energies. For the alkanes, Dunning’s correlation-consistent polarized valence double, triple, and quadruple-ζ basis sets (cc-pVDZ, cc-pVTZ, and cc-pVQZ) were employed with the frozen core approximation.^89,90^ The density-fitting approach was used for LSSMF methods considered.^24,74,78,79^ For the cc-pVXZ primary basis sets, cc-pVXZ-JKFIT^91^ and cc-pVXZ-RI^92^ auxiliary basis sets were employed for reference and correlation energies, respectively. Geometries of the C_*n*_H_2*n*+2_ structures considered were optimized at the B3LYP/cc-pVDZ and universal force field (UFF)^93^ levels for *n* = 6–10 and *n* = 50–70, respectively.

Previous studies demonstrated that the accuracies of level 1 and level 2 approaches are not satisfactory for bonded fragments, and level 3 should be used at least.^44,60^ Hence, in this study, we consider levels 3–6 for bonded fragments, while we consider levels 1 and 2 for nonbonded fragments.

### 3.1 S142 Set

**Figure 2:**
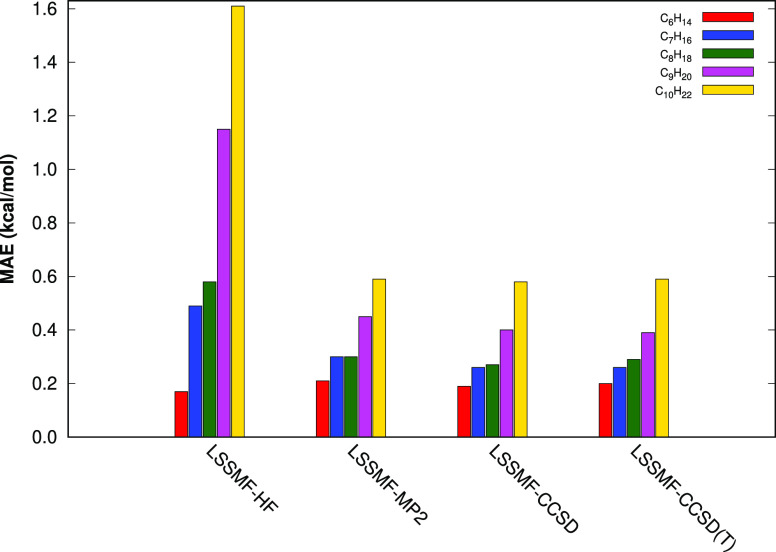
Mean absolute errors in the total energies of the C_*n*_H_2*n*+2_ (*n* = 6–10) isomers for the LSSMF(3,1)-HF, LSSMF(3,1)-MP2, LSSMF(3,1)-CCSD, and LSSMF(3,1)-CCSD(T) methods with respect to canonical methods. All computations are performed with the cc-pVDZ basis set and with the Δ_*nb*_ = 10.0 Å.

To assess the accuracy of the LSSMF(3,1) approach with respect to the canonical methods, we consider a set of alkanes, C_*n*_H_2*n*+2_ (*n* = 6–10), which includes 142 molecules, denoted by S142. For the first step of our assessment, we choose a safe cutoff value for nonbonded interactions: Δ_*nb*_ = 10.0 Å. In the next section, effects of different Δ_*nb*_ values are evaluated. Mean absolute errors (MAEs) of the LSSMF(3,1)-HF, LSSMF(3,1)-MP2, LSSMF(3,1)-CCSD, and LSSMF(3,1)-CCSD(T) methods with respect to canonical methods are depicted in Figure 2. For the C_*n*_H_2*n*+2_ set, total energies from MP2, CCSD, CCSD(T), LSSMF(3,1)-MP2, LSSMF(3,1)-CCSD, and LSSMF(3,1)-CCSD(T) methods and percentages of the LSSMF energies with respect to the canonical methods are reported in Tables S1–S6.

For the C_10_H_22_ isomers, which are the largest member of test set considered, total energies from MP2, CCSD, CCSD(T), LSSMF(3,1)-MP2, LSSMF(3,1)-CCSD, and LSSMF(3,1)-CCSD(T) methods and percentages of the LSSMF energies with respect to the canonical methods are reported in Tables S5 and S6. For the correlated methods, the percent coverage values are in 99.9990%–100.0005%, while that of LSSMF(3,1)-HF is in 99.9978%–100.0001%. Hence, all considered LSSMF methods cover a satisfactory portion of the total energy of the full methods. The MAE values (Figure 2) in total energies are 1.61 [LSSMF(3,1)-HF], 0.59 [LSSMF(3,1)-MP2], 0.58 [LSSMF(3,1)-CCSD], and 0.59 [LSSMF(3,1)-CCSD(T)] kcal mol^–1^. Further, the Δ_*max*_ values for total energies are 5.30 [LSSMF(3,1)-HF], 2.56 [LSSMF(3,1)-MP2], 2.27 [LSSMF(3,1)-CCSD], and 2.01 [LSSMF(3,1)-CCSD(T)] kcal mol^–1^. Hence, considering both error measures, MAE and Δ_*max*_, the results of the correlated LSSMF methods are in good agreement with the canonical methods. These results demonstrate that the high-level electron correlation methods are less prone to fragmentation errors since the dynamical electron correlation is an local phenomenon. Considering the results obtained for the whole alkane set, one can safely rely on the LSSMF-CC methods for high-accuracy studies in large-sized chemical systems, where the canonical methods are not computationally affordable.

### 3.2 Cutoff

**Figure 3:**
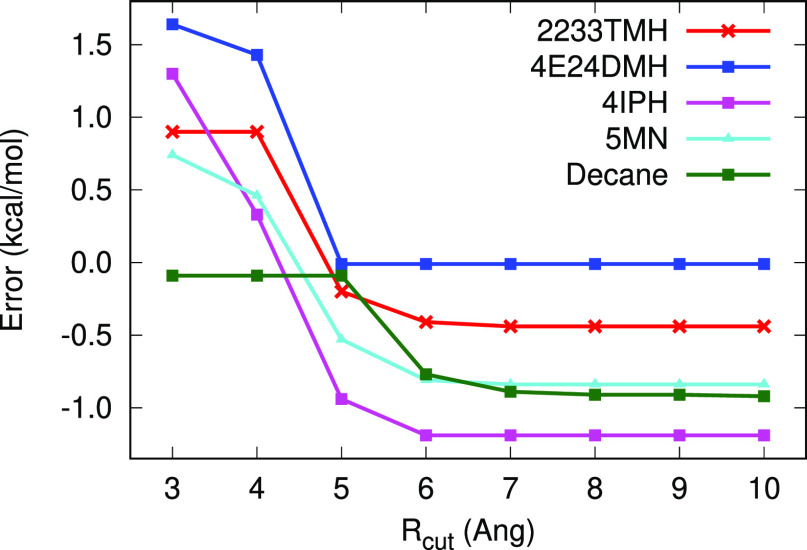
Errors of the LSSMF(3,1)-CCSD(T) method with respect to the full method with different cutoff distances for 2,2,3,3-tetramethylhexane (2233TMH), 4-ethyl-2,4-dimethylhexane (4E24DMH), 4-isopropylheptane (4IPH), 5-methylnonane (5MN), and *n*-decane (decane) molecules. All computations are performed with the cc-pVDZ basis set.

In the second step of our assessment of the LSSMF approaches, we investigate the effect of nonbonded cutoff tolerances on the accuracy. For this purpose, we consider five isomers of C_10_H_22_: 2,2,3,3-tetramethylhexane (2233TMH), 4-ethyl-2,4-dimethylhexane (4E24DMH), 4-isopropylheptane (4IPH), 5-methylnonane (5MN), and *n*-decane (decane). For these molecules, the total energies of the LSSMF(3,1)-CCSD(T) approach are computed with Δ_*nb*_ = 3–10 Å. The errors at each Δ_*nb*_ value with respect to full methods are depicted in Figure 3. Our results indicate that the maximum error is generally obtained at 3 Å, as expected, and errors are kept constant starting with 6 Å. In the case of the *n*-decane molecule, we obtain the lowest errors at Δ_*nb*_ = 3 Å. The reason why the lowest error is obtained at the shortest distance is because the *n*-decane molecule bonded energy is closer to CCSD(T) energy compared with the total LSSMF energy, which covers 100.0005% of the CCSD(T) energy. In other words, adding more nonbonded contribution, by increasing Δ_*nb*_, one obtains lower energies compared with CCSD(T). Overall, even though we use Δ_*nb*_ = 10 Å throughout this study, a Δ_*nb*_ value of 6.0 Å appears to be enough for the most purposes, which corresponds to a DCRR value of ∼4.0.

### 3.3 Frozen Natural Orbitals

To further increase the applicability of the LSSMF(3,1)-CCSD(T) approach, we also consider frozen natural orbitals (FNOs).^72,94−97^ The FNO approximation is very helpful to reduce the computational cost of CCSD(T), while it introduces negligible errors with tight enough occupation tolerances, such as 10^–5^. To improve the FNO–CC results, we employ the δ_*MP2*_ correction as suggested by DePrince and Sherrill.^97^ With the FNO approximation, we can consider larger basis sets for the canonical methods; hence, we employ the cc-pVTZ basis set. For the *n*-decane and four lowest energy isomers, we obtain MAE and Δ_*max*_ values of 0.74 and 1.04 kcal mol^–1^, respectively, for the LSSMF(3,1)-FNO–CCSD(T) approach (Tables S7 and S8). Hence, the fragmentation error is tolerable for the FNO–CCSD(T) method, as in the case of CCSD(T).

### 3.4 Basis Set Effects

**Figure 4:**
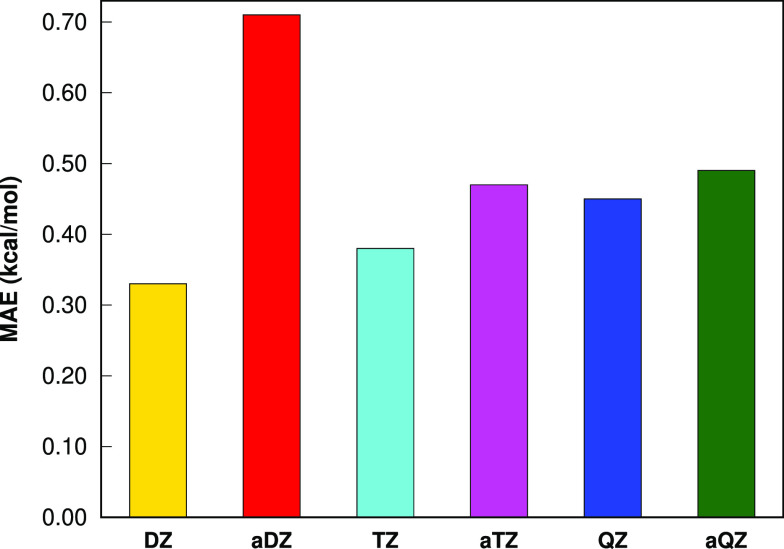
Mean absolute errors in the total energies of three C_7_H_16_ isomers for the LSSMF(3,1)-FNO–CCSD(T) method with respect to FNO–CCSD(T). All computations are performed with the FNO occupation tolerance of 10^–5^ and Δ_*nb*_ = 10.0 Å in the cc-pVDZ (DZ), aug-cc-pVDZ (aDZ), cc-pVTZ (TZ), aug-cc-pVTZ (aTZ), cc-pVQZ (QZ), and aug-cc-pVQZ (aQZ) basis sets.

To investigate the effect of basis sets, we also carry out total energy computations for the LSSMF(3,1)-FNO–CCSD(T) method with cc-pVDZ, cc-pVTZ, and cc-pVQZ basis sets for three C_7_H_16_ isomers. One of these isomers is *n*-heptane, and others are the lowest energy isomers: 2,2,3-trimethylbutane and 2,2-dimethylhexane. The MAE values with respect to FNO–CCSD(T) for different basis sets are depicted in Figure 4. The MAE values are 0.33 (cc-pVDZ), 0.38 (cc-pVTZ), and 0.45 (cc-pVQZ) kcal mol^–1^. Even though there is a slight increase with basis set size, the errors are still at the tolerable magnitudes.

To investigate the effect of diffuse basis sets, we also carried out energy computations for the LSSMF(3,1)-FNO–CCSD(T) method with the aug-cc-pVDZ, aug-cc-pVTZ, and aug-cc-pVQZ basis sets for the same isomers (Figure 4). The MAE values with respect to FNO–CCSD(T) are 0.71 (aug-cc-pVDZ), 0.47 (aug-cc-pVTZ), and 0.49 (aug-cc-pVQZ) kcal mol^–1^. The MAE of aug-cc-pVDZ is almost 2-fold increased compared to cc-pVDZ, while the MAE of triple and quadruple ζ basis sets are only slightly increased compared to cc-pVTZ and cc-pVQZ. Nevertheless, the errors are still at tolerable magnitudes.

### 3.5 L12 Set

To further investigate the performance of the LSSMF approach for larger molecules, we consider the L12 set (Table S9), which consists of 12 large molecules including 50–70 carbon atoms. For the L12 set, conventional CC computations are not computationally feasible. Hence, for this set, we investigate the errors of LSSMF-MP2 with respect to the canonical MP2 for absolute energies. For the L12 set, the cc-pVDZ basis set is employed. For the S142 set, bonded level 3 is only considered because higher levels covers either the whole molecules or a large portion of them. Hence, for the L12 set, we explore the effect of higher levels. For the L12 set, bonded levels 3–6 and nonbonded levels 1 and 2 are considered. For each combination of bonded and nonbonded levels, the cutoff values of 7.5 and 10.0 Å are employed for nonbonded interactions, respectively.

For the C_50_H_102_– C_70_H_142_ molecules, total energies from HF, MP2, LSSMF-HF, and LSSMF-MP2 methods and percentages of the LSSMF energies with respect to the canonical methods are reported in Tables S10–S25. For the entire set, the percent coverage values are in 99.9983%–100.0002% and 100.0000%–100.0008% for LSSMF-HF and LSSMF-MP2, respectively. Hence, both LSSMF methods cover large portions of the total energies of the corresponding canonical methods.

**Figure 5:**
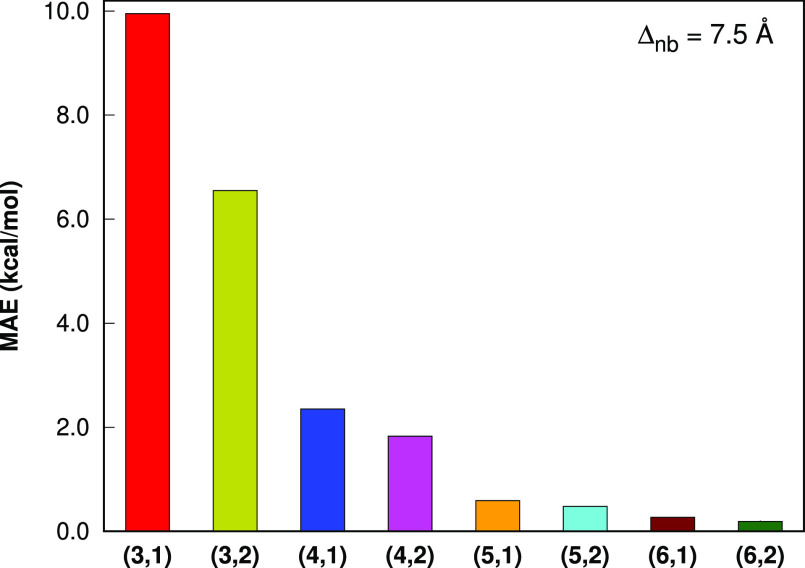
Mean absolute errors in the total energies of the L12 set (the largest member is C_70_H_142_) for the LSSMF-HF method with respect to HF. All computations are performed with the Δ_*nb*_ = 7.5 Å in the cc-pVDZ basis sets. The (*m*, *n*) notation indicates the bonded and nonbonded levels, respectively.

**Figure 6:**
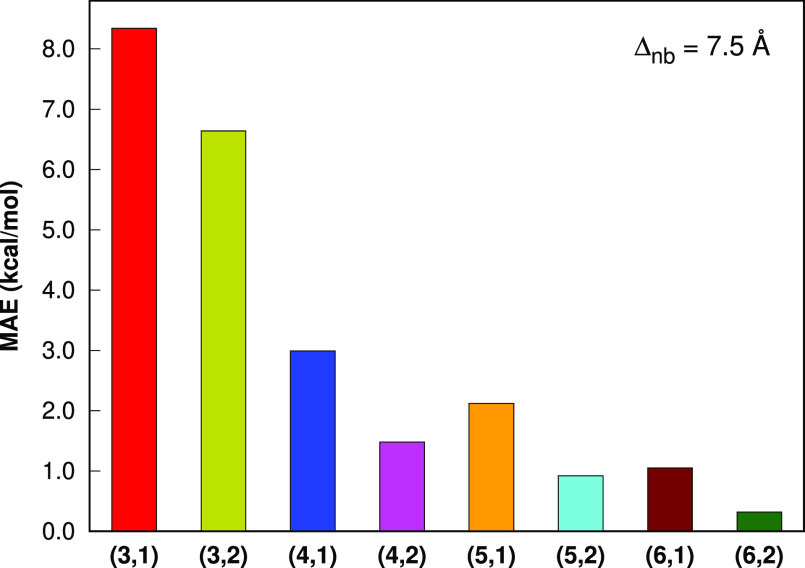
Mean absolute errors in the total energies of the L12 set (the largest member is C_70_H_142_) for the LSSMF-MP2 method with respect to MP2. All computations are performed with the Δ_*nb*_ = 7.5 Å in the cc-pVDZ basis sets. The (*m*, *n*) notation indicates the bonded and nonbonded levels, respectively.

With the nonbonded level 1 and the cutoff value of 7.5 Å, the MAE values (Figure 5) in the LSSMF-HF total energies with respect to HF are 9.95, 2.35, 0.59, and 0.27 kcal mol^–1^ for the bonded levels 3, 4, 5, and 6, respectively. With the nonbonded level 1 and the cutoff value of 7.5 Å, the MAE values (Figure 6) in the LSSMF-MP2 total energies with respect to MP2 are 8.34, 2.99, 2.12, and 1.05 kcal mol^–1^ for the bonded levels 3, 4, 5, and 6, respectively. Hence, at higher bonded levels, the errors of the LSSMF approach decrease systematically. The accuracy of bonded level 6 is substantially better than lower levels considered; there are 7.9-fold reduction in errors compared to level 3, which is advocated to be accurate in previous studies. To further improve our results, we also consider the nonbonded level 2 scheme proposed in this study. With the nonbonded level 2 and the cutoff value of 7.5 Å, the MAE values (Figure 5) in the LSSMF-HF total energies with respect to HF are 6.55, 1.83, 0.48, and 0.19 kcal mol^–1^ for the bonded levels 3, 4, 5, and 6, respectively. With the nonbonded level 2 and the cutoff value of 7.5 Å, the MAE values (Figure 6) in the LSSMF-MP2 total energies with respect to MP2 are 6.64, 1.48, 0.92, and 0.32 kcal mol^–1^ for the bonded levels 3, 4, 5, and 6, respectively. The NB level 2 fragmentation significantly improves upon NB level 1 and provides 1.26-, 2.02-, 2.30-, and 3.28-fold reductions in errors compared with NB level 1 for bonded level 3, 4, 5, and 6, respectively. Thus, the bonded level 6, NB level 2 combination, which may denoted by LSSMF(6,2), provides the best results. Further, it is also noteworthy that the LSSMF(5,2) level provides lower errors compared to LSSMF(6,1). Similarly, the LSSMF(4,2) level is better than LSSMF(5,1). Hence, it appears that instead of going a higher order in the bonded level, it is better to go higher nonbonded level at first.

**Figure 7:**
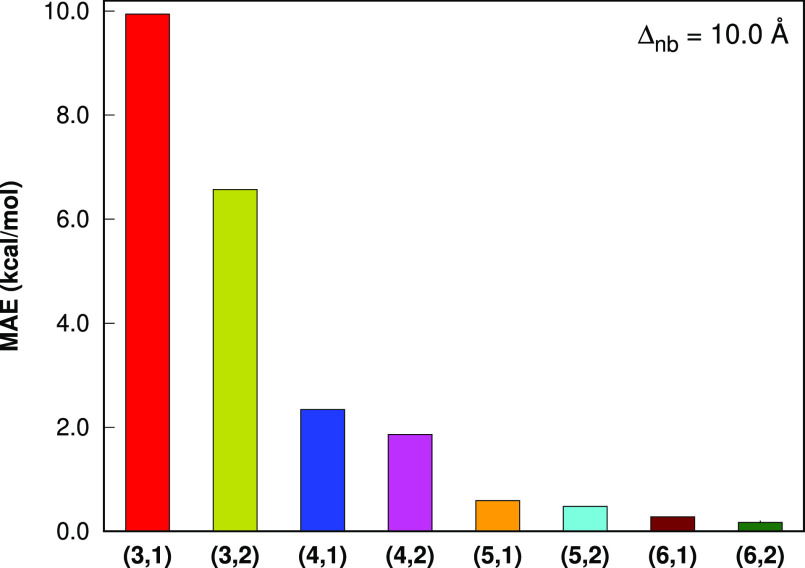
Mean absolute errors in the total energies of the L12 set (the largest member is C_70_H_142_) for the LSSMF-HF method with respect to HF. All computations are performed with the Δ_*nb*_ = 10.0 Å in the cc-pVDZ basis sets. The (*m*, *n*) notation indicates the bonded and nonbonded levels, respectively.

**Figure 8:**
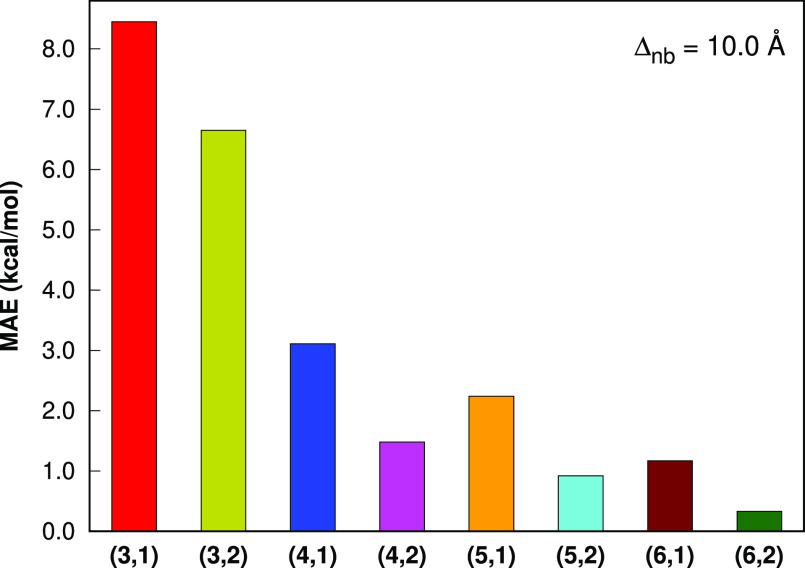
Mean absolute errors in the total energies of the L12 set (the largest member is C_70_H_142_) for the LSSMF-MP2 method with respect to MP2. All computations are performed with the Δ_*nb*_ = 10.0 Å in the cc-pVDZ basis sets. The (*m*, *n*) notation indicates the bonded and nonbonded levels, respectively.

With the nonbonded level 1 and the cutoff value of 10.0 Å, the MAE values (Figure 7) in the LSSMF-HF total energies with respect to HF are 9.94, 2.34, 0.59, 0.28 kcal mol^–1^ for the bonded levels 3, 4, 5, and 6, respectively. With the nonbonded level 1 and the cutoff value of 10.0 Å, the MAE values (Figure 8) in the LSSMF-MP2 total energies with respect to MP2 are 8.45, 3.11, 2.24, 1.17 kcal mol^–1^ for the bonded levels 3, 4, 5, and 6, respectively. With the nonbonded level 2 and the cutoff value of 10.0 Å, the MAE values (Figure 7) in the LSSMF-HF total energies with respect to HF are 6.57, 1.86, 0.48, 0.17 kcal mol^–1^ for the bonded levels 3, 4, 5, and 6, respectively. With the nonbonded level 2 and the cutoff value of 10.0 Å, the MAE values (Figure 8) in the LSSMF-MP2 total energies with respect to MP2 are 6.55, 1.48, 0.92, 0.33 kcal mol^–1^ for the bonded levels 3, 4, 5, and 6, respectively. These results are virtually the same as the previous results obtained for the cutoff value of 7.5 Å, which again demonstrates that our cutoff tolerance is accurate enough. Overall, our results demonstrate that the LSSMF(6,2) level approaches to the canonical method. Therefore, one may rely on the LSSMF methods for high-accuracy studies in large-sized chemical systems, where the canonical methods are not computationally feasible.

### 3.6 Timing

In our LSSMF implementation, we form groups, bonded, and nonbonded fragments at first; then, we write all fragment input files to disk. In the third step, we simultaneously submit all fragment jobs to our Linux clusters. Finally, we collect the energy values from output files, merge them, and compute the final LSSMF energy. Hence, our implementation is naturally parallel. The fragment formation procedure is the fastest step (step 1). We can form all fragments just in a few minutes owing to our efficient fragmentation algorithm. Writing fragment input files generally takes several minutes (step 2). Hence, the cost of overall computation is dependent on the cost of CC jobs (step 3), which is dependent on the number of cores that are available.

**Figure 9:**
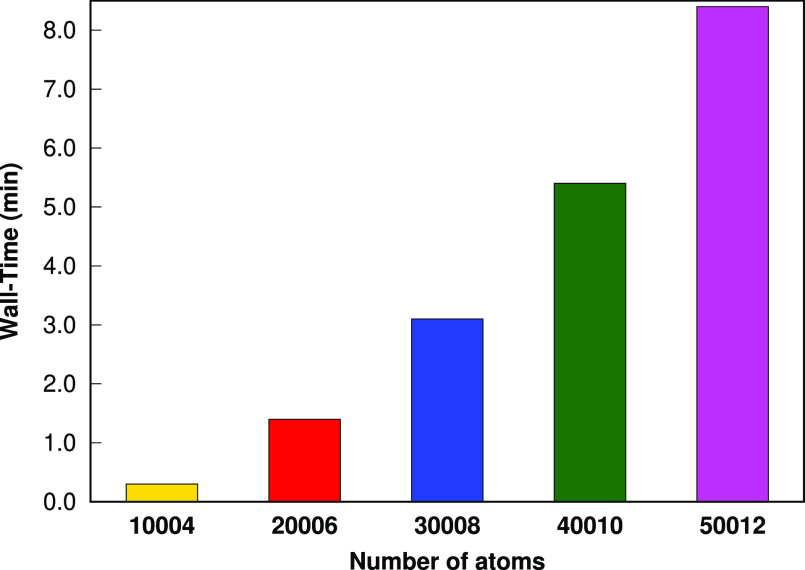
Total wall time (in min) for the LSSMF(3,1) code for a C_*n*_H_2*n*+2_ set. All procedures were performed on a single node (1 core) Intel(R) Xeon(R) Gold 5218 CPU *@* 2.30 GHz computer.

To illustrate the efficiency of our fragmentation algorithm, we consider a set of alkanes, which includes 10,004–50,012 atoms. Total wall times (in min) for the LSSMF(3,1) code (step 1 + step 2) for the C_*n*_H_2*n*+2_ (*n* = 3334; 6668; 10,002; 13,336; 16,670) set are depicted in Figure 9. For the largest member of the alkanes set considered, C_16670_H_33342_, the total time for the LSSMF code is just 8.4 min on a single node (1 core) computer. Hence, our LSSMF code is very efficient to form fragments and prepare necessary input files.

To illustrate the efficiency and applicability of the LSSMF(3,1)-CCSD(T) approach, we consider the C_3334_H_6670_ molecule, which includes 10,004 atoms. For the C_3334_H_6670_ molecule, the LSSMF(3,1)-CCSD(T)/cc-pVTZ energy computation is performed in a Linux cluster with 100 nodes, 4 cores, and 5 GB of memory provided to each node. In this system, the total wall time for energy computation is ∼24 h, which indicates that the introduced method is extremely efficient. As a second example, we consider a biomolecular complex (PDB code: 1GLA), which includes 10,488 atoms, to illustrate the efficiency the LSSMF approach. For 1GLA, the LSSMF(3,1)-FNO–CCSD(T)/cc-pVTZ energy computation with Δ_*nb*_ = 5 Å is performed in a cluster with 50 nodes (each node has 8 cores, 64 GB of memory, and Xeon Scalable 6148 2.40 GHz CPU). If this chemical system would run as a whole molecule, there would be 231,408 basis functions. At the LSSMF(3,1) level, 3170 groups and 11,445 bonded and 62,716 nonbonded fragments are formed for 1GLA. For the largest fragment, there are only 736 basis functions. The LSSMF(3,1)-FNO–CCSD(T) energy of the molecule is −267,117.064554 hartree (with the FNO occupation tolerance of 10^–4^). This computation is completed in ∼7 days, which shows the efficiency of our LSSMF method. The number of atoms are similar for the biomolecular complex and the linear alkane considered, C_3334_H_6670_. However, the biomolecular complex yields significantly larger fragments due to aromatic bonds in amino acids. Therefore, we observe different computational times.

## 4 Conclusions

In this research, efficient implementations of linear-scaling coupled-cluster methods, which employ the systematic molecular fragmentation approach, have been reported. For the branched molecules, a new fragmentation algorithm, which yields smaller-sized fragments compare with previous studies, has been introduced. The new linear-scaling SMF algorithm is denoted by LSSMF. Performances of the developed LSSMF-CC approaches, such as LSSMF-CCSD and LSSMF-CCSD(T), have been compared with their canonical versions for a set of alkane molecules, C_*n*_H_2*n*+2_ (*n* = 6–10), which includes 142 molecules. Our results demonstrate that the LSSMF approach introduces negligible errors compared with the canonical methods. For the alkanes set, the MAE values are between 0.19 and 0.58 and 0.20 and 0.59 kcal mol^–1^ for the LSSMF(3,1)-CCSD and LSSMF(3,1)-CCSD(T) methods, respectively. A similar performance has been observed in the case of the frozen natural orbitals-based CCSD(T) approach [LSSMF(3,1)-FNO–CCSD(T)]. Further, we investigate basis set effects on the LSSMF methods using cc-pVXZ (X = D,T,Q) basis sets. Our results indicate that the performance of the LSSMF(3,1)-FNO–CCSD(T) approach with large basis sets is similar to the small basis set cases.

To further assess the performances of the LSSMF approaches for large molecular systems, we consider the L12 set, which consists of 12 large molecules including 50–70 carbon atoms. For the L12 set, various bonded and nonbonded levels are considered. Our results demonstrate that the combination of bonded level 6 with nonbonded level 2, LSSMF(6,2), yields substantially accurate results for the MP2 method. The MAE value for the LSSMF(6,2)-MP2 method with respect to MP2 is 0.32 kcal mol^–1^ with the cutoff value of 7.5 Å. The LSSMF(6,2) approach yields more than a 26-fold reduction in errors compared with the LSSMF(3,1) approach. Hence, we obtain dramatic improvements over Collins’ original SMF approach.^59^

To illustrate the efficiency and applicability of the LSSMF approach, we consider an alkane molecule with 10,004 atoms at first. For the C_3334_H_6670_ molecule, the LSSMF(3,1)-CCSD(T)/cc-pVTZ energy computation, on a Linux cluster with 100 nodes, 4 cores, and 5 GB of memory provided to each node, is performed just in ∼24 h. Furthermore, we consider a biomolecular complex (PDB code: 1GLA), which includes 10,488 atoms, as a second test for assessment of the efficiency of LSSMF. The LSSMF(3,1)-FNO–CCSD(T)/cc-pVTZ single point energy computation is completed in ∼7 days for the biomolecular complex. Even though the number of atoms appears to be similar, the biomolecular complex includes larger fragments compared to the linear alkane considered, which accounts to the difference in wall time reported. Hence, our results demonstrate that the LSSMF-CC approaches are very efficient.

Our results demonstrate the LSSMF(6,2) level approaches to the canonical method. Therefore, one may rely on the LSSMF methods for high-accuracy studies in large-sized chemical systems, where the canonical methods are computationally prohibitive. Overall, we conclude that the LSSMF approach is promising for applications of electron correlation methods in large-scale chemical systems where canonical methods are computationally prohibitive.

## Supporting Information Available

Optimized geometries for the molecules considered and absolute energies for the chemical systems considered (PDF)

## References

[ref1] Bartlett R. J. Many-Body Perturbation Theory and Coupled Cluster Theory for Electron Correlation in Molecules. Annu. Rev. Phys. Chem. 1981, 32, 359–401. 10.1146/annurev.pc.32.100181.002043.

[ref2] Bartlett R. J. Coupled-Cluster Approach to Molecular Structure and Spectra: A Step Toward Predictive Quantum Chemistry. J. Phys. Chem. 1989, 93, 1697–1708. 10.1021/j100342a008.

[ref3] Crawford T. D.; Schaefer H. F. An Introduction to Coupled Cluster Theory for Computational Chemists. Rev. Comp. Chem. 2000, 14, 33–136. 10.1002/9780470125915.ch2.

[ref4] Bartlett R. J.; Musiał M. Coupled-Cluster Theory in Quantum Chemistry. Rev. Mod. Phys. 2007, 79, 291–352. 10.1103/RevModPhys.79.291.

[ref5] Bartlett R. J. Coupled-Cluster Theory and Its Equation-of-Motion Extensions. WIREs Comput. Mol. Sci. 2012, 2, 126–138. 10.1002/wcms.76.

[ref6] Purvis G. D.; Bartlett R. J. A Full Coupled-Cluster Singles and Doubles Model: The Inclusion of Disconnected Triples. J. Chem. Phys. 1982, 76, 1910–1918. 10.1063/1.443164.

[ref7] Bartlett R. J.; Sekino H.; Purvis G. D. Comparison of MBPT and Coupled-cluster Methods with Full CI. Importance of Triplet Excitation and Infinite Summations. Chem. Phys. Lett. 1983, 98, 66–71. 10.1016/0009-2614(83)80204-8.

[ref8] Lee Y. S.; Kucharski S. A.; Bartlett R. J. A Coupled Cluster Approach with Triple Excitations. J. Chem. Phys. 1984, 81, 5906–5912. 10.1063/1.447591.

[ref9] Pople J. A.; Head-Gordon M.; Raghavachari K. Quadratic Configuration Interaction. A General Technique for Determining Electron Correlation Energies. J. Chem. Phys. 1987, 87, 5968–5975. 10.1063/1.453520.

[ref10] Raghavachari K.; Trucks G. W.; Pople J. A.; Head-Gordon M. A Fifth-Order Perturbation Comparison of Electronic Correlation Theories. Chem. Phys. Lett. 1989, 157, 479–483. 10.1016/S0009-2614(89)87395-6.

[ref11] Bartlett R. J.; Watts J. D.; Kucharski S. A.; Noga J. Non-Iterative Fifth-Order Triple and Quadruple Excitation Energy Corrections in Correlated Methods. Chem. Phys. Lett. 1990, 165, 513–522. 10.1016/0009-2614(90)87031-L.

[ref12] Scuseria G. E.; Lee T. J. Comparison of Coupled-Cluster Methods which Include the Effects of Connected Triple Excitations. J. Chem. Phys. 1990, 93, 5851–5855. 10.1063/1.459684.

[ref13] Scuseria G. E.; Hamilton T. P.; Schaefer H. F. An Assessment for the Full Coupled Cluster Method Including All Single, Double, and Triple Excitations: The Diatomic Molecules LiH, Li_2_, BH, LiF, C_2_, BeO, CN^+^, BF, NO^+^, and F_2_. J. Chem. Phys. 1990, 92, 568–573. 10.1063/1.458407.

[ref14] Urban M.; Noga J.; Cole S. J.; Bartlett R. J. Towards a Full CCSDT Model for Electron Correlation. J. Chem. Phys. 1985, 83, 4041–4046. 10.1063/1.449067.

[ref15] Lee T. J.; Scuseria G. E. In Quantum Mechanical Electronic Structure Calculations with Chemical Accuracy; Langhoff S. R., Ed.; Kluwer Academic: Dordrecht, 1995; pp 47–108.

[ref16] Watts J. D.; Stanton J. F.; Bartlett R. J. A Benchmark Coupled-Cluster Single, Double, and Triple Excitation (CCSDT) Study of the Structure and Harmonic Vibrational Frequencies of the Ozone Molecule. Chem. Phys. Lett. 1991, 178, 471–474. 10.1016/0009-2614(91)87004-U.

[ref17] Scuseria G. E. Ab Initio Theoretical Predictions of the Equilibrium Geometries of C_60_, C_60_H_60_ and C_60_F_60_. Chem. Phys. Lett. 1991, 176, 423–427. 10.1016/0009-2614(91)90231-W.

[ref18] Gauss J.; Lauderdale W. J.; Stanton J. F.; Watts J. D.; Bartlett R. J. Analytic Energy Gradients for Open-Shell Coupled-Cluster Singles and Doubles (CCSD) Calculations Using Restricted Open-Shell Hartree–Fock (ROHF) Reference Functions. Chem. Phys. Lett. 1991, 182, 207–215. 10.1016/0009-2614(91)80203-A.

[ref19] Watts J. D.; Bartlett R. J. Coupled-Cluster Calculations on the C_2_ molecule and the C_2_^+^ and C_2_^–^ Molecular Ions. J. Chem. Phys. 1992, 96, 6073–6084. 10.1063/1.462649.

[ref20] Thomas J. R.; DeLeeuw B. J.; Vacek G.; Crawford T. D.; Yamaguchi Y.; Schaefer H. F. The Balance Between Theoretical Method and Basis Set Quality: A Systematic Study of Equilibrium Geometries, Dipole Moments, Harmonic Vibrational Frequencies, and Infrared Intensities. J. Chem. Phys. 1993, 99, 403–416. 10.1063/1.465764.

[ref21] Watts J. D.; Gauss J.; Bartlett R. J. Coupled-Cluster Methods With Noniterative Triple Excitations For Restricted Open-Shell Hartree–Fock And Other General Single Determinant Reference Functions. Energies And Analytical Gradients. J. Chem. Phys. 1993, 98, 8718–8733. 10.1063/1.464480.

[ref22] Crawford T. D.; Schaefer H. F. A Comparison of Two Approaches to Perturbational Triples Corrections to the Coupled-Cluster Singles and Doubles Method for High-Spin Open-Shell Systems. J. Chem. Phys. 1996, 104, 6259–6264. 10.1063/1.471287.

[ref23] Crawford T. D.; Lee T. J.; Schaefer H. F. A New Spin-Restricted Perturbative Triple Excitation Correction for Coupled Cluster Theory. J. Chem. Phys. 1997, 107, 7943–7950. 10.1063/1.475081.

[ref24] Bozkaya U.; Sherrill C. D. Analytic Energy Gradients for The Coupled-Cluster Singles and Doubles with Perturbative Triples Method with the Density-Fitting Approximation. J. Chem. Phys. 2017, 147, 044104–044114. 10.1063/1.4994918. 28764345

[ref25] Saebo S.; Tong W.; Pulay P. Efficient Elimination of Basis Set Superposition Errors by the Local Correlation Method: Accurate Ab Initio Studies of The Water Dimer. J. Chem. Phys. 1993, 98, 2170–2175. 10.1063/1.464195.

[ref26] Saebo S.; Pulay P. Local Treatment of Electron Correlation. Annu. Rev. Phys. Chem. 1993, 44, 213–236. 10.1146/annurev.pc.44.100193.001241.

[ref27] Schütz M.; Hetzer G.; Werner H.-J. Low-Order Scaling Local Electron Correlation Methods. I. Linear Scaling Local MP2. J. Chem. Phys. 1999, 111, 5691–5705. 10.1063/1.479957.

[ref28] Hampel C.; Werner H.-J. Local Treatment of Electron Correlation in Coupled Cluster Theory. J. Chem. Phys. 1996, 104, 6286–6297. 10.1063/1.471289.

[ref29] Schütz M.; Werner H.-J. Low-Order Scaling Local Electron Correlation Methods. IV. Linear Scaling Local Coupled-Cluster (LCCSD). J. Chem. Phys. 2001, 114, 661–681. 10.1063/1.1330207.

[ref30] Rauhut G.; Pulay P.; Werner H.-J. Integral Transformation with Low-Order Scaling for Large Local Second-Order Møller-Plesset Calculations. J. Comput. Chem. 1998, 19, 1241–1254. 10.1002/(SICI)1096-987X(199808)19:11<1241::AID-JCC4>3.0.CO;2-K.

[ref31] Schütz M.; Werner H.-J. Local Perturbative Triples Correction (T) with Linear Cost Scaling. Chem. Phys. Lett. 2000, 318, 370–378. 10.1016/S0009-2614(00)00066-X.

[ref32] Neese F.; Hansen A.; Liakos D. G. Efficient and Accurate Approximations to the Local Coupled Cluster Singles Doubles Method Using a Truncated Pair Natural Orbital Basis. J. Chem. Phys. 2009, 131, 064103 10.1063/1.3173827. 19691374

[ref33] Riplinger C.; Neese F. An Efficient and Near Linear Scaling Pair Natural Orbital Based Local Coupled Cluster Method. J. Chem. Phys. 2013, 138, 034106 10.1063/1.4773581. 23343267

[ref34] Riplinger C.; Sandhoefer B.; Hansen A.; Neese F. Natural Triple Excitations in Local Coupled Cluster Calculations with Pair Natural Orbitals. J. Chem. Phys. 2013, 139, 134101 10.1063/1.4821834. 24116546

[ref35] Rolik Z.; Szegedy L.; Ladjánszki I.; Ladóczki B.; Kállay M. An Efficient Linear-scaling CCSD(T) Method Based on Local Natural Orbitals. J. Chem. Phys. 2013, 139, 094105 10.1063/1.4819401. 24028100

[ref36] Li S.; Ma J.; Jiang Y. Linear Scaling Local Correlation Approach for Solving The Coupled Cluster Equations of Large Systems. J. Comput. Chem. 2002, 23, 237–244. 10.1002/jcc.10003. 11924736

[ref37] Li W.; Piecuch P.; Gour J. R.; Li S. Local Correlation Calculations Using Standard And Renormalized Coupled-Cluster Approaches. J. Chem. Phys. 2009, 131, 114109 10.1063/1.3218842. 19778102

[ref38] Li W.; Piecuch P. Improved Design of Orbital Domains within the Cluster-in-Molecule Local Correlation Framework: Single-Environment Cluster-in-Molecule Ansatz and Its Application to Local Coupled-Cluster Approach with Singles and Doubles. J. Phys. Chem. A 2010, 114, 8644–8657. 10.1021/jp100782u. 20373794

[ref39] Li W.; Piecuch P. Multilevel Extension of the Cluster-in-Molecule Local Correlation Methodology: Merging Coupled-Cluster and Møller-Plesset Perturbation Theories. J. Phys. Chem. A 2010, 114, 6721–6727. 10.1021/jp1038738. 20496942

[ref40] Rolik Z.; Kállay M. A General-Order Local Coupled-Cluster Method Based on the Cluster-in-Molecule Approach. J. Chem. Phys. 2011, 135, 104111 10.1063/1.3632085. 21932880

[ref41] Ziółkowski M.; Jansík B.; Kjærgaard T.; Jørgensen P. Linear Scaling Coupled Cluster Method with Correlation Energy Based Error Control. J. Chem. Phys. 2010, 133, 014107 10.1063/1.3456535. 20614959

[ref42] Eriksen J. J.; Baudin P.; Ettenhuber P.; Kristensen K.; Kjærgaard T.; Jørgensen P. Linear-Scaling Coupled Cluster with Perturbative Triple Excitations: The Divide–Expand–Consolidate CCSD(T) Model. J. Chem. Theory Comput. 2015, 11, 2984–2993. 10.1021/acs.jctc.5b00086. 26575735

[ref43] Gordon M. S.; Fedorov D. G.; Pruitt S. R.; Slipchenko L. V. Fragmentation Methods: A Route to Accurate Calculations on Large Systems. Chem. Rev. 2012, 112, 632–672. 10.1021/cr200093j. 21866983

[ref44] Collins M. A.; Bettens R. P. A. Energy-Based Molecular Fragmentation Methods. Chem. Rev. 2015, 115, 5607–5642. 10.1021/cr500455b. 25843427

[ref45] Raghavachari K.; Saha A. Accurate Composite and Fragment-Based Quantum Chemical Models for Large Molecules. Chem. Rev. 2015, 115, 5643–5677. 10.1021/cr500606e. 25849163

[ref46] Akimov A. V.; Prezhdo O. V. Large-Scale Computations in Chemistry: A Bird’s Eye View of a Vibrant Field. Chem. Rev. 2015, 115, 5797–5890. 10.1021/cr500524c. 25851499

[ref47] Gadre S. R.; Shirsat R. N.; Limaye A. C. Molecular Tailoring Approach for Simulation of Electrostatic Properties. J. Phys. Chem. 1994, 98, 9165–9169. 10.1021/j100088a013.

[ref48] Ganesh V.; Dongare R. K.; Balanarayan P.; Gadre S. R. Molecular Tailoring Approach for Geometry Optimization of Large Molecules: Energy Evaluation and Parallelization Strategies. J. Chem. Phys. 2006, 125, 104109 10.1063/1.2339019. 16999517

[ref49] Sahu N.; Gadre S. R. Molecular Tailoring Approach: A Route for ab Initio Treatment of Large Clusters. Acc. Chem. Res. 2014, 47, 2739–2747. 10.1021/ar500079b. 24798296

[ref50] Kitaura K.; Ikeo E.; Asada T.; Nakano T.; Uebayasi M. Fragment Molecular Orbital Method: An Approximate Computational Method for Large Molecules. Chem. Phys. Lett. 1999, 313, 701–706. 10.1016/S0009-2614(99)00874-X.

[ref51] Mochizuki Y.; Koikegami S.; Nakano T.; Amari S.; Kitaura K. Large Scale MP2 Calculations with Fragment Molecular Orbital Scheme. Chem. Phys. Lett. 2004, 396, 473–479. 10.1016/j.cplett.2004.08.082.

[ref52] Mochizuki Y.; Yamashita K.; Nakano T.; Okiyama Y.; Fukuzawa K.; Taguchi N.; Tanaka S. Higher-Order Correlated Calculations Based on Fragment Molecular Orbital Scheme. Theor. Chem. Acc. 2011, 130, 515–530. 10.1007/s00214-011-1036-3.

[ref53] Zhang D. W.; Zhang J. Z. H. Molecular Fractionation with Conjugate Caps for Full Quantum Mechanical Calculation of Protein–Molecule Interaction Energy. J. Chem. Phys. 2003, 119, 3599–3605. 10.1063/1.1591727.

[ref54] He X.; Zhu T.; Wang X.; Liu J.; Zhang J. Z. H. Fragment Quantum Mechanical Calculation of Proteins and Its Applications. Acc. Chem. Res. 2014, 47, 2748–2757. 10.1021/ar500077t. 24851673

[ref55] Deev V.; Collins M. A. Approximate Ab Initio Energies by Systematic Molecular Fragmentation. J. Chem. Phys. 2005, 122, 154102 10.1063/1.1879792. 15945620

[ref56] Collins M. A.; Deev V. A. Accuracy and Efficiency of Electronic Energies from Systematic Molecular Fragmentation. J. Chem. Phys. 2006, 125, 104104 10.1063/1.2347710. 16999512

[ref57] Collins M. A. Ab Initio Lattice Dynamics of Nonconducting Crystals by Systematic Fragmentation. J. Chem. Phys. 2011, 134, 164110 10.1063/1.3581845. 21528953

[ref58] Frankcombe T. J.; Collins M. A. Potential Energy Surfaces For Gas-surface Reactions. Phys. Chem. Chem. Phys. 2011, 13, 8379–8391. 10.1039/c0cp01843k. 21283905

[ref59] Collins M. A. Systematic Fragmentation of Large Molecules By Annihilation. Phys. Chem. Chem. Phys. 2012, 14, 7744–7751. 10.1039/c2cp23832b. 22373545

[ref60] Collins M. A.; Cvitkovic M. W.; Bettens R. P. A. The Combined Fragmentation and Systematic Molecular Fragmentation Methods. Acc. Chem. Res. 2014, 47, 2776–2785. 10.1021/ar500088d. 24972052

[ref61] Collins M. A. Molecular Forces, Geometries, and Frequencies by Systematic Molecular Fragmentation Including Embedded Charges. J. Chem. Phys. 2014, 141, 094108 10.1063/1.4894185. 25194365

[ref62] Kobayashi R.; Amos R.; Collins M. A. Microsolvation within the Systematic Molecular Fragmentation by Annihilation Approach. J. Phys. Chem. A 2017, 121, 334–341. 10.1021/acs.jpca.6b10919. 28001075

[ref63] Kobayashi R.; Amos R. D.; Reid D. M.; Collins M. A. Application of the Systematic Molecular Fragmentation by Annihilation Method to ab Initio NMR Chemical Shift Calculations. J. Phys. Chem. A 2018, 122, 9135–9141. 10.1021/acs.jpca.8b09565. 30398349

[ref64] Masoumifeshani E.; Korona T. Symmetrized Systematic Molecular Fragmentation Model and Its Application for Molecular Properties. Comput. Theor. Chem. 2021, 1202, 113303 10.1016/j.comptc.2021.113303.

[ref65] Le H.-A.; Tan H.-J.; Ouyang J. F.; Bettens R. P. A. Combined Fragmentation Method: A Simple Method for Fragmentation of Large Molecules. J. Chem. Theory Comput 2012, 8, 469–478. 10.1021/ct200783n. 26596597

[ref66] Li S.; Li W.; Ma J. Generalized Energy-Based Fragmentation Approach and Its Applications to Macromolecules and Molecular Aggregates. Acc. Chem. Res. 2014, 47, 2712–2720. 10.1021/ar500038z. 24873495

[ref67] Huang L.; Massa L.; Karle J. Kernel Energy Method Illustrated with Peptides. Int. J. Quantum Chem. 2005, 103, 808–817. 10.1002/qua.20542.

[ref68] Huang L.; Massa L.; Karle J. Kernel energy method: Application to insulin. Proc. Natl. Acad. Sci. 2005, 102, 12690–12693. 10.1073/pnas.0506378102. 16120673 PMC1200310

[ref69] Mayhall N. J.; Raghavachari K. Molecules-in-Molecules: An Extrapolated Fragment-Based Approach for Accurate Calculations on Large Molecules and Materials. J. Chem. Theory Comput. 2011, 7, 1336–1343. 10.1021/ct200033b. 26610128

[ref70] Mayhall N. J.; Raghavachari K. Many-Overlapping-Body (MOB) Expansion: A Generalized Many Body Expansion for Nondisjoint Monomers in Molecular Fragmentation Calculations of Covalent Molecules. J. Chem. Theory Comput. 2012, 8, 2669–2675. 10.1021/ct300366e. 26592112

[ref71] Richard R. M.; Herbert J. M. A Generalized Many-body Expansion and a Unified View of Fragment-based Methods In Electronic Structure Theory. J. Chem. Phys. 2012, 137, 064113 10.1063/1.4742816. 22897261

[ref72] Bozkaya U.; Ermiş B.; Alagöz Y.; Ünal A.; Uyar A. K. MacroQC 1.0: An Electronic Structure Theory Software for Large-Scale Applications. J. Chem. Phys. 2022, 156, 044801 10.1063/5.0077823. 35105088

[ref73] Bozkaya U. Orbital-Optimized Second-Order Perturbation Theory with Density-Fitting and Cholesky Decomposition Approximations: An Efficient Implementation. J. Chem. Theory Comput. 2014, 10, 2371–2378. 10.1021/ct500231c. 26580757

[ref74] Bozkaya U. Derivation of General Analytic Gradient Expressions for Density-Fitted Post-Hartree-Fock Methods: An Efficient Implementation for the Density-Fitted Second-Order Møller–Plesset Perturbation Theory. J. Chem. Phys. 2014, 141, 124108 10.1063/1.4896235. 25273413

[ref75] Bozkaya U. Analytic Energy Gradients and Spin Multiplicities for Orbital-Optimized Second-Order Perturbation Theory with Density-Fitting Approximation: An Efficient Implementation. J. Chem. Theory Comput. 2014, 10, 4389–4399. 10.1021/ct500634s. 26588136

[ref76] Bozkaya U. Orbital-Optimized MP3 and MP2.5 with Density-Fitting and Cholesky Decomposition Approximations. J. Chem. Theory Comput. 2016, 12, 1179–1188. 10.1021/acs.jctc.5b01128. 26854993

[ref77] Bozkaya U. Orbital-Optimized Linearized Coupled-Cluster Doubles with Density-Fitting and Cholesky Decomposition Approximations: An Efficient Implementation. Phys. Chem. Chem. Phys. 2016, 18, 11362–11373. 10.1039/C6CP00164E. 27056800

[ref78] Bozkaya U.; Sherrill C. D. Analytic Energy Gradients for the Coupled-Cluster Singles and Doubles Method with the Density-Fitting Approximation. J. Chem. Phys. 2016, 144, 174103 10.1063/1.4948318. 27155621

[ref79] Bozkaya U. A Noniterative Asymmetric Triple Excitation Correction for The Density-Fitted Coupled-Cluster Singles and Doubles Method: Preliminary Applications. J. Chem. Phys. 2016, 144, 144108 10.1063/1.4945706. 27083709

[ref80] Bozkaya U. Analytic Energy Gradients for Orbital-Optimized MP3 and MP2.5 with the Density-Fitting Approximation: An Efficient Implementation. J. Comput. Chem. 2018, 39, 351–360. 10.1002/jcc.25122. 29164639

[ref81] Bozkaya U.; Ünal A.; Alagöz Y. Energy and Analytic Gradients for The Orbital-Optimized Coupled-Cluster Doubles Method With The Density-Fitting Approximation: An Efficient Implementation. J. Chem. Phys. 2020, 153, 244115 10.1063/5.0035811. 33380091

[ref82] Alagöz Y.; Ünal A.; Bozkaya U. Efficient Implementations of The Symmetric and Asymmetric Triple Excitation Corrections for The Orbital-Optimized Coupled-Cluster Doubles Method With The Density-Fitting Approximation. J. Chem. Phys. 2021, 155, 114104 10.1063/5.0061351. 34551547

[ref83] Hehre W. J. Ab initio molecular orbital theory. Acc. Chem. Res. 1976, 9, 399–406. 10.1021/ar50107a003.

[ref84] Wheeler S. E.; Houk K. N.; Schleyer P. v. R.; Allen W. D. A Hierarchy of Homodesmotic Reactions for Thermochemistry. J. Am. Chem. Soc. 2009, 131, 2547–2560. 10.1021/ja805843n. 19182999 PMC2711007

[ref85] Hess B. A.; Schaad L. J. Ab Initio Calculation of Resonance Energies. Benzene and Cyclobutadiene. J. Am. Chem. Soc. 1983, 105, 7500–7505. 10.1021/ja00364a600.

[ref86] Addicoat M. A.; Collins M. A. Accurate Treatment of Nonbonded Interactions within Systematic Molecular Fragmentation. J. Chem. Phys. 2009, 131, 104103 10.1063/1.3222639.

[ref87] Cordero B.; Gómez V.; Platero-Prats A. E.; Revés M.; Echeverría J.; Cremades E.; Barragán F.; Alvarez S. Covalent Radii Revisited. Dalton Trans 2008, 2832–2838. 10.1039/b801115j. 18478144

[ref88] Reid D. M.; Collins M. A. Molecular Electrostatic Potentials by Systematic Molecular Fragmentation. J. Chem. Phys. 2013, 139, 184117 10.1063/1.4827020. 24320264

[ref89] Dunning T. H. Gaussian Basis Sets for Use in Correlated Molecular Calculations. I. The Atoms Boron Through Neon and Hydrogen. J. Chem. Phys. 1989, 90, 1007–1023. 10.1063/1.456153.

[ref90] Woon D. E.; Dunning T. H. Gaussian Basis Sets for Use in Correlated Molecular Calculations. V. Core-Valence Basis Sets for Boron through Neon. J. Chem. Phys. 1995, 103, 4572–4585. 10.1063/1.470645.

[ref91] Weigend F. A fully Direct RI-HF Algorithm: Implementation, Optimised Auxiliary Basis Sets, Demonstration of Accuracy and Efficiency. Phys. Chem. Chem. Phys. 2002, 4, 4285–4291. 10.1039/b204199p.

[ref92] Weigend F.; Köhn A.; Hättig C. Efficient Use of the Correlation Consistent Basis Sets in Resolution of the Identity MP2 Calculations. J. Chem. Phys. 2002, 116, 3175–3183. 10.1063/1.1445115.

[ref93] Rappe A. K.; Casewit C. J.; Colwell K. S.; Goddard W. A.; Skiff W. M. UFF, a Full Periodic Table Force Field for Molecular Mechanics and Molecular Dynamics Simulations. J. Am. Chem. Soc. 1992, 114, 10024–10035. 10.1021/ja00051a040.

[ref94] Taube A. G.; Bartlett R. J. Frozen Natural Orbitals: Systematic Basis Set Truncation for Coupled-Cluster Theory. Collect. Czech. Chem. Commun. 2005, 70, 837–850. 10.1135/cccc20050837.

[ref95] Taube A. G.; Bartlett R. J. Frozen Natural Orbital Coupled-Cluster Theory: Forces and Application to Decomposition of Nitroethane. J. Chem. Phys. 2008, 128, 164101 10.1063/1.2902285. 18447415

[ref96] Landau A.; Khistyaev K.; Dolgikh S.; Krylov A. I. Frozen Natural Orbitals for Ionized States within Equation-of-Motion Coupled-Cluster Formalism. J. Chem. Phys. 2010, 132, 014109 10.1063/1.3276630. 20078151

[ref97] DePrince A. E.; Sherrill C. D. Accurate Noncovalent Interaction Energies Using Truncated Basis Sets Based on Frozen Natural Orbitals. J. Chem. Theory Comput. 2013, 9, 293–299. 10.1021/ct300780u. 26589031

