# Supplementary material for: Linear-Scaling Systematic Molecular Fragmentation Approach for Perturbation Theory and Coupled-Cluster Methods
Source: J Chem Theory Comput. 2022 Aug 16;18(9):5349–59. doi: 10.1021/acs.jctc.2c00587 (PMC9476663; doi:10.1021/acs.jctc.2c00587)
Supplement: Supplementary file 1 — ct2c00587_si_001.pdf [file ct2c00587_si_001.pdf]

Supporting Information

Linear-Scaling Systematic Molecular Fragmentation Approach for Perturbation Theory and Coupled-Cluster Methods

Uğur Bozkaya,* and Betül Ermiş

Department of Chemistry, Hacettepe University, Ankara 06800, Turkey

E-mail: ugur.bozkaya@hacettepe.edu.tr

*To whom correspondence should be addressed

List of Tables

S1 Total energies (in hartrees) from MP2, CCSD, CCSD(T), LSSMF(3,1)-MP2, LSSMF(3,1)-CCSD, and LSSMF(3,1)-CCSD(T) methods, percentages of the LSSMF energies, and the MAE and Δ_{max} values (in kcal mol^{-1}) of the LSSMF approaches with respect to the canonical methods (Full) for the C_6H_{14} isomers. All computations are performed with the cc-pVDZ basis set and with Δ_{nb} = 10.0 Å. S7

S2 Total energies (in hartrees) from MP2, CCSD, CCSD(T), LSSMF(3,1)-MP2, LSSMF(3,1)-CCSD, and LSSMF(3,1)-CCSD(T) methods, percentages of the LSSMF energies, and the MAE and Δ_{max} values (in kcal mol^{-1}) of the LSSMF approaches with respect to the canonical methods (Full) for the C_7H_{16} isomers. All computations are performed with the cc-pVDZ basis set and with Δ_{nb} = 10.0 Å. S8

S3 Total energies (in hartrees) from MP2, CCSD, CCSD(T), LSSMF(3,1)-MP2, LSSMF(3,1)-CCSD, and LSSMF(3,1)-CCSD(T) methods, percentages of the LSSMF energies, and the MAE and Δ_{max} values (in kcal mol^{-1}) of the LSSMF approaches with respect to the canonical methods (Full) for the C_8H_{18} isomers. All computations are performed with the cc-pVDZ basis set and with Δ_{nb} = 10.0 Å. S9

S4 Total energies (in hartrees) from MP2, CCSD, CCSD(T), LSSMF(3,1)-MP2, LSSMF(3,1)-CCSD, and LSSMF(3,1)-CCSD(T) methods, percentages of the LSSMF energies, and the MAE and Δ_{max} values (in kcal mol^{-1}) of the LSSMF approaches with respect to the canonical methods (Full) for the C_9H_{20} isomers. All computations are performed with the cc-pVDZ basis set and with Δ_{nb} = 10.0 Å. S10

S5 Total energies (in hartrees) from MP2, CCSD, CCSD(T), LSSMF(3,1)-MP2, LSSMF(3,1)-CCSD, and LSSMF(3,1)-CCSD(T) methods, and percentages of the LSSMF energies with respect to the canonical methods (Full) for the first part of the $C_{10}H_{22}$ isomers. All computations are performed with the cc-pVDZ basis set and with Δ_{nb} = 10.0 Å. S11

S6 Total energies (in hartrees) from MP2, CCSD, CCSD(T), LSSMF(3,1)-MP2, LSSMF(3,1)-CCSD, and LSSMF(3,1)-CCSD(T) methods, percentages of the LSSMF energies, and the MAE and Δ_{max} values (in kcal mol^{-1}) of the LSSMF approaches with respect to the canonical methods (Full) for the second part of the $C_{10}H_{22}$ isomers. All computations are performed with the cc-pVDZ basis set and with $\Delta_{nb} = 10.0$ Å. S12

S7 Total energies (in hartrees) from FNO-CCSD, FNO-CCSD(T), LSSMF(3,1)-FNO-CCSD, and LSSMF(3,1)-FNO-CCSD(T) methods, percentages of the LSSMF energies, and the MAE and Δ_{max} values (in kcal mol^{-1}) of the LSSMF approaches with respect to the canonical methods (Full) for several $C_{10}H_{22}$ isomers. All computations are performed with the FNO occupation tolerance of 10^{-5} and $\Delta_{nb} = 10.0$ Å in a cc-pVTZ basis set. S13

S8 Total energies (in hartrees) from FNO-CCSD(T) and LSSMF(3,1)-FNO-CCSD(T) methods, percentages of the LSSMF energies, and the MAE and Δ_{max} values (in kcal mol^{-1}) of the LSSMF(3,1)-FNO-CCSD(T) approach with respect to CCSD(T) (Full) for several C_7H_{16} isomers. All computations are performed with the FNO occupation tolerance of 10^{-5} and $\Delta_{nb} = 10.0$ Å in the cc-pVXZ (X=D,T,Q) basis sets. S14

S9 The L12 set. The number of basis functions for the largest molecule is 1690 at the cc-pvDZ basis set. S15

S10 Total energies (in hartrees) from HF, MP2, LSSMF-HF, and LSSMF-MP2 methods, and percentages of the LSSMF energies, and the MAE and Δ_{max} values (in kcal mol^{-1}) of the LSSMF approaches with respect to the canonical methods (Full) for the L12 set. All computations are performed with the cc-pVDZ basis set, LSSMF bonded level 3, nonbonded level 1 and with $\Delta_{nb} = 7.5$ Å. S15

S11 Total energies (in hartrees) from HF, MP2, LSSMF-HF, and LSSMF-MP2 methods, and percentages of the LSSMF energies, and the MAE and Δ_{max} values (in kcal mol^{-1}) of the LSSMF approaches with respect to the canonical methods (Full) for the L12 set. All computations are performed with the cc-pVDZ basis set, LSSMF bonded level 3, nonbonded level 2 and with $\Delta_{nb} = 7.5$ Å. S16

S12 Total energies (in hartrees) from HF, MP2, LSSMF-HF, and LSSMF-MP2 methods, and percentages of the LSSMF energies, and the MAE and Δ_{max} values (in kcal mol^{-1}) of the LSSMF approaches with respect to the canonical methods (Full) for the L12 set. All computations are performed with the cc-pVDZ basis set, LSSMF bonded level 3, nonbonded level 1 and with $\Delta_{nb} = 10$ Å. S17

S13 Total energies (in hartrees) from HF, MP2, LSSMF-HF, and LSSMF-MP2 methods, and percentages of the LSSMF energies, and the MAE and Δ_{max} values (in kcal mol^{-1}) of the LSSMF approaches with respect to the canonical methods (Full) for the L12 set. All computations are performed with the cc-pVDZ basis set, LSSMF bonded level 3, nonbonded level 2 and with $\Delta_{nb} = 10$ Å. S18

S14 Total energies (in hartrees) from HF, MP2, LSSMF-HF, and LSSMF-MP2 methods, and percentages of the LSSMF energies, and the MAE and Δ_{max} values (in kcal mol^{-1}) of the LSSMF approaches with respect to the canonical methods (Full) for the L12 set. All computations are performed with the cc-pVDZ basis set, LSSMF bonded level 4, nonbonded level 1 and with $\Delta_{nb} = 7.5$ Å. S19

S15 Total energies (in hartrees) from HF, MP2, LSSMF-HF, and LSSMF-MP2 methods, and percentages of the LSSMF energies, and the MAE and Δ_{max} values (in kcal mol^{-1}) of the LSSMF approaches with respect to the canonical methods (Full) for the L12 set. All computations are performed with the cc-pVDZ basis set, LSSMF bonded level 4, nonbonded level 2 and with $\Delta_{nb} = 7.5$ Å. S20

S16 Total energies (in hartrees) from HF, MP2, LSSMF-HF, and LSSMF-MP2 methods, and percentages of the LSSMF energies, and the MAE and Δ_{max} values (in kcal mol^{-1}) of the LSSMF approaches with respect to the canonical methods (Full) for the L12 set. All computations are performed with the cc-pVDZ basis set, LSSMF bonded level 4, nonbonded level 1 and with $\Delta_{nb} = 10$ Å. S21
S17 Total energies (in hartrees) from HF, MP2, LSSMF-HF, and LSSMF-MP2 methods, and percentages of the LSSMF energies, and the MAE and Δ_{max} values (in kcal mol^{-1}) of the LSSMF approaches with respect to the canonical methods (Full) for the L12 set. All computations are performed with the cc-pVDZ basis set, LSSMF bonded level 4, nonbonded level 2 and with $\Delta_{nb} = 10$ Å. S22
S18 Total energies (in hartrees) from HF, MP2, LSSMF-HF, and LSSMF-MP2 methods, and percentages of the LSSMF energies, and the MAE and Δ_{max} values (in kcal mol^{-1}) of the LSSMF approaches with respect to the canonical methods (Full) for the L12 set. All computations are performed with the cc-pVDZ basis set, LSSMF bonded level 5, nonbonded level 1 and with $\Delta_{nb} = 7.5$ Å. S23
S19 Total energies (in hartrees) from HF, MP2, LSSMF-HF, and LSSMF-MP2 methods, and percentages of the LSSMF energies, and the MAE and Δ_{max} values (in kcal mol^{-1}) of the LSSMF approaches with respect to the canonical methods (Full) for the L12 set. All computations are performed with the cc-pVDZ basis set, LSSMF bonded level 5, nonbonded level 2 and with $\Delta_{nb} = 7.5$ Å. S24
S20 Total energies (in hartrees) from HF, MP2, LSSMF-HF, and LSSMF-MP2 methods, and percentages of the LSSMF energies, and the MAE and Δ_{max} values (in kcal mol^{-1}) of the LSSMF approaches with respect to the canonical methods (Full) for the L12 set. All computations are performed with the cc-pVDZ basis set, LSSMF bonded level 5, nonbonded level 1 and with $\Delta_{nb} = 10$ Å. S25

S21 Total energies (in hartrees) from HF, MP2, LSSMF-HF, and LSSMF-MP2 methods, and percentages of the LSSMF energies, and the MAE and Δ_{max} values (in kcal mol^{-1}) of the LSSMF approaches with respect to the canonical methods (Full) for the L12 set. All computations are performed with the cc-pVDZ basis set, LSSMF bonded level 5, nonbonded level 2 and with $\Delta_{nb} = 10$ Å. S26
S22 Total energies (in hartrees) from HF, MP2, LSSMF-HF, and LSSMF-MP2 methods, and percentages of the LSSMF energies, and the MAE and Δ_{max} values (in kcal mol^{-1}) of the LSSMF approaches with respect to the canonical methods (Full) for the L12 set. All computations are performed with the cc-pVDZ basis set, LSSMF bonded level 6, nonbonded level 1 and with $\Delta_{nb} = 7.5$ Å. S27
S23 Total energies (in hartrees) from HF, MP2, LSSMF-HF, and LSSMF-MP2 methods, and percentages of the LSSMF energies, and the MAE and Δ_{max} values (in kcal mol^{-1}) of the LSSMF approaches with respect to the canonical methods (Full) for the L12 set. All computations are performed with the cc-pVDZ basis set, LSSMF bonded level 6, nonbonded level 2 and with $\Delta_{nb} = 7.5$ Å. S28
S24 Total energies (in hartrees) from HF, MP2, LSSMF-HF, and LSSMF-MP2 methods, and percentages of the LSSMF energies, and the MAE and Δ_{max} values (in kcal mol^{-1}) of the LSSMF approaches with respect to the canonical methods (Full) for the L12 set. All computations are performed with the cc-pVDZ basis set, LSSMF bonded level 6, nonbonded level 1 and with $\Delta_{nb} = 10$ Å. S29
S25 Total energies (in hartrees) from HF, MP2, LSSMF-HF, and LSSMF-MP2 methods, and percentages of the LSSMF energies, and the MAE and Δ_{max} values (in kcal mol^{-1}) of the LSSMF approaches with respect to the canonical methods (Full) for the L12 set. All computations are performed with the cc-pVDZ basis set, LSSMF bonded level 6, nonbonded level 2 and with $\Delta_{nb} = 10$ Å. S30

Table S1: Total energies (in hartrees) from MP2, CCSD, CCSD(T), LSSMF(3,1)-MP2, LSSMF(3,1)-CCSD, and LSSMF(3,1)-CCSD(T) methods, percentages of the LSSMF energies, and the MAE and Δ_{max} values (in kcal mol^{-1}) of the LSSMF approaches with respect to the canonical methods (Full) for the C_6H_{14} isomers. All computations are performed with the cc-pVDZ basis set and with $\Delta_{nb} = 10.0$ Å.

	**MP2**			**CCSD**			**CCSD(T)**		
**Molecule**	**LSSMF**	**Full**	**%**	**LSSMF**	**Full**	**%**	**LSSMF**	**Full**	**%**
2,2-Dimethylbutane	−236.264 953	−236.265 415	99.9998	−236.351 852	−236.352 225	99.9998	−236.381 360	−236.381 741	99.9998
2,3-Dimethylbutane	−236.261 788	−236.262 227	99.9998	−236.349 351	−236.349 731	99.9998	−236.378 592	−236.378 984	99.9998
2-Methylpentane	−236.261 995	−236.261 792	100.0001	−236.350 475	−236.350 269	100.0001	−236.379 271	−236.379 051	100.0001
3-Methylpentane	−236.260 288	−236.260 434	99.9999	−236.348 608	−236.348 726	100.0000	−236.377 529	−236.377 640	100.0000
n-Hexane	−236.260 439	−236.259 978	100.0002	−236.349 841	−236.349 403	100.0002	−236.378 190	−236.377 738	100.0002
**MAE**			**0.21**			**0.19**			**0.20**
Δ_{max}			**0.29**			**0.28**			**0.28**

Table S2: Total energies (in hartrees) from MP2, CCSD, CCSD(T), LSSMF(3,1)-MP2, LSSMF(3,1)-CCSD, and LSSMF(3,1)-CCSD(T) methods, percentages of the LSSMF energies, and the MAE and Δ_{max} values (in kcal mol^{-1}) of the LSSMF approaches with respect to the canonical methods (Full) for the C_7H_{16} isomers. All computations are performed with the cc-pVDZ basis set and with Δ_{nb} = 10.0 Å.

	MP2			CCSD			CCSD(T)		
**Molecule**	**LSSMF**	**Full**	%	**LSSMF**	**Full**	%	**LSSMF**	**Full**	%
2,2,3-Trimethylbutane	−275.444 589	−275.445 313	99.9997	−275.543 263	−275.543 831	99.9998	−275.578 464	−275.579 042	99.9998
2,2-Dimethylpentane	−275.446 420	−275.446 213	100.0001	−275.546 177	−275.545 903	100.0001	−275.580 806	−275.580 504	100.0001
2,3-Dimethylpentane	−275.441 661	−275.441 954	99.9999	−275.541 947	−275.542 175	99.9999	−275.576 400	−275.576 623	99.9999
2,4-Dimethylpentane	−275.441 129	−275.441 220	100.0000	−275.541 701	−275.541 734	100.0000	−275.576 112	−275.576 053	100.0000
2-Methylhexane	−275.443 011	−275.442 547	100.0002	−275.544 434	−275.543 974	100.0002	−275.578 254	−275.577 776	100.0002
3,3-Dimethylpentane	−275.442 975	−275.443 744	99.9997	−275.542 447	−275.543 071	99.9998	−275.577 267	−275.577 902	99.9998
3-Ethylpentane	−275.439 219	−275.440 095	99.9997	−275.540 102	−275.540 880	99.9997	−275.574 370	−275.575 024	99.9998
3-Methlyhexane	−275.440 548	−275.440 657	100.0000	−275.541 584	−275.541 638	100.0000	−275.575 716	−275.575 698	100.0000
n-Heptane	−275.440 451	−275.439 724	100.0003	−275.542 681	−275.541 986	100.0003	−275.576 132	−275.575 416	100.0003
**MAE**			**0.30**			**0.26**			**0.26**
Δ_{max}			**0.55**			**0.49**			**0.45**

Table S3: Total energies (in hartrees) from MP2, CCSD, CCSD(T), LSSMF(3,1)-MP2, LSSMF(3,1)-CCSD, and LSSMF(3,1)-CCSD(T) methods, percentages of the LSSMF energies, and the MAE and Δ_{max} values (in kcal mol^{-1}) of the LSSMF approaches with respect to the canonical methods (Full) for the C_8H_{18} isomers. All computations are performed with the cc-pVDZ basis set and with $\Delta_{nb} = 10.0$ Å.

	**MP2**			**CCSD**			**CCSD(T)**		
**Molecule**	**LSSMF**	**Full**	**%**	**LSSMF**	**Full**	**%**	**LSSMF**	**Full**	**%**
2,2-Dimethylhexane	−314.627 508	−314.627 027	100.0002	−314.740 199	−314.739 647	100.0002	−314.779 858	−314.779 278	100.0002
2,3,3-Trimethylpentane	−314.622 389	−314.623 094	99.9998	−314.733 558	−314.734 071	99.9998	−314.774 161	−314.774 666	99.9998
2,3,4-Trimethylpentane	−314.620 266	−314.620 926	99.9998	−314.732 454	−314.732 949	99.9998	−314.772 676	−314.773 055	99.9999
2,3-Dimethylhexane	−314.623 052	−314.622 738	100.0001	−314.736 208	−314.735 840	100.0001	−314.775 776	−314.775 373	100.0001
2,4-Dimethylhexane	−314.624 433	−314.624 222	100.0001	−314.737 552	−314.737 266	100.0001	−314.777 148	−314.776 762	100.0001
2,5-Dimethylhexane	−314.625 821	−314.625 351	100.0001	−314.739 234	−314.738 740	100.0002	−314.778 538	−314.778 026	100.0002
2-Methylheptane	−314.623 941	−314.623 224	100.0002	−314.738 321	−314.737 619	100.0002	−314.777 157	−314.776 433	100.0002
3,3-Dimethylhexane	−314.624 859	−314.624 719	100.0000	−314.737 159	−314.736 915	100.0001	−314.777 122	−314.776 844	100.0001
3,4-Dimethylhexane	−314.621 659	−314.621 649	100.0000	−314.734 551	−314.734 453	100.0000	−314.774 351	−314.774 162	100.0001
3-Ethyl-2-methylpentane	−314.619 157	−314.619 099	100.0000	−314.732 111	−314.731 995	100.0000	−314.771 980	−314.771 745	100.0001
3-Ethyl-3-methylpentane	−314.618 287	−314.619 153	99.9997	−314.730 297	−314.730 997	99.9998	−314.770 644	−314.771 246	99.9998
3-Ethylhexane	−314.618 384	−314.618 240	100.0000	−314.732 351	−314.732 139	100.0001	−314.771 709	−314.771 379	100.0001
3-Methylheptane	−314.622 577	−314.621 993	100.0002	−314.736 736	−314.736 143	100.0002	−314.775 759	−314.775 138	100.0002
4-Methylheptane	−314.622 774	−314.621 926	100.0003	−314.736 902	−314.736 058	100.0003	−314.775 978	−314.775 082	100.0003
2,2,3,3-Tetramethylbutane	−314.625 823	−314.626 612	99.9997	−314.735 445	−314.735 942	99.9998	−314.776 764	−314.777 251	99.9998
2,2,3-Trimethylpentane	−314.624 454	−314.624 747	99.9999	−314.735 762	−314.735 885	100.0000	−314.776 274	−314.776 361	100.0000
2,2,4-Trimethylpentane	−314.624 738	−314.625 034	99.9999	−314.736 475	−314.736 596	100.0000	−314.776 863	−314.776 824	100.0000
n-Octane	−314.622 296	−314.621 318	100.0003	−314.737 610	−314.736 682	100.0003	−314.775 990	−314.775 036	100.0003
**MAE**			**0.30**			**0.27**			**0.29**
Δ_{max}			**0.61**			**0.58**			**0.60**

Table S4: Total energies (in hartrees) from MP2, CCSD, CCSD(T), LSSMF(3,1)-MP2, LSSMF(3,1)-CCSD, and LSSMF(3,1)-CCSD(T) methods, percentages of the LSSMF energies, and the MAE and Δ_{max} values (in kcal mol^{-1}) of the LSSMF approaches with respect to the canonical methods (Full) for the C_9H_{20} isomers. All computations are performed with the cc-pVDZ basis set and with $\Delta_{nb} = 10.0$ Å.

	MP2			CCSD			CCSD(T)		
**Molecule**	**LSSMF**	**Full**	%	**LSSMF**	**Full**	%	**LSSMF**	**Full**	%
2-2-3-3-Tetramethylpentane	−353.803 876	−353.804 353	99.9999	−353.925 902	−353.926 049	100.0000	−353.972 705	−353.972 799	100.0000
2-2-3-4-Tetramethylpentane	−353.795 120	−353.796 718	99.9995	−353.917 801	−353.919 035	99.9997	−353.964 013	−353.965 043	99.9997
2-2-3-Trimethylhexane	−353.805 919	−353.805 678	100.0001	−353.930 100	−353.929 688	100.0001	−353.975 707	−353.975 237	100.0001
2-2-4-4-Tetramethylpentane	−353.805 203	−353.805 592	99.9999	−353.928 176	−353.928 243	100.0000	−353.974 664	−353.974 462	100.0001
2-2-4-Trimethylhexane	−353.806 524	−353.806 331	100.0001	−353.930 895	−353.930 475	100.0001	−353.976 536	−353.975 929	100.0002
2-2-5-Trimethylhexane	−353.810 138	−353.809 599	100.0002	−353.934 806	−353.934 165	100.0002	−353.979 970	−353.979 297	100.0002
3-Ethyl-2,2-dimethylpentane	−353.801 818	−353.802 219	99.9999	−353.925 699	−353.925 863	100.0000	−353.971 733	−353.971 704	100.0000
2,2-Dimethylheptane	−353.808 467	−353.807 738	100.0002	−353.934 109	−353.933 319	100.0002	−353.978 788	−353.977 967	100.0002
2,3,3,4-tetramethylpentane	−353.801 433	−353.803 059	99.9995	−353.924 185	−353.925 498	99.9996	−353.970 716	−353.971 919	99.9997
2,3,3-Trimethylhexane	−353.800 494	−353.801 376	99.9998	−353.924 500	−353.925 126	99.9998	−353.970 412	−353.970 890	99.9999
2,3,4-Trimethylhexane	−353.802 276	−353.801 852	100.0001	−353.926 968	−353.926 403	100.0002	−353.972 449	−353.971 770	100.0002
2,3,5-Trimethylhexane	−353.802 344	−353.802 426	100.0000	−353.927 446	−353.927 382	100.0000	−353.972 792	−353.972 535	100.0001
2,3-Dimethylheptane	−353.803 981	−353.803 524	100.0001	−353.930 101	−353.929 597	100.0001	−353.974 659	−353.974 129	100.0001
2,4,4-Trimethylhexane	−353.802 906	−353.803 402	99.9999	−353.927 143	−353.927 400	99.9999	−353.972 908	−353.972 997	100.0000
3-Ethyl-2,4-dimethylpentane	−353.797 904	−353.799 085	99.9997	−353.922 661	−353.923 613	99.9997	−353.968 437	−353.969 096	99.9998
2,4-Dimethylheptane	−353.802 365	−353.801 942	100.0001	−353.928 546	−353.928 063	100.0001	−353.973 222	−353.972 621	100.0002
2,5-Dimethylheptane	−353.804 922	−353.804 454	100.0001	−353.931 111	−353.930 601	100.0001	−353.975 596	−353.975 061	100.0002
2-6-Dimethylheptane	−353.806 589	−353.805 852	100.0002	−353.932 982	−353.932 234	100.0002	−353.977 297	−353.976 525	100.0002
4-Ethyl-2-methylhexane	−353.796 794	−353.797 408	99.9998	−353.922 754	−353.923 208	99.9999	−353.967 871	−353.968 081	99.9999
2-Methyloctane	−353.804 895	−353.803 912	100.0003	−353.932 226	−353.931 272	100.0003	−353.976 081	−353.975 099	100.0003
3,3,4-Trimethylhexane	−353.798 077	−353.799 392	99.9996	−353.921 855	−353.922 914	99.9997	−353.967 951	−353.968 871	99.9997
3,3-Dimethylheptane	−353.805 954	−353.805 528	100.0001	−353.931 181	−353.930 647	100.0002	−353.976 181	−353.975 612	100.0002
3,4-Dimethylheptane	−353.802 028	−353.801 309	100.0002	−353.927 784	−353.926 991	100.0002	−353.972 671	−353.971 789	100.0002
3,5-Dimethylheptane	−353.800 624	−353.800 776	100.0000	−353.926 654	−353.926 701	100.0000	−353.971 433	−353.971 383	100.0000
3-Ethyl-2,3-dimethylpentane	−353.801 283	−353.802 346	99.9997	−353.924 837	−353.925 677	99.9998	−353.970 977	−353.971 676	99.9998
3-Ethyl-2-methylhexane	−353.799 031	−353.799 259	99.9999	−353.924 837	−353.924 922	100.0000	−353.969 948	−353.969 799	100.0000
3-Ethyl-3-methylhexane	−353.799 837	−353.800 293	99.9999	−353.924 633	−353.924 867	99.9999	−353.970 148	−353.970 243	100.0000
3-Ethyl-4-methylhexane	−353.797 965	−353.798 502	99.9998	−353.923 535	−353.923 883	99.9999	−353.968 808	−353.968 936	100.0000
3-Ethylheptane	−353.800 015	−353.799 651	100.0001	−353.926 890	−353.926 437	100.0001	−353.971 283	−353.970 696	100.0002
3-Methyloctane	−353.803 454	−353.802 778	100.0002	−353.930 596	−353.929 916	100.0002	−353.974 601	−353.973 897	100.0002
4,4-Dimethylheptane	−353.806 431	−353.805 562	100.0002	−353.931 576	−353.930 619	100.0003	−353.976 677	−353.975 648	100.0003
4-Ethylheptane	−353.799 971	−353.799 319	100.0002	−353.926 825	−353.926 132	100.0002	−353.971 265	−353.970 419	100.0002
4-Methyloctane	−353.803 735	−353.802 815	100.0003	−353.930 819	−353.929 907	100.0003	−353.974 883	−353.973 928	100.0003
3,3-Diethylpentane	−353.790 542	−353.792 949	99.9993	−353.914 995	−353.917 105	99.9994	−353.961 094	−353.962 908	99.9995
n-Nonane	−353.803 227	−353.801 986	100.0004	−353.931 497	−353.930 319	100.0003	−353.974 893	−353.973 683	100.0003
**MAE**			**0.45**			**0.40**			**0.39**
Δ_{max}			**1.51**			**1.32**			**1.14**

Table S5: Total energies (in hartrees) from MP2, CCSD, CCSD(T), LSSMF(3,1)-MP2, LSSMF(3,1)-CCSD, and LSSMF(3,1)-CCSD(T) methods, and percentages of the LSSMF energies with respect to the canonical methods (Full) for the first part of the $C_{10}H_{22}$ isomers. All computations are performed with the cc-pVDZ basis set and with $\Delta_{nb} = 10.0$ Å.

	MP2			CCSD			CCSD(T)		
**Molecule**	**LSSMF**	**Full**	**%**	**LSSMF**	**Full**	**%**	**LSSMF**	**Full**	**%**
2,2,3,3,4-Pentamethylpentane	−392.975 407	−392.978 166	99.9993	−393.108 961	−393.111 154	99.9994	−393.162 015	−393.163 949	99.9995
2,2,3,3-Tetramethylhexane	−392.985 624	−392.985 347	100.0001	−393.120 453	−393.119 848	100.0002	−393.172 418	−393.171 724	100.0002
2,2,3,4,4-Pentamethylpentane	−392.976 794	−392.978 597	99.9995	−393.110 850	−393.112 104	99.9997	−393.163 693	−393.164 635	99.9998
2,2,3,4-Tetramethylhexane	−392.981 045	−392.982 899	99.9995	−393.116 714	−393.118 180	99.9996	−393.168 495	−393.169 695	99.9997
2,2,3,5-Tetramethylhexane	−392.989 159	−392.988 561	100.0002	−393.125 210	−393.124 371	100.0002	−393.176 440	−393.175 485	100.0002
3-Ethyl-2,2,3-trimethylpentane	−392.977 767	−392.979 840	99.9995	−393.112 138	−393.113 725	99.9996	−393.164 727	−393.166 016	99.9997
2,2,3-Trimethylheptane	−392.986 990	−392.986 462	100.0001	−393.124 109	−393.123 413	100.0002	−393.174 744	−393.173 990	100.0002
2,2,4,4-Tetramethylhexane	−392.983 648	−392.984 257	99.9998	−393.119 111	−393.119 333	99.9999	−393.170 975	−393.170 923	100.0000
2,2,4,5-Tetramethylhexane	−392.988 236	−392.988 122	100.0000	−393.124 450	−393.124 035	100.0001	−393.175 723	−393.175 111	100.0002
3-Ethyl-2,2,4-trimethylpentane	−392.976 686	−392.978 190	99.9996	−393.112 315	−393.113 471	99.9997	−393.164 399	−393.165 163	99.9998
2,2,4-Trimethylheptane	−392.985 250	−392.985 188	100.0000	−393.122 609	−393.122 355	100.0001	−393.173 288	−393.172 855	100.0001
2,2,5,5-Tetramethylhexane	−392.994 671	−392.994 053	100.0002	−393.130 558	−393.129 750	100.0002	−393.181 599	−393.180 749	100.0002
2,2,5-Trimethylheptane	−392.989 364	−392.988 995	100.0001	−393.126 618	−393.126 090	100.0001	−393.177 111	−393.176 475	100.0002
2,2,6-Trimethylheptane	−392.990 395	−392.989 315	100.0003	−393.127 972	−393.126 803	100.0003	−393.178 197	−393.176 984	100.0003
3-Ethyl-2,2-dimethylhexane	−392.980 662	−392.981 409	99.9998	−393.117 142	−393.117 587	99.9999	−393.168 548	−393.168 659	100.0000
4-Ethyl-2,2-dimethylhexane	−392.983 258	−392.983 570	99.9999	−393.120 180	−393.120 272	100.0000	−393.171 231	−393.171 037	100.0000
2,2-Dimethyloctane	−392.989 417	−392.988 420	100.0003	−393.128 013	−393.126 968	100.0003	−393.177 710	−393.176 629	100.0003
2,3,3,4-Tetramethylhexane	−392.982 841	−392.982 632	100.0001	−393.118 175	−393.117 652	100.0001	−393.170 010	−393.169 338	100.0002
2,3,3,5-Tetramethylhexane	−392.983 546	−392.984 438	99.9998	−393.119 411	−393.119 949	99.9999	−393.171 061	−393.171 310	99.9999
2,3,3-Trimethylheptane	−392.985 887	−392.985 356	100.0001	−393.122 826	−393.122 128	100.0002	−393.173 590	−393.172 843	100.0002
2,3,4,4-Tetramethylhexane	−392.981 986	−392.982 789	99.9998	−393.117 528	−393.117 953	99.9999	−393.169 375	−393.169 550	100.0000
2,3,4,5-Tetramethylhexane	−392.983 375	−392.984 133	99.9998	−393.119 732	−393.120 235	99.9999	−393.171 079	−393.171 331	99.9999
3-Ethyl-2,3,4-trimethylpentane	−392.975 497	−392.979 581	99.9990	−393.110 569	−393.114 182	99.9991	−393.162 940	−393.166 140	99.9992
2,3,4-Trimethylheptane	−392.983 023	−392.983 419	99.9999	−393.120 411	−393.120 640	99.9999	−393.171 142	−393.171 144	100.0000
2,3,5-Trimethylheptane	−392.985 934	−392.985 986	100.0000	−393.123 543	−393.123 396	100.0000	−393.174 090	−393.173 745	100.0001
2,3,6-Trimethylheptane	−392.985 877	−392.985 162	100.0002	−393.123 805	−393.122 999	100.0002	−393.174 041	−393.173 129	100.0002
3-Ethyl-2,3-dimethylhexane	−392.982 440	−392.984 099	99.9996	−393.118 713	−393.120 045	99.9997	−393.170 142	−393.171 202	99.9997
4-Ethyl-2,3-dimethylhexane	−392.983 378	−392.982 192	100.0003	−393.120 583	−393.119 246	100.0003	−393.171 477	−393.169 939	100.0004
2,3-Dimethyloctane	−392.984 966	−392.984 394	100.0001	−393.123 936	−393.123 299	100.0002	−393.173 623	−393.172 891	100.0002
2,4,4-Trimethylheptane	−392.984 684	−392.984 058	100.0002	−393.121 755	−393.120 945	100.0002	−393.172 674	−393.171 634	100.0003
2,4,5-Trimethylheptane	−392.982 521	−392.981 990	100.0001	−393.120 113	−393.119 352	100.0002	−393.170 883	−393.169 875	100.0003
2,4,6-Trimethylheptane	−392.983 380	−392.983 122	100.0001	−393.121 343	−393.120 909	100.0001	−393.171 816	−393.171 131	100.0002
3-Ethyl-2,4-dimethylhexane	−392.980 984	−392.980 342	100.0002	−393.118 347	−393.117 538	100.0002	−393.169 305	−393.168 239	100.0003
3-Isopropyl-2,4-dimethylpentane	−392.973 458	−392.975 329	99.9995	−393.109 856	−393.111 382	99.9996	−393.161 716	−393.162 750	99.9997
4-Ethyl-2,4-dimethylhexane	−392.982 273	−392.982 833	99.9999	−393.118 913	−393.119 188	99.9999	−393.170 179	−393.170 169	100.0000
2,4-Dimethyloctane	−392.981 311	−392.980 200	100.0003	−393.120 290	−393.119 127	100.0003	−393.170 156	−393.168 816	100.0003
2,5,5-Trimethylheptane	−392.988 083	−392.987 993	100.0000	−393.125 121	−393.124 841	100.0001	−393.175 800	−393.175 390	100.0001
3-Ethyl-2,5-dimethylhexane	−392.983 064	−392.982 132	100.0002	−393.120 579	−393.119 449	100.0003	−393.171 392	−393.169 976	100.0004

Table S6: Total energies (in hartrees) from MP2, CCSD, CCSD(T), LSSMF(3,1)-MP2, LSSMF(3,1)-CCSD, and LSSMF(3,1)-CCSD(T) methods, percentages of the LSSMF energies, and the MAE and Δ_{max} values (in kcal mol^{-1}) of the LSSMF approaches with respect to the canonical methods (Full) for the second part of the $C_{10}H_{22}$ isomers. All computations are performed with the cc-pVDZ basis set and with $\Delta_{nb} = 10.0$ Å.

	MP2			CCSD			CCSD(T)		
**Molecule**	**LSSMF**	**Full**	**%**	**LSSMF**	**Full**	**%**	**LSSMF**	**Full**	**%**
2,5-Dimethyloctane	−392.985 481	−392.984 147	100.0003	−393.124 295	−393.122 927	100.0003	−393.174 096	−393.172 578	100.0004
2,6-Dimethyloctane	−392.985 830	−392.985 230	100.0002	−393.124 795	−393.124 124	100.0002	−393.174 448	−393.173 673	100.0002
2,7-Dimethyloctane	−392.986 661	−392.985 373	100.0003	−393.125 948	−393.124 683	100.0003	−393.175 329	−393.174 022	100.0003
3,3-Diethyl-2-methylpentane	−392.974 068	−392.978 043	99.9990	−393.109 924	−393.113 443	99.9991	−393.161 920	−393.165 018	99.9992
3-Ethyl-2-methylheptane	−392.984 281	−392.982 955	100.0003	−393.122 959	−393.121 577	100.0004	−393.172 932	−393.171 420	100.0004
4-Ethyl-2-methylheptane	−392.985 395	−392.985 217	100.0000	−393.123 849	−393.123 521	100.0001	−393.174 001	−393.173 387	100.0002
5-Ethyl-2-methylheptane	−392.983 583	−392.983 212	100.0001	−393.122 196	−393.121 728	100.0001	−393.172 156	−393.171 519	100.0002
2-Methylnonane	−392.985 000	−392.983 446	100.0004	−393.125 224	−393.123 729	100.0004	−393.174 148	−393.172 607	100.0004
3,3,4,4-Tetramethylhexane	−392.981 959	−392.982 037	100.0000	−393.116 379	−393.116 082	100.0001	−393.168 683	−393.168 281	100.0001
3,3,4-Trimethylheptane	−392.984 614	−392.984 606	100.0000	−393.121 212	−393.120 952	100.0001	−393.172 318	−393.171 907	100.0001
3,3,5-Trimethylheptane	−392.982 247	−392.981 924	100.0001	−393.119 147	−393.118 627	100.0001	−393.170 149	−393.169 436	100.0002
3,3-Diethylhexane	−392.981 661	−392.983 914	99.9994	−393.118 702	−393.120 590	99.9995	−393.169 757	−393.171 311	99.9996
4-Ethyl-3,3-dimethylhexane	−392.977 692	−392.979 764	99.9995	−393.113 910	−393.115 632	99.9996	−393.165 590	−393.166 941	99.9997
3,3-Dimethyloctane	−392.987 142	−392.987 244	100.0000	−393.125 245	−393.125 187	100.0000	−393.175 351	−393.175 183	100.0000
3,4,4-Trimethylheptane	−392.984 800	−392.984 675	100.0000	−393.121 348	−393.120 979	100.0001	−393.172 524	−393.171 968	100.0001
3,4,5-Trimethylheptane	−392.981 695	−392.983 067	99.9997	−393.118 731	−393.119 842	99.9997	−393.169 799	−393.170 594	99.9998
3,4-Diethylhexane	−392.982 500	−392.980 856	100.0004	−393.120 588	−393.118 851	100.0004	−393.171 093	−393.169 119	100.0005
3-Ethyl-3,4-dimethylhexane	−392.981 546	−392.981 667	100.0000	−393.117 709	−393.117 587	100.0000	−393.169 207	−393.168 886	100.0001
3,4-Dimethyloctane	−392.984 151	−392.983 224	100.0002	−393.122 937	−393.121 959	100.0002	−393.172 788	−393.171 743	100.0003
3,5-Dimethyloctane	−392.985 458	−392.984 889	100.0001	−393.124 151	−393.123 489	100.0002	−393.174 052	−393.173 268	100.0002
3,6-Dimethyloctane	−392.983 992	−392.983 672	100.0001	−393.122 794	−393.122 386	100.0001	−393.172 581	−393.172 076	100.0001
3-Ethyl-4-methylheptane	−392.983 434	−392.981 943	100.0004	−393.121 790	−393.120 213	100.0004	−393.172 058	−393.170 289	100.0004
3-Ethyloctane	−392.982 674	−392.982 420	100.0001	−393.122 323	−393.122 002	100.0001	−393.171 737	−393.171 257	100.0001
3-Isopropyl-2-methylhexane	−392.982 181	−392.981 482	100.0002	−393.119 762	−393.118 902	100.0002	−393.170 522	−393.169 419	100.0003
3-Ethyl-3-methylheptane	−392.984 641	−392.984 694	100.0000	−393.122 283	−393.122 156	100.0000	−393.172 784	−393.172 513	100.0001
3-Ethyl-5-methylheptane	−392.983 438	−392.983 746	99.9999	−393.121 765	−393.121 897	100.0000	−393.172 010	−393.171 874	100.0000
3-Methylnonane	−392.984 387	−392.983 445	100.0002	−393.124 484	−393.123 552	100.0002	−393.173 506	−393.172 544	100.0002
4,4-Dimethyloctane	−392.987 534	−392.986 382	100.0003	−393.125 611	−393.124 368	100.0003	−393.175 745	−393.174 430	100.0003
4,5-Dimethyloctane	−392.984 598	−392.983 511	100.0003	−393.123 229	−393.122 060	100.0003	−393.173 234	−393.171 925	100.0003
4-Ethyl-3-methylheptane	−392.983 455	−392.981 960	100.0004	−393.121 867	−393.120 303	100.0004	−393.172 086	−393.170 351	100.0004
4-Ethyl-4-methylheptane	−392.985 141	−392.985 650	99.9999	−393.122 624	−393.122 884	99.9999	−393.173 303	−393.173 314	100.0000
4-Ethyloctane	−392.983 137	−392.982 544	100.0002	−393.122 714	−393.122 062	100.0002	−393.172 206	−393.171 369	100.0002
4-Isopropylheptane	−392.983 863	−392.982 242	100.0004	−393.122 297	−393.120 588	100.0004	−393.172 486	−393.170 589	100.0005
4-Methylnonane	−392.984 377	−392.982 843	100.0004	−393.124 333	−393.122 836	100.0004	−393.173 482	−393.171 925	100.0004
4-n-Propylheptane	−392.983 885	−392.982 801	100.0003	−393.123 351	−393.122 216	100.0003	−393.172 962	−393.171 596	100.0003
5-Methylnonane	−392.984 703	−392.983 524	100.0003	−393.124 523	−393.123 319	100.0003	−393.173 799	−393.172 463	100.0003
n-Decane	−392.984 158	−392.982 654	100.0004	−393.125 384	−393.123 955	100.0004	−393.173 796	−393.172 329	100.0004
**MAE**			**0.59**			**0.58**			**0.59**
Δ_{max}			**2.56**			**2.27**			**2.01**

Table S7: Total energies (in hartrees) from FNO-CCSD, FNO-CCSD(T), LSSMF(3,1)-FNO-CCSD, and LSSMF(3,1)-FNO-CCSD(T) methods, percentages of the LSSMF energies, and the MAE and Δ_{max} values (in kcal mol^{-1}) of the LSSMF approaches with respect to the canonical methods (Full) for several $C_{10}H_{22}$ isomers. All computations are performed with the FNO occupation tolerance of 10^{-5} and $\Delta_{nb} = 10.0$ Å in a cc-pVTZ basis set.

	**CCSD**			**CCSD(T)**		
**Molecule**	**LSSMF**	**Full**	**%**	**LSSMF**	**Full**	**%**
2,2,5,5-Tetramethylhexane	−393.524 341	−393.523 059	100.0003	−393.602 096	−393.600 669	100.0004
2,2,6-Trimethylheptane	−393.521 472	−393.520 003	100.0004	−393.598 417	−393.596 753	100.0004
2,2-Dimethyloctane	−393.521 093	−393.520 195	100.0002	−393.597 419	−393.596 365	100.0003
3,3-Diethylhexane	−393.513 212	−393.514 480	99.9997	−393.591 338	−393.592 026	99.9998
n-Decane	−393.518 108	−393.517 132	100.0002	−393.593 034	−393.591 934	100.0003
**MAE**			**0.74**			**0.74**
Δ_{max}			**0.92**			**1.04**

Table S8: Total energies (in hartrees) from FNO-CCSD(T) and LSSMF(3,1)-FNO-CCSD(T) methods, percentages of the LSSMF energies, and the MAE and Δ_{max} values (in kcal mol^{-1}) of the LSSMF(3,1)-FNO-CCSD(T) approach with respect to CCSD(T) (Full) for several C_7H_{16} isomers. All computations are performed with the FNO occupation tolerance of 10^{-5} and $\Delta_{nb} = 10.0$ Å in the cc-pVXZ (X=D,T,Q) basis sets.

	**cc-pVDZ**			**cc-pVTZ**			**cc-pVQZ**		
**Molecule**	**LSSMF**	**Full**	**%**	**LSSMF**	**Full**	**%**	**LSSMF**	**Full**	**%**
2,2,3-Trimethylbutane	−275.578 464	−275.579 042	99.9998	−275.875 353	−275.876 084	99.9997	−275.962 537	−275.962 990	99.9998
2,2-Dimethylhexane	−275.580 806	−275.580 504	100.0001	−275.877 430	−275.877 015	100.0002	−275.964 706	−275.963 973	100.0003
n-Heptane	−275.576 132	−275.575 416	100.0003	−275.872 493	−275.871 798	100.0003	−275.959 800	−275.958 833	100.0004
**MAE**			**0.33**			**0.38**			**0.45**
Δ_{max}			**0.45**			**0.46**			**0.61**

Table S9: The L12 set. The number of basis functions for the largest molecule is 1690 at the cc-pvDZ basis set.

Set Entry	Name	Formula
1	Pentacontane	$C_{50}H_{102}$
2	14-Butyl-13-decyl-13-octyloctacosane	$C_{50}H_{102}$
3	9-Tert-butyl-11-heptyl-10-hexyl-11-octyl-10-pentylicosane	$C_{50}H_{102}$
4	8,14-Diethyl-9-heptyl-13-hexyl-10,15-dimethyl-11-pentyl-12-propyltricosane	$C_{50}H_{102}$
5	Hexacontane	$C_{60}H_{122}$
6	13-Methyl-11,13,15,17-tetraoctylheptacosane	$C_{60}H_{122}$
7	12-(2-Ethylhexyl)-11-heptyl-10-hexyl-12-nonyl-11-octyldocosane	$C_{60}H_{122}$
8	11-Decyl-10-heptyl-12,12-dimethyl-11-nonyl-10-octyltetracosane	$C_{60}H_{122}$
9	Heptacontane	$C_{70}H_{142}$
10	16-Decyl-16-pentadecyl-15-tridecyldotriacontane	$C_{70}H_{142}$
11	17,17,18-Tridodecyltetratriacontane	$C_{70}H_{142}$
12	15-(2,4,4,6,6-Pentamethylheptan-2-yl)-16-tetradecyl-16-tridecylhentriacontane	$C_{70}H_{142}$

Table S10: Total energies (in hartrees) from HF, MP2, LSSMF-HF, and LSSMF-MP2 methods, and percentages of the LSSMF energies, and the MAE and Δ_{max} values (in kcal mol^{-1}) of the LSSMF approaches with respect to the canonical methods (Full) for the L12 set. All computations are performed with the cc-pVDZ basis set, LSSMF bonded level 3, nonbonded level 1 and with $\Delta_{nb} = 7.5$ Å.

	HF			MP2		
**Molecule**	**LSSMF**	**Full**	**%**	**LSSMF**	**Full**	**%**
1	-1952.991 219	-1952.987 297	100.0002	-1960.203 890	-1960.190 335	100.0007
2	-1952.942 575	-1952.949 313	99.9997	-1960.200 288	-1960.187 563	100.0006
3	-1952.789 480	-1952.823 248	99.9983	-1960.126 133	-1960.121 466	100.0002
4	-1952.821 599	-1952.853 728	99.9984	-1960.152 655	-1960.146 331	100.0003
5	-2343.357 434	-2343.352 655	100.0002	-2352.009 703	-2351.993 178	100.0007
6	-2343.292 988	-2343.303 250	99.9996	-2352.020 786	-2352.002 237	100.0008
7	-2343.178 516	-2343.207 638	99.9988	-2351.960 174	-2351.946 323	100.0006
8	-2343.185 223	-2343.213 895	99.9988	-2351.946 006	-2351.939 182	100.0003
9	-2733.723 654	-2733.718 018	100.0002	-2743.815 521	-2743.796 025	100.0007
10	-2733.674 218	-2733.679 919	99.9998	-2743.812 259	-2743.793 968	100.0007
11	-2733.674 159	-2733.679 882	99.9998	-2743.812 240	-2743.793 960	100.0007
12	-2733.569 350	-2733.593 251	99.9991	-2743.768 480	-2743.758 118	100.0004
**MAE**			**9.95**			**8.34**
Δ_{max}			**21.19**			**12.23**

Table S11: Total energies (in hartrees) from HF, MP2, LSSMF-HF, and LSSMF-MP2 methods, and percentages of the LSSMF energies, and the MAE and Δ_{max} values (in kcal mol^{-1}) of the LSSMF approaches with respect to the canonical methods (Full) for the L12 set. All computations are performed with the cc-pVDZ basis set, LSSMF bonded level 3, nonbonded level 2 and with $\Delta_{nb} = 7.5$ Å.

	**HF**			**MP2**		
**Molecule**	**LSSMF**	**Full**	**%**	**LSSMF**	**Full**	**%**
1	-1952.991 138	-1952.987 297	100.0002	-1960.203 602	-1960.190 335	100.0007
2	-1952.943 093	-1952.949 313	99.9997	-1960.196 564	-1960.187 563	100.0005
3	-1952.800 015	-1952.823 248	99.9988	-1960.121 929	-1960.121 466	100.0000
4	-1952.832 548	-1952.853 728	99.9989	-1960.147 888	-1960.146 331	100.0001
5	-2343.357 337	-2343.352 655	100.0002	-2352.009 351	-2351.993 178	100.0007
6	-2343.297 501	-2343.303 250	99.9998	-2352.014 326	-2352.002 237	100.0005
7	-2343.191 033	-2343.207 638	99.9993	-2351.953 101	-2351.946 323	100.0003
8	-2343.191 603	-2343.213 895	99.9990	-2351.941 460	-2351.939 182	100.0001
9	-2733.723 541	-2733.718 018	100.0002	-2743.815 105	-2743.796 025	100.0007
10	-2733.674 995	-2733.679 919	99.9998	-2743.808 298	-2743.793 968	100.0005
11	-2733.674 948	-2733.679 882	99.9998	-2743.808 279	-2743.793 960	100.0005
12	-2733.599 415	-2733.593 251	100.0002	-2743.775 740	-2743.758 118	100.0006
**MAE**			**6.55**			**6.64**
Δ_{max}			**14.58**			**11.97**

Table S12: Total energies (in hartrees) from HF, MP2, LSSMF-HF, and LSSMF-MP2 methods, and percentages of the LSSMF energies, and the MAE and Δ_{max} values (in kcal mol^{-1}) of the LSSMF approaches with respect to the canonical methods (Full) for the L12 set. All computations are performed with the cc-pVDZ basis set, LSSMF bonded level 3, nonbonded level 1 and with $\Delta_{nb} = 10$ Å.

	HF			MP2		
**Molecule**	**LSSMF**	**Full**	**%**	**LSSMF**	**Full**	**%**
1	-1952.991 235	-1952.987 297	100.0002	-1960.203 982	-1960.190 335	100.0007
2	-1952.942 601	-1952.949 313	99.9997	-1960.200 441	-1960.187 563	100.0007
3	-1952.789 496	-1952.823 248	99.9983	-1960.126 237	-1960.121 466	100.0002
4	-1952.821 617	-1952.853 728	99.9984	-1960.152 768	-1960.146 331	100.0003
5	-2343.357 453	-2343.352 655	100.0002	-2352.009 819	-2351.993 178	100.0007
6	-2343.293 026	-2343.303 250	99.9996	-2352.021 019	-2352.002 237	100.0008
7	-2343.178 552	-2343.207 638	99.9988	-2351.960 404	-2351.946 323	100.0006
8	-2343.185 260	-2343.213 895	99.9988	-2351.946 235	-2351.939 182	100.0003
9	-2733.723 678	-2733.718 018	100.0002	-2743.815 660	-2743.796 025	100.0007
10	-2733.674 264	-2733.679 919	99.9998	-2743.812 522	-2743.793 968	100.0007
11	-2733.674 206	-2733.679 882	99.9998	-2743.812 512	-2743.793 960	100.0007
12	-2733.569 394	-2733.593 251	99.9991	-2743.768 750	-2743.758 118	100.0004
**MAE**			**9.94**			**8.45**
Δ_{max}			**21.18**			**12.32**

Table S13: Total energies (in hartrees) from HF, MP2, LSSMF-HF, and LSSMF-MP2 methods, and percentages of the LSSMF energies, and the MAE and Δ_{max} values (in kcal mol^{-1}) of the LSSMF approaches with respect to the canonical methods (Full) for the L12 set. All computations are performed with the cc-pVDZ basis set, LSSMF bonded level 3, nonbonded level 2 and with $\Delta_{nb} = 10$ Å.

	**HF**			**MP2**		
**Molecule**	**LSSMF**	**Full**	**%**	**LSSMF**	**Full**	**%**
1	-1952.991 109	-1952.987 297	100.0002	-1960.203 608	-1960.190 335	100.0007
2	-1952.943 042	-1952.949 313	99.9997	-1960.196 576	-1960.187 563	100.0005
3	-1952.799 984	-1952.823 248	99.9988	-1960.121 940	-1960.121 466	100.0000
4	-1952.832 512	-1952.853 728	99.9989	-1960.147 899	-1960.146 331	100.0001
5	-2343.357 301	-2343.352 655	100.0002	-2352.009 359	-2351.993 178	100.0007
6	-2343.297 426	-2343.303 250	99.9998	-2352.014 347	-2352.002 237	100.0005
7	-2343.190 960	-2343.207 638	99.9993	-2351.953 124	-2351.946 323	100.0003
8	-2343.191 525	-2343.213 895	99.9990	-2351.941 476	-2351.939 182	100.0001
9	-2733.723 498	-2733.718 018	100.0002	-2743.815 114	-2743.796 025	100.0007
10	-2733.674 905	-2733.679 919	99.9998	-2743.808 315	-2743.793 968	100.0005
11	-2733.674 854	-2733.679 882	99.9998	-2743.808 296	-2743.793 960	100.0005
12	-2733.599 327	-2733.593 251	100.0002	-2743.775 762	-2743.758 118	100.0006
**MAE**			**6.57**			**6.65**
Δ_{max}			**14.60**			**11.98**

Table S14: Total energies (in hartrees) from HF, MP2, LSSMF-HF, and LSSMF-MP2 methods, and percentages of the LSSMF energies, and the MAE and Δ_{max} values (in kcal mol^{-1}) of the LSSMF approaches with respect to the canonical methods (Full) for the L12 set. All computations are performed with the cc-pVDZ basis set, LSSMF bonded level 4, nonbonded level 1 and with $\Delta_{nb} = 7.5$ Å.

	**HF**			**MP2**		
**Molecule**	**LSSMF**	**Full**	**%**	**LSSMF**	**Full**	**%**
1	-1952.987 790	-1952.987 297	100.0000	-1960.193 308	-1960.190 335	100.0002
2	-1952.949 200	-1952.949 313	100.0000	-1960.192 217	-1960.187 563	100.0002
3	-1952.814 562	-1952.823 248	99.9996	-1960.125 667	-1960.121 466	100.0002
4	-1952.845 935	-1952.853 728	99.9996	-1960.152 140	-1960.146 331	100.0003
5	-2343.353 260	-2343.352 655	100.0000	-2351.996 823	-2351.993 178	100.0002
6	-2343.299 740	-2343.303 250	99.9999	-2352.009 640	-2352.002 237	100.0003
7	-2343.196 494	-2343.207 638	99.9995	-2351.952 598	-2351.946 323	100.0003
8	-2343.207 786	-2343.213 895	99.9997	-2351.943 075	-2351.939 182	100.0002
9	-2733.718 737	-2733.718 018	100.0000	-2743.800 342	-2743.796 025	100.0002
10	-2733.679 625	-2733.679 919	100.0000	-2743.799 798	-2743.793 968	100.0002
11	-2733.679 577	-2733.679 882	100.0000	-2743.799 786	-2743.793 960	100.0002
12	-2733.588 010	-2733.593 251	99.9998	-2743.760 512	-2743.758 118	100.0001
**MAE**			**2.35**			**2.99**
Δ_{max}			**6.99**			**4.65**

Table S15: Total energies (in hartrees) from HF, MP2, LSSMF-HF, and LSSMF-MP2 methods, and percentages of the LSSMF energies, and the MAE and Δ_{max} values (in kcal mol^{-1}) of the LSSMF approaches with respect to the canonical methods (Full) for the L12 set. All computations are performed with the cc-pVDZ basis set, LSSMF bonded level 4, nonbonded level 2 and with $\Delta_{nb} = 7.5$ Å.

	**HF**			**MP2**		
**Molecule**	**LSSMF**	**Full**	**%**	**LSSMF**	**Full**	**%**
1	-1952.988 090	-1952.987 297	100.0000	-1960.193 296	-1960.190 335	100.0002
2	-1952.949 022	-1952.949 313	100.0000	-1960.190 638	-1960.187 563	100.0002
3	-1952.815 007	-1952.823 248	99.9996	-1960.120 940	-1960.121 466	100.0000
4	-1952.850 885	-1952.853 728	99.9999	-1960.148 385	-1960.146 331	100.0001
5	-2343.353 628	-2343.352 655	100.0000	-2351.996 807	-2351.993 178	100.0002
6	-2343.301 923	-2343.303 250	99.9999	-2352.004 852	-2352.002 237	100.0001
7	-2343.199 831	-2343.207 638	99.9997	-2351.945 155	-2351.946 323	100.0000
8	-2343.207 588	-2343.213 895	99.9997	-2351.939 019	-2351.939 182	100.0000
9	-2733.719 173	-2733.718 018	100.0000	-2743.800 323	-2743.796 025	100.0002
10	-2733.679 482	-2733.679 919	100.0000	-2743.797 794	-2743.793 968	100.0001
11	-2733.679 438	-2733.679 882	100.0000	-2743.797 777	-2743.793 960	100.0001
12	-2733.588 856	-2733.593 251	99.9998	-2743.758 282	-2743.758 118	100.0000
**MAE**			**1.83**			**1.48**
Δ_{max}			**5.17**			**2.70**

Table S16: Total energies (in hartrees) from HF, MP2, LSSMF-HF, and LSSMF-MP2 methods, and percentages of the LSSMF energies, and the MAE and Δ_{max} values (in kcal mol^{-1}) of the LSSMF approaches with respect to the canonical methods (Full) for the L12 set. All computations are performed with the cc-pVDZ basis set, LSSMF bonded level 4, nonbonded level 1 and with $\Delta_{nb} = 10$ Å.

	HF			MP2		
**Molecule**	**LSSMF**	**Full**	**%**	**LSSMF**	**Full**	**%**
1	-1952.987 805	-1952.987 297	100.0000	-1960.193 400	-1960.190 335	100.0002
2	-1952.949 225	-1952.949 313	100.0000	-1960.192 371	-1960.187 563	100.0002
3	-1952.814 578	-1952.823 248	99.9996	-1960.125 771	-1960.121 466	100.0002
4	-1952.845 954	-1952.853 728	99.9996	-1960.152 254	-1960.146 331	100.0003
5	-2343.353 280	-2343.352 655	100.0000	-2351.996 938	-2351.993 178	100.0002
6	-2343.299 778	-2343.303 250	99.9999	-2352.009 873	-2352.002 237	100.0003
7	-2343.196 531	-2343.207 638	99.9995	-2351.952 828	-2351.946 323	100.0003
8	-2343.207 824	-2343.213 895	99.9997	-2351.943 304	-2351.939 182	100.0002
9	-2733.718 760	-2733.718 018	100.0000	-2743.800 481	-2743.796 025	100.0002
10	-2733.679 670	-2733.679 919	100.0000	-2743.800 061	-2743.793 968	100.0002
11	-2733.679 624	-2733.679 882	100.0000	-2743.800 059	-2743.793 960	100.0002
12	-2733.588 055	-2733.593 251	99.9998	-2743.760 782	-2743.758 118	100.0001
**MAE**			**2.34**			**3.11**
Δ_{max}			**6.97**			**4.79**

Table S17: Total energies (in hartrees) from HF, MP2, LSSMF-HF, and LSSMF-MP2 methods, and percentages of the LSSMF energies, and the MAE and Δ_{max} values (in kcal mol^{-1}) of the LSSMF approaches with respect to the canonical methods (Full) for the L12 set. All computations are performed with the cc-pVDZ basis set, LSSMF bonded level 4, nonbonded level 2 and with Δ_{nb} = 10 Å.

	HF			MP2		
**Molecule**	**LSSMF**	**Full**	**%**	**LSSMF**	**Full**	**%**
1	-1952.988 061	-1952.987 297	100.0000	-1960.193 302	-1960.190 335	100.0002
2	-1952.948 971	-1952.949 313	100.0000	-1960.190 650	-1960.187 563	100.0002
3	-1952.814 975	-1952.823 248	99.9996	-1960.120 951	-1960.121 466	100.0000
4	-1952.850 849	-1952.853 728	99.9999	-1960.148 396	-1960.146 331	100.0001
5	-2343.353 592	-2343.352 655	100.0000	-2351.996 815	-2351.993 178	100.0002
6	-2343.301 848	-2343.303 250	99.9999	-2352.004 873	-2352.002 237	100.0001
7	-2343.199 758	-2343.207 638	99.9997	-2351.945 179	-2351.946 323	100.0000
8	-2343.207 510	-2343.213 895	99.9997	-2351.939 035	-2351.939 182	100.0000
9	-2733.719 130	-2733.718 018	100.0000	-2743.800 333	-2743.796 025	100.0002
10	-2733.679 393	-2733.679 919	100.0000	-2743.797 811	-2743.793 968	100.0001
11	-2733.679 344	-2733.679 882	100.0000	-2743.797 794	-2743.793 960	100.0001
12	-2733.588 768	-2733.593 251	99.9998	-2743.758 303	-2743.758 118	100.0000
**MAE**			**1.86**			**1.48**
Δ_{max}			**5.19**			**2.70**

Table S18: Total energies (in hartrees) from HF, MP2, LSSMF-HF, and LSSMF-MP2 methods, and percentages of the LSSMF energies, and the MAE and Δ_{max} values (in kcal mol^{-1}) of the LSSMF approaches with respect to the canonical methods (Full) for the L12 set. All computations are performed with the cc-pVDZ basis set, LSSMF bonded level 5, nonbonded level 1 and with $\Delta_{nb} = 7.5$ Å.

	**HF**			**MP2**		
**Molecule**	**LSSMF**	**Full**	**%**	**LSSMF**	**Full**	**%**
1	-1952.987 717	-1952.987 297	100.0000	-1960.191 915	-1960.190 335	100.0001
2	-1952.950 006	-1952.949 313	100.0000	-1960.189 904	-1960.187 563	100.0001
3	-1952.822 869	-1952.823 248	100.0000	-1960.125 435	-1960.121 466	100.0002
4	-1952.851 856	-1952.853 728	99.9999	-1960.150 158	-1960.146 331	100.0002
5	-2343.353 173	-2343.352 655	100.0000	-2351.995 122	-2351.993 178	100.0001
6	-2343.301 593	-2343.303 250	99.9999	-2352.007 238	-2352.002 237	100.0002
7	-2343.204 852	-2343.207 638	99.9999	-2351.953 218	-2351.946 323	100.0003
8	-2343.214 312	-2343.213 895	100.0000	-2351.942 973	-2351.939 182	100.0002
9	-2733.718 635	-2733.718 018	100.0000	-2743.798 334	-2743.796 025	100.0001
10	-2733.680 808	-2733.679 919	100.0000	-2743.797 177	-2743.793 968	100.0001
11	-2733.680 767	-2733.679 882	100.0000	-2743.797 168	-2743.793 960	100.0001
12	-2733.593 161	-2733.593 251	100.0000	-2743.760 641	-2743.758 118	100.0001
**MAE**			**0.59**			**2.12**
Δ_{max}			**1.75**			**4.33**

Table S19: Total energies (in hartrees) from HF, MP2, LSSMF-HF, and LSSMF-MP2 methods, and percentages of the LSSMF energies, and the MAE and Δ_{max} values (in kcal mol^{-1}) of the LSSMF approaches with respect to the canonical methods (Full) for the L12 set. All computations are performed with the cc-pVDZ basis set, LSSMF bonded level 5, nonbonded level 2 and with $\Delta_{nb} = 7.5$ Å.

	HF			MP2		
**Molecule**	**LSSMF**	**Full**	**%**	**LSSMF**	**Full**	**%**
1	-1952.987 687	-1952.987 297	100.0000	-1960.191 765	-1960.190 335	100.0001
2	-1952.949 883	-1952.949 313	100.0000	-1960.189 180	-1960.187 563	100.0001
3	-1952.822 316	-1952.823 248	100.0000	-1960.122 914	-1960.121 466	100.0001
4	-1952.851 952	-1952.853 728	99.9999	-1960.146 683	-1960.146 331	100.0000
5	-2343.353 136	-2343.352 655	100.0000	-2351.994 937	-2351.993 178	100.0001
6	-2343.302 846	-2343.303 250	100.0000	-2352.003 474	-2352.002 237	100.0001
7	-2343.205 336	-2343.207 638	99.9999	-2351.946 937	-2351.946 323	100.0000
8	-2343.213 831	-2343.213 895	100.0000	-2351.940 951	-2351.939 182	100.0001
9	-2733.718 591	-2733.718 018	100.0000	-2743.798 114	-2743.796 025	100.0001
10	-2733.680 582	-2733.679 919	100.0000	-2743.796 125	-2743.793 968	100.0001
11	-2733.680 540	-2733.679 882	100.0000	-2743.796 112	-2743.793 960	100.0001
12	-2733.592 942	-2733.593 251	100.0000	-2743.759 010	-2743.758 118	100.0000
**MAE**			**0.48**			**0.92**
Δ_{max}			**1.44**			**1.35**

Table S20: Total energies (in hartrees) from HF, MP2, LSSMF-HF, and LSSMF-MP2 methods, and percentages of the LSSMF energies, and the MAE and Δ_{max} values (in kcal mol^{-1}) of the LSSMF approaches with respect to the canonical methods (Full) for the L12 set. All computations are performed with the cc-pVDZ basis set, LSSMF bonded level 5, nonbonded level 1 and with $\Delta_{nb} = 10$ Å.

	HF			MP2		
**Molecule**	**LSSMF**	**Full**	**%**	**LSSMF**	**Full**	**%**
1	-1952.987 733	-1952.987 297	100.0000	-1960.192 007	-1960.190 335	100.0001
2	-1952.950 032	-1952.949 313	100.0000	-1960.190 057	-1960.187 563	100.0001
3	-1952.822 885	-1952.823 248	100.0000	-1960.125 539	-1960.121 466	100.0002
4	-1952.851 874	-1952.853 728	99.9999	-1960.150 272	-1960.146 331	100.0002
5	-2343.353 193	-2343.352 655	100.0000	-2351.995 238	-2351.993 178	100.0001
6	-2343.301 631	-2343.303 250	99.9999	-2352.007 471	-2352.002 237	100.0002
7	-2343.204 888	-2343.207 638	99.9999	-2351.953 448	-2351.946 323	100.0003
8	-2343.214 349	-2343.213 895	100.0000	-2351.943 203	-2351.939 182	100.0002
9	-2733.718 658	-2733.718 018	100.0000	-2743.798 472	-2743.796 025	100.0001
10	-2733.680 853	-2733.679 919	100.0000	-2743.797 440	-2743.793 968	100.0001
11	-2733.680 814	-2733.679 882	100.0000	-2743.797 441	-2743.793 960	100.0001
12	-2733.593 206	-2733.593 251	100.0000	-2743.760 911	-2743.758 118	100.0001
**MAE**			**0.59**			**2.24**
Δ_{max}			**1.73**			**4.47**

Table S21: Total energies (in hartrees) from HF, MP2, LSSMF-HF, and LSSMF-MP2 methods, and percentages of the LSSMF energies, and the MAE and Δ_{max} values (in kcal mol^{-1}) of the LSSMF approaches with respect to the canonical methods (Full) for the L12 set. All computations are performed with the cc-pVDZ basis set, LSSMF bonded level 5, nonbonded level 2 and with $\Delta_{nb} = 10$ Å.

	**HF**			**MP2**		
**Molecule**	**LSSMF**	**Full**	**%**	**LSSMF**	**Full**	**%**
1	-1952.987 658	-1952.987 297	100.0000	-1960.191 771	-1960.190 335	100.0001
2	-1952.949 833	-1952.949 313	100.0000	-1960.189 192	-1960.187 563	100.0001
3	-1952.822 285	-1952.823 248	100.0000	-1960.122 925	-1960.121 466	100.0001
4	-1952.851 915	-1952.853 728	99.9999	-1960.146 694	-1960.146 331	100.0000
5	-2343.353 100	-2343.352 655	100.0000	-2351.994 945	-2351.993 178	100.0001
6	-2343.302 771	-2343.303 250	100.0000	-2352.003 495	-2352.002 237	100.0001
7	-2343.205 263	-2343.207 638	99.9999	-2351.946 961	-2351.946 323	100.0000
8	-2343.213 753	-2343.213 895	100.0000	-2351.940 966	-2351.939 182	100.0001
9	-2733.718 548	-2733.718 018	100.0000	-2743.798 123	-2743.796 025	100.0001
10	-2733.680 493	-2733.679 919	100.0000	-2743.796 142	-2743.793 968	100.0001
11	-2733.680 446	-2733.679 882	100.0000	-2743.796 129	-2743.793 960	100.0001
12	-2733.592 854	-2733.593 251	100.0000	-2743.759 032	-2743.758 118	100.0000
**MAE**			**0.48**			**0.92**
Δ_{max}			**1.49**			**1.36**

Table S22: Total energies (in hartrees) from HF, MP2, LSSMF-HF, and LSSMF-MP2 methods, and percentages of the LSSMF energies, and the MAE and Δ_{max} values (in kcal mol^{-1}) of the LSSMF approaches with respect to the canonical methods (Full) for the L12 set. All computations are performed with the cc-pVDZ basis set, LSSMF bonded level 6, nonbonded level 1 and with $\Delta_{nb} = 7.5$ Å.

	HF			MP2		
**Molecule**	**LSSMF**	**Full**	**%**	**LSSMF**	**Full**	**%**
1	-1952.987 508	-1952.987 297	100.0000	-1960.190 910	-1960.190 335	100.0000
2	-1952.949 502	-1952.949 313	100.0000	-1960.188 295	-1960.187 563	100.0000
3	-1952.823 352	-1952.823 248	100.0000	-1960.122 994	-1960.121 466	100.0001
4	-1952.852 981	-1952.853 728	100.0000	-1960.148 221	-1960.146 331	100.0001
5	-2343.352 916	-2343.352 655	100.0000	-2351.993 889	-2351.993 178	100.0000
6	-2343.302 182	-2343.303 250	100.0000	-2352.005 577	-2352.002 237	100.0001
7	-2343.207 159	-2343.207 638	100.0000	-2351.951 627	-2351.946 323	100.0002
8	-2343.214 483	-2343.213 895	100.0000	-2351.940 783	-2351.939 182	100.0001
9	-2733.718 331	-2733.718 018	100.0000	-2743.796 873	-2743.796 025	100.0000
10	-2733.680 289	-2733.679 919	100.0000	-2743.795 120	-2743.793 968	100.0000
11	-2733.680 252	-2733.679 882	100.0000	-2743.795 111	-2743.793 960	100.0000
12	-2733.593 742	-2733.593 251	100.0000	-2743.759 457	-2743.758 118	100.0000
**MAE**			**0.27**			**1.05**
Δ_{max}			**0.67**			**3.33**

Table S23: Total energies (in hartrees) from HF, MP2, LSSMF-HF, and LSSMF-MP2 methods, and percentages of the LSSMF energies, and the MAE and Δ_{max} values (in kcal mol^{-1}) of the LSSMF approaches with respect to the canonical methods (Full) for the L12 set. All computations are performed with the cc-pVDZ basis set, LSSMF bonded level 6, nonbonded level 2 and with $\Delta_{nb} = 7.5$ Å.

	HF			MP2		
**Molecule**	**LSSMF**	**Full**	**%**	**LSSMF**	**Full**	**%**
1	-1952.987 545	-1952.987 297	100.0000	-1960.190 904	-1960.190 335	100.0000
2	-1952.949 443	-1952.949 313	100.0000	-1960.187 933	-1960.187 563	100.0000
3	-1952.823 137	-1952.823 248	100.0000	-1960.121 907	-1960.121 466	100.0000
4	-1952.853 224	-1952.853 728	100.0000	-1960.146 275	-1960.146 331	100.0000
5	-2343.352 962	-2343.352 655	100.0000	-2351.993 881	-2351.993 178	100.0000
6	-2343.302 977	-2343.303 250	100.0000	-2352.002 461	-2352.002 237	100.0000
7	-2343.207 186	-2343.207 638	100.0000	-2351.946 861	-2351.946 323	100.0000
8	-2343.214 248	-2343.213 895	100.0000	-2351.939 740	-2351.939 182	100.0000
9	-2733.718 385	-2733.718 018	100.0000	-2743.796 863	-2743.796 025	100.0000
10	-2733.680 198	-2733.679 919	100.0000	-2743.794 601	-2743.793 968	100.0000
11	-2733.680 161	-2733.679 882	100.0000	-2743.794 590	-2743.793 960	100.0000
12	-2733.593 570	-2733.593 251	100.0000	-2743.758 625	-2743.758 118	100.0000
**MAE**			**0.19**			**0.32**
Δ_{max}			**0.32**			**0.53**

Table S24: Total energies (in hartrees) from HF, MP2, LSSMF-HF, and LSSMF-MP2 methods, and percentages of the LSSMF energies, and the MAE and Δ_{max} values (in kcal mol^{-1}) of the LSSMF approaches with respect to the canonical methods (Full) for the L12 set. All computations are performed with the cc-pVDZ basis set, LSSMF bonded level 6, nonbonded level 1 and with $\Delta_{nb} = 10$ Å.

	**HF**			**MP2**		
**Molecule**	**LSSMF**	**Full**	**%**	**LSSMF**	**Full**	**%**
1	-1952.987 524	-1952.987 297	100.0000	-1960.191 003	-1960.190 335	100.0000
2	-1952.949 527	-1952.949 313	100.0000	-1960.188 449	-1960.187 563	100.0000
3	-1952.823 368	-1952.823 248	100.0000	-1960.123 098	-1960.121 466	100.0001
4	-1952.852 999	-1952.853 728	100.0000	-1960.148 334	-1960.146 331	100.0001
5	-2343.352 936	-2343.352 655	100.0000	-2351.994 004	-2351.993 178	100.0000
6	-2343.302 221	-2343.303 250	100.0000	-2352.005 810	-2352.002 237	100.0002
7	-2343.207 195	-2343.207 638	100.0000	-2351.951 857	-2351.946 323	100.0002
8	-2343.214 520	-2343.213 895	100.0000	-2351.941 012	-2351.939 182	100.0001
9	-2733.718 354	-2733.718 018	100.0000	-2743.797 011	-2743.796 025	100.0000
10	-2733.680 334	-2733.679 919	100.0000	-2743.795 383	-2743.793 968	100.0001
11	-2733.680 299	-2733.679 882	100.0000	-2743.795 383	-2743.793 960	100.0001
12	-2733.593 787	-2733.593 251	100.0000	-2743.759 726	-2743.758 118	100.0001
**MAE**			**0.28**			**1.17**
Δ_{max}			**0.65**			**3.47**

Table S25: Total energies (in hartrees) from HF, MP2, LSSMF-HF, and LSSMF-MP2 methods, and percentages of the LSSMF energies, and the MAE and Δ_{max} values (in kcal mol^{-1}) of the LSSMF approaches with respect to the canonical methods (Full) for the L12 set. All computations are performed with the cc-pVDZ basis set, LSSMF bonded level 6, nonbonded level 2 and with $\Delta_{nb} = 10$ Å.

	**HF**			**MP2**		
**Molecule**	**LSSMF**	**Full**	**%**	**LSSMF**	**Full**	**%**
1	-1952.987 516	-1952.987 297	100.0000	-1960.190 910	-1960.190 335	100.0000
2	-1952.949 393	-1952.949 313	100.0000	-1960.187 946	-1960.187 563	100.0000
3	-1952.823 105	-1952.823 248	100.0000	-1960.121 918	-1960.121 466	100.0000
4	-1952.853 188	-1952.853 728	100.0000	-1960.146 286	-1960.146 331	100.0000
5	-2343.352 926	-2343.352 655	100.0000	-2351.993 889	-2351.993 178	100.0000
6	-2343.302 902	-2343.303 250	100.0000	-2352.002 482	-2352.002 237	100.0000
7	-2343.207 113	-2343.207 638	100.0000	-2351.946 885	-2351.946 323	100.0000
8	-2343.214 170	-2343.213 895	100.0000	-2351.939 756	-2351.939 182	100.0000
9	-2733.718 342	-2733.718 018	100.0000	-2743.796 872	-2743.796 025	100.0000
10	-2733.680 109	-2733.679 919	100.0000	-2743.794 618	-2743.793 968	100.0000
11	-2733.680 067	-2733.679 882	100.0000	-2743.794 607	-2743.793 960	100.0000
12	-2733.593 482	-2733.593 251	100.0000	-2743.758 647	-2743.758 118	100.0000
**MAE**			**0.17**			**0.33**
Δ_{max}			**0.34**			**0.53**

Optimized Geometries at the B3LYP/cc-pVDZ Level

2,2-Dimethylbutane

0 1

C	-1.6150520000	0.9917020000	0.0002090000
H	-1.5890060000	1.6425770000	-0.8899920000
H	-1.5890360000	1.6421660000	0.8907100000
H	-2.5822070000	0.4617070000	0.0000700000
C	-0.4365420000	-0.0008130000	0.0000000000
C	0.8796060000	0.8215030000	0.0001550000
H	0.8643090000	1.4885380000	0.8817220000
H	0.8643080000	1.4888660000	-0.8811640000
C	2.1936690000	0.0335330000	0.0000110000
H	2.2923640000	-0.6093960000	-0.8895290000
H	3.0540500000	0.7224030000	0.0001300000
H	2.2923670000	-0.6097110000	0.8893220000
C	-0.5308720000	-0.8845060000	-1.2582330000
H	-0.4484140000	-0.2798530000	-2.1773970000
H	0.2627520000	-1.6480580000	-1.2850830000
H	-1.4977620000	-1.4138740000	-1.2923650000
C	-0.5308640000	-0.8850230000	1.2578730000
H	0.2627510000	-1.6485970000	1.2843990000
H	-0.4483840000	-0.2807490000	2.1772850000
H	-1.4977610000	-1.4143900000	1.2917970000

2,3-Dimethylbutane

0 1

C	-1.8083530000	-0.7473550000	-0.5489750000
H	-1.8693070000	-1.4187560000	0.3240300000
H	-1.6280200000	-1.3718900000	-1.4397620000
H	-2.7977500000	-0.2752300000	-0.6659390000
C	-0.7115940000	0.3134200000	-0.3751660000
H	-0.7510520000	0.9676100000	-1.2672930000
C	0.7115830000	-0.3134270000	-0.3751780000
H	0.7510170000	-0.9676370000	-1.2672920000
C	1.8083380000	0.7473430000	-0.5490400000
H	1.8693180000	1.4187610000	0.3239490000
H	2.7977320000	0.2752140000	-0.6660230000
H	1.6279810000	1.3718590000	-1.4398350000
C	-1.0065400000	1.1946950000	0.8484910000
H	-1.0464570000	0.6007720000	1.7771220000
H	-0.2507200000	1.9838700000	0.9872290000
H	-1.9841010000	1.6925760000	0.7388700000
C	1.0065630000	-1.1946770000	0.8484890000
H	0.2507430000	-1.9838450000	0.9872670000
H	1.9841190000	-1.6925640000	0.7388470000
H	1.0465120000	-0.6007360000	1.7771060000

2-Methylpentane

0 1

C	2.1566130000	-1.0381580000	0.0338140000
H	1.9232300000	-2.0353120000	-0.3747390000
H	2.2469010000	-1.1405050000	1.1297630000
H	3.1441590000	-0.7380660000	-0.3537000000
C	1.0752170000	-0.0129270000	-0.3333700000
H	1.0277590000	0.0456700000	-1.4386470000
C	-0.3050800000	-0.4814160000	0.1647390000
H	-0.2939920000	-0.5160290000	1.2719550000
H	-0.4600090000	-1.5252540000	-0.1667060000
C	-1.4987900000	0.3587150000	-0.3041250000
H	-1.4827030000	0.4274550000	-1.4079690000
H	-1.4009720000	1.3941150000	0.0663400000
C	-2.8446920000	-0.2114470000	0.1512620000
H	-2.9043780000	-0.2656350000	1.2518760000
H	-3.6862320000	0.4087500000	-0.1975830000
H	-2.9995390000	-1.2329130000	-0.2367170000
C	1.4525450000	1.3783850000	0.1952000000
H	0.7361290000	2.1530310000	-0.1201430000
H	1.4868380000	1.3828140000	1.2994540000
H	2.4479280000	1.6829620000	-0.1683110000

3-Methylpentane

0 1

C	2.2414470000	-0.9122040000	-0.1623500000
H	2.2091490000	-1.1120810000	-1.2474290000
H	2.7528250000	-1.7636810000	0.3153830000
H	2.8740140000	-0.0234420000	-0.0081330000
C	0.8315200000	-0.7290160000	0.4085920000
H	0.2916820000	-1.6860800000	0.3124350000
H	0.8951300000	-0.5242150000	1.4946690000
C	-0.0006650000	0.3861890000	-0.2550490000
H	-0.0085370000	0.1931200000	-1.3470160000
C	-1.4663440000	0.3772940000	0.2260070000
H	-1.4814540000	0.4891830000	1.3271090000
H	-1.9660470000	1.2780850000	-0.1732790000
C	-2.2846960000	-0.8530320000	-0.1773360000
H	-1.8963940000	-1.7817950000	0.2706520000
H	-3.3335660000	-0.7507750000	0.1454050000
H	-2.2870170000	-0.9889450000	-1.2726680000
C	0.6171240000	1.7733650000	-0.0273820000
H	0.6509180000	2.0160360000	1.0497390000
H	1.6448130000	1.8409820000	-0.4163490000
H	0.0241720000	2.5580310000	-0.5254110000

n-Hexane

0 1

C	-3.2226320000	0.2079950000	0.0000010000
H	-3.3149990000	0.8553050000	-0.8889690000
H	-3.3149990000	0.8553000000	0.8889730000
H	-4.0817110000	-0.4823040000	-0.0000010000
C	-1.8922760000	-0.5485730000	-0.0000020000
H	-1.8460270000	-1.2138730000	0.8821310000
H	-1.8460280000	-1.2138700000	-0.8821380000
C	-0.6685720000	0.3737180000	0.0000000000
H	-0.7148940000	1.0400740000	-0.8825360000
H	-0.7148940000	1.0400700000	0.8825380000
C	0.6685720000	-0.3737180000	-0.0000010000
H	0.7148940000	-1.0400700000	-0.8825390000
H	0.7148940000	-1.0400730000	0.8825350000
C	1.8922760000	0.5485730000	0.0000010000
H	1.8460270000	1.2138710000	0.8821370000
H	1.8460280000	1.2138730000	-0.8821330000
C	3.2226320000	-0.2079950000	0.0000010000
H	3.3149990000	-0.8553020000	-0.8889700000
H	4.0817110000	0.4823040000	0.0000030000
H	3.3149980000	-0.8553030000	0.8889720000

2,2,3-Trimethylbutane

0 1

C	1.3588020000	1.2446800000	-0.5692610000
H	0.9806530000	2.1801060000	-0.1294640000
H	1.2338080000	1.3108680000	-1.6635240000
H	2.4403650000	1.1994910000	-0.3601350000
C	0.6467060000	-0.0000010000	0.0004120000
C	-1.6407410000	-1.2523180000	-0.0164750000
H	-1.7341180000	-1.3168340000	1.0806900000
H	-1.1778570000	-2.1852080000	-0.3706730000
C	-0.8585580000	0.0000030000	-0.4476140000
C	-1.6407400000	1.2523180000	-0.0164540000
H	-2.6637410000	1.2192350000	-0.4257690000
H	-1.1778900000	2.1852090000	-0.3706940000
H	-0.8327360000	0.0000140000	-1.5546620000
H	-2.6637210000	-1.2192590000	-0.4258430000
H	-1.7340610000	1.3168530000	1.0807140000
C	0.7974940000	-0.0000830000	1.5342300000
H	0.3396660000	-0.8907880000	1.9930950000
H	1.8643020000	-0.0000590000	1.8140920000
H	0.3395990000	0.8905340000	1.9931970000
C	1.3588340000	-1.2446010000	-0.5694000000
H	1.2338720000	-1.3106480000	-1.6636740000
H	2.4403900000	-1.1994250000	-0.3602370000
H	0.9806850000	-2.1800880000	-0.1297350000

2,2-Dimethylhexane

0 1

C	1.9099940000	1.2259700000	-0.0002120000
H	1.7598490000	1.8593330000	0.8901380000
H	1.7598560000	1.8590300000	-0.8907780000
H	2.9601930000	0.8896030000	-0.0001510000
C	0.9421610000	0.0270860000	-0.0000150000
C	-0.5059720000	0.5838920000	-0.0000920000
H	-0.6194890000	1.2426590000	-0.8819480000
H	-0.6194730000	1.2429390000	0.8815560000
C	-1.6568350000	-0.4302160000	0.0000770000
H	-1.5804950000	-1.0893360000	0.8824790000
H	-1.5805280000	-1.0895920000	-0.8821360000
C	-3.0306600000	0.2470860000	0.0000050000
H	-3.1619310000	0.8876740000	-0.8889530000
H	-3.8464700000	-0.4938390000	0.0001220000
H	-3.1619020000	0.8879230000	0.8887890000
C	1.2050570000	-0.8226110000	-1.2581880000
H	0.5745990000	-1.7256060000	-1.2859880000
H	1.0079260000	-0.2453140000	-2.1775210000
H	2.2562020000	-1.1550000000	-1.2921720000
C	1.2050700000	-0.8222160000	1.2584210000
H	2.2562030000	-1.1546360000	1.2924740000
H	1.0079940000	-0.2446130000	2.1775750000
H	0.5745740000	-1.7251740000	1.2865320000

2,3-Dimethylpentane

0 1

C	2.2004270000	0.6641440000	-0.7968060000
H	2.4352400000	1.3563860000	0.0292320000
H	1.8985280000	1.2715020000	-1.6664070000
H	3.1367940000	0.1452270000	-1.0604920000
C	1.1087160000	-0.3439600000	-0.4081480000
C	-1.4192680000	-0.7010340000	-0.1780610000
H	-1.3705960000	-1.2774050000	0.7638310000
C	-2.8247940000	-0.1105500000	-0.3317190000
H	-2.9145030000	0.4700770000	-1.2661190000
H	-3.5863570000	-0.9066720000	-0.3618060000
H	-3.0881100000	0.5605310000	0.5016360000
H	0.9860880000	-1.0292260000	-1.2686130000
H	-1.2530470000	-1.4320870000	-0.9901140000
C	-0.2767210000	0.3355020000	-0.2112310000
C	-0.3281250000	1.2776680000	1.0020280000
H	-1.2729950000	1.8421900000	1.0293630000
H	0.4873680000	2.0173740000	0.9815460000
H	-0.4373810000	0.9542700000	-1.1161560000
H	-0.2519530000	0.7204460000	1.9510460000
C	1.5648200000	-1.1948050000	0.7874130000
H	0.8221950000	-1.9611190000	1.0603000000
H	2.5060240000	-1.7183630000	0.5516570000
H	1.7523770000	-0.5749260000	1.6802440000

2-4-Dimethylheptane

0 1

C	2.4042230000	-1.0607350000	-0.2603080000
H	2.1175580000	-1.9546560000	-0.8385080000
H	2.6293040000	-1.3883720000	0.7702000000
H	3.3376480000	-0.6639770000	-0.6933010000
C	1.2888070000	-0.0044330000	-0.2673600000
H	1.0849920000	0.2527720000	-1.3247770000
C	-0.0176080000	-0.5736630000	0.3425320000
H	0.0283190000	-1.6765240000	0.3069750000
H	-0.0651250000	-0.3142700000	1.4180000000
C	-1.3385710000	-0.1570790000	-0.3372550000
H	-1.2699130000	-0.4694190000	-1.3980890000
C	-2.5187160000	-0.9107930000	0.2920400000
H	-2.6410780000	-0.6356130000	1.3546180000
H	-3.4651570000	-0.6760470000	-0.2223730000
H	-2.3721500000	-2.0026130000	0.2466020000
C	1.7705750000	1.2678280000	0.4472570000
H	2.0404340000	1.0440520000	1.4946040000
H	2.6658350000	1.6860190000	-0.0428230000
H	1.0017430000	2.0546130000	0.4655350000
C	-1.5995690000	1.3550020000	-0.3174220000
H	-1.6413660000	1.7380810000	0.7175990000
H	-0.8213840000	1.9188030000	-0.8545040000
H	-2.5644970000	1.5903960000	-0.7966700000

2-Methylhexane

0 1

C	2.8236980000	-0.9067450000	-0.0441050000
H	2.6619140000	-1.9083900000	-0.4756100000
H	2.9609970000	-1.0318490000	1.0446070000
H	3.7676420000	-0.5114950000	-0.4542500000
C	1.6465530000	0.0313550000	-0.3433440000
H	1.5525750000	0.1138830000	-1.4441070000
C	1.9241630000	1.4359670000	0.2107990000
H	2.8730920000	1.8362420000	-0.1829830000
H	1.1300630000	2.1519120000	-0.0527710000
H	2.0045920000	1.4147410000	1.3124630000
C	0.3305400000	-0.5659340000	0.1902840000
H	0.2499100000	-1.6088830000	-0.1688090000
H	0.3897180000	-0.6317560000	1.2943870000
C	-0.9458860000	0.1850020000	-0.2057510000
H	-0.9247710000	1.2118310000	0.2015690000
H	-0.9778550000	0.2956530000	-1.3067370000
C	-2.2292170000	-0.5075620000	0.2670970000
H	-2.2630140000	-1.5327550000	-0.1459800000
H	-2.1940180000	-0.6264050000	1.3659900000
C	-3.5034380000	0.2440000000	-0.1249320000
H	-4.4073060000	-0.2787150000	0.2276450000
H	-3.5170260000	1.2603510000	0.3047390000
H	-3.5849870000	0.3491390000	-1.2204470000

3,3-Dimethylheptane

0 1

C	2.6240310000	-0.1950000000	0.0000140000
H	2.7813040000	0.4362550000	0.8893960000
H	2.7813200000	0.4362760000	-0.8893500000
H	3.4183220000	-0.9591240000	0.0000130000
C	1.2451430000	-0.8636800000	-0.0000070000
H	1.1737900000	-1.5279090000	0.8808990000
C	-1.2451430000	-0.8636800000	-0.0000430000
H	-1.1738030000	-1.5279270000	0.8808500000
C	-2.6240310000	-0.1950000000	-0.0000270000
H	-2.7813050000	0.4363020000	-0.8893750000
H	-3.4183210000	-0.9591250000	-0.0000640000
H	-2.7813180000	0.4362290000	0.8893710000
H	1.1738030000	-1.5278770000	-0.8809380000
H	-1.1737900000	-1.5278590000	-0.8809860000
C	0.0000000000	0.0659360000	0.0000020000
C	-0.0000180000	0.9550970000	1.2583180000
H	-0.8848900000	1.6100570000	1.2897790000
H	-0.0000400000	0.3456060000	2.1782360000
H	0.8848620000	1.6100440000	1.2898130000
C	0.0000180000	0.9551710000	-1.2582610000
H	0.0000400000	0.3457330000	-2.1782150000
H	-0.8848630000	1.6101200000	-1.2897190000
H	0.8848900000	1.6101330000	-1.2896850000

3-Ethylpentane

0 1

C	-2.5891170000	-0.6103870000	0.2511780000
H	-2.8033480000	0.4604560000	0.3989120000
H	-2.5867000000	-1.0874610000	1.2465410000
H	-3.4318260000	-1.0356600000	-0.3177470000
C	-1.2630140000	-0.8423240000	-0.4790760000
H	-1.2891990000	-0.3353740000	-1.4610610000
C	1.2630640000	-0.8422580000	-0.4791070000
H	1.2892190000	-0.3352770000	-1.4610780000
C	2.5891680000	-0.6102970000	0.2511370000
H	2.5867810000	-1.0874040000	1.2464830000
H	3.4318850000	-1.0355200000	-0.3178130000
H	2.8033630000	0.4605490000	0.3989040000
H	-1.1668510000	-1.9190950000	-0.7070170000
H	1.1669350000	-1.9190250000	-0.7070790000
C	0.0000230000	-0.4080890000	0.2937520000
C	-0.0000110000	1.0898750000	0.6788440000
H	0.8758720000	1.2896540000	1.3195120000
H	-0.8758480000	1.2895910000	1.3195950000
H	0.0000510000	-0.9707340000	1.2489670000
C	-0.0001050000	2.0843050000	-0.4884650000
H	-0.8886050000	1.9695520000	-1.1310120000
H	-0.0001080000	3.1211930000	-0.1145540000
H	0.8883230000	1.9695990000	-1.1311210000

3-Methlyhexane

0 1

C	-2.3705630000	-1.3006010000	-0.1664120000
H	-1.8867070000	-1.9890770000	0.5446870000
H	-2.1231640000	-1.6476070000	-1.1846780000
H	-3.4593800000	-1.4099400000	-0.0350810000
C	-1.9382080000	0.1536970000	0.0430090000
H	-2.5358560000	0.7980570000	-0.6264460000
H	-2.1922900000	0.4719260000	1.0722490000
C	-0.4451100000	0.4464060000	-0.2154890000
H	-0.2084740000	0.0567510000	-1.2243060000
C	-0.2032120000	1.9626000000	-0.2267890000
H	-0.4184050000	2.4024320000	0.7633400000
H	0.8361750000	2.2185020000	-0.4848360000
H	-0.8569480000	2.4623230000	-0.9604790000
C	0.4606000000	-0.2720950000	0.8059520000
H	0.2168370000	0.1139520000	1.8141350000
H	0.2035810000	-1.3458870000	0.8223370000
C	1.9783590000	-0.1521790000	0.5860200000
H	2.2878810000	0.9066570000	0.6371180000
H	2.4846130000	-0.6485620000	1.4329590000
C	2.4837590000	-0.7691120000	-0.7222140000
H	2.0852710000	-0.2479500000	-1.6081860000
H	3.5833650000	-0.7237570000	-0.7836810000
H	2.1897540000	-1.8301130000	-0.8035990000

n-Heptane

0 1

C	3.2171710000	0.8842760000	0.2852810000
H	2.5889210000	1.7437160000	-0.0003400000
H	3.2354180000	0.8393510000	1.3882070000
H	4.2428730000	1.1008270000	-0.0552130000
C	2.7017540000	-0.4287940000	-0.3125600000
H	2.6997800000	-0.3550810000	-1.4162340000
H	3.4122590000	-1.2373920000	-0.0652510000
C	1.3020380000	-0.8436370000	0.1668920000
H	1.0873170000	-1.8624800000	-0.2043850000
H	1.3039970000	-0.9190170000	1.2713390000
C	0.1666320000	0.0884510000	-0.2738120000
H	0.3379080000	1.1060480000	0.1216760000
H	0.1838900000	0.1848320000	-1.3764850000
C	-1.2203000000	-0.3892860000	0.1693790000
H	-1.2368210000	-0.4905490000	1.2714170000
H	-1.4015240000	-1.4059910000	-0.2286320000
C	-2.3613920000	0.5347540000	-0.2691010000
H	-2.1816020000	1.5498180000	0.1311300000
H	-2.3432560000	0.6371130000	-1.3700840000
C	-3.7422770000	0.0473140000	0.1757680000
H	-3.8046130000	-0.0302140000	1.2748300000
H	-4.5400490000	0.7316460000	-0.1555780000
H	-3.9662530000	-0.9510910000	-0.2374760000

2,2-Dimethylhexane

0 1

C	2.5560560000	1.0885130000	0.0000010000
C	1.4744780000	-0.0090750000	0.0000050000
C	1.6539180000	-0.8793780000	-1.2584830000
C	1.6539250000	-0.8793760000	1.2584950000
C	0.0882180000	0.6883380000	0.0000070000
C	-1.1560850000	-0.2082390000	-0.0000020000
C	-2.4646980000	0.5918310000	-0.0000050000
C	-3.7143580000	-0.2915540000	-0.0000160000
H	2.4702220000	1.7337500000	0.8904390000
H	3.5677660000	0.6495590000	-0.0000030000
H	2.4702150000	1.7337500000	-0.8904360000
H	0.9403120000	-1.7181800000	-1.2868290000
H	1.5124230000	-0.2851290000	-2.1772730000
H	2.6686010000	-1.3102450000	-1.2931890000
H	0.9403230000	-1.7181800000	1.2868440000
H	2.6686100000	-1.3102370000	1.2931990000
H	1.5124300000	-0.2851250000	2.1772850000
H	0.0408760000	1.3551600000	0.8815730000
H	0.0408810000	1.3551710000	-0.8815510000
H	-1.1459880000	-0.8726820000	-0.8827130000
H	-1.1459960000	-0.8726900000	0.8827020000
H	-2.4834870000	1.2585690000	0.8820310000
H	-2.4834780000	1.2585780000	-0.8820350000
H	-4.6369970000	0.3111630000	-0.0000170000
H	-3.7427120000	-0.9447950000	-0.8889770000
H	-3.7427210000	-0.9448040000	0.8889390000

2,3,3-Trimethylpentane

0 1

C	1.5734340000	1.6407110000	-0.0002140000
C	1.1825680000	0.2265170000	-0.4663150000
C	2.3420410000	-0.7323960000	-0.1427260000
C	-0.2226530000	-0.2635140000	0.0453870000
C	-0.5808900000	-1.5854050000	-0.6654320000
C	-0.2147680000	-0.5027750000	1.5685990000
C	-1.2842470000	0.8170120000	-0.3140480000
C	-2.7593310000	0.4393050000	-0.1302770000
H	0.8717890000	2.4144390000	-0.3432760000
H	1.6372330000	1.7057510000	1.0992460000
H	2.5673400000	1.9051180000	-0.3967090000
H	1.0927280000	0.2646200000	-1.5691740000
H	2.1535160000	-1.7631040000	-0.4768600000
H	3.2627110000	-0.3917750000	-0.6440840000
H	2.5565210000	-0.7620480000	0.9385440000
H	-1.5554620000	-1.9705230000	-0.3287390000
H	-0.6336620000	-1.4494430000	-1.7593720000
H	0.1587560000	-2.3736960000	-0.4612210000
H	-1.2084280000	-0.8180430000	1.9241860000
H	0.4931580000	-1.2979120000	1.8501450000
H	0.0606420000	0.4068680000	2.1264140000
H	-1.0859450000	1.7179790000	0.2895380000
H	-1.1289440000	1.1173740000	-1.3670280000
H	-3.4011620000	1.3103850000	-0.3405340000
H	-3.0752610000	-0.3672830000	-0.8101580000
H	-2.9824510000	0.1145610000	0.8992430000

2,3,4-Trimethylpentane

0 1

C	-1.9880760000	1.4432050000	-0.0427320000
C	-1.4796030000	0.0312860000	0.2873170000
C	-1.8348230000	-0.9397210000	-0.8489810000
C	0.0180310000	0.0350990000	0.7179950000
C	0.3848900000	-1.2446010000	1.4858480000
C	1.0338460000	0.3845120000	-0.4162150000
C	2.2191900000	1.1887130000	0.1428760000
C	1.5592890000	-0.8211670000	-1.2146830000
H	-1.8054390000	2.1431640000	0.7894160000
H	-1.4995220000	1.8577120000	-0.9409940000
H	-3.0727470000	1.4332590000	-0.2402710000
H	-2.0376100000	-0.3043530000	1.1824570000
H	-1.4961890000	-1.9674610000	-0.6437460000
H	-2.9275960000	-0.9770280000	-0.9920000000
H	-1.3938650000	-0.6225450000	-1.8086340000
H	0.0921260000	0.8628390000	1.4477120000
H	1.4270160000	-1.2200150000	1.8433150000
H	-0.2619640000	-1.3623390000	2.3706760000
H	0.2706860000	-2.1518580000	0.8714100000
H	0.5108710000	1.0451580000	-1.1305800000
H	2.9325630000	1.4545500000	-0.6549860000
H	1.8833500000	2.1244100000	0.6191400000
H	2.7761180000	0.6104430000	0.9006940000
H	2.1759000000	-0.4752840000	-2.0609870000
H	2.1979770000	-1.4713780000	-0.5942450000
H	0.7518580000	-1.4432260000	-1.6269300000

2,3-Dimethylhexane

0 1

C	-1.9536240000	-1.4499880000	-0.8132610000
C	-1.7490340000	-0.0675940000	-0.1763820000
C	-2.0813830000	-0.1108570000	1.3231560000
C	-0.3448980000	0.5256370000	-0.4933860000
C	-0.3088530000	2.0408770000	-0.2397830000
C	0.8076620000	-0.2019710000	0.2300870000
C	2.2023320000	0.0633900000	-0.3528140000
C	3.3074230000	-0.7100580000	0.3712130000
H	-1.7617600000	-1.4254060000	-1.8988350000
H	-1.2864270000	-2.2099090000	-0.3725510000
H	-2.9879640000	-1.8012720000	-0.6642370000
H	-2.4778410000	0.6145190000	-0.6541110000
H	-1.9650770000	0.8730070000	1.8049890000
H	-3.1246220000	-0.4315050000	1.4793210000
H	-1.4362920000	-0.8259270000	1.8606650000
H	-0.1930140000	0.3746170000	-1.5800950000
H	0.6420500000	2.4871420000	-0.5701670000
H	-1.1184670000	2.5540370000	-0.7847140000
H	-0.4264320000	2.2768540000	0.8315740000
H	0.8065540000	0.0777700000	1.3001320000
H	0.6297340000	-1.2915830000	0.2030050000
H	2.2040430000	-0.2088090000	-1.4246820000
H	2.4309170000	1.1428080000	-0.3136330000
H	4.2975570000	-0.5104330000	-0.0696550000
H	3.3559800000	-0.4337930000	1.4385260000
H	3.1333130000	-1.7987270000	0.3214800000

2,4-Dimethylhexane

0 1

C	2.9445160000	0.2368470000	-0.7722480000
C	1.6347150000	-0.3222840000	-0.1981410000
C	1.8778980000	-0.9473510000	1.1825630000
C	0.5604950000	0.7838770000	-0.1671160000
C	-0.8609940000	0.3982690000	0.2927280000
C	-1.7151820000	1.6674030000	0.4343930000
C	-1.5137790000	-0.6329690000	-0.6494230000
C	-2.8749750000	-1.1629670000	-0.1871930000
H	2.7961440000	0.6571120000	-1.7807200000
H	3.3430930000	1.0431420000	-0.1313990000
H	3.7185290000	-0.5449750000	-0.8432180000
H	1.2938960000	-1.1242310000	-0.8786600000
H	0.9763740000	-1.4352890000	1.5853040000
H	2.6721630000	-1.7106860000	1.1357490000
H	2.1965890000	-0.1806510000	1.9113810000
H	0.9290880000	1.5951090000	0.4892670000
H	0.4869990000	1.2287160000	-1.1780330000
H	-0.7880450000	-0.0648010000	1.2953780000
H	-2.7182900000	1.4513990000	0.8329610000
H	-1.2385380000	2.3906200000	1.1163250000
H	-1.8432070000	2.1674790000	-0.5422740000
H	-1.6140380000	-0.1816850000	-1.6553810000
H	-0.8312640000	-1.4911920000	-0.7712100000
H	-3.2434960000	-1.9484480000	-0.8668410000
H	-2.8098830000	-1.6033950000	0.8229300000
H	-3.6422870000	-0.3731780000	-0.1549350000

2,5-Dimethylhexane

0 1

C	-3.0875490000	-0.8367940000	-0.4733720000
C	-1.9774630000	0.2095250000	-0.3063860000
C	-2.2764820000	1.1242070000	0.8898840000
C	-0.6014490000	-0.4760350000	-0.1976880000
C	0.6014480000	0.4760350000	-0.1976870000
C	1.9774620000	-0.2095240000	-0.3063930000
C	3.0875470000	0.8367970000	-0.4733800000
C	2.2764870000	-1.1242100000	0.8898720000
H	-2.9101250000	-1.4766880000	-1.3535210000
H	-3.1440300000	-1.4954170000	0.4112990000
H	-4.0743080000	-0.3607320000	-0.5961880000
H	-1.9654110000	0.8380450000	-1.2185940000
H	-1.5401260000	1.9371950000	0.9879370000
H	-3.2708940000	1.5903040000	0.7917550000
H	-2.2704040000	0.5507210000	1.8341910000
H	-0.5928480000	-1.1006250000	0.7148020000
H	-0.4951290000	-1.1778140000	-1.0462040000
H	0.4951250000	1.1778200000	-1.0461980000
H	0.5928500000	1.1006200000	0.7148070000
H	1.9654060000	-0.8380400000	-1.2186040000
H	4.0743050000	0.3607350000	-0.5962040000
H	2.9101170000	1.4766950000	-1.3535250000
H	3.1440320000	1.4954150000	0.4112940000
H	3.2708990000	-1.5903060000	0.7917370000
H	2.2704120000	-0.5507280000	1.8341820000
H	1.5401320000	-1.9372000000	0.9879250000

2-Methylheptane

0 1

C	-3.3831360000	-0.9927660000	-0.0151770000
C	-2.2605370000	0.0074520000	-0.3229020000
C	-2.5861660000	1.3827400000	0.2768660000
C	-0.9028910000	-0.5408950000	0.1561180000
C	0.3261830000	0.2736070000	-0.2638790000
C	1.6524790000	-0.3684740000	0.1577490000
C	2.8878650000	0.4389540000	-0.2546450000
C	4.2070390000	-0.2109990000	0.1689630000
H	-3.1870110000	-1.9743790000	-0.4774420000
H	-3.4820520000	-1.1517390000	1.0732390000
H	-4.3563980000	-0.6331830000	-0.3878260000
H	-2.2037530000	0.1224150000	-1.4232950000
H	-1.8347220000	2.1421190000	0.0098380000
H	-3.5641600000	1.7478980000	-0.0780320000
H	-2.6320820000	1.3294090000	1.3794320000
H	-0.9233550000	-0.6317770000	1.2597900000
H	-0.7883470000	-1.5717100000	-0.2277840000
H	0.3185000000	0.4042620000	-1.3628920000
H	0.2712500000	1.2911350000	0.1626910000
H	1.6583330000	-0.5075320000	1.2557330000
H	1.7212510000	-1.3851240000	-0.2742260000
H	2.8808630000	0.5789460000	-1.3515830000
H	2.8203640000	1.4539340000	0.1792220000
H	5.0748520000	0.3932840000	-0.1413250000
H	4.2598450000	-0.3324720000	1.2645270000
H	4.3216080000	-1.2132010000	-0.2786250000

3,3-Dimethylhexane

0 1

C	-2.2101680000	-1.6324730000	0.0199720000
C	-1.8461440000	-0.2758320000	-0.5933800000
C	-0.6030310000	0.4613750000	-0.0201070000
C	-0.4821600000	1.8002760000	-0.7746990000
C	-0.8048880000	0.7508870000	1.4805330000
C	0.6618400000	-0.4074840000	-0.2526080000
C	2.0140570000	0.1833340000	0.1697800000
C	3.1759030000	-0.7795930000	-0.0932520000
H	-3.1162780000	-2.0367560000	-0.4600620000
H	-1.4122740000	-2.3803450000	-0.1133670000
H	-2.4205220000	-1.5574630000	1.0991560000
H	-1.6938610000	-0.4062970000	-1.6806280000
H	-2.7142220000	0.4007940000	-0.4915850000
H	0.3427950000	2.4194050000	-0.3891740000
H	-0.3072250000	1.6371210000	-1.8517080000
H	-1.4091720000	2.3890230000	-0.6735660000
H	0.0018940000	1.3842780000	1.8818160000
H	-1.7552140000	1.2844750000	1.6513540000
H	-0.8273910000	-0.1739480000	2.0790780000
H	0.5321060000	-1.3689270000	0.2756180000
H	0.7116790000	-0.6606670000	-1.3289910000
H	2.2022780000	1.1284310000	-0.3682450000
H	1.9954310000	0.4401990000	1.2434140000
H	4.1404140000	-0.3420080000	0.2113650000
H	3.0479040000	-1.7244970000	0.4622880000
H	3.2492150000	-1.0357500000	-1.1642010000

3,4-Dimethylhexane

0 1

C	2.1783260000	-1.5761100000	0.5612140000
C	1.8825580000	-0.4196790000	-0.3990900000
C	0.8965430000	0.6474600000	0.1236370000
C	0.8635450000	1.8411030000	-0.8444430000
C	-0.5163460000	0.0887520000	0.4641120000
C	-1.3162620000	1.0803940000	1.3249770000
C	-1.3149900000	-0.3637220000	-0.7766820000
C	-2.5448070000	-1.2216530000	-0.4593700000
H	2.9715380000	-2.2270910000	0.1590000000
H	2.5206710000	-1.2043410000	1.5425580000
H	1.2961170000	-2.2120600000	0.7382620000
H	2.8325880000	0.0924710000	-0.6353170000
H	1.5214780000	-0.8233100000	-1.3627180000
H	1.3132050000	1.0146600000	1.0820810000
H	0.1923360000	2.6418680000	-0.4970090000
H	1.8691800000	2.2792900000	-0.9528530000
H	0.5300330000	1.5377430000	-1.8508950000
H	-0.3540840000	-0.8091850000	1.0882120000
H	-2.2549460000	0.6354130000	1.6899650000
H	-0.7373260000	1.3939820000	2.2094190000
H	-1.5836510000	1.9902770000	0.7615380000
H	-1.6319550000	0.5235950000	-1.3540970000
H	-0.6537440000	-0.9399850000	-1.4465030000
H	-3.0424780000	-1.5564820000	-1.3840220000
H	-2.2664450000	-2.1235510000	0.1127580000
H	-3.2939240000	-0.6725700000	0.1334920000

3-Ethyl-2-methylpentane

0 1

C	2.2745300000	0.4017930000	-1.1260500000
C	1.3928160000	-0.4712040000	-0.2211540000
C	1.9745940000	-0.5397310000	1.1993730000
C	-0.1087010000	-0.0582260000	-0.2604200000
C	-1.0157260000	-1.1591180000	0.3554790000
C	-2.2657170000	-1.4907690000	-0.4690610000
C	-0.3613310000	1.3321710000	0.3607430000
C	-1.7688350000	1.8988250000	0.1455410000
H	1.8772440000	0.4473970000	-2.1538350000
H	2.3564620000	1.4361530000	-0.7524070000
H	3.2983970000	-0.0037250000	-1.1814020000
H	1.4270530000	-1.4956600000	-0.6384380000
H	1.3641330000	-1.1703820000	1.8655490000
H	2.9921560000	-0.9638380000	1.1829180000
H	2.0457360000	0.4607910000	1.6573450000
H	-0.3682280000	0.0145770000	-1.3348160000
H	-0.4306290000	-2.0882870000	0.4721750000
H	-1.3157270000	-0.8697700000	1.3791120000
H	-2.8664720000	-2.2777420000	0.0164090000
H	-2.9163280000	-0.6131130000	-0.6074120000
H	-1.9903560000	-1.8564540000	-1.4732380000
H	-0.1586870000	1.2872380000	1.4459180000
H	0.3668600000	2.0498930000	-0.0539830000
H	-1.8482270000	2.9167020000	0.5611460000
H	-2.0205900000	1.9575450000	-0.9272480000
H	-2.5425770000	1.2862270000	0.6355020000

3-Ethyl-3-methylpentane

0 1

C	2.5760120000	-0.0185170000	-0.7390060000
C	1.7114670000	-0.0748680000	0.5261290000
C	0.1645610000	-0.0053700000	0.3484090000
C	-0.4465100000	-0.1906610000	1.7510370000
C	-0.1909220000	1.4042840000	-0.2027470000
C	-1.6610080000	1.7276720000	-0.4944660000
C	-0.2791240000	-1.1518960000	-0.6110150000
C	-1.7196720000	-1.6722800000	-0.5041150000
H	3.6434410000	-0.0834040000	-0.4719730000
H	2.3615570000	-0.8518830000	-1.4267910000
H	2.4398840000	0.9200250000	-1.2996060000
H	1.9449380000	-1.0096840000	1.0677550000
H	2.0152820000	0.7463070000	1.2007100000
H	-1.5425230000	-0.0975270000	1.7533490000
H	-0.1984770000	-1.1833100000	2.1629820000
H	-0.0478560000	0.5652410000	2.4482210000
H	0.1928810000	2.1459150000	0.5223680000
H	0.3814370000	1.5723370000	-1.1311060000
H	-1.7638290000	2.7881370000	-0.7780870000
H	-2.0589000000	1.1318760000	-1.3306550000
H	-2.3127410000	1.5605980000	0.3781580000
H	-0.0901150000	-0.8334560000	-1.6514440000
H	0.3925700000	-2.0131690000	-0.4411300000
H	-1.8929320000	-2.4537110000	-1.2625240000
H	-1.9200440000	-2.1267120000	0.4790490000
H	-2.4733990000	-0.8877650000	-0.6646360000

3-Ethylhexane

0 1

C	-3.0126580000	-0.9834530000	0.3395810000
C	-1.6264090000	-1.1645130000	-0.2866110000
C	-0.5672570000	-0.1130920000	0.1115960000
C	0.8252030000	-0.5143380000	-0.4419910000
C	1.9956800000	-0.3276180000	0.5337060000
C	3.3449400000	-0.7325100000	-0.0644810000
C	-0.9946500000	1.3041700000	-0.3231180000
C	-0.0645470000	2.4440060000	0.1028150000
H	-3.6694190000	-1.8322210000	0.0884610000
H	-2.9486520000	-0.9282400000	1.4402830000
H	-3.5168230000	-0.0685030000	-0.0095670000
H	-1.2408000000	-2.1597990000	-0.0023690000
H	-1.7169590000	-1.1815710000	-1.3897040000
H	-0.5060260000	-0.1197670000	1.2186210000
H	0.8033420000	-1.5781290000	-0.7410710000
H	1.0310900000	0.0459050000	-1.3738620000
H	2.0437010000	0.7216240000	0.8717730000
H	1.8011400000	-0.9276370000	1.4420630000
H	4.1644200000	-0.6068510000	0.6616840000
H	3.3408710000	-1.7890010000	-0.3836830000
H	3.5896070000	-0.1230330000	-0.9514850000
H	-1.1021880000	1.3128510000	-1.4250420000
H	-2.0001030000	1.5123660000	0.0795950000
H	-0.4797560000	3.4195920000	-0.1987430000
H	0.0700270000	2.4660860000	1.1978360000
H	0.9347190000	2.3604160000	-0.3537750000

3-Methylheptane

0 1

C	-2.8176430000	-1.5674970000	-0.2373130000
C	-2.5627400000	-0.1292560000	0.2236950000
C	-1.2373430000	0.4957530000	-0.2590690000
C	-1.2357590000	2.0052560000	0.0230860000
C	-0.0116500000	-0.2058880000	0.3588050000
C	1.3461110000	0.2033010000	-0.2255990000
C	2.5207660000	-0.5894430000	0.3591200000
C	3.8765770000	-0.1822650000	-0.2223790000
H	-3.8083120000	-1.9179110000	0.0951550000
H	-2.7916710000	-1.6437440000	-1.3381160000
H	-2.0725480000	-2.2747840000	0.1610480000
H	-3.3937490000	0.5061240000	-0.1314770000
H	-2.5989810000	-0.0811090000	1.3287980000
H	-1.1865710000	0.3552820000	-1.3576710000
H	-0.3329450000	2.5009750000	-0.3657320000
H	-2.1071030000	2.4963330000	-0.4406010000
H	-1.2831300000	2.2015140000	1.1090680000
H	-0.0112390000	-0.0226170000	1.4511950000
H	-0.1202730000	-1.2977080000	0.2367130000
H	1.3265070000	0.0654980000	-1.3237500000
H	1.5260300000	1.2803490000	-0.0586410000
H	2.5357800000	-0.4593560000	1.4572070000
H	2.3539520000	-1.6688430000	0.1858740000
H	4.6991690000	-0.7694120000	0.2171010000
H	3.9053350000	-0.3341790000	-1.3150620000
H	4.0898320000	0.8838190000	-0.0331830000

4-Methylheptane

0 1

C	-3.5564750000	-0.9278340000	0.1263280000
C	-2.1091840000	-0.7581640000	-0.3434960000
C	-1.4470940000	0.5079710000	0.2139560000
C	-0.0103770000	0.7863470000	-0.2740340000
C	0.3975830000	2.2218410000	0.0892860000
C	0.9939090000	-0.2501300000	0.2681820000
C	2.3975430000	-0.1929380000	-0.3483360000
C	3.3239090000	-1.2917460000	0.1793220000
H	-4.0136320000	-1.8394960000	-0.2913120000
H	-3.6143110000	-0.9998440000	1.2260090000
H	-4.1804030000	-0.0706940000	-0.1799060000
H	-1.5298240000	-1.6520850000	-0.0540600000
H	-2.0825630000	-0.7232070000	-1.4486010000
H	-2.0799970000	1.3741910000	-0.0541880000
H	-1.4498690000	0.4671010000	1.3207910000
H	-0.0137100000	0.7046990000	-1.3795020000
H	1.3979230000	2.4798390000	-0.2910520000
H	-0.3132290000	2.9535090000	-0.3286940000
H	0.4103340000	2.3602770000	1.1850850000
H	1.0711580000	-0.1297590000	1.3667390000
H	0.5918220000	-1.2654040000	0.1044810000
H	2.3133400000	-0.2790780000	-1.4477670000
H	2.8587960000	0.7911120000	-0.1552940000
H	4.3241560000	-1.2333430000	-0.2796460000
H	3.4538550000	-1.2143700000	1.2724770000
H	2.9172800000	-2.2955250000	-0.0328150000

2,2,3,3-Tetramethylbutane

0 1

C	1.3587140000	-0.5827520000	1.3155910000
C	0.7963860000	-0.0000410000	-0.0002760000
C	1.3587390000	1.4307750000	-0.1539690000
C	1.3577030000	-0.8486140000	-1.1627600000
C	-0.7962930000	0.0000160000	0.0003360000
C	-1.3591680000	0.3079790000	-1.4050400000
C	-1.3583260000	-1.3713130000	0.4365160000
C	-1.3576070000	1.0639240000	0.9697080000
H	1.1513150000	-1.6590290000	1.4151890000
H	2.4539570000	-0.4585340000	1.3417860000
H	0.9523620000	-0.0759390000	2.2049980000
H	0.9521260000	1.9477910000	-1.0374350000
H	1.1522030000	2.0553370000	0.7283770000
H	2.4538850000	1.3909460000	-0.2755470000
H	1.1483500000	-0.3976960000	-2.1446170000

H	2.4530910000	-0.9323020000	-1.0695150000
H	0.9520790000	-1.8725600000	-1.1668230000
H	-2.4543180000	0.4242560000	-1.3520050000
H	-0.9527830000	1.2421130000	-1.8236790000
H	-1.1529150000	-0.5017000000	-2.1213120000
H	-2.4535120000	-1.3836960000	0.3097850000
H	-0.9517710000	-2.2004440000	-0.1638730000
H	-1.1514880000	-1.5872630000	1.4956510000
H	-2.4529190000	0.9616920000	1.0435450000
H	-0.9515610000	0.9585210000	1.9881610000
H	-1.1489930000	2.0886720000	0.6266830000

2,2,3-Trimethylpentane

0 1

C	-1.0225130000	-0.1648000000	-0.0292000000
C	0.4466150000	0.3231210000	-0.3013160000
C	1.5226020000	-0.5400290000	0.3990850000
C	-1.2438100000	-1.5386080000	-0.6962220000
C	-1.3167600000	-0.2867300000	1.4792280000
C	-2.0351690000	0.8172160000	-0.6540540000
C	0.6714700000	1.8092950000	0.0289960000
C	2.9103350000	-0.4538910000	-0.2463920000
H	0.5983830000	0.2059330000	-1.3924920000
H	1.2141120000	-1.5977720000	0.4045080000
H	1.5947770000	-0.2409620000	1.4604850000
H	-1.0802040000	-1.4807500000	-1.7854910000
H	-0.5730640000	-2.3147780000	-0.2968000000
H	-2.2774640000	-1.8855640000	-0.5323890000
H	-1.1553130000	0.6664590000	2.0082770000
H	-2.3680740000	-0.5772350000	1.6423560000
H	-0.6866390000	-1.0506640000	1.9611750000
H	-2.0387770000	1.7928670000	-0.1447180000
H	-3.0568220000	0.4081940000	-0.5860470000
H	-1.8178780000	0.9930900000	-1.7214620000
H	0.4342700000	2.0303900000	1.0835400000
H	0.0636020000	2.4763660000	-0.5993040000
H	1.7239380000	2.0900840000	-0.1318020000
H	3.3082380000	0.5739260000	-0.2484710000
H	2.8831170000	-0.7993330000	-1.2940980000
H	3.6371800000	-1.0836950000	0.2919830000

2,2,4-Trimethylpentane

0 1

C	1.1900270000	-0.0554640000	-0.0067240000
C	-0.2070350000	-0.6413480000	-0.3697070000
C	-1.4956130000	-0.0887570000	0.2803290000
C	2.2458680000	-1.0782230000	-0.4775690000
C	1.3383220000	0.1403290000	1.5134300000
C	1.4731020000	1.2757430000	-0.7320190000
C	-1.8303040000	1.3669010000	-0.0777610000
C	-2.6757970000	-0.9985730000	-0.0996320000
H	-0.1734970000	-1.7177820000	-0.1211720000
H	-0.3231020000	-0.5978110000	-1.4699190000
H	-1.3791110000	-0.1489120000	1.3780800000
H	2.1367060000	-2.0393010000	0.0520960000
H	3.2677720000	-0.7061830000	-0.2949480000
H	2.1508010000	-1.2786740000	-1.5581980000
H	2.3670510000	0.4473130000	1.7663970000
H	0.6594250000	0.9178770000	1.8984800000
H	1.1257360000	-0.7935370000	2.0610790000
H	0.7972820000	2.0820810000	-0.4147110000
H	2.5042310000	1.6118090000	-0.5299580000
H	1.3690760000	1.1609390000	-1.8241080000
H	-1.8522610000	1.5127930000	-1.1720820000
H	-2.8240280000	1.6422050000	0.3133450000
H	-1.1078260000	2.0825720000	0.3408850000
H	-2.8528590000	-0.9808540000	-1.1897010000
H	-3.6063660000	-0.6720260000	0.3929040000
H	-2.4904550000	-2.0461590000	0.1894540000

n-Octane

0 1

C	-4.4972400000	-0.2557210000	-0.0000010000
C	-3.1901900000	0.5404760000	-0.0000040000
C	-1.9394190000	-0.3448890000	0.0000010000
C	-0.6252660000	0.4427290000	-0.0000020000
C	0.6252660000	-0.4427290000	0.0000020000
C	1.9394190000	0.3448890000	-0.0000010000
C	3.1901900000	-0.5404760000	0.0000040000
C	4.4972400000	0.2557210000	0.0000010000
H	-5.3767470000	0.4083400000	-0.0000040000
H	-4.5699300000	-0.9055510000	-0.8889540000
H	-4.5699310000	-0.9055420000	0.8889590000
H	-3.1636860000	1.2068360000	-0.8821250000
H	-3.1636860000	1.2068440000	0.8821110000
H	-1.9656760000	-1.0122870000	0.8825050000
H	-1.9656750000	-1.0122950000	-0.8824970000
H	-0.5999380000	1.1099880000	-0.8823830000
H	-0.5999380000	1.1099960000	0.8823730000
H	0.5999380000	-1.1099880000	0.8823830000
H	0.5999380000	-1.1099960000	-0.8823730000
H	1.9656760000	1.0122870000	-0.8825050000
H	1.9656750000	1.0122950000	0.8824970000
H	3.1636850000	-1.2068360000	0.8821250000
H	3.1636860000	-1.2068440000	-0.8821110000
H	5.3767470000	-0.4083410000	0.0000040000
H	4.5699310000	0.9055420000	-0.8889590000
H	4.5699300000	0.9055510000	0.8889540000

2,2,3,3-Tetramethylpentane

0 1

C	0.4076420000	-0.3328950000	0.0362010000
C	-1.1055950000	0.1775750000	0.0015800000
C	1.3788490000	0.8833030000	0.1362280000
C	0.7331500000	-1.1501590000	-1.2337420000
C	0.6449640000	-1.2498300000	1.2571780000
C	-2.0972070000	-0.9863810000	0.2262180000
C	-1.4524110000	0.8236340000	-1.3589620000
C	-1.3784380000	1.2290290000	1.1010550000
C	2.8691660000	0.6000040000	-0.0961400000
H	1.2680160000	1.3381870000	1.1342490000
H	1.0669490000	1.6573590000	-0.5851340000
H	1.7037030000	-1.6588310000	-1.1286580000
H	-0.0187370000	-1.9329220000	-1.4179340000
H	0.7895230000	-0.5196290000	-2.1344770000
H	0.3349400000	-0.7744580000	2.2011930000
H	1.7135760000	-1.4977230000	1.3521820000
H	0.1041060000	-2.2037940000	1.1661420000
H	-3.1290110000	-0.6322840000	0.0670130000
H	-1.9299610000	-1.8227650000	-0.4705960000
H	-2.0447470000	-1.3843620000	1.2507960000
H	-0.7506540000	1.6260220000	-1.6354220000
H	-2.4580050000	1.2733200000	-1.3129240000
H	-1.4627510000	0.0876600000	-2.1770440000
H	-0.8515810000	2.1765860000	0.9141630000
H	-1.0887460000	0.8722500000	2.1020120000
H	-2.4561720000	1.4586960000	1.1369290000
H	3.0765780000	0.2675590000	-1.1253120000
H	3.2643470000	-0.1684720000	0.5880690000
H	3.4574060000	1.5165180000	0.0739390000

2,2,3,4-Tetramethylpentane

0 1

C	-1.2166000000	0.0956000000	0.0930000000
C	0.1906000000	-0.5963000000	0.2964000000
C	1.4062000000	0.3907000000	0.3695000000
C	-1.4667000000	1.1278000000	1.2179000000
C	-2.3540000000	-0.9477000000	0.1865000000
C	-1.3479000000	0.8174000000	-1.2579000000
C	0.4540000000	-1.7445000000	-0.6953000000
C	1.6344000000	1.2349000000	-0.8912000000
C	2.7001000000	-0.3779000000	0.6811000000
H	0.1429000000	-1.0772000000	1.2859000000
H	1.2516000000	1.0748000000	1.2122000000
H	-0.7765000000	1.9751000000	1.1583000000
H	-2.4802000000	1.5417000000	1.1558000000
H	-1.3564000000	0.6681000000	2.2064000000
H	-2.2578000000	-1.5571000000	1.0918000000
H	-2.3644000000	-1.6182000000	-0.6788000000
H	-3.3374000000	-0.4636000000	0.2201000000
H	-0.9009000000	1.8114000000	-1.2634000000
H	-2.4066000000	0.9891000000	-1.4935000000
H	-0.9410000000	0.2327000000	-2.0870000000
H	0.5852000000	-1.3898000000	-1.7205000000
H	1.3458000000	-2.3166000000	-0.4225000000
H	-0.3635000000	-2.4706000000	-0.6960000000
H	2.6691000000	1.5975000000	-0.9284000000
H	1.0215000000	2.1376000000	-0.8784000000
H	1.4590000000	0.6800000000	-1.8155000000
H	3.4955000000	0.3218000000	0.9630000000
H	3.0639000000	-0.9450000000	-0.1817000000
H	2.5605000000	-1.0655000000	1.5217000000

2,2,3-Trimethylhexane

0 1

C	2.650917000	0.526510000	-0.534324000
C	1.439911000	-0.277382000	-0.016812000
C	1.438996000	-1.631730000	-0.756944000
C	1.621632000	-0.530365000	1.493206000
C	0.105761000	0.493632000	-0.325258000
C	-1.152582000	-0.190323000	0.258149000
C	-2.461013000	0.169559000	-0.459367000
C	-3.686497000	-0.509373000	0.157611000
C	0.140631000	1.973665000	0.095823000
H	2.810194000	1.454441000	0.035558000
H	3.572571000	-0.072598000	-0.449581000
H	2.527601000	0.798493000	-1.596554000
H	0.619003000	-2.290419000	-0.432149000
H	1.343232000	-1.488076000	-1.846543000
H	2.382411000	-2.172622000	-0.574428000
H	0.834034000	-1.184587000	1.899021000
H	2.589461000	-1.022487000	1.686201000
H	1.610937000	0.408368000	2.070345000
H	0.004117000	0.472376000	-1.427995000
H	-1.042126000	-1.286798000	0.222506000
H	-1.246394000	0.068103000	1.329384000
H	-2.611666000	1.263394000	-0.452037000
H	-2.378169000	-0.117440000	-1.524043000
H	-4.610190000	-0.246198000	-0.382870000
H	-3.589323000	-1.608490000	0.136369000
H	-3.820846000	-0.211900000	1.211748000
H	-0.827228000	2.459323000	-0.103712000
H	0.341542000	2.084257000	1.175028000
H	0.904295000	2.547704000	-0.448756000

2,2,4,4-Tetramethylpentane

0 1

C	2.433478000	-0.312712000	1.155805000
C	1.386204000	-0.003482000	0.062050000
C	1.734542000	1.377390000	-0.532071000
C	1.515441000	-1.076007000	-1.035730000
C	0.000000000	0.000001000	0.781783000
C	-1.386205000	0.003482000	0.062050000
C	-2.433479000	0.312715000	1.155804000
C	-1.515439000	1.076004000	-1.035732000
C	-1.734543000	-1.377392000	-0.532069000
H	2.281149000	-1.317404000	1.584081000
H	3.455835000	-0.272739000	0.744778000
H	2.375386000	0.416081000	1.981572000
H	1.081966000	1.655085000	-1.371036000
H	1.656819000	2.169364000	0.231719000
H	2.771761000	1.380768000	-0.907627000
H	0.875462000	-0.861698000	-1.905153000
H	2.555664000	-1.120762000	-1.400602000
H	1.253281000	-2.077684000	-0.658007000
H	-0.015454000	-0.877146000	1.454608000
H	0.015453000	0.877151000	1.454603000
H	-3.455836000	0.272743000	0.744776000
H	-2.375389000	-0.416078000	1.981571000
H	-2.281149000	1.317407000	1.584079000
H	-2.555661000	1.120759000	-1.400608000
H	-1.253279000	2.077682000	-0.658011000
H	-0.875457000	0.861693000	-1.905154000
H	-2.771760000	-1.380769000	-0.907628000
H	-1.081963000	-1.655087000	-1.371030000
H	-1.656823000	-2.169364000	0.231724000

2,2,4-Trimethylhexane

0 1

C	2.532563000	-1.599918000	0.123275000
C	2.431625000	-0.121511000	-0.260770000
C	1.109297000	0.567337000	0.140027000
C	1.273359000	2.092706000	0.043999000
C	-0.084963000	0.064869000	-0.713851000
C	-1.453362000	-0.163835000	-0.012047000
C	-2.479462000	-0.545778000	-1.096921000
C	-1.354326000	-1.323469000	0.998104000
C	-1.951877000	1.100183000	0.712923000
H	3.498055000	-2.027344000	-0.192657000
H	2.450935000	-1.733725000	1.215543000
H	1.738319000	-2.206247000	-0.342410000
H	3.268337000	0.423832000	0.211586000
H	2.576666000	-0.007453000	-1.352067000
H	0.922843000	0.313231000	1.200222000
H	0.348801000	2.627700000	0.306750000
H	2.071320000	2.452468000	0.714824000
H	1.546086000	2.388837000	-0.984370000
H	-0.237699000	0.774636000	-1.547709000
H	0.186041000	-0.892218000	-1.192449000
H	-3.468010000	-0.751833000	-0.653592000
H	-2.604093000	0.266654000	-1.832336000
H	-2.163667000	-1.448940000	-1.645705000
H	-2.332249000	-1.514504000	1.470819000
H	-1.034107000	-2.255800000	0.503399000
H	-0.635760000	-1.108811000	1.805506000
H	-2.948426000	0.924830000	1.152134000
H	-1.280367000	1.397565000	1.534044000
H	-2.040142000	1.953607000	0.020039000

2,2,5-Trimethylhexane

0 1

C	2.764634000	1.005387000	-0.625208000
C	1.780869000	-0.004365000	-0.002799000
C	2.103799000	-1.408162000	-0.549951000
C	1.962539000	0.005515000	1.527141000
C	0.342560000	0.431274000	-0.392988000
C	-0.816367000	-0.420677000	0.141151000
C	-2.210855000	0.002357000	-0.365045000
C	-3.269393000	-1.024384000	0.060170000
C	-2.611045000	1.408557000	0.103666000
H	2.576492000	2.026751000	-0.253807000
H	3.808368000	0.746260000	-0.380861000
H	2.672512000	1.026806000	-1.724111000
H	1.464500000	-2.184756000	-0.100988000
H	1.968626000	-1.450961000	-1.644102000
H	3.150403000	-1.679897000	-0.332467000
H	1.325010000	-0.743633000	2.022932000
H	3.007501000	-0.220946000	1.797455000
H	1.714358000	0.993171000	1.951702000
H	0.209825000	1.476384000	-0.061857000
H	0.281513000	0.454771000	-1.497668000
H	-0.657649000	-1.475384000	-0.143333000
H	-0.826090000	-0.400498000	1.246649000
H	-2.175020000	0.011073000	-1.472175000
H	-4.266221000	-0.759516000	-0.329236000
H	-3.022524000	-2.034795000	-0.305566000
H	-3.346383000	-1.078465000	1.160591000
H	-3.618593000	1.672783000	-0.257753000
H	-2.628884000	1.464188000	1.206906000
H	-1.918195000	2.183656000	-0.259135000

3-Ethyl-2,2-dimethylpentane

0 1

C	1.830392000	1.219965000	-0.409890000
C	1.196360000	-0.137318000	-0.041283000
C	1.739962000	-1.176201000	-1.045662000
C	1.654979000	-0.530870000	1.377194000
C	-0.371222000	-0.060504000	-0.145544000
C	-1.074079000	-1.372921000	0.274291000
C	-0.998857000	1.148034000	0.607822000
C	-1.394411000	2.330879000	-0.283745000
C	-2.501490000	-1.502385000	-0.268692000
H	1.549820000	2.020547000	0.291343000
H	2.930346000	1.143105000	-0.393351000
H	1.536772000	1.538055000	-1.424104000
H	1.401679000	-2.199417000	-0.822311000
H	1.427406000	-0.934631000	-2.075575000
H	2.842396000	-1.187870000	-1.027026000
H	1.304172000	-1.536545000	1.657322000
H	2.756127000	-0.538391000	1.436125000
H	1.291559000	0.176864000	2.139999000
H	-0.585554000	0.079876000	-1.222973000
H	-0.495334000	-2.244084000	-0.071357000
H	-1.094411000	-1.439661000	1.377013000
H	-1.901268000	0.809431000	1.144100000
H	-0.315355000	1.498950000	1.399439000
H	-1.840216000	3.146358000	0.309900000
H	-0.530520000	2.748040000	-0.824650000
H	-2.138313000	2.025022000	-1.039114000
H	-2.974871000	-2.436718000	0.074291000
H	-3.147355000	-0.669646000	0.056145000
H	-2.506885000	-1.511355000	-1.372162000

2,2-Dimethylheptane

0 1

C	3.062556000	1.173249000	-0.000008000
C	2.037685000	0.022708000	-0.000008000
C	0.618381000	0.649872000	0.000012000
C	-0.580264000	-0.307241000	0.000003000
C	-1.925948000	0.427997000	0.000016000
C	-3.139849000	-0.507083000	0.000005000
C	-4.477919000	0.235826000	0.000016000
C	2.259843000	-0.837841000	-1.258443000
C	2.259870000	-0.837864000	1.258408000
H	4.095132000	0.786082000	-0.000025000
H	2.943810000	1.813145000	-0.890446000
H	2.943833000	1.813125000	0.890447000
H	0.538033000	1.313507000	0.881556000
H	0.538022000	1.313537000	-0.881508000
H	-0.536749000	-0.970271000	-0.882561000
H	-0.536745000	-0.970294000	0.882550000
H	-1.979311000	1.093944000	0.882406000
H	-1.979314000	1.093969000	-0.882356000
H	-3.086875000	-1.171884000	-0.882127000
H	-3.086873000	-1.171907000	0.882118000
H	-5.329852000	-0.463255000	0.000009000
H	-4.576951000	0.882141000	0.888986000
H	-4.576954000	0.882164000	-0.888935000
H	1.587876000	-1.710386000	-1.287116000
H	2.090204000	-0.251212000	-2.177363000
H	3.294199000	-1.219226000	-1.292725000
H	1.587905000	-1.710411000	1.287078000
H	3.294227000	-1.219248000	1.292661000
H	2.090249000	-0.251253000	2.177342000

2,3,3,4-Tetramethylpentane

0 1

C	0.0015660000	-0.4482840000	-0.0076500000
C	-1.2762180000	0.4481000000	-0.2313740000
C	1.2650130000	0.4626080000	0.2378450000
C	-0.1971180000	-1.3702380000	1.2154640000
C	0.2146190000	-1.3372380000	-1.2525800000
C	-1.5745530000	1.4239590000	0.9199320000
C	-2.5572740000	-0.3457350000	-0.5491520000
C	2.5585600000	-0.3182290000	0.5366590000
C	1.5476130000	1.4705210000	-0.8895670000
H	-1.0669630000	1.0568450000	-1.1282550000
H	1.0462970000	1.0461800000	1.1490870000
H	-1.1473060000	-1.9218050000	1.1507030000
H	-0.1994670000	-0.8071480000	2.1627970000
H	0.6018530000	-2.1236830000	1.2828050000
H	0.2078570000	-0.7515220000	-2.1860730000
H	-0.5723140000	-2.1014190000	-1.3382080000
H	1.1734490000	-1.8750640000	-1.2009410000
H	-0.7478670000	2.1223790000	1.1171520000
H	-2.4617310000	2.0321560000	0.6785350000
H	-1.7945240000	0.8907610000	1.8599050000
H	-2.4412250000	-1.0143220000	-1.4146450000
H	-2.8918590000	-0.9550800000	0.3066160000
H	-3.3773890000	0.3518870000	-0.7856630000
H	3.3675530000	0.3864160000	0.7900510000
H	2.4532980000	-1.0092170000	1.3857810000
H	2.9026010000	-0.9014090000	-0.3334700000
H	1.7758390000	0.9636710000	-1.8420650000
H	0.7099140000	2.1602840000	-1.0700700000
H	2.4250760000	2.0867260000	-0.6334200000

2,3,3-Trimethylhexane

0 1

C	3.453937000	0.323850000	-0.215569000
C	2.069411000	0.222035000	0.432283000
C	1.054488000	-0.469931000	-0.492319000
C	-0.429831000	-0.618243000	-0.025816000
C	-0.986194000	-1.866960000	-0.747158000
C	-0.506750000	-0.865686000	1.493575000
C	-1.290446000	0.624124000	-0.463492000
C	-2.748227000	0.575543000	0.028196000
C	-0.699752000	1.995430000	-0.095675000
H	4.183467000	0.795494000	0.462548000
H	3.844961000	-0.672259000	-0.485739000
H	3.421507000	0.924717000	-1.140780000
H	2.157155000	-0.343850000	1.375336000
H	1.728590000	1.230159000	0.715008000
H	1.060405000	0.033195000	-1.477748000
H	1.446584000	-1.485051000	-0.686351000
H	-2.046169000	-2.049401000	-0.514151000
H	-0.895176000	-1.764787000	-1.841878000
H	-0.425605000	-2.768146000	-0.449237000
H	-1.526994000	-1.142307000	1.801747000
H	0.156063000	-1.696913000	1.785956000
H	-0.207855000	0.015884000	2.081839000
H	-1.319353000	0.578492000	-1.569280000
H	-3.332043000	1.382896000	-0.443429000
H	-3.252378000	-0.372603000	-0.210488000
H	-2.812954000	0.725447000	1.118874000
H	-1.378117000	2.797165000	-0.431524000
H	-0.577338000	2.112473000	0.994324000
H	0.275425000	2.178430000	-0.569177000

2,3,4-Trimethylhexane

0 1

C	2.569307000	-0.902392000	-0.956540000
C	1.395891000	-0.707866000	0.015704000
C	1.908513000	-0.600993000	1.461121000
C	0.474291000	0.475773000	-0.413339000
C	-0.968043000	0.331114000	0.161898000
C	1.120823000	1.837446000	-0.104235000
C	-1.778342000	1.636029000	0.096200000
C	-1.740444000	-0.800809000	-0.554298000
C	-2.974748000	-1.314083000	0.194348000
H	2.214896000	-1.061078000	-1.988473000
H	3.244058000	-0.029602000	-0.966244000
H	3.176039000	-1.778270000	-0.672546000
H	0.787990000	-1.627633000	-0.040508000
H	1.090660000	-0.444011000	2.182932000
H	2.430927000	-1.526777000	1.753322000
H	2.624111000	0.229040000	1.581478000
H	0.375704000	0.410389000	-1.514705000
H	-0.876708000	0.053052000	1.230033000
H	0.597663000	2.660759000	-0.611951000
H	1.118341000	2.060169000	0.975584000
H	2.168138000	1.865727000	-0.443950000
H	-2.810319000	1.477496000	0.445691000
H	-1.346279000	2.431613000	0.720546000
H	-1.836301000	2.014074000	-0.939879000
H	-2.038132000	-0.439120000	-1.556752000
H	-1.068555000	-1.656159000	-0.734099000
H	-3.470092000	-2.122918000	-0.367199000
H	-2.699934000	-1.717902000	1.184009000
H	-3.725695000	-0.524160000	0.357559000

2,3,5-Trimethylhexane

C	-3.185406000	0.593548000	-0.410209000
C	-1.833222000	-0.132054000	-0.333426000
C	-0.784911000	0.726496000	0.422826000
C	0.631378000	0.796294000	-0.192598000
C	-2.023904000	-1.523983000	0.288673000
C	1.454537000	1.873533000	0.532982000
C	1.360951000	-0.573956000	-0.285844000
C	2.577461000	-0.512435000	-1.222856000
C	1.763146000	-1.158661000	1.076917000
H	-3.934792000	-0.006403000	-0.953166000
H	-3.585211000	0.788860000	0.600682000
H	-3.093557000	1.564238000	-0.924879000
H	-1.475197000	-0.268765000	-1.371907000
H	-1.155727000	1.765117000	0.474759000
H	-0.720672000	0.391400000	1.474539000
H	0.498310000	1.137392000	-1.238061000
H	-1.081066000	-2.088807000	0.348923000
H	-2.424557000	-1.438445000	1.314354000
H	-2.737822000	-2.127770000	-0.296249000
H	2.471018000	1.970013000	0.120920000
H	0.968371000	2.858576000	0.442170000
H	1.549236000	1.656322000	1.610125000
H	0.646718000	-1.276854000	-0.750350000
H	3.013838000	-1.515060000	-1.364553000
H	2.300967000	-0.124580000	-2.217439000
H	3.374858000	0.134968000	-0.821084000
H	2.174138000	-2.174599000	0.956188000
H	2.540885000	-0.548776000	1.565999000
H	0.910081000	-1.229514000	1.770242000

2,3-Dimethylheptane

0 1

C	3.261518000	-0.379447000	-0.959324000
C	2.084295000	0.439969000	-0.410093000
C	0.810682000	-0.429058000	-0.197759000
C	-0.446676000	0.446644000	-0.017276000
C	-1.783920000	-0.297003000	-0.123945000
C	-3.000507000	0.632654000	-0.044495000
C	-4.336729000	-0.105965000	-0.149179000
C	2.516010000	1.232301000	0.833684000
H	4.107820000	0.277090000	-1.220693000
H	3.634349000	-1.110391000	-0.222166000
H	2.976857000	-0.936194000	-1.867702000
H	-0.402636000	0.964427000	0.958876000
H	-0.430173000	1.244000000	-0.783530000
H	-1.816382000	-0.856601000	-1.078510000
H	-1.863647000	-1.055447000	0.675338000
H	-2.964432000	1.197623000	0.905491000
H	-2.932823000	1.389539000	-0.847832000
H	-5.189904000	0.589012000	-0.088914000
H	-4.418484000	-0.652044000	-1.104646000
H	-4.450830000	-0.844954000	0.662431000
H	1.702677000	1.860071000	1.230600000
H	2.852235000	0.564959000	1.644925000
H	3.359107000	1.900380000	0.592011000
C	0.957235000	-1.459848000	0.932969000
H	1.816642000	1.177241000	-1.190841000
H	0.672449000	-0.992042000	-1.141718000
H	0.105749000	-2.157495000	0.949396000
H	1.868480000	-2.067409000	0.817952000
H	1.001497000	-0.973253000	1.922038000

2,4,4-Trimethylhexane

0 1

C	-2.626391000	-1.481222000	0.066935000
C	-2.145335000	-0.178087000	-0.580605000
C	-0.834086000	0.463188000	-0.034721000
C	-0.726340000	1.851680000	-0.698861000
C	-0.928709000	0.639517000	1.492458000
C	0.361728000	-0.452561000	-0.433621000
C	1.730850000	-0.324879000	0.274388000
C	2.628719000	-1.494165000	-0.163286000
C	2.463716000	1.003955000	0.038590000
H	-3.540290000	-1.839612000	-0.434246000
H	-1.881302000	-2.290262000	-0.004161000
H	-2.871297000	-1.347468000	1.132824000
H	-2.016117000	-0.341901000	-1.666380000
H	-2.951085000	0.572448000	-0.486066000
H	0.168935000	2.404137000	-0.382390000
H	-0.693175000	1.761171000	-1.797692000
H	-1.603229000	2.470665000	-0.443997000
H	-0.089279000	1.235936000	1.883370000
H	-1.859387000	1.164503000	1.767189000
H	-0.922346000	-0.326644000	2.022066000
H	0.041613000	-1.497122000	-0.280725000
H	0.521025000	-0.351158000	-1.524417000
H	1.565882000	-0.431429000	1.362325000
H	3.598229000	-1.471923000	0.360861000
H	2.156153000	-2.468519000	0.043386000
H	2.835695000	-1.448580000	-1.247224000
H	3.479002000	0.966512000	0.467832000
H	2.567410000	1.215561000	-1.040148000
H	1.948652000	1.859134000	0.499937000

3-Ethyl-2,4-dimethylpentane

0 1

C	2.401033000	0.468439000	-1.098149000
C	1.567187000	-0.508652000	-0.255356000
C	2.056655000	-0.513732000	1.200939000
C	0.032713000	-0.284176000	-0.429921000
C	-0.754673000	-1.527110000	0.042978000
C	-2.198257000	-1.636994000	-0.461140000
C	-0.464089000	1.087140000	0.144156000
C	-1.456062000	1.786166000	-0.801772000
C	-1.038488000	1.030789000	1.570201000
H	2.098231000	0.446241000	-2.158187000
H	2.304902000	1.509100000	-0.745004000
H	3.471725000	0.209337000	-1.050330000
H	1.762915000	-1.518165000	-0.663631000
H	1.502167000	-1.233519000	1.823534000
H	3.122648000	-0.792124000	1.247164000
H	1.962212000	0.481006000	1.668367000
H	-0.115723000	-0.231023000	-1.525295000
H	-0.204820000	-2.419197000	-0.307088000
H	-0.748431000	-1.592088000	1.144407000
H	-2.641402000	-2.602566000	-0.166373000
H	-2.848115000	-0.844758000	-0.058586000
H	-2.243697000	-1.573578000	-1.561879000
H	0.420941000	1.745785000	0.192543000
H	-1.730320000	2.783363000	-0.417803000
H	-1.021947000	1.923274000	-1.805858000
H	-2.388371000	1.210589000	-0.919785000
H	-1.241047000	2.051353000	1.935901000
H	-1.991141000	0.477910000	1.612967000
H	-0.346840000	0.557834000	2.283324000

2,4-Dimethylheptane

0 1

C	4.071059000	-0.768731000	-0.209253000
C	2.856482000	0.149398000	-0.371572000
C	1.575756000	-0.446660000	0.224395000
C	0.285092000	0.365707000	-0.010330000
C	-0.949568000	-0.405623000	0.525582000
C	-2.201187000	-0.406575000	-0.376537000
C	-3.282965000	-1.320479000	0.215646000
C	-2.770884000	0.993450000	-0.642604000
C	0.382934000	1.775290000	0.595588000
H	4.979176000	-0.319387000	-0.642786000
H	4.276968000	-0.974659000	0.855217000
H	3.909169000	-1.740456000	-0.706480000
H	3.075962000	1.123809000	0.097999000
H	2.693388000	0.358540000	-1.445584000
H	1.430427000	-1.457472000	-0.200217000
H	1.713650000	-0.594957000	1.313590000
H	0.168129000	0.473009000	-1.106822000
H	-0.667659000	-1.460553000	0.689992000
H	-1.225424000	-0.014638000	1.523815000
H	-1.898462000	-0.834945000	-1.352518000
H	-4.164211000	-1.379672000	-0.444248000
H	-2.907309000	-2.345679000	0.368831000
H	-3.626018000	-0.942366000	1.194960000
H	-3.666317000	0.936197000	-1.283651000
H	-3.068500000	1.486211000	0.300023000
H	-2.045406000	1.648182000	-1.149881000
H	-0.559306000	2.331922000	0.481477000
H	0.603626000	1.717680000	1.676429000
H	1.177807000	2.374572000	0.124374000

2,5-Dimethylheptane

0 1

C	-3.970492000	-0.518420000	0.347683000
C	-2.592976000	-0.768828000	-0.273391000
C	-1.476696000	0.188679000	0.190965000
C	-0.111609000	-0.292156000	-0.343529000
C	1.111431000	0.423269000	0.243494000
C	2.473723000	-0.058104000	-0.295098000
C	3.599242000	0.864601000	0.192396000
C	2.778027000	-1.516062000	0.077680000
C	-1.772100000	1.642717000	-0.205379000
H	-4.693077000	-1.285835000	0.025905000
H	-3.922895000	-0.549274000	1.450028000
H	-4.386072000	0.460843000	0.061199000
H	-2.282284000	-1.803024000	-0.039690000
H	-2.670258000	-0.719290000	-1.376404000
H	-1.437054000	0.137619000	1.297788000
H	-0.030052000	-1.374290000	-0.142520000
H	-0.101789000	-0.187183000	-1.446110000
H	1.032935000	1.506306000	0.046214000
H	1.104897000	0.314331000	1.345844000
H	2.437112000	0.009437000	-1.400162000
H	4.576338000	0.558754000	-0.216462000
H	3.421736000	1.911073000	-0.106162000
H	3.678180000	0.841680000	1.293769000
H	3.761235000	-1.826892000	-0.312658000
H	2.800934000	-1.643125000	1.174895000
H	2.028950000	-2.215866000	-0.324486000
H	-0.996118000	2.333680000	0.158525000
H	-1.822248000	1.744960000	-1.304429000
H	-2.731776000	1.991918000	0.205983000

2,6-Dimethylheptane

0 1

C	3.755175000	-0.773611000	0.547889000
C	2.585670000	0.179758000	0.267689000
C	2.901432000	1.078296000	-0.936395000
C	1.274861000	-0.609829000	0.088796000
C	0.000000000	0.238230000	-0.000001000
C	-1.274861000	-0.609829000	-0.088798000
C	-2.585670000	0.179758000	-0.267689000
C	-3.755176000	-0.773611000	-0.547887000
C	-2.901430000	1.078296000	0.936395000
H	3.564812000	-1.394996000	1.438542000
H	3.921208000	-1.456012000	-0.304514000
H	4.693620000	-0.220393000	0.717380000
H	2.463066000	0.831993000	1.154720000
H	2.112941000	1.825314000	-1.118216000
H	3.845641000	1.626751000	-0.783549000
H	3.012011000	0.476695000	-1.856400000
H	1.359008000	-1.243042000	-0.816008000
H	1.171149000	-1.311340000	0.937452000
H	-0.047776000	0.902651000	0.881527000
H	0.047775000	0.902653000	-0.881527000
H	-1.171150000	-1.311338000	-0.937456000
H	-1.359008000	-1.243044000	0.816005000
H	-2.463068000	0.831993000	-1.154720000
H	-4.693622000	-0.220392000	-0.717376000
H	-3.564816000	-1.394995000	-1.438540000
H	-3.921208000	-1.456012000	0.304516000
H	-3.845639000	1.626752000	0.783551000
H	-3.012007000	0.476696000	1.856401000
H	-2.112938000	1.825314000	1.118215000

4-Ethyl-2-methylhexane

0 1

C	1.962227000	-1.126141000	0.401387000
C	3.328059000	-0.862546000	-0.239001000
C	0.822194000	-0.210500000	-0.101230000
C	-0.534511000	-0.855027000	0.268657000
C	-1.840405000	-0.214023000	-0.264492000
C	-2.892762000	-1.300439000	-0.539432000
C	-2.429195000	0.839805000	0.686288000
C	1.016851000	1.241692000	0.404815000
C	0.605952000	2.340941000	-0.581677000
H	1.684622000	-2.177936000	0.211282000
H	2.039687000	-1.029589000	1.501045000
H	4.092856000	-1.547065000	0.162508000
H	3.288927000	-1.011476000	-1.331754000
H	3.684187000	0.165135000	-0.059188000
H	0.885793000	-0.193311000	-1.207954000
H	-0.495324000	-1.893521000	-0.104656000
H	-0.601534000	-0.943078000	1.370774000
H	-1.614161000	0.274423000	-1.230947000
H	-3.834186000	-0.863506000	-0.912066000
H	-2.537508000	-2.026709000	-1.288894000
H	-3.129065000	-1.860999000	0.382535000
H	-3.338080000	1.295673000	0.258915000
H	-2.712432000	0.375400000	1.647381000
H	-1.723558000	1.653082000	0.908420000
H	0.480341000	1.368458000	1.362213000
H	2.081590000	1.396497000	0.648545000
H	0.780508000	3.342768000	-0.155255000
H	1.193879000	2.270840000	-1.512709000
H	-0.456994000	2.282342000	-0.862088000

2-Methyloctane

C	-2.861157000	0.062934000	-0.338893000
C	-1.537801000	-0.560270000	0.144958000
C	-0.267193000	0.212352000	-0.228257000
C	1.022610000	-0.502148000	0.191024000
C	2.298065000	0.266036000	-0.170024000
C	3.588519000	-0.448707000	0.245014000
C	4.856056000	0.328090000	-0.118686000
C	-4.034216000	-0.889355000	-0.069758000
C	-3.130088000	1.438078000	0.288339000
H	-2.783070000	0.200171000	-1.435433000
H	-1.581922000	-0.683969000	1.244759000
H	-1.461716000	-1.582880000	-0.269261000
H	-0.250869000	0.380504000	-1.322044000
H	-0.284445000	1.216073000	0.232530000
H	1.001594000	-0.681492000	1.282776000
H	1.054530000	-1.503983000	-0.277637000
H	2.318169000	0.448919000	-1.261413000
H	2.268402000	1.266635000	0.302009000
H	3.567920000	-0.631834000	1.335403000
H	3.618942000	-1.447704000	-0.228098000
H	5.765507000	-0.210428000	0.193394000
H	4.924495000	0.495318000	-1.207337000
H	4.871498000	1.318574000	0.367593000
H	-4.984051000	-0.473940000	-0.444855000
H	-3.879639000	-1.867750000	-0.554002000
H	-4.154962000	-1.069756000	1.013132000
H	-4.085324000	1.855866000	-0.070382000
H	-3.193124000	1.362410000	1.388739000
H	-2.340703000	2.167205000	0.047822000

3,3,4-Trimethylhexane

C	1.690743000	2.060507000	0.218005000
C	1.507036000	0.831303000	-0.684173000
C	0.828174000	-0.448254000	-0.091862000
C	1.410932000	-1.642948000	-0.881258000
C	1.200715000	-0.623230000	1.393401000
C	-0.733716000	-0.432500000	-0.308110000
C	-1.445459000	-1.629959000	0.348914000
C	-1.427797000	0.886670000	0.097120000
C	-2.927535000	0.946433000	-0.220715000
H	2.154802000	2.879244000	-0.356723000
H	0.746828000	2.444588000	0.630825000
H	2.358027000	1.845931000	1.067635000
H	0.960222000	1.122304000	-1.599616000
H	2.513484000	0.539444000	-1.032283000
H	0.973379000	-2.605121000	-0.575562000
H	1.236870000	-1.523517000	-1.964150000
H	2.499814000	-1.715253000	-0.724849000
H	0.875366000	-1.602947000	1.775933000
H	2.293923000	-0.571156000	1.528010000
H	0.751873000	0.151107000	2.034841000
H	-0.872600000	-0.531203000	-1.403076000
H	-2.477759000	-1.723442000	-0.019988000
H	-0.945688000	-2.586072000	0.136601000
H	-1.500060000	-1.516888000	1.444935000
H	-1.281311000	1.068655000	1.177695000
H	-0.942529000	1.724347000	-0.425557000
H	-3.325705000	1.954889000	-0.022701000
H	-3.120368000	0.717456000	-1.283404000
H	-3.517124000	0.239508000	0.383511000

3,3-Dimethylheptane

0 1

C	-2.701034000	-1.655296000	0.075154000
C	-2.373842000	-0.295418000	-0.551340000
C	-1.121132000	0.456476000	-0.019192000
C	-1.062210000	1.813812000	-0.747749000
C	-1.257793000	0.707881000	1.495575000
C	0.149643000	-0.378389000	-0.331976000
C	1.503136000	0.209803000	0.088913000
C	2.682769000	-0.702310000	-0.270142000
C	4.039004000	-0.133276000	0.152781000
H	-3.611969000	-2.075889000	-0.381179000
H	-1.895007000	-2.391312000	-0.074579000
H	-2.886260000	-1.579045000	1.158788000
H	-2.256461000	-0.424469000	-1.643066000
H	-3.246081000	0.370694000	-0.420386000
H	-0.218422000	2.431448000	-0.402749000
H	-0.957409000	1.677367000	-1.837538000
H	-1.985366000	2.391184000	-0.572273000
H	-0.459662000	1.368810000	1.868629000
H	-2.220489000	1.194614000	1.726545000
H	-1.208561000	-0.228561000	2.074156000
H	0.047882000	-1.369086000	0.145378000
H	0.172883000	-0.570617000	-1.421514000
H	1.659232000	1.194490000	-0.386834000
H	1.514669000	0.391461000	1.178685000
H	2.534735000	-1.691722000	0.201172000
H	2.682886000	-0.885237000	-1.360734000
H	4.865160000	-0.810408000	-0.118293000
H	4.233578000	0.839788000	-0.330129000
H	4.083420000	0.026781000	1.243777000

3,4-Dimethylheptane

0 1

C	3.658158000	-1.067092000	0.198966000
C	2.592416000	-0.159850000	-0.421270000
C	1.255369000	-0.207800000	0.329207000
C	0.146242000	0.703080000	-0.244206000
C	-1.264351000	0.236696000	0.223096000
C	-2.351189000	1.305390000	0.024455000
C	0.444044000	2.170737000	0.104475000
C	-1.680199000	-1.073266000	-0.484453000
C	-2.812590000	-1.844625000	0.200836000
H	4.603960000	-1.025187000	-0.365171000
H	3.877581000	-0.773374000	1.239822000
H	3.327256000	-2.119758000	0.217681000
H	2.974058000	0.875720000	-0.454856000
H	2.424913000	-0.454909000	-1.473813000
H	0.912874000	-1.256672000	0.343786000
H	1.420221000	0.065553000	1.389524000
H	0.166655000	0.610263000	-1.348769000
H	-1.193077000	0.027900000	1.309789000
H	-3.344589000	0.913689000	0.292967000
H	-2.180347000	2.199116000	0.642154000
H	-2.398767000	1.628417000	-1.030698000
H	-0.222832000	2.871294000	-0.419112000
H	0.335927000	2.349080000	1.189156000
H	1.475321000	2.441181000	-0.170878000
H	-1.972481000	-0.830103000	-1.523563000
H	-0.809334000	-1.743843000	-0.569390000
H	-3.052979000	-2.768043000	-0.350900000
H	-2.530784000	-2.135314000	1.227474000
H	-3.740967000	-1.254625000	0.268160000

3,5-Dimethylheptane

0 1

C	3.828340000	-0.648694000	-0.310950000
C	2.524727000	-0.652537000	0.493065000
C	1.309862000	-0.018644000	-0.215072000
C	0.027986000	-0.297152000	0.598523000
C	-1.312075000	0.142873000	-0.047447000
C	-2.380244000	-0.958671000	0.120548000
C	-3.715027000	-0.689479000	-0.579904000
C	1.529569000	1.473841000	-0.498485000
C	-1.799423000	1.491303000	0.506884000
H	4.624987000	-1.185992000	0.228758000
H	3.696992000	-1.145075000	-1.288034000
H	4.197585000	0.371166000	-0.504284000
H	2.270633000	-1.696777000	0.749477000
H	2.685007000	-0.136164000	1.458807000
H	1.194126000	-0.532080000	-1.191210000
H	-0.007904000	-1.384991000	0.784584000
H	0.127412000	0.169032000	1.597506000
H	-1.142877000	0.261521000	-1.135990000
H	-1.961341000	-1.905836000	-0.264382000
H	-2.555455000	-1.123165000	1.201164000
H	-4.390833000	-1.555078000	-0.486298000
H	-4.239789000	0.181713000	-0.156601000
H	-3.569084000	-0.497909000	-1.657332000
H	0.687448000	1.912157000	-1.055610000
H	1.643942000	2.042626000	0.441541000
H	2.435286000	1.641900000	-1.101781000
H	-2.687297000	1.862244000	-0.029082000
H	-2.068273000	1.396414000	1.574188000
H	-1.022853000	2.267238000	0.431602000

3-Ethyl-2,3-dimethylpentane

0 1

C	2.563259000	-1.110634000	0.096943000
C	1.258290000	-1.026873000	-0.710252000
C	0.000010000	-0.341616000	-0.064619000
C	-1.258257000	-1.026904000	-0.710240000
C	-2.563204000	-1.110713000	0.096987000
C	0.000019000	-0.565639000	1.459138000
C	-0.000015000	1.192029000	-0.433518000
C	-1.249561000	1.967183000	0.019593000
C	1.249462000	1.967243000	0.019680000
H	3.348241000	-1.579746000	-0.519416000
H	2.945221000	-0.132661000	0.422597000
H	2.446262000	-1.734749000	0.996897000
H	1.460149000	-0.537172000	-1.680403000
H	0.985164000	-2.065040000	-0.963596000
H	-1.460149000	-0.537194000	-1.680380000
H	-0.985105000	-2.065059000	-0.963605000
H	-3.348197000	-1.579812000	-0.519368000
H	-2.446171000	-1.734860000	0.996914000
H	-2.945169000	-0.132757000	0.422690000
H	-0.885935000	-0.126496000	1.940001000
H	0.000018000	-1.642425000	1.698804000
H	0.885979000	-0.126500000	1.939989000
H	0.000021000	1.214943000	-1.540411000
H	-1.164065000	3.023260000	-0.285006000
H	-2.174542000	1.577862000	-0.427633000
H	-1.369946000	1.956301000	1.115979000
H	1.163963000	3.023305000	-0.284975000
H	1.369738000	1.956413000	1.116078000
H	2.174500000	1.577930000	-0.427433000

3-Ethyl-2-methylhexane

0 1

C	3.765494000	-0.085944000	0.059832000
C	2.367319000	0.131090000	-0.525119000
C	1.264713000	-0.554082000	0.291960000
C	-0.173378000	-0.372155000	-0.249790000
C	-1.068382000	-1.506808000	0.299401000
C	-2.484504000	-1.626984000	-0.273956000
C	-0.685737000	1.082154000	-0.000025000
C	-1.681009000	1.573575000	-1.064033000
C	-1.236922000	1.310181000	1.416148000
H	4.540781000	0.403382000	-0.551762000
H	3.839816000	0.323044000	1.082121000
H	4.013841000	-1.159655000	0.116969000
H	2.171931000	1.215451000	-0.599369000
H	2.338502000	-0.253089000	-1.561693000
H	1.494119000	-1.634224000	0.334775000
H	1.312075000	-0.200892000	1.339786000
H	-0.120233000	-0.507318000	-1.347989000
H	-0.540630000	-2.456496000	0.100067000
H	-1.122897000	-1.431416000	1.400508000
H	-2.963023000	-2.556913000	0.075127000
H	-3.137297000	-0.793936000	0.028523000
H	-2.471685000	-1.655442000	-1.376835000
H	0.200461000	1.734244000	-0.099169000
H	-1.912084000	2.641960000	-0.915338000
H	-1.265964000	1.462065000	-2.079489000
H	-2.634762000	1.025054000	-1.033439000
H	-1.426885000	2.382703000	1.587212000
H	-2.190046000	0.780794000	1.579731000
H	-0.531582000	0.974529000	2.193753000

3-Ethyl-3-methylhexane

0 1

C	-2.358199000	-1.771983000	-0.108898000
C	-1.917325000	-0.395170000	-0.617074000
C	-0.643517000	0.232925000	0.024056000
C	-0.599195000	1.699033000	-0.495955000
C	0.615641000	2.566625000	-0.150441000
C	-0.778606000	0.227657000	1.558527000
C	0.599198000	-0.577464000	-0.454780000
C	1.873866000	-0.551698000	0.403918000
C	3.018519000	-1.335254000	-0.245808000
H	-3.227365000	-2.129895000	-0.684388000
H	-1.567047000	-2.532834000	-0.211258000
H	-2.657901000	-1.745583000	0.950804000
H	-1.753916000	-0.450235000	-1.709113000
H	-2.755569000	0.311062000	-0.475842000
H	-0.702201000	1.668545000	-1.596304000
H	-1.508216000	2.208655000	-0.126828000
H	0.458384000	3.595989000	-0.512606000
H	0.793128000	2.629152000	0.935448000
H	1.539882000	2.197450000	-0.621793000
H	0.034112000	0.787283000	2.045285000
H	-1.730716000	0.694420000	1.863555000
H	-0.761497000	-0.793374000	1.970866000
H	0.309325000	-1.637852000	-0.559322000
H	0.849965000	-0.243731000	-1.479576000
H	2.204172000	0.482793000	0.589740000
H	1.660979000	-0.984736000	1.396464000
H	3.920205000	-1.334291000	0.387838000
H	2.735411000	-2.387484000	-0.421663000
H	3.296566000	-0.903370000	-1.222575000

3-Ethyl-4-methylhexane

0 1

C	-2.233294000	-1.989494000	-0.449787000
C	-2.057609000	-0.617274000	0.208076000
C	-0.821311000	0.185381000	-0.264273000
C	-1.030029000	1.677859000	0.080753000
C	-0.011488000	2.678388000	-0.475445000
C	0.503230000	-0.459775000	0.256213000
C	1.696353000	-0.250542000	-0.702674000
C	2.919688000	-1.119242000	-0.391170000
C	0.838559000	-0.064144000	1.703455000
H	-3.145942000	-2.487922000	-0.084446000
H	-2.321678000	-1.896264000	-1.545916000
H	-1.389028000	-2.667352000	-0.243463000
H	-2.962226000	-0.017835000	0.003990000
H	-2.022180000	-0.734027000	1.307151000
H	-0.798812000	0.111503000	-1.369469000
H	-2.026211000	1.959141000	-0.304842000
H	-1.100808000	1.797035000	1.177087000
H	-0.349318000	3.712216000	-0.294385000
H	0.980854000	2.575934000	-0.010530000
H	0.118461000	2.558906000	-1.564473000
H	0.324512000	-1.551015000	0.261777000
H	1.356018000	-0.475291000	-1.729476000
H	1.999234000	0.809876000	-0.711302000
H	3.701904000	-0.987607000	-1.156433000
H	3.370978000	-0.868902000	0.582446000
H	2.654764000	-2.190726000	-0.368136000
H	1.653126000	-0.685054000	2.107355000
H	1.159028000	0.988152000	1.780166000
H	-0.027271000	-0.197712000	2.372013000

3-Ethylheptane

0 1

C	2.122467000	-2.323790000	0.362348000
C	2.350383000	-0.917534000	-0.199012000
C	1.240260000	0.098960000	0.142103000
C	1.742617000	1.529806000	-0.141146000
C	0.793496000	2.665544000	0.250228000
C	-0.085125000	-0.226582000	-0.596155000
C	-1.355277000	-0.102497000	0.256422000
C	-2.642774000	-0.403850000	-0.517958000
C	-3.904801000	-0.309419000	0.342843000
H	2.943666000	-3.002915000	0.081224000
H	2.066649000	-2.306938000	1.464321000
H	1.185873000	-2.772504000	-0.008041000
H	3.312659000	-0.535330000	0.186237000
H	2.464664000	-0.967946000	-1.298529000
H	1.052880000	0.021973000	1.231841000
H	2.700536000	1.671783000	0.391044000
H	1.983273000	1.610104000	-1.218575000
H	1.259896000	3.646635000	0.063213000
H	-0.147348000	2.638534000	-0.322857000
H	0.532527000	2.621307000	1.321454000
H	-0.174999000	0.423657000	-1.486629000
H	-0.044764000	-1.255938000	-0.993365000
H	-1.278528000	-0.794097000	1.116963000
H	-1.422653000	0.910333000	0.692070000
H	-2.725224000	0.293921000	-1.371976000
H	-2.572840000	-1.414806000	-0.961040000
H	-4.812606000	-0.527943000	-0.242566000
H	-3.870265000	-1.023531000	1.183442000
H	-4.020861000	0.699872000	0.773730000

3-Methyloctane

0 1

C	-2.878688000	-0.810751000	0.236479000
C	-1.737372000	0.136644000	-0.185185000
C	-0.382612000	-0.421091000	0.294032000
C	0.860649000	0.304431000	-0.234651000
C	2.174444000	-0.368185000	0.178964000
C	3.425442000	0.350021000	-0.338175000
C	4.731461000	-0.333412000	0.073482000
C	-4.251758000	-0.486848000	-0.359528000
H	-2.600624000	-1.839734000	-0.054577000
H	-1.715504000	0.155138000	-1.293567000
H	-0.364686000	-0.418687000	1.401312000
H	-0.318971000	-1.483941000	-0.004374000
H	0.810577000	0.355508000	-1.339145000
H	0.870236000	1.351295000	0.117321000
H	2.221121000	-0.431422000	1.282941000
H	2.179442000	-1.414292000	-0.182320000
H	3.375817000	0.417612000	-1.440852000
H	3.423281000	1.393441000	0.028142000
H	5.611064000	0.207172000	-0.311990000
H	4.827083000	-0.385872000	1.171633000
H	4.780705000	-1.366834000	-0.310438000
H	-4.995781000	-1.247048000	-0.071174000
H	-4.635458000	0.488862000	-0.020730000
H	-4.212508000	-0.462932000	-1.462351000
C	-1.974601000	1.572038000	0.306488000
H	-2.947199000	-0.818231000	1.341117000
H	-1.181564000	2.256784000	-0.031671000
H	-2.929632000	1.978090000	-0.061288000
H	-1.999186000	1.608009000	1.410571000

4,4-Dimethylheptane

0 1

C	-3.535166000	-1.099385000	-0.193833000
C	-2.126828000	-0.804881000	0.332012000
C	-1.438789000	0.322225000	-0.449810000
C	-0.005472000	0.740339000	-0.017602000
C	0.420971000	1.909405000	-0.928120000
C	-0.011947000	1.226105000	1.445734000
C	0.958709000	-0.463275000	-0.195759000
C	2.449740000	-0.220178000	0.077064000
C	3.285150000	-1.493753000	-0.084330000
H	-4.018623000	-1.908880000	0.376705000
H	-4.183463000	-0.208987000	-0.124744000
H	-3.511074000	-1.406295000	-1.253584000
H	-2.190246000	-0.538629000	1.401508000
H	-1.524534000	-1.728307000	0.280935000
H	-1.405073000	0.037694000	-1.518613000
H	-2.083138000	1.220231000	-0.400428000
H	1.407226000	2.311064000	-0.648163000
H	0.471250000	1.595355000	-1.984548000
H	-0.302557000	2.739047000	-0.861809000
H	0.962490000	1.650875000	1.734449000
H	-0.768958000	2.014936000	1.593074000
H	-0.237687000	0.409110000	2.149758000
H	0.622334000	-1.284379000	0.461996000
H	0.849827000	-0.842485000	-1.229887000
H	2.839935000	0.554433000	-0.605258000
H	2.587342000	0.174222000	1.099181000
H	4.353345000	-1.303795000	0.109070000
H	2.954468000	-2.281796000	0.613892000
H	3.198937000	-1.903030000	-1.105673000

4-Ethylheptane

0 1

C	3.894001000	-0.907550000	0.007979000
C	2.639535000	-0.118022000	-0.375075000
C	1.353590000	-0.713544000	0.210107000
C	0.062154000	0.060399000	-0.129468000
C	-1.164198000	-0.836066000	0.139885000
C	-2.533305000	-0.261617000	-0.241700000
C	-3.674058000	-1.258401000	-0.017191000
C	-0.007687000	1.416387000	0.622601000
C	-0.532055000	2.592261000	-0.209517000
H	4.801125000	-0.468114000	-0.437613000
H	4.035737000	-0.927436000	1.102194000
H	3.828139000	-1.954836000	-0.333625000
H	2.756572000	0.930350000	-0.047053000
H	2.549348000	-0.081336000	-1.476634000
H	1.256082000	-1.752644000	-0.154499000
H	1.449097000	-0.789409000	1.310848000
H	0.086310000	0.274023000	-1.216683000
H	-1.023996000	-1.787839000	-0.405707000
H	-1.174157000	-1.106554000	1.214108000
H	-2.736418000	0.654342000	0.339944000
H	-2.518081000	0.046845000	-1.303448000
H	-4.649373000	-0.827475000	-0.295649000
H	-3.529970000	-2.173961000	-0.616030000
H	-3.734653000	-1.564461000	1.041352000
H	-0.628541000	1.294548000	1.529321000
H	0.997175000	1.682440000	0.992324000
H	-0.564189000	3.519849000	0.385848000
H	0.119495000	2.779406000	-1.080161000
H	-1.547566000	2.409177000	-0.594575000

4-Methyloctane

0 1

C	4.357721000	-0.780647000	-0.012950000
C	3.154777000	0.088229000	-0.390161000
C	1.840471000	-0.419869000	0.214935000
C	0.570416000	0.350473000	-0.198003000
C	-0.684267000	-0.392858000	0.302723000
C	-2.026156000	0.148264000	-0.204283000
C	-3.226311000	-0.696643000	0.238780000
C	-4.567415000	-0.153128000	-0.259473000
C	0.603115000	1.809763000	0.279785000
H	5.289752000	-0.397651000	-0.459213000
H	4.500482000	-0.812512000	1.080873000
H	4.225336000	-1.820684000	-0.357250000
H	3.345160000	1.126481000	-0.067585000
H	3.057461000	0.124155000	-1.491391000
H	1.713126000	-1.480602000	-0.070913000
H	1.921174000	-0.414771000	1.319637000
H	0.531994000	0.356023000	-1.305936000
H	-0.601150000	-1.454433000	0.004631000
H	-0.684206000	-0.391539000	1.410156000
H	-2.175966000	1.186021000	0.143474000
H	-2.005561000	0.198315000	-1.309924000
H	-3.092897000	-1.734759000	-0.117921000
H	-3.239941000	-0.756726000	1.342914000
H	-5.408848000	-0.781795000	0.074119000
H	-4.747800000	0.870066000	0.112503000
H	-4.598177000	-0.112248000	-1.361828000
H	-0.286889000	2.368051000	-0.049047000
H	0.640778000	1.859956000	1.382964000
H	1.482067000	2.347157000	-0.108381000

n-Nonane

0 1

C	-5.125606000	0.368964000	-0.000001000
C	-3.850369000	-0.477241000	0.000000000
C	-2.566400000	0.359332000	-0.000001000
C	-1.283338000	-0.477982000	0.000000000
C	0.000000000	0.359304000	-0.000001000
C	1.283338000	-0.477982000	0.000001000
C	2.566400000	0.359332000	0.000000000
C	3.850369000	-0.477241000	0.000001000
C	5.125606000	0.368964000	0.000000000
H	-6.030238000	-0.260441000	-0.000001000
H	-5.172910000	1.021127000	0.888949000
H	-5.172909000	1.021124000	-0.888953000
H	-3.849475000	-1.144120000	0.882127000
H	-3.849474000	-1.144123000	-0.882125000
H	-2.567088000	1.027241000	-0.882495000
H	-2.567088000	1.027244000	0.882491000
H	-1.283329000	-1.145707000	0.882380000
H	-1.283328000	-1.145710000	-0.882377000
H	0.000000000	1.027012000	-0.882350000
H	0.000000000	1.027014000	0.882347000
H	1.283328000	-1.145707000	0.882380000
H	1.283329000	-1.145710000	-0.882377000
H	2.567088000	1.027241000	-0.882494000
H	2.567087000	1.027244000	0.882492000
H	3.849474000	-1.144120000	0.882128000
H	3.849475000	-1.144123000	-0.882123000
H	6.030238000	-0.260441000	0.000002000
H	5.172910000	1.021124000	-0.888952000
H	5.172909000	1.021127000	0.888951000

2,2,3,3,4-Pentamethylpentane

0 1

C	-0.1828360000	0.5265010000	0.1105920000
C	1.2363330000	-0.2046670000	-0.0808220000
C	-1.4045840000	-0.3204840000	-0.4382960000
C	-0.1614730000	1.8614940000	-0.6752140000
C	-0.4044180000	0.8760150000	1.5987520000
C	2.4039970000	0.8127760000	-0.0064700000
C	1.5330600000	-1.2497640000	1.0225970000
C	1.3449330000	-0.8934790000	-1.4598460000
C	-1.5625160000	-1.7514960000	0.1110160000
C	-2.7716320000	0.3749250000	-0.2547290000
H	-1.2459350000	-0.4087720000	-1.5274560000
H	0.5902500000	2.5546460000	-0.2740430000
H	0.0564780000	1.7094640000	-1.7446990000
H	-1.1272720000	2.3797370000	-0.6083620000
H	-0.5850690000	-0.0121300000	2.2220530000
H	-1.2694160000	1.5459270000	1.7190970000
H	0.4659890000	1.4026240000	2.0202230000
H	2.3606400000	1.4366240000	0.9005080000
H	3.3612180000	0.2669510000	0.0220370000
H	2.4389140000	1.4818380000	-0.8782640000
H	0.7486840000	-2.0068470000	1.1381810000
H	2.4682630000	-1.7815780000	0.7799280000
H	1.6818800000	-0.7717240000	2.0028180000
H	0.6803190000	-1.7633530000	-1.5591380000
H	1.1175130000	-0.2001830000	-2.2851380000
H	2.3752470000	-1.2543690000	-1.6149600000
H	-1.6539920000	-1.7634840000	1.2096990000
H	-2.4873310000	-2.1968490000	-0.2914650000
H	-0.7397980000	-2.4224300000	-0.1668910000
H	-2.8071320000	1.3944010000	-0.6621060000
H	-3.0640310000	0.4246110000	0.8071670000
H	-3.5505970000	-0.2060220000	-0.7746690000

2,2,3,3-Tetramethylhexane

0 1

C	0.1111560000	0.5151160000	-0.0340670000
C	1.4387400000	-0.3725010000	0.0170730000
C	-1.1447410000	-0.4058700000	-0.0953500000
C	0.0253840000	1.4271710000	1.2105090000
C	0.1154990000	1.4262230000	-1.2824690000
C	1.6147150000	-1.0454530000	1.3971660000
C	1.4190170000	-1.4909540000	-1.0494090000
C	2.6995870000	0.4832560000	-0.2420000000
C	-2.5145900000	0.2517760000	0.1337320000
C	-3.6663610000	-0.7408860000	-0.0532540000
H	-1.1697740000	-0.9012140000	-1.0810720000
H	-1.0385750000	-1.2169970000	0.6460230000
H	-0.7773370000	2.1713790000	1.0935220000
H	0.9589790000	1.9888770000	1.3685630000
H	-0.1850980000	0.8601800000	2.1303600000
H	0.2771240000	0.8578570000	-2.2119910000
H	0.8938420000	2.2014510000	-1.2209350000
H	-0.8481730000	1.9492820000	-1.3828190000
H	0.7268130000	-1.6213820000	1.7015850000
H	1.8309230000	-0.3141260000	2.1905750000
H	2.4627800000	-1.7491010000	1.3634730000
H	0.6659870000	-2.2634390000	-0.8329110000
H	2.3998750000	-1.9935270000	-1.0790480000
H	1.2232590000	-1.1011830000	-2.0608350000
H	2.7618880000	1.3560290000	0.4267810000
H	3.6032860000	-0.1239290000	-0.0684620000
H	2.7484450000	0.8479670000	-1.2790000000
H	-2.5684630000	0.6753010000	1.1508670000
H	-2.6555370000	1.0998160000	-0.5590630000
H	-4.6436160000	-0.2643360000	0.1267450000
H	-3.6793910000	-1.1533780000	-1.0766890000
H	-3.5776700000	-1.5927860000	0.6427380000

2,2,3,4,4-Pentamethylpentane

0 1

C	-0.0055350000	0.5062810000	-0.4547120000
C	-1.3814440000	-0.1589240000	-0.0231390000
C	1.3783330000	-0.1507880000	-0.0288110000
C	0.0040140000	2.0155430000	-0.1180890000
C	-1.6532300000	-1.4723810000	-0.7933350000
C	-1.4932780000	-0.4303320000	1.4904020000
C	-2.5445830000	0.7891870000	-0.4103880000
C	2.4605460000	0.3587980000	-1.0132620000
C	1.8177760000	0.2360600000	1.4013890000
C	1.4140830000	-1.6895250000	-0.1341090000
H	-0.0143130000	0.4350400000	-1.5586170000
H	0.9859500000	2.4700920000	-0.3077200000
H	-0.2422040000	2.2058860000	0.9388870000
H	-0.7203210000	2.5711750000	-0.7287420000
H	-1.5519930000	-1.3215760000	-1.8813310000
H	-2.6863550000	-1.8079150000	-0.6033820000
H	-0.9899400000	-2.2951030000	-0.5043920000
H	-1.3095650000	0.4776940000	2.0867120000
H	-2.5103510000	-0.7803090000	1.7353690000
H	-0.7923520000	-1.2064480000	1.8307380000
H	-2.4940440000	1.0789080000	-1.4735930000
H	-2.5640910000	1.7084710000	0.1924810000
H	-3.5074330000	0.2764230000	-0.2538010000
H	3.4454240000	-0.0589970000	-0.7470550000
H	2.2343400000	0.0454270000	-2.0460890000
H	2.5606830000	1.4544040000	-1.0096590000
H	1.1092080000	-0.1110170000	2.1669170000
H	1.9365070000	1.3232200000	1.5209090000
H	2.7948750000	-0.2231700000	1.6267510000
H	0.7692610000	-2.1839250000	0.6060420000
H	1.1235170000	-2.0405960000	-1.1362510000
H	2.4431050000	-2.0411970000	0.0501480000

2,2,3,4-Tetramethylhexane

0 1

C	0.0004230000	-0.3705770000	0.2820000000
C	-1.4892350000	-0.1622510000	-0.2029300000

C	1.0803720000	0.5744290000	-0.3348610000
C	2.5253720000	0.0992520000	-0.0458770000
C	0.1178020000	-0.4537170000	1.8148500000
C	-2.2450440000	-1.4981070000	-0.0163990000
C	-2.2518270000	0.9223610000	0.5880200000
C	-1.5463570000	0.1832680000	-1.7045980000
C	0.9583410000	2.0572410000	0.0534480000
C	2.8670770000	-1.3249080000	-0.4941080000
H	0.2588180000	-1.3722120000	-0.1048090000
H	0.9637110000	0.5147210000	-1.4309060000
H	2.7507810000	0.2124080000	1.0298820000
H	3.2059450000	0.8003280000	-0.5611710000
H	1.1039920000	-0.8381430000	2.1172860000
H	-0.6325960000	-1.1343050000	2.2442590000
H	-0.0135740000	0.5256600000	2.3010610000
H	-3.2984330000	-1.3952260000	-0.3256860000
H	-2.2439610000	-1.8341270000	1.0324030000
H	-1.7942900000	-2.2989720000	-0.6260750000
H	-2.3279370000	0.6728130000	1.6576090000
H	-3.2811400000	1.0136070000	0.2021150000
H	-1.7836600000	1.9139060000	0.5084190000
H	-1.1372320000	1.1810610000	-1.9251150000
H	-2.5917570000	0.1747020000	-2.0551220000
H	-0.9901430000	-0.5521110000	-2.3099000000
H	1.0031740000	2.2042400000	1.1451320000
H	1.7895990000	2.6341310000	-0.3850140000
H	0.0245010000	2.5118940000	-0.3069390000
H	2.6140070000	-1.4830910000	-1.5568350000
H	2.3333560000	-2.0911140000	0.0907530000
H	3.9452990000	-1.5221130000	-0.3786110000

2,2,3,5-Tetramethylhexane

0 1

C	-1.6948480000	-0.2536760000	-0.1043650000
C	-0.3527720000	0.3794410000	0.4139320000
C	0.8548100000	0.1180040000	-0.5181970000
C	2.2432120000	0.1650200000	0.1567600000
C	-0.4590380000	1.8866140000	0.7079880000
C	-2.8651630000	0.1556530000	0.8140650000
C	-1.5931880000	-1.7926300000	-0.0595430000
C	-2.0199080000	0.1859880000	-1.5461060000
C	2.4855800000	-1.0444350000	1.0711990000
C	3.3505740000	0.2616960000	-0.9017470000
H	-0.1509710000	-0.1242230000	1.3775280000
H	0.8357180000	0.8638590000	-1.3331140000
H	0.7606480000	-0.8647760000	-1.0107130000
H	2.2985190000	1.0773530000	0.7799410000
H	-1.1659900000	2.1085510000	1.5206160000
H	0.5179680000	2.2932550000	1.0131910000
H	-0.7801820000	2.4519140000	-0.1831990000
H	-3.7784120000	-0.3979930000	0.5401190000
H	-3.0987830000	1.2285620000	0.7399660000
H	-2.6439330000	-0.0708400000	1.8710910000
H	-0.7905320000	-2.1817610000	-0.7044480000
H	-1.4006260000	-2.1476480000	0.9669320000
H	-2.5362060000	-2.2520240000	-0.3993760000
H	-1.2600970000	-0.1612960000	-2.2637290000
H	-2.9890640000	-0.2324460000	-1.8651980000
H	-2.0914990000	1.2821000000	-1.6351600000
H	2.4782070000	-1.9832400000	0.4891650000
H	3.4651160000	-0.9723560000	1.5721960000
H	1.7197880000	-1.1349750000	1.8578790000
H	4.3497050000	0.2991580000	-0.4370520000
H	3.3301240000	-0.6141360000	-1.5744060000
H	3.2349470000	1.1629100000	-1.5261520000

3-Ethyl-2,2,3-trimethyl-pentane

0 1

C	0.3153440000	-0.1932590000	0.3132990000
C	-1.2152360000	-0.1386280000	-0.1604000000
C	1.2094830000	-0.7171640000	-0.8497130000
C	0.8549650000	1.1792180000	0.8292190000
C	0.4474150000	-1.1574410000	1.5180320000
C	-2.0676510000	0.7236370000	0.7969900000
C	-1.8459170000	-1.5524860000	-0.1804100000
C	-1.3848370000	0.4335070000	-1.5881010000

C	2.6800690000	-1.0084340000	-0.5206050000
C	0.9440210000	2.3822400000	-0.1183280000
H	1.1834390000	0.0039450000	-1.6812290000
H	0.7599350000	-1.6428990000	-1.2479300000
H	0.2724190000	1.4711230000	1.7187480000
H	1.8706840000	0.9878850000	1.2145340000
H	0.3039700000	-2.2091750000	1.2290780000
H	1.4452380000	-1.0769910000	1.9751780000
H	-0.2828320000	-0.9230610000	2.3082250000
H	-3.1339520000	0.6329810000	0.5315450000
H	-1.9666900000	0.4051780000	1.8466150000
H	-1.8099990000	1.7919110000	0.7423550000
H	-1.2534070000	-2.2685040000	-0.7718050000
H	-1.9717090000	-1.9701280000	0.8291730000
H	-2.8471800000	-1.5036160000	-0.6394510000
H	-0.9755600000	-0.2405440000	-2.3554950000
H	-0.9118220000	1.4158510000	-1.7169000000
H	-2.4587670000	0.5548060000	-1.8070940000
H	2.7926170000	-1.7978200000	0.2389680000
H	3.2069230000	-1.3517630000	-1.4258960000
H	3.2140970000	-0.1162110000	-0.1552530000
H	1.4943990000	3.2011700000	0.3742350000
H	-0.0417200000	2.7833180000	-0.3982160000
H	1.4839740000	2.1454020000	-1.0492790000

2,2,3-Trimethylheptane

0 1

C	1.9395090000	-0.2892990000	-0.0509620000
C	0.6009350000	0.4964520000	-0.2972950000
C	-0.6323510000	-0.1616060000	0.3649370000
C	3.1318930000	0.4904960000	-0.6431520000
C	1.8806980000	-1.6524400000	-0.7722280000
C	2.1989090000	-0.5262780000	1.4501590000
C	0.6787640000	1.9814740000	0.0988770000
C	-1.9722810000	0.1984900000	-0.2909750000
C	-3.1791090000	-0.4500960000	0.3964500000
C	-4.5130020000	-0.1104000000	-0.2726390000
H	0.4385700000	0.4606750000	-1.3922340000
H	-0.6665210000	0.1195680000	1.4337560000
H	-0.5343370000	-1.2595280000	0.3465090000
H	4.0484310000	-0.1207840000	-0.5991670000
H	2.9560060000	0.7500810000	-1.7010750000
H	3.3348100000	1.4236080000	-0.0959080000
H	1.0691520000	-2.2946870000	-0.3969910000
H	1.7310860000	-1.5208320000	-1.8572510000
H	2.8244250000	-2.2049600000	-0.6310030000
H	1.4252410000	-1.1639560000	1.9059610000
H	2.2324090000	0.4194580000	2.0148570000
H	3.1686780000	-1.0304230000	1.5967310000
H	-2.1132770000	1.2945830000	-0.2999400000
H	-1.9501410000	-0.1114770000	-1.3531350000
H	1.4173120000	2.5371890000	-0.4970030000
H	0.9424060000	2.1048690000	1.1631340000
H	-0.2928550000	2.4770200000	-0.0524970000
H	-3.2066420000	-0.1353050000	1.4561610000
H	-3.0428580000	-1.5474670000	0.4117230000
H	-5.3596950000	-0.5912120000	0.2435400000
H	-4.6961920000	0.9778410000	-0.2711190000
H	-4.5297990000	-0.4450180000	-1.3240760000

2,2,4,4-Tetramethylhexane

0 1

C	1.0475440000	-0.4591020000	-0.0057480000
C	-0.1193720000	0.5625920000	-0.1879310000
C	-1.6370590000	0.2293860000	-0.0168100000
C	2.3296080000	0.1753480000	-0.6283040000
C	1.3189140000	-0.7696510000	1.4820530000
C	0.8189430000	-1.7885360000	-0.7503760000
C	-2.3690830000	1.5909080000	0.0009150000
C	-2.1977900000	-0.5777750000	-1.2066590000
C	-1.9687400000	-0.5032780000	1.2964670000
C	2.8111530000	1.5148760000	-0.0599710000
H	0.0940340000	1.3990760000	0.4997480000
H	-0.0207580000	0.9902450000	-1.2030080000
H	3.1465950000	-0.5621020000	-0.5272560000
H	2.1605630000	0.2899110000	-1.7147500000

H	2.2485590000	-1.3546500000	1.5880000000
H	1.4333220000	0.1482490000	2.0804850000
H	0.5101290000	-1.3589940000	1.9355930000
H	1.7253760000	-2.4146930000	-0.6931180000
H	-0.0060620000	-2.3721260000	-0.3158600000
H	0.5968230000	-1.6253400000	-1.8174180000
H	-2.1562940000	2.1715500000	-0.9124390000
H	-2.0596280000	2.2013980000	0.8655990000
H	-3.4611100000	1.4505760000	0.0611530000
H	-1.9724900000	-0.0805950000	-2.1652030000
H	-3.2949110000	-0.6612840000	-1.1272080000
H	-1.7951340000	-1.5986270000	-1.2550970000
H	-1.5706540000	-1.5290470000	1.3129540000
H	-1.5678230000	0.0314580000	2.1728980000
H	-3.0621520000	-0.5763850000	1.4237040000
H	3.7432420000	1.8255920000	-0.5595340000
H	2.0778590000	2.3229190000	-0.2102680000
H	3.0258050000	1.4542650000	1.0192180000

2,2,4,5-Tetramethylhexane

0 1

C	-0.7814060000	0.5555630000	0.4240600000
C	1.7372980000	-0.1568860000	-0.0922100000
C	0.2841610000	0.0779610000	-0.5978420000
C	-2.1629670000	-0.1433720000	0.2492760000
C	-0.9411240000	2.0852710000	0.4092520000
C	2.6059090000	-0.5037420000	-1.3175190000
C	1.7834000000	-1.3425210000	0.8912350000
C	2.3269760000	1.0915060000	0.5901730000
C	-2.7721960000	0.0192750000	-1.1522450000
C	-2.1209200000	-1.6245790000	0.6507710000
H	-0.4400880000	0.2762800000	1.4373060000
H	-0.0407270000	-0.8731180000	-1.0526330000
H	0.3243620000	0.8014700000	-1.4324120000
H	-2.8441670000	0.3612520000	0.9606560000
H	0.0091950000	2.5963700000	0.6216640000
H	-1.6736250000	2.4167730000	1.1643940000
H	-1.2898210000	2.4447290000	-0.5734140000
H	2.2179460000	-1.3924980000	-1.8428520000
H	3.6461120000	-0.7175120000	-1.0199960000
H	2.6272480000	0.3296440000	-2.0394880000
H	1.1795620000	-1.1556350000	1.7934880000
H	2.8178380000	-1.5347660000	1.2220120000
H	1.4056760000	-2.2656140000	0.4204010000
H	2.3065180000	1.9653630000	-0.0821070000
H	3.3779770000	0.9149350000	0.8747570000
H	1.7797890000	1.3569720000	1.5085580000
H	-2.1730280000	-0.4979380000	-1.9205930000
H	-3.7858770000	-0.4128760000	-1.1847210000
H	-2.8566120000	1.0764080000	-1.4494770000
H	-1.7411550000	-1.7539360000	1.6777540000
H	-1.4742430000	-2.2149380000	-0.0204420000
H	-3.1276600000	-2.0722290000	0.6074390000

3-Ethyl-2,2,4-trimethylpentane

0 1

C	-0.1896040000	0.2453020000	-0.3561640000
C	1.1242430000	-0.6102530000	-0.0953660000
C	-1.5381530000	-0.5431370000	-0.2407640000
C	-0.2757910000	1.5730430000	0.4398540000
C	2.2623680000	-0.0830010000	-1.0014510000
C	0.9331730000	-2.0958420000	-0.4728650000
C	1.6043750000	-0.5513710000	1.3702930000
C	-2.6788720000	0.1607850000	-0.9980630000
C	-1.9853180000	-0.8717200000	1.1929300000
C	0.7126130000	2.6891840000	0.0781950000
H	-0.1333890000	0.5244850000	-1.4255550000
H	-1.3921780000	-1.5014310000	-0.7614660000
H	-0.2079060000	1.3699120000	1.5213670000
H	-1.2881230000	1.9808270000	0.2872160000
H	3.1865030000	-0.6619590000	-0.8345600000
H	2.4975160000	0.9734550000	-0.8163970000
H	1.9951770000	-0.1844340000	-2.0667250000
H	0.2263280000	-2.6175620000	0.1899630000
H	0.5776870000	-2.2124410000	-1.5099560000
H	1.8992790000	-2.6219240000	-0.3980800000

H	0.8357580000	-0.9016330000	2.0762600000
H	2.4897120000	-1.1957190000	1.5023150000
H	1.8963600000	0.4660200000	1.6714890000
H	-2.3945510000	0.3838980000	-2.0398750000
H	-2.9812450000	1.1087770000	-0.5233020000
H	-3.5735960000	-0.4829590000	-1.0276140000
H	-2.1957110000	0.0390100000	1.7776410000
H	-2.9133110000	-1.4675050000	1.1768600000
H	-1.2323450000	-1.4552360000	1.7440890000
H	1.7435110000	2.4703530000	0.3952640000
H	0.7313740000	2.8768260000	-1.0088690000
H	0.4189520000	3.6313030000	0.5703480000

2,2,4-Trimethylheptane

0 1

C	2.1000140000	-0.2182430000	0.0176240000
C	0.6375070000	-0.5022720000	-0.4399230000
C	-0.5533440000	0.2874280000	0.1517700000
C	-1.8683280000	-0.3636250000	-0.3343660000
C	2.7002990000	1.0273960000	-0.6658910000
C	2.1896960000	-0.0671970000	1.5474570000
C	2.9499300000	-1.4354690000	-0.4061360000
C	-0.5321720000	1.7902330000	-0.1662070000
C	-3.1414840000	0.0829520000	0.3946850000
C	-4.3922610000	-0.6567250000	-0.0881340000
H	0.6001650000	-0.4193420000	-1.5432180000
H	0.4399630000	-1.5670110000	-0.2199770000
H	-0.5271450000	0.1714950000	1.2516680000
H	-1.9831060000	-0.1770430000	-1.4201170000
H	-1.7783180000	-1.4602650000	-0.2274470000
H	2.6453150000	0.9399550000	-1.7640490000
H	2.1900130000	1.9571670000	-0.3787610000
H	3.7639740000	1.1392300000	-0.3951910000
H	3.2418240000	0.0166500000	1.8675480000
H	1.7545040000	-0.9403890000	2.0625750000
H	1.6638630000	0.8314330000	1.9072640000
H	2.8846330000	-1.6068640000	-1.4938630000
H	2.6116730000	-2.3556630000	0.0987640000
H	4.0127230000	-1.2835220000	-0.1542460000
H	0.3015070000	2.3086070000	0.3287610000
H	-0.4376350000	1.9635510000	-1.2525680000
H	-1.4572060000	2.2837540000	0.1703160000
H	-3.2953890000	1.1685440000	0.2678080000
H	-3.0130390000	-0.0812620000	1.4808830000
H	-4.2968530000	-1.7460770000	0.0600500000
H	-4.5675900000	-0.4857280000	-1.1641810000
H	-5.2930160000	-0.3240960000	0.4527050000

2,2,5,5-Tetramethylhexane

0 1

C	2.0192240000	-0.0043380000	0.0000000000
C	-2.0192240000	0.0043390000	0.0000000000
C	0.5582610000	0.5262750000	-0.0000280000
C	-0.5582590000	-0.5262710000	-0.0000180000
C	2.3030180000	-0.8471790000	1.2582890000
C	2.3030220000	-0.8473030000	-1.2582050000
C	2.9603920000	1.2158930000	-0.0000590000
C	-2.9603880000	-1.2158950000	-0.0000280000
C	-2.3030270000	0.8472720000	-1.2582260000
C	-2.3030180000	0.8472080000	1.2582710000
H	0.4443420000	1.1814020000	0.8816460000
H	0.4443610000	1.1813610000	-0.8817340000
H	-0.4443560000	-1.1813720000	-0.8817130000
H	-0.4443390000	-1.1813830000	0.8816670000
H	1.6984990000	-1.7676100000	1.2846270000
H	3.3630500000	-1.1497710000	1.2936780000
H	2.0883560000	-0.2761880000	2.1776370000
H	1.6985190000	-1.7677480000	-1.2844420000
H	3.3630590000	-1.1498800000	-1.2935750000
H	2.0883390000	-0.2764120000	-2.1776110000
H	2.7960840000	1.8457070000	0.8902950000
H	2.7960970000	1.8456100000	-0.8904830000
H	4.0178310000	0.9031700000	-0.0000340000
H	-4.0178280000	-0.9031750000	-0.0000060000
H	-2.7960750000	-1.8456890000	0.8903380000
H	-2.7960950000	-1.8456310000	-0.8904390000

H	-1.6985330000	1.7677220000	-1.2844830000
H	-2.0883390000	0.2763610000	-2.1776180000
H	-3.3630670000	1.1498390000	-1.2936040000
H	-1.6985000000	1.7676400000	1.2845860000
H	-3.3630510000	1.1498000000	1.2936550000
H	-2.0883550000	0.2762390000	2.1776320000

2,2,5-Trimethylheptane

0 1

C	2.0576210000	-0.0485210000	0.0048460000
C	0.6120400000	-0.1335020000	-0.5568820000
C	-0.4812280000	0.6219680000	0.2101430000
C	-1.8620560000	0.6303980000	-0.4799770000
C	-2.4572340000	-0.7762530000	-0.7236820000
C	2.9662680000	-0.9020500000	-0.9009110000
C	2.5731890000	1.4033750000	-0.0072530000
C	2.1228230000	-0.6049050000	1.4398920000
C	-2.8364320000	1.5370660000	0.2871470000
C	-2.6846170000	-1.6360380000	0.5251340000
H	0.3418460000	-1.2027560000	-0.6201220000
H	0.6297800000	0.2338940000	-1.6004450000
H	-0.1737050000	1.6730130000	0.3484510000
H	-0.5871170000	0.2064960000	1.2278930000
H	-1.7191390000	1.0803000000	-1.4816880000
H	-3.4189820000	-0.6501730000	-1.2531860000
H	-1.8062320000	-1.3277170000	-1.4238820000
H	4.0125890000	-0.8758980000	-0.5534840000
H	2.9487350000	-0.5364030000	-1.9413720000
H	2.6421700000	-1.9563010000	-0.9114200000
H	1.9954560000	2.0536530000	0.6686350000
H	2.5200840000	1.8373600000	-1.0201940000
H	3.6259970000	1.4454760000	0.3185510000
H	1.7449220000	-1.6405460000	1.4839070000
H	3.1622240000	-0.6135350000	1.8087520000
H	1.5291000000	0.0006920000	2.1428580000
H	-3.8204960000	1.5807820000	-0.2085860000
H	-2.4492480000	2.5671580000	0.3537950000
H	-2.9982960000	1.1829320000	1.3191970000
H	-3.1353690000	-2.6042320000	0.2526130000
H	-3.3643380000	-1.1506460000	1.2444290000
H	-1.7422260000	-1.8527840000	1.0545570000

2,2,6-Trimethylheptane

0 1

C	-2.2664230000	0.0000000000	-0.0643550000
C	-0.9702170000	-0.0000040000	0.7886340000
C	0.3709040000	0.0000050000	0.0421480000
C	1.5649070000	-0.0000100000	1.0083280000
C	2.9716780000	-0.0000010000	0.3692130000
C	-2.3431360000	1.2580470000	-0.9504770000
C	-2.3431440000	-1.2580500000	-0.9504720000
C	-3.4671790000	0.0000050000	0.9016810000
C	3.2484840000	1.2663170000	-0.4542970000
C	3.2484960000	-1.2663090000	-0.4543090000
H	-1.0008070000	0.8812870000	1.4568350000
H	-1.0008060000	-0.8813050000	1.4568210000
H	0.4298360000	-0.8827180000	-0.6167900000
H	0.4298380000	0.8827490000	-0.6167620000
H	1.4840690000	-0.8817700000	1.6712260000
H	1.4840700000	0.8817330000	1.6712500000
H	3.6861840000	-0.0000030000	1.2141160000
H	-2.2675060000	2.1774500000	-0.3453730000
H	-3.3015710000	1.2944190000	-1.4949090000
H	-1.5377010000	1.2819490000	-1.7015690000
H	-1.5376990000	-1.2819680000	-1.7015520000
H	-2.2675360000	-2.1774510000	-0.3453620000
H	-3.3015710000	-1.2944110000	-1.4949170000
H	-4.4219990000	-0.0000040000	0.3498160000
H	-3.4555840000	-0.8902630000	1.5527570000
H	-3.4555910000	0.8902850000	1.5527410000
H	2.6135660000	1.3154230000	-1.3550490000
H	4.2973640000	1.2944340000	-0.7931990000
H	3.0631260000	2.1795490000	0.1358240000
H	2.6135820000	-1.3154100000	-1.3550640000
H	4.2973770000	-1.2944150000	-0.7932100000
H	3.0631430000	-2.1795480000	0.1358010000

3-Ethyl-2,2-dimethylhexane

0 1

C	-0.0923600000	0.0200070000	0.3172860000
C	1.3512450000	-0.5461900000	0.0209180000
C	-1.0921450000	-0.2698610000	-0.8270050000
C	-0.1679950000	1.4974940000	0.7862520000
C	1.3190540000	-2.0845100000	0.1647490000
C	2.3677160000	-0.0152650000	1.0521260000
C	1.8622710000	-0.2086320000	-1.3960470000
C	-2.5824390000	-0.0568000000	-0.5023320000
C	0.1824840000	2.6027620000	-0.2186260000
C	-3.1370040000	-0.9623290000	0.6020480000
H	-0.4313840000	-0.5614430000	1.1945390000
H	-0.9678580000	-1.3145850000	-1.1598620000
H	-0.8402740000	0.3509860000	-1.7031100000
H	-1.1991200000	1.6726020000	1.1387850000
H	0.4582740000	1.6212360000	1.6840780000
H	0.6061960000	-2.5545690000	-0.5304970000
H	2.3125750000	-2.5137420000	-0.0465960000
H	1.0364630000	-2.3827310000	1.1882790000
H	2.0277460000	-0.1988450000	2.0853010000
H	3.3370050000	-0.5260050000	0.9276370000
H	2.5509990000	1.0647980000	0.9435520000
H	1.8930980000	0.8746580000	-1.5827320000
H	1.2375340000	-0.6660980000	-2.1786960000
H	2.8864430000	-0.5962140000	-1.5296220000
H	-2.7703010000	0.9994800000	-0.2401190000
H	-3.1562270000	-0.2355520000	-1.4290500000
H	1.2492690000	2.6011120000	-0.4916250000
H	-0.3979250000	2.5195500000	-1.1518810000
H	-0.0381680000	3.5928410000	0.2136800000
H	-2.6731440000	-0.7578710000	1.5809290000
H	-2.9622800000	-2.0274110000	0.3701290000
H	-4.2238820000	-0.8222650000	0.7206580000

4-Ethyl-2,2-dimethylhexane

0 1

C	-1.8271290000	-0.0235580000	0.0195040000
C	0.8351500000	-0.0338290000	-0.4759640000
C	-0.4464270000	-0.7511200000	0.0141630000
C	1.2724510000	1.1520110000	0.4097220000
C	1.9667440000	-1.0719140000	-0.6906060000
C	-2.9131050000	-1.1201290000	0.0679080000
C	-2.0350960000	0.8129930000	-1.2562130000
C	-2.0186660000	0.8666960000	1.2647320000
C	2.5585350000	1.8562560000	-0.0385910000
C	2.5117660000	-1.7507890000	0.5719940000
H	0.6215510000	0.3779670000	-1.4799940000
H	-0.5631920000	-1.6398810000	-0.6321040000
H	-0.2762370000	-1.1482890000	1.0313310000
H	1.3795310000	0.8164010000	1.4574980000
H	0.4642620000	1.8994370000	0.4195110000
H	2.8019900000	-0.5853450000	-1.2219190000
H	1.5916390000	-1.8481840000	-1.3814070000
H	-3.9209280000	-0.6769720000	0.1319440000
H	-2.8809750000	-1.7569580000	-0.8317750000
H	-2.7786700000	-1.7738320000	0.9462810000
H	-1.3420720000	1.6675750000	-1.3126760000
H	-1.8904440000	0.2026940000	-2.1637950000
H	-3.0599570000	1.2193670000	-1.2895700000
H	-1.3226260000	1.7167650000	1.2932880000
H	-1.8747050000	0.2852930000	2.1910230000
H	-3.0414620000	1.2796070000	1.2882530000
H	2.7389530000	2.7614200000	0.5638210000
H	3.4482700000	1.2147470000	0.0625020000
H	2.4957600000	2.1700680000	-1.0951460000
H	1.7310280000	-2.3096630000	1.1127290000
H	2.9459910000	-1.0230960000	1.2773660000
H	3.3069560000	-2.4688210000	0.3129530000

2,2-Dimethyloctane

0 1

C	-2.6098300000	-0.0237760000	0.0000010000
C	-1.2237340000	0.6740090000	-0.0000070000
C	0.0207080000	-0.2227870000	-0.0000060000
C	1.3288570000	0.5777800000	-0.0000070000
C	-2.7890430000	-0.8941430000	-1.2584770000
C	-2.7890380000	-0.8941070000	1.2585020000
C	-3.6916850000	1.0735520000	-0.0000140000
C	2.5865770000	-0.2970020000	0.0000040000
C	3.8944250000	0.5017940000	0.0000030000
C	5.1444720000	-0.3811590000	-0.0000010000
H	-1.1764230000	1.3408230000	-0.8815310000
H	-1.1764200000	1.3408320000	0.8815090000
H	0.0097090000	-0.8870530000	0.8825140000
H	0.0097070000	-0.8870520000	-0.8825270000
H	1.3486280000	1.2453360000	-0.8822840000
H	1.3486210000	1.2453480000	0.8822630000
H	-3.8036490000	-1.3251830000	-1.2932410000
H	-2.0753010000	-1.7328280000	-1.2867540000
H	-2.6476050000	-0.2998790000	-2.1772680000
H	-3.8036390000	-1.3251620000	1.2932750000
H	-2.6476150000	-0.2998150000	2.1772770000
H	-2.0752860000	-1.7327830000	1.2868080000
H	-3.6059640000	1.7188340000	0.8903970000
H	-3.6059720000	1.7188030000	-0.8904470000
H	-4.7033010000	0.6343860000	-0.0000010000
H	2.5679390000	-0.9646340000	0.8825170000
H	2.5679440000	-0.9646500000	-0.8824960000
H	3.9128950000	1.1684410000	0.8821190000
H	3.9128920000	1.1684410000	-0.8821140000
H	5.1727570000	-1.0344500000	0.8889260000
H	5.1727980000	-1.0343870000	-0.8889720000
H	6.0670260000	0.2216620000	0.0000420000

2,3,3,4-Tetramethylhexane

0 1

C	-0.4720190000	0.5222270000	0.1027370000
C	1.0116470000	0.2059250000	-0.3322840000
C	-1.3288320000	-0.8004600000	0.0526440000
C	-1.0884860000	1.5763550000	-0.8423900000
C	-0.4826820000	1.1075850000	1.5323590000
C	1.7957690000	-0.6348830000	0.7047830000
C	1.8258910000	1.4601480000	-0.7003940000
C	-1.5888200000	-1.3246690000	-1.3713140000
C	-2.6747310000	-0.7063630000	0.7938860000
C	2.9743230000	-1.4145090000	0.1109060000
H	0.9435910000	-0.4025280000	-1.2517140000
H	-0.7416920000	-1.5741680000	0.5778420000
H	-0.9477550000	1.3188350000	-1.9039040000
H	-2.1708180000	1.6783850000	-0.6645520000
H	-0.6431840000	2.5694200000	-0.6802520000
H	-0.2329590000	0.3535290000	2.2951650000
H	-1.4693250000	1.5232120000	1.7873980000
H	0.2443140000	1.9298040000	1.6270390000
H	1.1246520000	-1.3559670000	1.1984900000
H	2.1652950000	0.0282760000	1.5075780000
H	1.8641090000	2.1820680000	0.1326450000
H	1.4217330000	1.9821960000	-1.5795110000
H	2.8659510000	1.1892170000	-0.9400110000
H	-2.0436500000	-2.3277360000	-1.3262160000
H	-2.2901860000	-0.6760260000	-1.9215770000
H	-0.6719940000	-1.4114480000	-1.9740160000
H	-3.2240790000	-1.6570480000	0.6959460000
H	-3.3220200000	0.0836370000	0.3765500000
H	-2.5546130000	-0.5096760000	1.8689700000
H	3.7117660000	-0.7530500000	-0.3722990000
H	2.6311630000	-2.1365020000	-0.6498600000
H	3.5073400000	-1.9825730000	0.8906920000

2,3,3,5-Tetramethylhexane

0 1

C	0.6545910000	-0.4723920000	0.4226210000
C	1.2889590000	0.5157600000	-0.6183010000
C	-0.7989010000	-0.0430050000	0.7995130000

C	-1.8596470000	0.2799820000	-0.2789200000
C	1.4521230000	-0.4892640000	1.7463900000
C	0.6767710000	-1.9122040000	-0.1389580000
C	1.3135160000	1.9821750000	-0.1545180000
C	2.6994060000	0.1123880000	-1.0837250000
C	-2.3519220000	-0.9341590000	-1.0811150000
C	-3.0587180000	0.9674440000	0.3969570000
H	0.6407990000	0.4686410000	-1.5128770000
H	-0.7169540000	0.8508090000	1.4411870000
H	-1.2139700000	-0.8289610000	1.4589630000
H	-1.4304740000	1.0056780000	-0.9932710000
H	2.4969800000	-0.8010410000	1.5918630000
H	0.9996270000	-1.2050490000	2.4528480000
H	1.4596960000	0.4950380000	2.2400040000
H	0.3197840000	-1.9652640000	-1.1779450000
H	0.0406930000	-2.5766400000	0.4683000000
H	1.6943250000	-2.3325540000	-0.1218810000
H	1.6942930000	2.6288500000	-0.9620420000
H	0.3179140000	2.3592790000	0.1232600000
H	1.9792800000	2.1232160000	0.7131540000
H	2.7272740000	-0.8951320000	-1.5241680000
H	3.0573110000	0.8151840000	-1.8539620000
H	3.4292610000	0.1398740000	-0.2574750000
H	-1.5698650000	-1.3693380000	-1.7187270000
H	-2.7184020000	-1.7305000000	-0.4096430000
H	-3.1883200000	-0.6472980000	-1.7403000000
H	-3.5330310000	0.2955660000	1.1340940000
H	-3.8282580000	1.2460760000	-0.3415010000
H	-2.7550360000	1.8832180000	0.9304530000

2,3,3-Trimethylheptane

0 1

C	-0.8379080000	0.5877680000	0.0000060000
C	-2.2014170000	-0.1985060000	0.0000010000
C	0.3661440000	-0.3928690000	-0.0000050000
C	-0.7879350000	1.4984270000	-1.2464050000
C	-0.7879310000	1.4984070000	1.2464310000
C	1.7716440000	0.2259880000	-0.0000020000
C	-2.4421560000	-1.0588320000	1.2525140000
C	-2.4421650000	-1.0588020000	-1.2525300000
C	2.8829280000	-0.8307730000	-0.0000040000
C	4.2905430000	-0.2300570000	-0.0000040000
H	-2.9802160000	0.5883980000	0.0000140000
H	0.2854040000	-1.0537650000	-0.8811030000
H	0.2854070000	-1.0537780000	0.8810840000
H	0.0588590000	2.2001700000	-1.1951720000
H	-0.6820550000	0.9241140000	-2.1798600000
H	-1.7073200000	2.1029270000	-1.3262850000
H	0.0588680000	2.2001460000	1.1952100000
H	-0.6820550000	0.9240770000	2.1798770000
H	-1.7073120000	2.1029110000	1.3263210000
H	1.9018950000	0.8773650000	-0.8827130000
H	1.9018940000	0.8773600000	0.8827130000
H	-1.7344610000	-1.9022280000	1.3161030000
H	-3.4554870000	-1.4918320000	1.2218270000
H	-2.3631710000	-0.4819870000	2.1857190000
H	-2.3631930000	-0.4819330000	-2.1857220000
H	-1.7344670000	-1.9021930000	-1.3161490000
H	-3.4554940000	-1.4918060000	-1.2218450000
H	2.7622410000	-1.4869370000	-0.8820320000
H	2.7622420000	-1.4869390000	0.8820220000
H	5.0655590000	-1.0135870000	-0.0000020000
H	4.4561920000	0.4025070000	-0.8889670000
H	4.4561910000	0.4025120000	0.8889550000

2,3,4,4-Tetramethylhexane

0 1

C	-1.0059680000	-0.4407900000	0.1607330000
C	0.3425420000	-0.0979110000	-0.5764540000
C	1.4712810000	0.4811700000	0.3408640000
C	-1.6447880000	0.8322060000	0.7928290000
C	-0.8153000000	-1.4615790000	1.3012860000
C	-2.0124980000	-1.0430590000	-0.8454300000
C	0.8647120000	-1.2415010000	-1.4661250000
C	2.2778130000	1.5559980000	-0.4081820000
C	2.4376460000	-0.5603690000	0.9329940000

C	-2.0149110000	1.9835760000	-0.1490170000
H	0.0794170000	0.7197000000	-1.2696960000
H	0.9850590000	0.9884180000	1.1908820000
H	-2.5587610000	0.5050290000	1.3210480000
H	-0.9797760000	1.2201480000	1.5832110000
H	-0.3231350000	-2.3836940000	0.9538270000
H	-0.2196880000	-1.0487350000	2.1302390000
H	-1.7945990000	-1.7512790000	1.7177670000
H	-2.1024360000	-0.4355560000	-1.7603960000
H	-1.7246210000	-2.0604940000	-1.1506700000
H	-3.0145880000	-1.1110210000	-0.3897290000
H	0.1687170000	-1.4685240000	-2.2862970000
H	1.0324250000	-2.1742570000	-0.9041650000
H	1.8243370000	-0.9655570000	-1.9315400000
H	3.0668300000	1.9809070000	0.2344100000
H	1.6346730000	2.3863430000	-0.7430920000
H	2.7735120000	1.1376750000	-1.3015130000
H	3.0685240000	-1.0197020000	0.1544570000
H	3.1169800000	-0.0764620000	1.6543530000
H	1.9183100000	-1.3720340000	1.4619930000
H	-1.1337790000	2.4225060000	-0.6436550000
H	-2.5048890000	2.7935310000	0.4156470000
H	-2.7156860000	1.6666200000	-0.9380680000

2,3,4,5-Tetramethylhexane

0 1

C	-0.7121930000	0.5697290000	0.3208760000
C	0.7121950000	0.5697310000	-0.3208720000
C	-1.5412180000	-0.7196750000	0.0407780000
C	1.5412170000	-0.7196770000	-0.0407890000
C	-1.4874840000	1.8447530000	-0.0636330000
C	1.4874890000	1.8447480000	0.0636520000
C	-1.8900000000	-0.9263910000	-1.4413220000
C	-2.8105170000	-0.7932840000	0.9055780000
C	1.8899860000	-0.9264200000	1.4413100000
C	2.8105250000	-0.7932690000	-0.9055790000
H	-0.5705000000	0.6008500000	1.4182640000
H	0.5705020000	0.6008660000	-1.4182600000
H	-0.9182860000	-1.5747240000	0.3526340000
H	0.9182880000	-1.5747210000	-0.3526670000
H	-1.5307550000	1.9806180000	-1.1576620000
H	-1.0219660000	2.7459580000	0.3602510000
H	-2.5223090000	1.8172520000	0.3089570000
H	2.5223150000	1.8172490000	-0.3089370000
H	1.5307610000	1.9806000000	1.1576830000
H	1.0219740000	2.7459600000	-0.3602210000
H	-2.5449290000	-0.1239850000	-1.8201490000
H	-2.4267580000	-1.8790050000	-1.5832360000
H	-0.9938770000	-0.9550980000	-2.0816120000
H	-3.2846640000	-1.7842400000	0.8118870000
H	-3.5643370000	-0.0459340000	0.6079900000
H	-2.5824640000	-0.6324070000	1.9726290000
H	0.9938570000	-0.9551390000	2.0815910000
H	2.4267430000	-1.8790360000	1.5832120000
H	2.5449100000	-0.1240200000	1.8201570000
H	3.5643410000	-0.0459240000	-0.6079690000
H	2.5824820000	-0.6323720000	-1.9726290000
H	3.2846720000	-1.7842260000	-0.8119020000

3-Ethyl-2,3,4-trimethylpentane

0 1

C	0.0429070000	0.0394170000	0.4538360000
C	-1.1655310000	-0.8711380000	-0.0100060000
C	1.3069480000	-0.2194920000	-0.4476910000
C	-0.3177000000	1.5562370000	0.4706660000
C	0.3628380000	-0.3349430000	1.9211180000
C	-1.3906610000	-1.0022450000	-1.5293770000
C	-2.5055490000	-0.5037560000	0.6544290000
C	2.5033380000	0.7029500000	-0.1482590000
C	1.7974730000	-1.6795180000	-0.4255290000
C	-0.6242120000	2.2691020000	-0.8526510000
H	-0.9167580000	-1.8863240000	0.3486220000
H	1.0020620000	0.0005060000	-1.4842420000
H	0.5137390000	2.0868980000	0.9634590000
H	-1.1762320000	1.6960250000	1.1481050000
H	-0.4834680000	-0.1023200000	2.5853630000

H	0.5828440000	-1.4074290000	2.0344250000
H	1.2283470000	0.2282550000	2.3028860000
H	-0.5205310000	-1.4151280000	-2.0597760000
H	-1.6444920000	-0.0423620000	-2.0032120000
H	-2.2345640000	-1.6874340000	-1.7157040000
H	-2.9152580000	0.4416960000	0.2621870000
H	-3.2531810000	-1.2877080000	0.4503380000
H	-2.4274720000	-0.4068200000	1.7477260000
H	3.3390390000	0.4587790000	-0.8247070000
H	2.8773010000	0.5786420000	0.8815150000
H	2.2702080000	1.7677510000	-0.2945120000
H	1.0003370000	-2.4048750000	-0.6446790000
H	2.5877490000	-1.8236980000	-1.1805720000
H	2.2321910000	-1.9482590000	0.5511770000
H	0.1751130000	2.1401450000	-1.5999310000
H	-1.5628430000	1.9214970000	-1.3093750000
H	-0.7332400000	3.3524800000	-0.6782980000

2,3,4-Trimethylheptane

0 1

C	0.6222640000	-0.3450770000	-0.1055800000
C	-0.3252270000	0.7668120000	0.4339250000
C	2.0789730000	0.1538780000	-0.3520800000
C	-1.7758280000	0.2794180000	0.6782970000
C	0.5816820000	-1.5998580000	0.7848950000
C	-0.3246800000	2.0307930000	-0.4450630000
C	-2.5506480000	-0.2251990000	-0.5466160000
C	2.8283470000	0.5462940000	0.9305720000
C	2.9045650000	-0.8541890000	-1.1677450000
C	-3.9869850000	-0.6302200000	-0.2025120000
H	0.2412790000	-0.6351140000	-1.1035110000
H	0.0550370000	1.0627900000	1.4297830000
H	2.0009680000	1.0589190000	-0.9776300000
H	-2.3382310000	1.1209460000	1.1238040000
H	-1.7772260000	-0.5103570000	1.4488860000
H	0.7869320000	-1.3559540000	1.8410270000
H	-0.4010660000	-2.0927910000	0.7491610000
H	1.3239100000	-2.3475260000	0.4667120000
H	-0.5179920000	1.7950150000	-1.5053890000
H	-1.1077710000	2.7329150000	-0.1142700000
H	0.6325560000	2.5710610000	-0.3988480000
H	-2.5703730000	0.5556800000	-1.3270740000
H	-2.0296950000	-1.0895940000	-0.9954850000
H	3.8283750000	0.9425920000	0.6885630000
H	2.2980990000	1.3230180000	1.5045160000
H	2.9730910000	-0.3209770000	1.5962010000
H	3.8714490000	-0.4146580000	-1.4635740000
H	3.1279590000	-1.7705510000	-0.5966170000
H	2.3782620000	-1.1539540000	-2.0893830000
H	-4.5295280000	-0.9961040000	-1.0892450000
H	-4.5552940000	0.2215890000	0.2089990000
H	-4.0055170000	-1.4328510000	0.5548110000

2,3,5-Trimethylheptane

0 1

C	1.0239100000	0.4274880000	0.6279410000
C	-0.4705940000	0.0698800000	0.7920710000
C	-1.4415370000	0.4929800000	-0.3292470000
C	1.6615240000	-0.1304930000	-0.6768140000
C	-2.8784920000	0.0062080000	-0.0437640000
C	1.7831090000	0.0206540000	1.9020500000
C	-1.4394830000	2.0127630000	-0.5489230000
C	1.6932690000	-1.6649410000	-0.7520940000
C	3.0631190000	0.4485340000	-0.9248560000
C	-3.0740600000	-1.5130360000	-0.0481590000
H	1.0980690000	1.5280810000	0.5525590000
H	-0.5529610000	-1.0192080000	0.9489600000
H	-0.8242880000	0.5291630000	1.7350300000
H	-1.1217440000	0.0128710000	-1.2738220000
H	1.0266050000	0.2227930000	-1.5092560000
H	-3.5477640000	0.4485180000	-0.8032620000
H	-3.2110140000	0.4198090000	0.9274890000
H	1.7272600000	-1.0659790000	2.0823990000
H	2.8473310000	0.2991500000	1.8599330000
H	1.3491080000	0.5184780000	2.7845910000
H	-0.4493670000	2.3914220000	-0.8464990000

H	-1.7354610000	2.5440000000	0.3731960000
H	-2.1526140000	2.2989640000	-1.3394360000
H	2.3413340000	-2.0981890000	0.0281530000
H	0.6919130000	-2.1100430000	-0.6403010000
H	2.0921070000	-1.9945170000	-1.7256890000
H	3.7954510000	0.0868340000	-0.1840280000
H	3.4364990000	0.1564530000	-1.9202750000
H	3.0588620000	1.5505570000	-0.8800290000
H	-2.5111340000	-2.0114610000	0.7571770000
H	-4.1362630000	-1.7730460000	0.0891370000
H	-2.7465260000	-1.9548750000	-1.0052570000

2,3,6-Trimethylheptane

0 1

C	1.1559450000	0.7685590000	-0.0472400000
C	-0.3141570000	0.4787420000	0.3298930000
C	1.9724420000	-0.5015330000	-0.4210810000
C	-1.1549160000	-0.1902290000	-0.7667640000
C	-2.6678090000	-0.3284200000	-0.4756310000
C	1.8128210000	1.6129960000	1.0570100000
C	3.3356920000	-0.1489880000	-1.0365830000
C	2.1469840000	-1.4944750000	0.7384850000
C	-3.3821670000	1.0265300000	-0.3645290000
C	-2.9559480000	-1.2068220000	0.7508080000
H	1.1317830000	1.3908140000	-0.9635760000
H	-0.7771850000	1.4434800000	0.6008980000
H	-0.3409400000	-0.1306520000	1.2506820000
H	1.3990940000	-1.0188430000	-1.2110530000
H	-0.7593870000	-1.1999530000	-0.9741550000
H	-1.0338870000	0.3814690000	-1.7061600000
H	-3.0960070000	-0.8471420000	-1.3540680000
H	2.8529750000	1.8806080000	0.8155540000
H	1.8190520000	1.0839480000	2.0247730000
H	1.2579060000	2.5538170000	1.2052660000
H	3.2292020000	0.5642940000	-1.8710240000
H	3.8320100000	-1.0513810000	-1.4300710000
H	4.0188180000	0.3009550000	-0.2969180000
H	2.6688430000	-2.4024410000	0.3936830000
H	2.7487390000	-1.0640570000	1.5566150000
H	1.1821770000	-1.8124830000	1.1648300000
H	-4.4731540000	0.8896390000	-0.2815500000
H	-3.1908420000	1.6566470000	-1.2493400000
H	-3.0569660000	1.5911830000	0.5254190000
H	-2.4525850000	-2.1853320000	0.6736720000
H	-2.6155930000	-0.7281520000	1.6844880000
H	-4.0373670000	-1.3945740000	0.8558150000

3-Ethyl-2,3-dimethylhexane

0 1

C	0.4047540000	0.3137900000	0.2627980000
C	1.2165130000	-0.8180020000	-0.4719120000
C	-1.0308590000	-0.1746090000	0.6085100000
C	0.3467860000	1.5592520000	-0.6715010000
C	1.0866490000	0.7054910000	1.5909460000
C	-1.9262540000	-0.6353720000	-0.5496760000
C	2.6749290000	-0.4429450000	-0.7942820000
C	1.2070130000	-2.1756030000	0.2524350000
C	-0.5456980000	2.7259830000	-0.2306370000
C	-3.3230370000	-1.0478500000	-0.0747680000
H	0.7100390000	-0.9689850000	-1.4428440000
H	-0.9585500000	-0.9930000000	1.3456930000
H	-1.5490380000	0.6399080000	1.1445460000
H	1.3713590000	1.9462460000	-0.7945180000
H	0.0305390000	1.2340930000	-1.6786380000
H	0.4891790000	1.4527180000	2.1364730000
H	1.1994460000	-0.1623030000	2.2598770000
H	2.0859380000	1.1389570000	1.4289520000
H	-1.4608290000	-1.4852450000	-1.0782560000
H	-2.0231620000	0.1698140000	-1.2990590000
H	3.1427520000	-1.2406780000	-1.3941840000
H	2.7604700000	0.4887060000	-1.3723980000
H	3.2808000000	-0.3294410000	0.1199710000
H	0.1939300000	-2.5819050000	0.3876000000
H	1.7787850000	-2.9164950000	-0.3301740000
H	1.6795020000	-2.1142870000	1.2471490000
H	-1.6139000000	2.4577950000	-0.2116200000

H	-0.4365810000	3.5707300000	-0.9302780000
H	-0.2787710000	3.0993470000	0.7712680000
H	-3.9548040000	-1.3799840000	-0.9144610000
H	-3.2704210000	-1.8770480000	0.6516080000
H	-3.8414640000	-0.2097580000	0.4218170000

4-Ethyl-2,3-dimethylhexane

0 1

C	-0.6173060000	0.3253180000	0.4477130000
C	0.8102650000	-0.0199960000	-0.0824860000
C	-1.7513810000	-0.3719010000	-0.3608950000
C	1.8530960000	1.0519190000	0.3084350000
C	1.2800170000	-1.4319310000	0.3356890000
C	-0.7352110000	0.0720700000	1.9623120000
C	-3.1383840000	-0.1726680000	0.2726080000
C	-1.8039820000	0.1057400000	-1.8216550000
C	1.7148660000	2.3878520000	-0.4289070000
C	2.3920960000	-2.0089980000	-0.5472470000
H	-0.7621000000	1.4107500000	0.2907330000
H	0.7614370000	-0.0154760000	-1.1855970000
H	-1.5465460000	-1.4598350000	-0.3667680000
H	2.8623370000	0.6527320000	0.1108860000
H	1.8140030000	1.2257860000	1.3990400000
H	0.4246620000	-2.1283830000	0.3104260000
H	1.6198980000	-1.4118780000	1.3867360000
H	-0.8007890000	-1.0037340000	2.1957530000
H	-1.6266590000	0.5560700000	2.3871190000
H	0.1350130000	0.4735610000	2.5046590000
H	-3.2198650000	-0.6306580000	1.2691870000
H	-3.9197050000	-0.6239710000	-0.3605200000
H	-3.3769880000	0.9012090000	0.3744710000
H	-2.6079500000	-0.4110170000	-2.3708610000
H	-2.0136680000	1.1890730000	-1.8714650000
H	-0.8674880000	-0.0768710000	-2.3691360000
H	2.5031110000	3.0933680000	-0.1193680000
H	0.7463450000	2.8768000000	-0.2344470000
H	1.8015710000	2.2516030000	-1.5206480000
H	2.6937000000	-3.0115630000	-0.2027370000
H	2.0591240000	-2.1038710000	-1.5951100000
H	3.2960990000	-1.3781270000	-0.5457480000

2,3-Dimethyloctane

0 1

C	-1.5182640000	0.6658650000	-0.3771230000
C	-2.0861830000	-0.7584850000	-0.1130440000
C	-0.1057110000	0.8905760000	0.2023290000
C	0.9903460000	-0.0539520000	-0.3057200000
C	-2.4527430000	1.7935370000	0.0900190000
C	2.3953040000	0.3529970000	0.1534350000
C	-2.3247570000	-1.0697740000	1.3725200000
C	-3.3577410000	-1.0319780000	-0.9310770000
C	3.5022000000	-0.5876820000	-0.3343230000
C	4.9017410000	-0.1607850000	0.1145750000
H	-1.4256940000	0.7525190000	-1.4776660000
H	-1.3225820000	-1.4690690000	-0.4771560000
H	-0.1435030000	0.8474320000	1.3067740000
H	0.1919640000	1.9279470000	-0.0383530000
H	0.7922770000	-1.0877040000	0.0296850000
H	0.9644670000	-0.0836040000	-1.4118450000
H	-2.5504640000	1.8122140000	1.1884440000
H	-3.4641200000	1.7014380000	-0.3348060000
H	-2.0558740000	2.7750640000	-0.2168830000
H	2.6100770000	1.3804960000	-0.1969880000
H	2.4193680000	0.4015320000	1.2589190000
H	-1.4147830000	-0.9360150000	1.9789400000
H	-2.6525530000	-2.1146950000	1.5001720000
H	-3.1111390000	-0.4273170000	1.8031400000
H	-3.6595830000	-2.0887250000	-0.8429720000
H	-4.2093820000	-0.4215550000	-0.5873050000
H	-3.2026090000	-0.8171090000	-2.0015930000
H	3.2947820000	-1.6121580000	0.0265720000
H	3.4701430000	-0.6445550000	-1.4382990000
H	4.9764820000	-0.1274330000	1.2150800000
H	5.6747600000	-0.8560520000	-0.2508510000
H	5.1528120000	0.8454310000	-0.2625510000

2,4,4-Trimethylheptane

0 1

C	0.2548950000	0.8367790000	0.0355470000
C	-1.1745420000	0.5657290000	-0.5309230000
C	1.2137650000	-0.3252160000	-0.3484000000
C	-2.1760450000	-0.3433520000	0.2195180000
C	0.7508320000	2.1419350000	-0.6251900000
C	0.2064650000	1.0471780000	1.5612070000
C	2.6629310000	-0.2440990000	0.1525200000
C	-1.7699900000	-1.8204450000	0.3335240000
C	-3.5491730000	-0.2391770000	-0.4656140000
C	3.5070070000	-1.4302300000	-0.3242480000
H	-1.6703960000	1.5486470000	-0.6242370000
H	-1.0693840000	0.1919190000	-1.5675720000
H	0.7892530000	-1.2779430000	0.0050480000
H	1.2283340000	-0.3980920000	-1.4523810000
H	-2.2942220000	0.0522890000	1.2448390000
H	0.0997940000	2.9901280000	-0.3558210000
H	1.7741920000	2.4000800000	-0.3113450000
H	0.7480410000	2.0557980000	-1.7251370000
H	-0.5528640000	1.8011070000	1.8288710000
H	1.1729120000	1.4081290000	1.9469040000
H	-0.0397060000	0.1187820000	2.1011610000
H	2.6794650000	-0.2127660000	1.2559770000
H	3.1363390000	0.6928670000	-0.1878490000
H	-2.5914500000	-2.4123570000	0.7709690000
H	-1.5432260000	-2.2507130000	-0.6576420000
H	-0.8880210000	-1.9691480000	0.9737290000
H	-3.5043410000	-0.6240800000	-1.4998030000
H	-4.3103870000	-0.8259600000	0.0741610000
H	-3.9004240000	0.8045490000	-0.5153090000
H	4.5422000000	-1.3652390000	0.0482020000
H	3.5509130000	-1.4740440000	-1.4260240000
H	3.0861070000	-2.3885600000	0.0256090000

2,4,5-Trimethylheptane

0 1

C	0.1580620000	-0.5842890000	0.4172340000
C	1.6712320000	-0.4423900000	0.0663760000
C	-0.7697100000	-0.4441040000	-0.8185960000
C	-2.1282050000	0.2767100000	-0.6256370000
C	2.0906230000	1.0011730000	-0.2830900000
C	-0.0950520000	-1.8960490000	1.1805500000
C	2.1308490000	-1.4092060000	-1.0374100000
C	-1.9597010000	1.7729590000	-0.3222540000
C	-3.0580360000	-0.3835530000	0.4022860000
C	2.0280720000	1.9976190000	0.8786430000
H	-0.0732990000	0.2384290000	1.1155390000
H	2.2228130000	-0.7148760000	0.9871650000
H	-0.9636790000	-1.4494920000	-1.2343430000
H	-0.2385370000	0.1047950000	-1.6148620000
H	-2.6352590000	0.2060680000	-1.6067750000
H	3.1273240000	0.9720700000	-0.6637830000
H	1.4792270000	1.3740340000	-1.1254020000
H	0.1241560000	-2.7772290000	0.5545560000
H	0.5405170000	-1.9607880000	2.0796570000
H	-1.1411060000	-1.9838140000	1.5068420000
H	1.8562160000	-2.4527800000	-0.8181780000
H	1.6953410000	-1.1483450000	-2.0164090000
H	3.2268610000	-1.3746220000	-1.1494730000
H	-1.4905050000	1.9414790000	0.6618870000
H	-2.9368990000	2.2836750000	-0.3071820000
H	-1.3333880000	2.2710610000	-1.0809080000
H	-4.0509670000	0.0962960000	0.3909510000
H	-3.2033650000	-1.4558510000	0.1917500000
H	-2.6650260000	-0.2909710000	1.4287390000
H	1.0030020000	2.1364010000	1.2581790000
H	2.6516490000	1.6621700000	1.7251950000
H	2.3961270000	2.9890620000	0.5682420000

2,4,6-Trimethylheptane

0 1

C	0.0899250000	0.5302640000	0.4404390000
C	0.8419710000	-0.0599000000	-0.7732030000

C	-1.3756640000	0.8573990000	0.0709310000
C	2.2834480000	-0.6084360000	-0.6007060000
C	-2.3014360000	-0.3052830000	-0.3416500000
C	0.7492440000	1.7902620000	1.0235710000
C	2.4282710000	-1.5437480000	0.6080920000
C	3.3854830000	0.4634830000	-0.6114580000
C	-2.4471770000	-1.3616270000	0.7625210000
C	-3.6777540000	0.2418340000	-0.7469310000
H	0.0729770000	-0.2323480000	1.2393810000
H	0.8500690000	0.6960240000	-1.5819680000
H	0.2352220000	-0.8969300000	-1.1586120000
H	-1.3674260000	1.6055940000	-0.7446310000
H	-1.8417100000	1.3651090000	0.9366310000
H	2.4570410000	-1.2254950000	-1.5027710000
H	-1.8718260000	-0.8008710000	-1.2315020000
H	0.1207990000	2.2254700000	1.8184240000
H	0.8867420000	2.5647420000	0.2487040000
H	1.7341950000	1.5816080000	1.4652120000
H	1.6611880000	-2.3363110000	0.6014130000
H	3.4152470000	-2.0351670000	0.6074270000
H	2.3357120000	-0.9978140000	1.5620340000
H	4.3803490000	-0.0083760000	-0.6789520000
H	3.2793840000	1.1383040000	-1.4771490000
H	3.3818620000	1.0849470000	0.2971300000
H	-1.4875100000	-1.8415910000	1.0114360000
H	-3.1441670000	-2.1591770000	0.4564060000
H	-2.8448050000	-0.9100700000	1.6890300000
H	-3.5950300000	0.9769540000	-1.5644950000
H	-4.1691730000	0.7454410000	0.1042660000
H	-4.3470050000	-0.5655260000	-1.0870500000

3-Ethyl-2,4-dimethylhexane

0 1

C	0.5166900000	0.2986140000	0.3897570000
C	-1.0183920000	0.0590740000	0.5399170000
C	1.3455770000	-0.9712350000	0.0071710000
C	0.8576540000	1.5180180000	-0.4955240000
C	-1.7235990000	-0.4623030000	-0.7299150000
C	-1.3285420000	-0.8223120000	1.7618380000
C	1.5122440000	-1.2185750000	-1.5030070000
C	2.7283600000	-0.9414270000	0.6795600000
C	0.4388480000	2.8727090000	0.0866690000
C	-3.2531500000	-0.3613660000	-0.6882110000
H	0.8511070000	0.5686830000	1.4097040000
H	-1.4559560000	1.0512010000	0.7561370000
H	0.8122140000	-1.8452330000	0.4207280000
H	1.9491920000	1.5383160000	-0.6573410000
H	0.4136490000	1.3999600000	-1.4991320000
H	-1.3653950000	0.1045490000	-1.6059890000
H	-1.4365890000	-1.5144580000	-0.9070320000
H	-2.4098650000	-0.8694830000	1.9629200000
H	-0.8440180000	-0.4264720000	2.6695450000
H	-0.9782710000	-1.8590690000	1.6226460000
H	0.5518830000	-1.2568810000	-2.0363310000
H	2.0234550000	-2.1806100000	-1.6738810000
H	2.1276080000	-0.4373680000	-1.9786480000
H	2.6419700000	-0.8911420000	1.7772710000
H	3.3127940000	-1.8423900000	0.4290220000
H	3.3181720000	-0.0675980000	0.3516690000
H	-0.6539210000	2.9662770000	0.1927020000
H	0.8823370000	3.0332180000	1.0845760000
H	0.7732780000	3.6990780000	-0.5614770000
H	-3.6966150000	-0.6939050000	-1.6408380000
H	-3.5800940000	0.6785470000	-0.5153480000
H	-3.6910720000	-0.9823990000	0.1095800000

3-Isopropyl-2,4-dimethylpentane

0 1

C	0.1653150000	-0.1127350000	-0.3776000000
C	1.6158670000	-0.2013110000	0.2095650000
C	-0.4059230000	1.3436030000	-0.3355260000
C	-0.7351570000	-1.2722880000	0.1713690000
C	2.5225890000	0.9759920000	-0.1941090000
C	2.3352820000	-1.4979800000	-0.2093590000
C	-1.7445150000	1.5170460000	-1.0716720000
C	-0.4821710000	2.0151470000	1.0495350000

C	-1.3755250000	-1.0408940000	1.5498670000
C	-1.7921000000	-1.7671450000	-0.8327970000
H	0.2828230000	-0.3271430000	-1.4586280000
H	1.5415400000	-0.2037570000	1.3137960000
H	0.3198340000	1.9340100000	-0.9197150000
H	-0.0529860000	-2.1267810000	0.3029200000
H	3.5467680000	0.8088290000	0.1772980000
H	2.5852990000	1.0671650000	-1.2930560000
H	2.1879490000	1.9439640000	0.2038700000
H	2.4426460000	-1.5462250000	-1.3075060000
H	1.8195780000	-2.4132650000	0.1148680000
H	3.3490250000	-1.5303550000	0.2220120000
H	-2.5871690000	1.0690160000	-0.5218870000
H	-1.9725050000	2.5897740000	-1.1910850000
H	-1.7204820000	1.0688510000	-2.0781040000
H	-1.3332230000	1.6542880000	1.6446790000
H	0.4285220000	1.8600880000	1.6477000000
H	-0.6121690000	3.1039380000	0.9285870000
H	-0.6473460000	-0.6847630000	2.2952920000
H	-2.2004670000	-0.3122090000	1.5088150000
H	-1.7989880000	-1.9859270000	1.9296500000
H	-2.6082330000	-1.0456040000	-0.9851010000
H	-2.2495950000	-2.7041030000	-0.4723340000
H	-1.3427900000	-1.9763980000	-1.8177060000

4-Ethyl-2,4-dimethylhexane

0 1

C	-0.8018800000	0.0052230000	0.3254770000
C	0.4428000000	-0.3464150000	-0.5437000000
C	-2.0791230000	-0.2273790000	-0.5358630000
C	-0.7816880000	1.4888470000	0.7890130000
C	1.8706330000	0.0356510000	-0.0862070000
C	-0.8982990000	-0.8689180000	1.5945680000
C	-2.3699360000	-1.6664650000	-0.9790640000
C	-0.7259430000	2.5694440000	-0.2969220000
C	2.4086200000	-0.7982610000	1.0869730000
C	2.8285140000	-0.0886070000	-1.2832920000
H	0.2883450000	0.1267600000	-1.5297410000
H	0.4353910000	-1.4336570000	-0.7411520000
H	-2.0208210000	0.4059460000	-1.4374540000
H	-2.9461430000	0.1446910000	0.0406890000
H	0.0736320000	1.6295100000	1.4727660000
H	-1.6811500000	1.6569190000	1.4091950000
H	1.8727350000	1.0965060000	0.2227150000
H	-0.1602900000	-0.5717550000	2.3535520000
H	-0.7378720000	-1.9364250000	1.3777810000
H	-1.8943940000	-0.7669800000	2.0583910000
H	-3.2956100000	-1.7004830000	-1.5765280000
H	-2.5100520000	-2.3458390000	-0.1233450000
H	-1.5645390000	-2.0809510000	-1.6063060000
H	-0.7024300000	3.5708610000	0.1631900000
H	0.1728630000	2.4813230000	-0.9286470000
H	-1.6028820000	2.5401280000	-0.9627320000
H	1.8516840000	-0.6315810000	2.0194560000
H	3.4641040000	-0.5489920000	1.2872010000
H	2.3616000000	-1.8776510000	0.8578640000
H	3.8556700000	0.1996060000	-1.0055720000
H	2.8640390000	-1.1285450000	-1.6535900000
H	2.5139250000	0.5518900000	-2.1236330000

2,4-Dimethyloctane

0 1

C	-0.4226000000	-0.6007220000	0.5275590000
C	-1.0529490000	0.0600470000	-0.7195280000
C	1.0443460000	-1.0132180000	0.2519910000
C	-2.5916430000	0.2409670000	-0.7962170000
C	2.0452410000	0.1230340000	0.0027100000
C	-0.5356000000	0.2383130000	1.8116340000
C	3.4730640000	-0.3811740000	-0.2387200000
C	-3.3655530000	-1.0440410000	-0.4693480000
C	-3.1367510000	1.4343860000	0.0046760000
C	4.4800430000	0.7442840000	-0.4864490000
H	-0.9618360000	-1.5481970000	0.7083890000
H	-0.5847630000	1.0473260000	-0.8856130000
H	-0.7614790000	-0.5575860000	-1.5891340000
H	1.4003860000	-1.6113600000	1.1115340000

H	1.0601090000	-1.6968520000	-0.6171820000
H	-2.7922420000	0.4724290000	-1.8596760000
H	2.0516320000	0.8149290000	0.8644800000
H	1.7282660000	0.7245930000	-0.8685670000
H	-0.1757820000	1.2701260000	1.6626280000
H	-1.5730650000	0.3023180000	2.1708190000
H	0.0621340000	-0.2084800000	2.6242100000
H	3.4733100000	-1.0736380000	-1.1009770000
H	3.7980250000	-0.9827270000	0.6305370000
H	-3.2845420000	-1.3105410000	0.5980140000
H	-2.9959980000	-1.9014860000	-1.0566070000
H	-4.4382610000	-0.9232920000	-0.6939660000
H	-4.1945520000	1.6178550000	-0.2486290000
H	-3.0873320000	1.2692200000	1.0921780000
H	-2.5766290000	2.3576350000	-0.2186930000
H	4.2032500000	1.3418230000	-1.3720680000
H	4.5283390000	1.4342730000	0.3734050000
H	5.4954500000	0.3503750000	-0.6549350000

2,5,5-Trimethylheptane

0 1

C	1.4661180000	-0.4867790000	0.0822090000
C	-0.0355540000	-0.2634250000	0.4159980000
C	-0.9836920000	-0.0404700000	-0.7710440000
C	2.0743680000	0.6906990000	-0.7269000000
C	-2.4802820000	0.1295030000	-0.4152990000
C	1.6483710000	-1.7760550000	-0.7432130000
C	2.2235850000	-0.6622130000	1.4136440000
C	1.9784930000	2.0854640000	-0.0997910000
C	-2.7513070000	1.3705110000	0.4478890000
C	-3.0885920000	-1.1287840000	0.2213940000
H	-0.1113170000	0.5924250000	1.1083150000
H	-0.3743620000	-1.1414320000	0.9940890000
H	-0.8992730000	-0.8857620000	-1.4762380000
H	-0.6668770000	0.8555380000	-1.3336460000
H	3.1385480000	0.4549590000	-0.9110620000
H	1.6033640000	0.7152160000	-1.7252480000
H	-2.9997020000	0.2867800000	-1.3794710000
H	2.7187090000	-1.9885010000	-0.9035040000
H	1.1778740000	-1.7024670000	-1.7364760000
H	1.2096280000	-2.6456920000	-0.2255360000
H	3.3035080000	-0.8061590000	1.2413350000
H	2.1010320000	0.2088760000	2.0768380000
H	1.8522200000	-1.5446260000	1.9610450000
H	2.4667780000	2.8323300000	-0.7468300000
H	2.4734230000	2.1338010000	0.8834610000
H	0.9337870000	2.4082790000	0.0383730000
H	-2.3172990000	1.2706250000	1.4570050000
H	-3.8346580000	1.5327830000	0.5739550000
H	-2.3286320000	2.2806280000	-0.0101040000
H	-2.9044080000	-2.0231220000	-0.3972900000
H	-2.6715640000	-1.3233090000	1.2237710000
H	-4.1798220000	-1.0218760000	0.3378960000

3-Ethyl-2,5-dimethylhexane

0 1

C	0.3738100000	0.2228980000	-0.0928860000
C	-0.6700390000	-0.3646910000	0.8952540000
C	1.6621100000	-0.6465460000	-0.2076120000
C	-2.1575980000	-0.3353780000	0.4611930000
C	0.6899870000	1.6972060000	0.2510440000
C	2.3757200000	-0.8821210000	1.1330810000
C	1.3967400000	-1.9811100000	-0.9202270000
C	-2.4381550000	-1.2668670000	-0.7274680000
C	-2.7093550000	1.0719020000	0.1915560000
C	1.4608320000	2.4715490000	-0.8229740000
H	-0.0720110000	0.2232460000	-1.1052430000
H	-0.5784470000	0.1588190000	1.8640910000
H	-0.4281550000	-1.4188650000	1.1109420000
H	2.3612590000	-0.0846880000	-0.8521460000
H	-2.7221710000	-0.7396830000	1.3228790000
H	-0.2594640000	2.2217520000	0.4426810000
H	1.2432380000	1.7389310000	1.2068560000
H	1.7589290000	-1.4790440000	1.8257780000
H	3.3170750000	-1.4343420000	0.9767430000
H	2.6301080000	0.0613190000	1.6414460000

H	0.7179540000	-2.6305330000	-0.3421570000
H	2.3354690000	-2.5408690000	-1.0662360000
H	0.9420220000	-1.8247910000	-1.9124210000
H	-1.9223760000	-0.9308390000	-1.6429490000
H	-2.1079010000	-2.2979300000	-0.5191250000
H	-3.5167230000	-1.2998350000	-0.9546810000
H	-2.2353000000	1.5389240000	-0.6884680000
H	-3.7931890000	1.0307840000	-0.0081110000
H	-2.5548940000	1.7421550000	1.0530980000
H	1.5847200000	3.5277370000	-0.5331230000
H	0.9264130000	2.4539330000	-1.7887620000
H	2.4691380000	2.0627650000	-0.9968640000

2,5-Dimethyloctane

0 1

C	0.9304190000	1.2783700000	0.3096190000
C	-0.4145510000	0.7268080000	-0.2094210000
C	-1.1676890000	-0.1683880000	0.7843340000
C	1.9555780000	0.1898480000	0.7044840000
C	-2.5795620000	-0.6309230000	0.3557080000
C	1.5183460000	2.2832340000	-0.6927790000
C	2.3899280000	-0.7790010000	-0.4037780000
C	-3.5687370000	0.5333770000	0.1991870000
C	-2.5572850000	-1.5096940000	-0.9036220000
C	3.4364170000	-1.7877730000	0.0782950000
H	0.7093180000	1.8392430000	1.2384090000
H	-0.2445700000	0.1824730000	-1.1554070000
H	-1.0474770000	1.5918490000	-0.4743960000
H	-0.5665340000	-1.0703640000	0.9960440000
H	-1.2562370000	0.3682700000	1.7476440000
H	1.5546510000	-0.3983740000	1.5492780000
H	2.8545520000	0.6974640000	1.1016210000
H	-2.9524640000	-1.2621850000	1.1844240000
H	1.7156390000	1.8166910000	-1.6725020000
H	0.8229760000	3.1211450000	-0.8655650000
H	2.4686360000	2.7070340000	-0.3275580000
H	1.5115910000	-1.3254490000	-0.7910270000
H	2.7990010000	-0.2147330000	-1.2603110000
H	-3.5962890000	1.1640980000	1.1036660000
H	-3.3070860000	1.1820930000	-0.6536640000
H	-4.5906650000	0.1592810000	0.0215180000
H	-3.5567110000	-1.9268050000	-1.1107970000
H	-1.8584640000	-2.3557720000	-0.7919140000
H	-2.2509240000	-0.9379610000	-1.7958090000
H	4.3474040000	-1.2781920000	0.4366080000
H	3.7354750000	-2.4784520000	-0.7267450000
H	3.0510050000	-2.3965020000	0.9143210000

2,6-Dimethyloctane

0 1

C	-2.3910720000	-0.6052240000	-0.2168730000
C	-0.9772860000	-0.5896970000	-0.8428900000
C	0.1909640000	-0.2952380000	0.1087930000
C	1.5507950000	-0.3396700000	-0.5999260000
C	2.7795860000	-0.1379890000	0.3074970000
C	-2.8079800000	0.7300650000	0.4325470000
C	-2.5700840000	-1.7634650000	0.7761400000
C	2.8165750000	1.2558870000	0.9499550000
C	4.0739960000	-0.4036000000	-0.4731220000
C	-2.7998950000	1.9395880000	-0.5063970000
H	-3.0921350000	-0.7852780000	-1.0550300000
H	-0.8063020000	-1.5714370000	-1.3223470000
H	-0.9552050000	0.1460450000	-1.6665720000
H	0.0475990000	0.6943610000	0.5751420000
H	0.1972290000	-1.0260980000	0.9378790000
H	1.6498280000	-1.3163230000	-1.1091670000
H	1.5713870000	0.4217050000	-1.4041300000
H	2.7175910000	-0.8856730000	1.1225890000
H	-2.1607430000	0.9348660000	1.3050880000
H	-3.8256110000	0.6037850000	0.8441250000
H	-1.9488470000	-1.6294370000	1.6776690000
H	-2.2948760000	-2.7297760000	0.3217210000
H	-3.6178050000	-1.8377980000	1.1112860000
H	1.9365480000	1.4466060000	1.5838930000
H	2.8495790000	2.0437080000	0.1760660000
H	3.7110890000	1.3757150000	1.5832840000

H	4.0858300000	-1.4190500000	-0.9025150000
H	4.9617890000	-0.3003520000	0.1723630000
H	4.1847060000	0.3115270000	-1.3074740000
H	-1.7855690000	2.1815770000	-0.8633370000
H	-3.4290540000	1.7606050000	-1.3954840000
H	-3.1906250000	2.8367740000	0.0006020000

2,7-Dimethyloctane

0 1

C	0.6136130000	0.2743980000	-0.4393570000
C	-0.7757130000	-0.1195920000	0.0774510000
C	1.7575930000	-0.4251400000	0.3040000000
C	-1.9129960000	0.6007370000	-0.6591880000
C	3.1699110000	-0.1672370000	-0.2548710000
C	-3.3533840000	0.2427860000	-0.2292630000
C	3.5805070000	1.3093640000	-0.1654370000
C	4.2001460000	-1.0554430000	0.4558050000
C	-3.7047110000	-1.2275000000	-0.5008610000
C	-3.6478090000	0.6199020000	1.2300340000
H	0.7262830000	1.3706660000	-0.3632010000
H	0.6805600000	0.0369490000	-1.5183570000
H	-0.8955470000	-1.2137070000	-0.0199280000
H	-0.8300870000	0.0973000000	1.1596040000
H	1.5716310000	-1.5151880000	0.2893590000
H	1.7356050000	-0.1307490000	1.3716050000
H	-1.7754600000	1.6919300000	-0.5414430000
H	-1.8142720000	0.4011810000	-1.7426260000
H	3.1607050000	-0.4519230000	-1.3254620000
H	-4.0192380000	0.8575350000	-0.8640370000
H	3.5795510000	1.6543450000	0.8840910000
H	2.9030020000	1.9654500000	-0.7339670000
H	4.5974990000	1.4615410000	-0.5633160000
H	3.9424880000	-2.1237920000	0.3668420000
H	4.2523420000	-0.8144060000	1.5323570000
H	5.2097800000	-0.9164280000	0.0353750000
H	-3.5029100000	-1.5033580000	-1.5495880000
H	-3.1264290000	-1.9121920000	0.1423370000
H	-4.7724380000	-1.4198740000	-0.3042500000
H	-3.0608240000	0.0087050000	1.9362460000
H	-4.7125340000	0.4632520000	1.4702630000
H	-3.4126450000	1.6791120000	1.4282190000

3,3-Diethyl-2-methylpentane

0 1

C	-0.1430490000	0.0475830000	-0.1253130000
C	1.2564820000	0.3868360000	0.5194830000
C	-0.0594080000	-1.0719050000	-1.2120740000
C	-0.6843830000	1.3096660000	-0.8671220000
C	-1.1222410000	-0.3508570000	1.0160800000
C	1.9381430000	-0.7570090000	1.2942190000
C	2.2829300000	0.9828360000	-0.4608280000
C	-0.0090470000	-2.5436640000	-0.7774000000
C	-0.8387990000	2.6002360000	-0.0543770000
C	-2.5966600000	-0.5328160000	0.6331340000
H	1.0405000000	1.1714900000	1.2671470000
H	-0.9341030000	-0.9583850000	-1.8767410000
H	0.8098570000	-0.8608780000	-1.8579850000
H	-1.6655210000	1.0509610000	-1.2984870000
H	-0.0384010000	1.5092940000	-1.7386020000
H	-0.7698370000	-1.2862450000	1.4770920000
H	-1.0570720000	0.4074810000	1.8161340000
H	1.2659910000	-1.2602100000	2.0042650000
H	2.3517890000	-1.5210830000	0.6167770000
H	2.7844490000	-0.3554470000	1.8759410000
H	2.5464450000	0.2724980000	-1.2621880000
H	1.9352090000	1.9110690000	-0.9371670000
H	3.2164680000	1.2224030000	0.0744850000
H	0.8505040000	-2.7765790000	-0.1338920000
H	0.0656530000	-3.1899750000	-1.6678450000
H	-0.9185430000	-2.8492210000	-0.2362120000
H	-1.5013210000	2.4661240000	0.8160220000
H	0.1234280000	2.9838140000	0.3202230000
H	-1.2813740000	3.3926520000	-0.6800600000
H	-2.7280950000	-1.2508110000	-0.1926090000
H	-3.1689640000	-0.9167080000	1.4935090000
H	-3.0708690000	0.4123280000	0.3253730000

3-Ethyl-2-methylheptane

0 1

C	0.8171900000	0.2559270000	-0.2222690000
C	1.2776870000	-1.2286150000	-0.1222490000
C	-0.5523710000	0.4941970000	0.4515850000
C	1.8760020000	1.2514320000	0.3033130000
C	-1.7604060000	-0.0863670000	-0.2941960000
C	2.3416270000	-1.5718380000	-1.1759450000
C	1.7580500000	-1.6344610000	1.2802180000
C	-3.0973560000	0.1995510000	0.3993180000
C	1.7191310000	2.6768700000	-0.2371420000
C	-4.3043460000	-0.3650750000	-0.3533570000
H	0.6854070000	0.4645930000	-1.3022990000
H	0.3935080000	-1.8487060000	-0.3554910000
H	-0.5295570000	0.0936370000	1.4820970000
H	-0.7126700000	1.5807900000	0.5633330000
H	1.8427560000	1.2719540000	1.4077130000
H	2.8849890000	0.8919980000	0.0392600000
H	-1.7873710000	0.3294320000	-1.3195350000
H	-1.6489610000	-1.1793460000	-0.4149950000
H	2.6054100000	-2.6416790000	-1.1355030000
H	1.9829810000	-1.3536350000	-2.1955220000
H	3.2732900000	-1.0014740000	-1.0209770000
H	1.9541270000	-2.7184630000	1.3227500000
H	1.0129700000	-1.4047740000	2.0587360000
H	2.6954470000	-1.1220280000	1.5531030000
H	-3.0723150000	-0.2177280000	1.4230840000
H	-3.2170420000	1.2920630000	0.5221910000
H	2.4894380000	3.3467360000	0.1784300000
H	0.7380290000	3.1120880000	0.0140900000
H	1.8172980000	2.6982650000	-1.3360600000
H	-5.2486310000	-0.1427580000	0.1695570000
H	-4.3775980000	0.0603780000	-1.3689550000
H	-4.2327470000	-1.4610640000	-0.4606630000

4-Ethyl-2-methylheptane

0 1

C	-0.3271990000	-0.7909760000	0.1899980000
C	0.9674900000	-0.5451830000	-0.6147290000
C	-1.0169800000	0.5103860000	0.6617810000
C	2.0797170000	0.2837080000	0.0582700000
C	-1.2610050000	-1.7247670000	-0.6103170000
C	-1.5086370000	1.4614640000	-0.4378260000
C	2.5844680000	-0.3539510000	1.3602890000
C	3.2426660000	0.4957590000	-0.9216120000
C	-2.5187760000	-2.1842660000	0.1329760000
C	-2.1772110000	2.7177510000	0.1278780000
H	-0.0500760000	-1.3424680000	1.1082660000
H	0.7076380000	-0.0646140000	-1.5757150000
H	1.3929290000	-1.5302250000	-0.8861110000
H	-1.8734470000	0.2442010000	1.3064920000
H	-0.3240100000	1.0625950000	1.3213950000
H	1.6712530000	1.2814130000	0.3050300000
H	-0.6772540000	-2.6155970000	-0.9037700000
H	-1.5505780000	-1.2373010000	-1.5591230000
H	-2.2226330000	0.9375440000	-1.0977320000
H	-0.6641930000	1.7612410000	-1.0832340000
H	1.7912320000	-0.4401250000	2.1195330000
H	3.3995960000	0.2427140000	1.8021650000
H	2.9793680000	-1.3686020000	1.1732430000
H	2.9046330000	0.9877700000	-1.8486680000
H	3.7005510000	-0.4683560000	-1.2053660000
H	4.0340810000	1.1218500000	-0.4776870000
H	-3.2052820000	-1.3498340000	0.3495020000
H	-2.2636300000	-2.6601530000	1.0957140000
H	-3.0807910000	-2.9221380000	-0.4623210000
H	-1.4805630000	3.2901070000	0.7643280000
H	-3.0529260000	2.4612120000	0.7485100000
H	-2.5231030000	3.3892250000	-0.6746970000

5-Ethyl-2-methylheptane

0 1

C	1.2129850000	-0.3437330000	0.3638660000
C	-0.2306150000	-0.3284640000	-0.1846890000

C	-1.2943220000	0.1758170000	0.8007200000
C	-2.7643240000	0.0643760000	0.3330570000
C	1.7222470000	1.0489670000	0.8040890000
C	2.1471990000	-1.0435970000	-0.6460860000
C	-3.2218570000	-1.3887410000	0.1392050000
C	-3.0518690000	0.9051750000	-0.9196260000
C	1.8208910000	2.1076150000	-0.3008800000
C	3.5844580000	-1.2644610000	-0.1654190000
H	1.2036150000	-0.9707260000	1.2779280000
H	-0.2612970000	0.2711780000	-1.1117490000
H	-0.4793090000	-1.3595650000	-0.4907060000
H	-1.0983970000	1.2341550000	1.0470210000
H	-1.1893700000	-0.3802610000	1.7512740000
H	-3.3733770000	0.4872330000	1.1542800000
H	2.7130640000	0.9289230000	1.2746740000
H	1.0674520000	1.4280980000	1.6070780000
H	1.7023180000	-2.0235390000	-0.8955990000
H	2.1588720000	-0.4760450000	-1.5946790000
H	-4.3036170000	-1.4353340000	-0.0691820000
H	-2.7064880000	-1.8724200000	-0.7075860000
H	-3.0286860000	-1.9955940000	1.0397740000
H	-4.1291180000	0.9053240000	-1.1550080000
H	-2.5243640000	0.5124260000	-1.8053200000
H	-2.7398540000	1.9541920000	-0.7821170000
H	2.5145900000	1.8059870000	-1.1028050000
H	0.8437170000	2.3102390000	-0.7694320000
H	2.1906710000	3.0619920000	0.1081910000
H	4.1575930000	-1.8567170000	-0.8971700000
H	4.1265900000	-0.3164980000	-0.0177480000
H	3.6066300000	-1.8107790000	0.7934620000

2-Methylnonane

0 1

C	1.0020000000	0.0062180000	-0.0757720000
C	2.1737830000	-0.0363320000	0.9142580000
C	-0.3657930000	-0.0300880000	0.6160170000
C	3.5947430000	-0.0099060000	0.3075890000
C	-1.5494550000	0.0186730000	-0.3557220000
C	-2.9170220000	-0.0186050000	0.3338770000
C	-4.1004390000	0.0324200000	-0.6383000000
C	3.8845200000	1.2851610000	-0.4653490000
C	3.8934170000	-1.2457270000	-0.5537570000
C	-5.4620050000	-0.0065250000	0.0593570000
H	1.0695610000	-0.8438360000	-0.7785510000
H	1.0643100000	0.9190110000	-0.6953500000
H	2.0782530000	-0.9457890000	1.5365530000
H	2.0775090000	0.8162730000	1.6123250000
H	-0.4395700000	0.8172600000	1.3238930000
H	-0.4382930000	-0.9450000000	1.2342570000
H	4.2906000000	-0.0377740000	1.1674180000
H	-1.4772500000	0.9343690000	-0.9728740000
H	-1.4752550000	-0.8280100000	-1.0643560000
H	-2.9911420000	-0.9351750000	0.9498190000
H	-2.9919960000	0.8273680000	1.0435600000
H	-4.0267590000	0.9489310000	-1.2527520000
H	-4.0248060000	-0.8127210000	-1.3474980000
H	4.9404170000	1.3283300000	-0.7799600000
H	3.6824680000	2.1768830000	0.1516130000
H	3.2687310000	1.3624270000	-1.3772110000
H	4.9485900000	-1.2574990000	-0.8736190000
H	3.2754840000	-1.2664400000	-1.4673460000
H	3.7017570000	-2.1796360000	0.0009230000
H	-6.2905770000	0.0321230000	-0.6662620000
H	-5.5815230000	-0.9290730000	0.6529980000
H	-5.5830130000	0.8462410000	0.7492340000

3,3,4,4-Tetramethylhexane

0 1

C	-0.7862440000	0.2956950000	-0.1544280000
C	0.7862440000	0.2956950000	0.1544280000
C	-1.4839930000	-0.8699220000	0.6117550000
C	1.4839890000	-0.8699310000	-0.6117450000
C	-1.0477700000	0.1491700000	-1.6712260000
C	-1.4301990000	1.6263850000	0.2970360000
C	1.0477700000	0.1491830000	1.6712280000
C	1.4302020000	1.6263780000	-0.2970490000

C	-2.9291880000	-1.1956470000	0.2113410000
C	2.9291880000	-1.1956470000	-0.2113390000
H	-1.4715310000	-0.6369910000	1.6891040000
H	-0.8890090000	-1.7914900000	0.4967900000
H	1.4715180000	-0.6370130000	-1.6890970000
H	0.8890080000	-1.7914990000	-0.4967650000
H	-0.4515140000	0.8649890000	-2.2577700000
H	-0.8196980000	-0.8612810000	-2.0432600000
H	-2.1048320000	0.3512250000	-1.9031700000
H	-1.1069350000	2.4722480000	-0.3277170000
H	-1.1949520000	1.8716130000	1.3447410000
H	-2.5267960000	1.5706480000	0.2149760000
H	0.8197000000	-0.8612640000	2.0432720000
H	0.4515140000	0.8650070000	2.2577660000
H	2.1048320000	0.3512420000	1.9031700000
H	1.1949530000	1.8715980000	-1.3447550000
H	1.1069430000	2.4722480000	0.3276980000
H	2.5267990000	1.5706380000	-0.2149910000
H	-3.0034080000	-1.5713160000	-0.8211630000
H	-3.5985920000	-0.3242040000	0.2971590000
H	-3.3345230000	-1.9795160000	0.8718110000
H	3.5985890000	-0.3242030000	-0.2971710000
H	3.0034180000	-1.5713050000	0.8211690000
H	3.3345210000	-1.9795220000	-0.8718040000

3,3,4-Trimethylheptane

0 1

C	1.2949840000	-0.1911280000	0.2679140000
C	-0.0678180000	-0.4938980000	-0.4541870000
C	1.4879960000	1.3365490000	0.5023020000
C	-1.3102970000	-0.0802140000	0.3686100000
C	1.3648500000	-0.8767090000	1.6485070000
C	2.4733770000	-0.7094430000	-0.5853090000
C	-0.2027520000	-1.9577130000	-0.9131370000
C	-2.5783100000	0.1485380000	-0.4662330000
C	1.4659380000	2.2474390000	-0.7301260000
C	-3.7881530000	0.5456710000	0.3835330000
H	-0.0680280000	0.1238690000	-1.3709210000
H	0.7318250000	1.6939540000	1.2215490000
H	2.4576990000	1.4635980000	1.0166850000
H	-1.5175790000	-0.8513950000	1.1335960000
H	-1.1061230000	0.8483280000	0.9275020000
H	2.3485010000	-0.7001320000	2.1147750000
H	0.6004020000	-0.4883440000	2.3399090000
H	1.2302760000	-1.9674120000	1.5741730000
H	2.4142630000	-0.3637050000	-1.6301060000
H	3.4323980000	-0.3565390000	-0.1704220000
H	2.5120890000	-1.8090670000	-0.6031580000
H	-0.1328720000	-2.6599240000	-0.0655130000
H	0.5659040000	-2.2392060000	-1.6474640000
H	-1.1791190000	-2.1264080000	-1.3931740000
H	-2.8249600000	-0.7600830000	-1.0428430000
H	-2.3796270000	0.9373410000	-1.2154920000
H	0.4865010000	2.2465040000	-1.2347060000
H	2.2241300000	1.9571030000	-1.4751410000
H	1.6782770000	3.2888810000	-0.4381310000
H	-3.5944800000	1.4734470000	0.9488850000
H	-4.6820190000	0.7149130000	-0.2385100000
H	-4.0363490000	-0.2402750000	1.1172600000

3,3,5-Trimethylheptane

0 1

C	-1.1560030000	-0.4663260000	0.0846500000
C	0.3759820000	-0.6184350000	-0.1810350000
C	1.4062710000	0.2360070000	0.5955410000
C	-1.7226280000	0.7482770000	-0.7037380000
C	-1.4425630000	-0.3332600000	1.5921940000
C	-1.8215950000	-1.7605220000	-0.4316940000
C	2.8293890000	-0.3565080000	0.4337590000
C	1.3977040000	1.7363710000	0.2580210000
C	-3.2224380000	1.0334720000	-0.5570380000
C	3.4237240000	-0.3210900000	-0.9791830000
H	0.5413360000	-0.4917330000	-1.2664620000
H	0.6291990000	-1.6724980000	0.0344430000
H	1.1739550000	0.1416070000	1.6712060000
H	-1.1723480000	1.6553170000	-0.4110240000

H	-1.4934230000	0.5914400000	-1.7738560000
H	-2.5215920000	-0.4017130000	1.8023700000
H	-0.9489290000	-1.1402870000	2.1593860000
H	-1.0873610000	0.6274720000	1.9988530000
H	-2.9165380000	-1.7345380000	-0.3195180000
H	-1.5966790000	-1.9234600000	-1.4995570000
H	-1.4543490000	-2.6392490000	0.1239160000
H	3.5056680000	0.1888880000	1.1167170000
H	2.8168160000	-1.4018800000	0.7917360000
H	0.4906690000	2.2383300000	0.6255730000
H	1.4574650000	1.9151120000	-0.8284930000
H	2.2583620000	2.2436170000	0.7263560000
H	-3.5014790000	1.2616670000	0.4844550000
H	-3.8472530000	0.1902170000	-0.8920820000
H	-3.5044070000	1.9076740000	-1.1664040000
H	2.8249340000	-0.9064960000	-1.6957270000
H	3.4976280000	0.7073460000	-1.3689940000
H	4.4412720000	-0.7447570000	-0.9817630000

3,3-Diethylhexane

0 1

C	0.4010750000	-0.0000050000	0.0712970000
C	-0.9898290000	0.0000010000	-0.6234320000
C	0.5684270000	-1.2454430000	0.9879360000
C	1.4608240000	-0.0000100000	-1.0677770000
C	0.5684390000	1.2454300000	0.9879390000
C	-2.2367300000	0.0000160000	0.2708100000
C	0.4446440000	-2.6236040000	0.3296570000
C	2.9365610000	0.0000090000	-0.6552660000
C	0.4446650000	2.6235920000	0.3296610000
C	-3.5343600000	0.0000110000	-0.5434330000
H	-1.0424780000	0.8754700000	-1.2953040000
H	-1.0424890000	-0.8754750000	-1.2952920000
H	1.5504110000	-1.1781090000	1.4875140000
H	-0.1711360000	-1.1741180000	1.8040900000
H	1.2731600000	-0.8751890000	-1.7135340000
H	1.2731460000	0.8751510000	-1.7135540000
H	1.5504230000	1.1780870000	1.4875150000
H	-0.1711230000	1.1741090000	1.8040930000
H	-2.2285700000	-0.8814650000	0.9354610000
H	-2.2285640000	0.8815140000	0.9354390000
H	0.5827370000	-3.4193720000	1.0797950000
H	-0.5444900000	-2.7796460000	-0.1304410000
H	1.2027590000	-2.7818760000	-0.4542730000
H	3.2049880000	0.8885930000	-0.0615090000
H	3.2050140000	-0.8885750000	-0.0615210000
H	3.5826020000	0.0000250000	-1.5484140000
H	1.2027940000	2.7818680000	-0.4542560000
H	0.5827450000	3.4193600000	1.0798020000
H	-0.5444610000	2.7796330000	-0.1304530000
H	-3.5988240000	-0.8887040000	-1.1944840000
H	-4.4221420000	0.0000500000	0.1095110000
H	-3.5987910000	0.8886850000	-1.1945430000

4-Ethyl-3,3-dimethylhexane

0 1

C	-0.8637290000	-0.4221730000	0.3961530000
C	0.4751790000	-0.1158620000	-0.3799990000
C	-1.9924460000	0.5773070000	0.0131010000
C	0.8778680000	1.3788530000	-0.5092420000
C	1.6639580000	-0.9837990000	0.0976740000
C	-0.6987930000	-0.3622520000	1.9309560000
C	-1.3372900000	-1.8520540000	0.0437050000
C	-2.4308620000	0.6134130000	-1.4559090000
C	1.3413170000	2.1291020000	0.7465870000
C	2.8597770000	-1.0096210000	-0.8618310000
H	0.2724640000	-0.4347090000	-1.4202100000
H	-1.6981000000	1.5929670000	0.3276700000
H	-2.8734540000	0.3264300000	0.6312200000
H	0.0498690000	1.9399550000	-0.9700470000
H	1.6929890000	1.4270500000	-1.2515130000
H	1.3198110000	-2.0204360000	0.2408220000
H	2.0079830000	-0.6457330000	1.0900990000
H	0.0754730000	-1.0576470000	2.2893720000
H	-0.4381410000	0.6443100000	2.2882240000
H	-1.6443250000	-0.6485540000	2.4215730000

H	-2.3382030000	-2.0401530000	0.4672460000
H	-1.3980250000	-2.0140240000	-1.0440990000
H	-0.6652320000	-2.6207240000	0.4546390000
H	-3.2330380000	1.3563010000	-1.5966810000
H	-2.8255340000	-0.3580300000	-1.7933380000
H	-1.6091160000	0.8900850000	-2.1355480000
H	2.1514760000	1.6014120000	1.2758330000
H	1.7289360000	3.1237880000	0.4704830000
H	0.5259140000	2.2904860000	1.4680650000
H	3.3440870000	-0.0248010000	-0.9587460000
H	2.5554340000	-1.3296040000	-1.8734430000
H	3.6288590000	-1.7158490000	-0.5087970000

3,3-Dimethyloctane

0 1

C	-1.8035850000	-0.4363980000	0.0140300000
C	-0.3842730000	-0.3795410000	0.6407290000
C	-2.1573970000	0.8605990000	-0.7628600000
C	0.7972800000	-0.1268720000	-0.3044110000
C	-1.9090470000	-1.6212790000	-0.9664610000
C	-2.8156380000	-0.6653360000	1.1545430000
C	2.1484050000	-0.1344790000	0.4207190000
C	-2.0873890000	2.1779430000	0.0164700000
C	3.3458570000	0.1212190000	-0.5004990000
C	4.6890920000	0.1067050000	0.2329030000
H	-0.2136070000	-1.3354310000	1.1704630000
H	-0.3757030000	0.3957200000	1.4281080000
H	-1.4985750000	0.9334270000	-1.6460450000
H	-3.1787280000	0.7393400000	-1.1677850000
H	0.8171960000	-0.8907430000	-1.1020720000
H	0.6728170000	0.8443610000	-0.8164580000
H	-2.9378660000	-1.7188590000	-1.3516350000
H	-1.6455860000	-2.5720390000	-0.4731940000
H	-1.2444430000	-1.4966110000	-1.8360320000
H	-2.7634000000	0.1283810000	1.9166660000
H	-3.8490650000	-0.6973390000	0.7701430000
H	-2.6194950000	-1.6228240000	1.6653850000
H	2.2834400000	-1.1069580000	0.9315260000
H	2.1378520000	0.6273490000	1.2235640000
H	-2.3868570000	3.0213690000	-0.6268530000
H	-1.0691520000	2.3921960000	0.3799030000
H	-2.7588030000	2.1809520000	0.8903160000
H	3.2130250000	1.0945570000	-1.0083040000
H	3.3557770000	-0.6389780000	-1.3035730000
H	5.5291780000	0.2931120000	-0.4555680000
H	4.7243440000	0.8802720000	1.0191680000
H	4.8678190000	-0.8666160000	0.7213060000

3,4,4-Trimethylheptane

0 1

C	-0.0633340000	-0.9469690000	0.1803620000
C	1.0302400000	0.0405450000	-0.3672520000
C	-1.3819570000	-0.2004360000	0.5409910000
C	1.4358920000	1.1372120000	0.6448350000
C	0.4137220000	-1.6659940000	1.4594380000
C	-0.3830070000	-2.0209860000	-0.8834790000
C	2.2827520000	-0.6740440000	-0.9068200000
C	-2.0611720000	0.6328010000	-0.5537950000
C	2.1308280000	2.3499770000	0.0144290000
C	-3.3995560000	1.2169700000	-0.0905700000
H	0.5674900000	0.5551710000	-1.2295430000
H	-2.1009770000	-0.9656420000	0.8885550000
H	-1.2099180000	0.4481200000	1.4177110000
H	0.5446480000	1.4982440000	1.1825760000
H	2.0965560000	0.6993540000	1.4148370000
H	0.5919630000	-0.9593170000	2.2852020000
H	-0.3495350000	-2.3843650000	1.8023340000
H	1.3432790000	-2.2326490000	1.2926630000
H	0.4507460000	-2.7270420000	-1.0159250000
H	-1.2640410000	-2.6116670000	-0.5816760000
H	-0.5999000000	-1.5775680000	-1.8688840000
H	3.0177510000	0.0566540000	-1.2775600000
H	2.0515760000	-1.3458990000	-1.7458360000
H	2.7835350000	-1.2699700000	-0.1257420000
H	-1.4012240000	1.4592000000	-0.8701830000
H	-2.2283960000	0.0147150000	-1.4531270000

H	3.0774510000	2.0816360000	-0.4813650000
H	1.4861050000	2.8297900000	-0.7419870000
H	2.3672660000	3.1091990000	0.7776340000
H	-3.2691530000	1.8658310000	0.7926020000
H	-4.1092620000	0.4199510000	0.1900700000
H	-3.8724010000	1.8217960000	-0.8811910000

3,4,5-Trimethylheptane

0 1

C	-0.0219930000	-0.4813630000	-0.3630820000
C	1.4229560000	-0.5222280000	0.2191810000
C	-1.0772010000	0.0896480000	0.6319890000
C	1.8964780000	0.8106300000	0.8496250000
C	-2.4622310000	0.3444430000	-0.0044580000
C	-0.4365700000	-1.8558320000	-0.9199800000
C	2.4464310000	-1.0113810000	-0.8228620000
C	-1.2444730000	-0.7620850000	1.9009390000
C	1.9591540000	2.0194530000	-0.0918890000
C	-2.4757670000	1.2838830000	-1.2138260000
H	0.0088890000	0.2164160000	-1.2193190000
H	1.4269480000	-1.2654750000	1.0385900000
H	-0.7095640000	1.0812170000	0.9504690000
H	1.2606780000	1.0615210000	1.7151030000
H	2.9034230000	0.6375550000	1.2702270000
H	-3.1096840000	0.7734440000	0.7812550000
H	-2.9352020000	-0.6160700000	-0.2774630000
H	0.1933050000	-2.1561830000	-1.7698830000
H	-1.4760590000	-1.8509350000	-1.2818720000
H	-0.3557300000	-2.6467650000	-0.1550410000
H	2.2838160000	-2.0626530000	-1.1020630000
H	2.4059980000	-0.4146260000	-1.7501260000
H	3.4718920000	-0.9363150000	-0.4247360000
H	-0.2947280000	-0.9026400000	2.4399210000
H	-1.6471270000	-1.7623970000	1.6699640000
H	-1.9495840000	-0.2814490000	2.5989570000
H	2.3462070000	2.9046000000	0.4388250000
H	0.9672900000	2.2865370000	-0.4918520000
H	2.6240050000	1.8379800000	-0.9519910000
H	-3.5092880000	1.5053270000	-1.5261730000
H	-1.9580340000	0.8535800000	-2.0861870000
H	-1.9881530000	2.2463180000	-0.9804350000

3,4-Diethylhexane

0 1

C	-0.7795120000	0.0147360000	0.0628170000
C	0.7795070000	-0.0147350000	0.0629000000
C	-1.3900550000	-1.3744830000	0.3538210000
C	-1.3680970000	0.6245780000	-1.2299540000
C	1.3682290000	-0.6246420000	-1.2297780000
C	1.3900190000	1.3744990000	0.3538970000
C	-1.2565760000	-1.8453120000	1.8056880000
C	-2.7988610000	1.1520140000	-1.0778600000
C	2.7989780000	-1.1520660000	-1.0775060000
C	1.2563780000	1.8454060000	1.8057240000
H	-1.0873880000	0.6752030000	0.8952800000
H	1.0872950000	-0.6751590000	0.8954290000
H	-0.9367590000	-2.1231340000	-0.3214780000
H	-2.4621960000	-1.3555060000	0.0942860000
H	-1.3383770000	-0.1277800000	-2.0384400000
H	-0.7288910000	1.4566470000	-1.5710180000
H	1.3385920000	0.1276740000	-2.0383060000
H	0.7290600000	-1.4567300000	-1.5708670000
H	0.9367990000	2.1231140000	-0.3214930000
H	2.4621890000	1.3555070000	0.0944820000
H	-1.7178650000	-2.8366290000	1.9450570000
H	-1.7561760000	-1.1462460000	2.4981600000
H	-0.2045190000	-1.9277430000	2.1241590000
H	-3.1735280000	1.5658420000	-2.0282230000
H	-2.8478970000	1.9553860000	-0.3229260000
H	-3.5034120000	0.3639190000	-0.7650590000
H	2.8479360000	-1.9554010000	-0.3225280000
H	3.1737490000	-1.5659390000	-2.0278090000
H	3.5034930000	-0.3639530000	-0.7646670000
H	1.7176490000	2.8367310000	1.9450910000
H	1.7559020000	1.1463780000	2.4982900000
H	0.2042850000	1.9278510000	2.1240730000

3-Ethyl-3,4-dimethylhexane

0 1

C	0.6376620000	0.0185590000	0.1867550000
C	-0.9007160000	-0.2250020000	0.4262040000
C	0.8817090000	1.4073520000	-0.4767850000
C	1.2139190000	-1.0834400000	-0.7485170000
C	1.3722500000	-0.0130470000	1.5467420000
C	-1.7419300000	-0.2427810000	-0.8716040000
C	-1.2063480000	-1.4744670000	1.2733180000
C	0.3045440000	2.6385010000	0.2322390000
C	2.7308850000	-1.0799790000	-0.9806910000
C	-3.2377290000	0.0145810000	-0.6529260000
H	-1.2480870000	0.6413070000	1.0181950000
H	1.9721130000	1.5457700000	-0.5664360000
H	0.5063230000	1.3874310000	-1.5143800000
H	0.7133500000	-1.0087090000	-1.7291110000
H	0.9354480000	-2.0706430000	-0.3421300000
H	1.4451590000	-1.0349790000	1.9496960000
H	0.8545960000	0.6013990000	2.3009850000
H	2.3984150000	0.3766780000	1.4559180000
H	-1.6124990000	-1.2141400000	-1.3816740000
H	-1.3647770000	0.5184210000	-1.5732020000
H	-2.2912500000	-1.5818060000	1.4264770000
H	-0.8563410000	-2.3999160000	0.7866150000
H	-0.7479810000	-1.4258200000	2.2714620000
H	-0.7970220000	2.6405000000	0.2450950000
H	0.6231830000	3.5584580000	-0.2846380000
H	0.6490280000	2.7163450000	1.2762670000
H	3.0107960000	-1.8998430000	-1.6622040000
H	3.0851120000	-0.1432630000	-1.4398730000
H	3.2954060000	-1.2279550000	-0.0464440000
H	-3.7069920000	-0.7518010000	-0.0152910000
H	-3.4079580000	0.9933260000	-0.1721260000
H	-3.7815030000	0.0175790000	-1.6116100000

3,4-Dimethyloctane

0 1

C	-0.3837410000	0.6821450000	0.5933020000
C	-1.8150010000	0.0698630000	0.5341050000
C	0.6756800000	-0.1608400000	-0.1474560000
C	-2.3135400000	-0.1901950000	-0.9033820000
C	2.1305090000	0.1993860000	0.1815460000
C	-0.3771290000	2.1467250000	0.1264320000
C	-1.9319680000	-1.1856150000	1.4130790000
C	3.1526200000	-0.7088890000	-0.5113750000
C	-3.8212890000	-0.4419920000	-1.0136200000
C	4.6036860000	-0.3457810000	-0.1872830000
H	-0.1033450000	0.6855430000	1.6645430000
H	-2.4860270000	0.8331590000	0.9742530000
H	0.5210070000	-0.0706400000	-1.2386350000
H	0.5253140000	-1.2282720000	0.0919040000
H	-1.7685680000	-1.0512210000	-1.3308840000
H	-2.0587810000	0.6754910000	-1.5384350000
H	2.2805150000	0.1451340000	1.2768070000
H	2.3370850000	1.2474070000	-0.1005980000
H	0.6008420000	2.6234510000	0.2954540000
H	-1.1288880000	2.7400050000	0.6728170000
H	-0.6011570000	2.2355130000	-0.9503030000
H	-2.9707410000	-1.5464170000	1.4691080000
H	-1.3186640000	-2.0165410000	1.0250260000
H	-1.6004770000	-0.9799500000	2.4443740000
H	2.9965910000	-0.6636360000	-1.6052240000
H	2.9612090000	-1.7591440000	-0.2227960000
H	-4.3977890000	0.4052130000	-0.6037210000
H	-4.1250350000	-0.5719370000	-2.0650770000
H	-4.1336820000	-1.3480290000	-0.4696880000
H	4.8004350000	-0.4121660000	0.8965580000
H	4.8377620000	0.6859450000	-0.5011360000
H	5.3134330000	-1.0177560000	-0.6964380000

3,5-Dimethyloctane

0 1

C	0.7082550000	0.9022580000	-0.1482770000

C	-0.3999050000	-0.1700010000	-0.0783980000
C	-1.8163980000	0.2997830000	0.3133940000
C	2.0785690000	0.2775240000	-0.4850140000
C	-2.8302970000	-0.8630660000	0.2653220000
C	0.3755970000	2.0066460000	-1.1623950000
C	-1.8404910000	0.9543520000	1.7022000000
C	2.6588940000	-0.6755960000	0.5672060000
C	-3.0840010000	-1.4612530000	-1.1217950000
C	4.0694470000	-1.1575160000	0.2166930000
H	0.8050430000	1.3743270000	0.8476520000
H	-0.4437550000	-0.6659460000	-1.0650550000
H	-0.0999420000	-0.9528080000	0.6416050000
H	-2.1550120000	1.0525740000	-0.4234640000
H	2.0058020000	-0.2498240000	-1.4563480000
H	2.8023530000	1.0982910000	-0.6437610000
H	-2.4948890000	-1.6595850000	0.9568050000
H	-3.7904750000	-0.4976740000	0.6716190000
H	0.2395430000	1.5848730000	-2.1741340000
H	1.1894620000	2.7480060000	-1.2192480000
H	-0.5463130000	2.5498140000	-0.9028580000
H	-2.8632660000	1.2613970000	1.9761900000
H	-1.2032550000	1.8508570000	1.7542300000
H	-1.4872320000	0.2496110000	2.4759730000
H	2.0004690000	-1.5522970000	0.6929030000
H	2.6776780000	-0.1652540000	1.5483300000
H	-3.8761160000	-2.2264720000	-1.0804690000
H	-3.4093690000	-0.6864830000	-1.8375290000
H	-2.1872020000	-1.9442380000	-1.5413870000
H	4.7713190000	-0.3107850000	0.1262420000
H	4.4670570000	-1.8401780000	0.9850670000
H	4.0800900000	-1.6969920000	-0.7459850000

3,6-Dimethyloctane

0 1

C	-0.4016320000	-0.0323080000	0.4508500000
C	0.7737450000	0.6590950000	-0.2512070000
C	-1.7806880000	0.1853480000	-0.2064340000
C	2.1379310000	0.5093000000	0.4541720000
C	-2.8132360000	-0.7886110000	0.3966320000
C	2.6057230000	-0.9545340000	0.6280450000
C	-2.2449810000	1.6453710000	-0.0995900000
C	3.2002390000	1.3688650000	-0.2479030000
C	-4.1833320000	-0.7977880000	-0.2877110000
C	2.7970010000	-1.7594240000	-0.6626100000
H	-0.4505790000	0.3056660000	1.5043950000
H	-0.2109170000	-1.1187940000	0.4921520000
H	0.5651880000	1.7390120000	-0.3424900000
H	0.8502420000	0.2834560000	-1.2890180000
H	-1.6806910000	-0.0639770000	-1.2821120000
H	2.0166440000	0.9145140000	1.4775680000
H	-2.9383160000	-0.5559310000	1.4715220000
H	-2.3909050000	-1.8088150000	0.3571000000
H	1.8916400000	-1.4871810000	1.2796400000
H	3.5595220000	-0.9407580000	1.1860700000
H	-2.3787980000	1.9354610000	0.9581230000
H	-3.2045740000	1.8088200000	-0.6139770000
H	-1.5184330000	2.3413430000	-0.5464970000
H	4.1788980000	1.2879860000	0.2537780000
H	3.3390000000	1.0692370000	-1.3003370000
H	2.9111160000	2.4327610000	-0.2484290000
H	-4.7091360000	0.1647470000	-0.1834650000
H	-4.0890950000	-1.0092720000	-1.3669850000
H	-4.8350690000	-1.5735090000	0.1460000000
H	1.8554310000	-1.8703330000	-1.2254350000
H	3.1616100000	-2.7745520000	-0.4358010000
H	3.5326020000	-1.2917650000	-1.3372710000

3-Ethyl-4-methylheptane

0 1

C	-0.8392320000	0.0758320000	-0.1179380000
C	0.2430180000	-0.6165140000	0.7645390000
C	1.6446490000	0.0152410000	0.6178200000
C	-1.2822980000	1.4471930000	0.4397950000
C	-2.0578820000	-0.8396020000	-0.3744220000
C	-0.1502660000	-0.6904760000	2.2495110000
C	2.3326550000	-0.2073880000	-0.7346580000

C	-1.9091950000	2.3755840000	-0.6061050000
C	-1.7923950000	-2.0239720000	-1.3092380000
C	3.7403820000	0.3923190000	-0.7913930000
H	-0.3758960000	0.2657590000	-1.1041700000
H	0.3324310000	-1.6565440000	0.4013300000
H	1.5916280000	1.1001270000	0.8281250000
H	2.2953270000	-0.4042830000	1.4072840000
H	-1.9958800000	1.2939450000	1.2694570000
H	-0.4140950000	1.9644620000	0.8826880000
H	-2.8713530000	-0.2340250000	-0.8088430000
H	-2.4487920000	-1.2094460000	0.5909060000
H	-1.1563590000	-1.1149670000	2.3946770000
H	0.5578140000	-1.3263740000	2.8055290000
H	-0.1371040000	0.3044090000	2.7245360000
H	1.7245390000	0.2234630000	-1.5492730000
H	2.3866190000	-1.2929750000	-0.9382910000
H	-2.2144430000	3.3340190000	-0.1555280000
H	-1.1945650000	2.6014980000	-1.4160620000
H	-2.8053500000	1.9341270000	-1.0722460000
H	-1.4414250000	-1.6800600000	-2.2974740000
H	-2.7094460000	-2.6139550000	-1.4698770000
H	-1.0291450000	-2.7118510000	-0.9100990000
H	4.2182790000	0.2134390000	-1.7682200000
H	4.3931350000	-0.0432890000	-0.0155330000
H	3.7174710000	1.4832200000	-0.6263840000

3-Ethyloctane

0 1

C	1.5113350000	-0.3252230000	0.3371110000
C	0.1536040000	-0.5258890000	-0.3678130000
C	-1.0712500000	0.0060660000	0.3857570000
C	1.8397070000	1.1577150000	0.6299870000
C	2.6227100000	-1.0537540000	-0.4478970000
C	-2.4000040000	-0.3581850000	-0.2861030000
C	-3.6321360000	0.1756820000	0.4522100000
C	1.9933560000	2.0662370000	-0.5953060000
C	3.9991220000	-1.0635320000	0.2236290000
C	-4.9538290000	-0.2004740000	-0.2215070000
H	1.4375870000	-0.8240950000	1.3242070000
H	0.0162560000	-1.6097020000	-0.5380430000
H	0.1870320000	-0.0727040000	-1.3762110000
H	-1.0654140000	-0.3906920000	1.4189340000
H	-1.0112140000	1.1048990000	0.4835190000
H	1.0548120000	1.5702560000	1.2867360000
H	2.7664590000	1.2019200000	1.2273120000
H	2.7091530000	-0.6165320000	-1.4595210000
H	2.2968420000	-2.0974510000	-0.6058970000
H	-2.4763580000	-1.4588000000	-0.3733010000
H	-2.4024590000	0.0262740000	-1.3239740000
H	-3.5590540000	1.2761270000	0.5333000000
H	-3.6261020000	-0.2041120000	1.4908060000
H	1.0718800000	2.1087200000	-1.1991680000
H	2.8072200000	1.7325820000	-1.2600720000
H	2.2277600000	3.0979850000	-0.2862520000
H	3.9425610000	-1.4730100000	1.2471140000
H	4.4361790000	-0.0544820000	0.2956630000
H	4.7097050000	-1.6864610000	-0.3435350000
H	-5.8192050000	0.1980110000	0.3324410000
H	-5.0056550000	0.1951370000	-1.2503780000
H	-5.0736700000	-1.2957230000	-0.2840970000

3-Isopropyl-2-methylhexane

0 1

C	-0.2001100000	0.0135100000	-0.3977120000
C	-0.6330900000	-1.4594810000	-0.1225780000
C	-1.2842500000	1.0860890000	-0.0545720000
C	1.1819320000	0.3553870000	0.2010370000
C	2.3733620000	-0.3516780000	-0.4597120000
C	-1.0404000000	-1.7693610000	1.3262180000
C	-1.7309640000	-1.9239910000	-1.0929550000
C	-1.2191760000	2.2642370000	-1.0409190000
C	-1.2446510000	1.6200120000	1.3886980000
C	3.7200040000	0.0838980000	0.1242490000
H	-0.0740480000	0.0635690000	-1.4961580000
H	0.2543720000	-2.0793400000	-0.3428490000
H	-2.2699000000	0.6090240000	-0.1973960000

H	1.3478470000	1.4437440000	0.1113660000
H	1.1928170000	0.1445210000	1.2855590000
H	2.3589390000	-0.1456160000	-1.5461710000
H	2.2753450000	-1.4467000000	-0.3581690000
H	-0.2749810000	-1.4567190000	2.0541000000
H	-1.1907680000	-2.8541250000	1.4556590000
H	-1.9883840000	-1.2770260000	1.5989860000
H	-1.4298890000	-1.7752910000	-2.1431480000
H	-1.9477230000	-2.9962410000	-0.9553320000
H	-2.6780310000	-1.3799500000	-0.9370080000
H	-0.2420070000	2.7759390000	-0.9938230000
H	-1.3704110000	1.9300280000	-2.0803700000
H	-1.9926070000	3.0166120000	-0.8134410000
H	-0.3387080000	2.2193660000	1.5763020000
H	-1.2744090000	0.8183860000	2.1401220000
H	-2.1103010000	2.2781160000	1.5720310000
H	4.5608000000	-0.4301470000	-0.3690950000
H	3.8762970000	1.1698270000	0.0050320000
H	3.7798040000	-0.1397080000	1.2032740000

3-Ethyl-3-methylheptane

0 1

C	1.0188220000	0.0067590000	0.2828790000
C	-0.4376460000	0.0438280000	0.8260760000
C	1.3256840000	-1.4286340000	-0.2310040000
C	1.2249870000	1.0231910000	-0.8738340000
C	1.9535240000	0.3455910000	1.4627810000
C	-1.5802200000	-0.2284530000	-0.1611720000
C	-2.9619610000	-0.1585360000	0.5012120000
C	2.7647770000	-1.7255120000	-0.6702320000
C	0.8818560000	2.4875130000	-0.5753810000
C	-4.1135360000	-0.4334280000	-0.4685010000
H	-0.6113840000	1.0284700000	1.2955380000
H	-0.5061010000	-0.6884600000	1.6522650000
H	1.0494710000	-2.1402590000	0.5689170000
H	0.6532540000	-1.6509060000	-1.0771750000
H	2.2801570000	0.9735390000	-1.1929060000
H	0.6364300000	0.6902530000	-1.7472200000
H	3.0049030000	0.4327050000	1.1462280000
H	1.6719210000	1.2980830000	1.9386680000
H	1.9005700000	-0.4350820000	2.2403330000
H	-1.5478850000	0.4990740000	-0.9923220000
H	-1.4595440000	-1.2240270000	-0.6237940000
H	-3.0957010000	0.8384720000	0.9605830000
H	-3.0019940000	-0.8823660000	1.3363150000
H	2.8482240000	-2.7684980000	-1.0169060000
H	3.0898750000	-1.0814040000	-1.5030820000
H	3.4867830000	-1.5977370000	0.1518400000
H	1.4716990000	2.8883110000	0.2647280000
H	1.0974030000	3.1179220000	-1.4534970000
H	-0.1832090000	2.6265640000	-0.3288060000
H	-4.0279230000	-1.4381240000	-0.9168920000
H	-4.1233520000	0.2961050000	-1.2964880000
H	-5.0913240000	-0.3765520000	0.0367300000

3-Ethyl-5-methylheptane

0 1

C	1.0135170000	-0.3707890000	-0.0514050000
C	-0.1402160000	-0.0716290000	0.9310440000
C	-1.5282790000	0.2611520000	0.3442460000
C	2.2776610000	-0.7508950000	0.7500160000
C	1.2564100000	0.7579190000	-1.0802760000
C	-2.0928720000	-0.8973390000	-0.5018850000
C	-2.4808540000	0.6570370000	1.4830570000
C	3.4673660000	-1.2234350000	-0.0903260000
C	1.6683500000	2.1178380000	-0.5039660000
C	-3.4147600000	-0.5949700000	-1.2151460000
H	0.7366540000	-1.2673230000	-0.6375610000
H	-0.2524440000	-0.9451370000	1.6011300000
H	0.1640230000	0.7620620000	1.5898360000
H	-1.4258830000	1.1404290000	-0.3202670000
H	2.0008960000	-1.5535540000	1.4567410000
H	2.5869480000	0.1022710000	1.3811430000
H	2.0288260000	0.4225920000	-1.7935140000
H	0.3430790000	0.8874200000	-1.6860900000
H	-2.2173830000	-1.7843710000	0.1487650000

H	-1.3466950000	-1.1842680000	-1.2619700000
H	-2.6494270000	-0.1921280000	2.1690350000
H	-3.4630270000	0.9850070000	1.1096210000
H	-2.0619900000	1.4848600000	2.0785030000
H	3.1841320000	-2.0616890000	-0.7502810000
H	4.2905620000	-1.5729270000	0.5538050000
H	3.8720050000	-0.4221140000	-0.7294770000
H	1.8193790000	2.8518620000	-1.3122670000
H	2.6121160000	2.0585290000	0.0626760000
H	0.9027220000	2.5309740000	0.1731050000
H	-4.2400540000	-0.4147970000	-0.5080610000
H	-3.3266710000	0.2991360000	-1.8565340000
H	-3.7157020000	-1.4361710000	-1.8605000000

3-Methylnonane

0 1

C	-2.3299060000	0.1530740000	-0.1883580000
C	-1.0017180000	-0.4535480000	0.3056370000
C	0.2718670000	0.2574150000	-0.1677560000
C	1.5577270000	-0.4600500000	0.2589540000
C	-3.5038250000	-0.7792820000	0.1730950000
C	2.8389640000	0.2387270000	-0.2071810000
C	-2.5454920000	1.5770470000	0.3446720000
C	4.1236420000	-0.4843030000	0.2115470000
C	-4.8525230000	-0.4043970000	-0.4479710000
C	5.3979800000	0.2190740000	-0.2612060000
H	-2.2792730000	0.2071160000	-1.2946350000
H	-1.0122260000	-0.4876140000	1.4124910000
H	-0.9560910000	-1.5074060000	-0.0260640000
H	0.2966650000	1.2925710000	0.2165000000
H	0.2524950000	0.3430870000	-1.2710100000
H	1.5738840000	-0.5547010000	1.3613420000
H	1.5443390000	-1.4954370000	-0.1312120000
H	-3.6019180000	-0.8197020000	1.2748140000
H	-3.2418010000	-1.8050390000	-0.1431730000
H	2.8572150000	1.2722140000	0.1883270000
H	2.8205580000	0.3388120000	-1.3092670000
H	-3.4809200000	2.0180190000	-0.0331820000
H	-2.5975730000	1.5775830000	1.4483640000
H	-1.7282220000	2.2530260000	0.0491800000
H	4.1429140000	-0.5827930000	1.3128220000
H	4.1039840000	-1.5171230000	-0.1831650000
H	-4.7833180000	-0.3478040000	-1.5481350000
H	-5.2227490000	0.5694310000	-0.0892270000
H	-5.6214220000	-1.1549930000	-0.2029310000
H	5.4248900000	0.3015320000	-1.3613280000
H	6.3026730000	-0.3255370000	0.0541550000
H	5.4655920000	1.2422100000	0.1467270000

4,4-Dimethyloctane

0 1

C	0.5827250000	0.7994100000	-0.0164090000
C	-0.5055450000	-0.2870010000	-0.2303690000
C	1.9689160000	0.2339180000	-0.4347570000
C	-1.9630470000	0.1110410000	0.0396360000
C	0.6185290000	1.2604460000	1.4544680000
C	0.3038550000	2.0220930000	-0.9134890000
C	2.5155190000	-0.9790030000	0.3301090000
C	-2.9434290000	-1.0479530000	-0.1794100000
C	3.9003230000	-1.4022240000	-0.1696480000
C	-4.4022220000	-0.6644410000	0.0792220000
H	-0.4288770000	-0.6491900000	-1.2732500000
H	-0.2666850000	-1.1563910000	0.4074400000
H	1.9251720000	-0.0215200000	-1.5105440000
H	2.7064370000	1.0545390000	-0.3534140000
H	-2.0712610000	0.4765970000	1.0767520000
H	-2.2576420000	0.9519120000	-0.6130550000
H	0.7363360000	0.4135270000	2.1493870000
H	1.4584130000	1.9547410000	1.6263890000
H	-0.3057860000	1.7905280000	1.7336770000
H	0.2425190000	1.7320470000	-1.9761590000
H	1.1111570000	2.7673740000	-0.8185690000
H	-0.6390100000	2.5236640000	-0.6456610000
H	2.5756040000	-0.7504780000	1.4084840000
H	1.8235940000	-1.8335370000	0.2355210000
H	-2.6618180000	-1.8926790000	0.4763980000

H	-2.8383730000	-1.4225250000	-1.2146230000
H	4.2814700000	-2.2740840000	0.3862660000
H	3.8745910000	-1.6733760000	-1.2390540000
H	4.6345570000	-0.5863480000	-0.0555480000
H	-4.7250710000	0.1542490000	-0.5866290000
H	-5.0810520000	-1.5164560000	-0.0874780000
H	-4.5480250000	-0.3203130000	1.1175500000

4,5-Dimethyloctane

0 1

C	0.8239610000	1.1708860000	0.0970060000
C	-0.4658780000	0.6418020000	-0.5969390000
C	1.8110810000	0.0612220000	0.5193480000
C	-1.3869010000	-0.1567130000	0.3486570000
C	0.5391190000	2.0929110000	1.2939640000
C	-1.2229150000	1.7753530000	-1.3086830000
C	2.3477120000	-0.8284640000	-0.6090390000
C	-2.4818690000	-0.9726240000	-0.3522330000
C	3.4441210000	-1.7873970000	-0.1370370000
C	-3.3172090000	-1.8072430000	0.6222580000
H	1.3463180000	1.7791980000	-0.6668520000
H	-0.1288530000	-0.0554910000	-1.3856200000
H	1.3520420000	-0.5753450000	1.2988590000
H	2.6715300000	0.5498630000	1.0133340000
H	-0.7777620000	-0.8533540000	0.9518910000
H	-1.8618570000	0.5317350000	1.0720330000
H	1.4745680000	2.5401840000	1.6679300000
H	-0.1382050000	2.9204300000	1.0317270000
H	0.0842940000	1.5405840000	2.1332460000
H	-0.5509330000	2.3457660000	-1.9712240000
H	-2.0427630000	1.3857120000	-1.9317180000
H	-1.6669560000	2.4863460000	-0.5916440000
H	2.7425150000	-0.1890800000	-1.4207500000
H	1.5264390000	-1.4145400000	-1.0570580000
H	-2.0150320000	-1.6388490000	-1.1015680000
H	-3.1516890000	-0.3018860000	-0.9177910000
H	3.0743730000	-2.4591190000	0.6567180000
H	3.8127460000	-2.4186690000	-0.9617320000
H	4.3075510000	-1.2371820000	0.2745340000
H	-3.8178130000	-1.1670440000	1.3688310000
H	-2.6891200000	-2.5268160000	1.1750310000
H	-4.0987230000	-2.3808450000	0.0980090000

4-Ethyl-3-methylheptane

0 1

C	-0.1785220000	0.6225070000	0.2686680000
C	1.0595470000	-0.3012950000	0.4799470000
C	-1.3719210000	-0.1295920000	-0.3606890000
C	0.1585410000	1.9068770000	-0.5216520000
C	1.8184260000	-0.6127530000	-0.8284290000
C	1.9976030000	0.2580580000	1.5614730000
C	-2.1064790000	-1.0914180000	0.5824820000
C	-0.8421030000	3.0494070000	-0.3173300000
C	2.7753340000	-1.8060050000	-0.7349250000
C	-3.2730190000	-1.8168770000	-0.0930370000
H	-0.4988220000	0.9431090000	1.2794080000
H	0.6726680000	-1.2640860000	0.8624230000
H	-1.0294910000	-0.6845280000	-1.2541710000
H	-2.1057550000	0.6052160000	-0.7359640000
H	1.1584630000	2.2682100000	-0.2273070000
H	0.2287850000	1.6675650000	-1.5980020000
H	1.0903030000	-0.8146930000	-1.6329500000
H	2.3822890000	0.2814600000	-1.1494420000
H	2.4289850000	1.2294260000	1.2650540000
H	1.4610660000	0.4090390000	2.5125910000
H	2.8380890000	-0.4241050000	1.7634800000
H	-2.4811250000	-0.5242390000	1.4546610000
H	-1.4022840000	-1.8390310000	0.9887350000
H	-0.8860040000	3.3533220000	0.7426270000
H	-0.5586100000	3.9368060000	-0.9063140000
H	-1.8639630000	2.7688660000	-0.6214030000
H	3.2587370000	-2.0026890000	-1.7057150000
H	2.2400170000	-2.7244770000	-0.4385720000
H	3.5785900000	-1.6402800000	0.0012850000
H	-2.9263740000	-2.4257850000	-0.9456320000
H	-3.7916880000	-2.4906770000	0.6080890000

H	-4.0183150000	-1.1018870000	-0.4819240000

4-Ethyl-4-methylheptane

0 1

C	0.1522920000	-0.7194370000	0.1759630000
C	-1.2315560000	-0.4997250000	-0.4976120000
C	0.7831180000	0.6176050000	0.6488040000
C	1.0456730000	-1.4367790000	-0.8763460000
C	-0.0148670000	-1.6295620000	1.4101010000
C	-2.3071190000	0.2496480000	0.2998040000
C	1.0406620000	1.6904790000	-0.4167580000
C	2.4980730000	-1.7286560000	-0.4843370000
C	-3.6360580000	0.3353070000	-0.4574640000
C	1.6531410000	2.9640730000	0.1740930000
H	-1.0799940000	0.0279230000	-1.4566810000
H	-1.6302790000	-1.4943720000	-0.7724320000
H	1.7372890000	0.3918410000	1.1580690000
H	0.1366500000	1.0495230000	1.4338070000
H	0.5501580000	-2.3892020000	-1.1388870000
H	1.0439510000	-0.8411060000	-1.8060710000
H	-0.6642930000	-1.1668900000	2.1698810000
H	-0.4611890000	-2.5994300000	1.1318310000
H	0.9520480000	-1.8325740000	1.8970370000
H	-1.9619500000	1.2714300000	0.5356220000
H	-2.4773000000	-0.2464120000	1.2709180000
H	0.0978360000	1.9484710000	-0.9301890000
H	1.7131710000	1.2951310000	-1.1979700000
H	2.5703190000	-2.3487840000	0.4234890000
H	3.0745350000	-0.8069530000	-0.3038770000
H	3.0085310000	-2.2767040000	-1.2930290000
H	-4.3973540000	0.8820470000	0.1221700000
H	-4.0390000000	-0.6688370000	-0.6744310000
H	-3.5145770000	0.8556760000	-1.4230650000
H	0.9899650000	3.4116030000	0.9341550000
H	2.6193100000	2.7543280000	0.6644140000
H	1.8320080000	3.7255700000	-0.6022400000

4-Ethyloctane

0 1

C	0.5033610000	-0.0471460000	0.4079470000
C	-0.7584120000	-0.5005500000	-0.3560600000
C	1.7627410000	-0.6201090000	-0.2753420000
C	0.5687870000	1.4819840000	0.6341540000
C	-2.0982690000	-0.1410180000	0.2965310000
C	3.0860160000	-0.3872490000	0.4635930000
C	-3.3054590000	-0.7437050000	-0.4316590000
C	0.6491340000	2.3445610000	-0.6309180000
C	4.2685290000	-1.1014960000	-0.1966890000
C	-4.6460340000	-0.3795000000	0.2103890000
H	0.4495670000	-0.5046490000	1.4159470000
H	-0.7093690000	-1.5991440000	-0.4706320000
H	-0.7345510000	-0.0979360000	-1.3859480000
H	1.8444840000	-0.2191580000	-1.3031730000
H	1.6180850000	-1.7097060000	-0.3968730000
H	-0.3125530000	1.7893450000	1.2226170000
H	1.4364770000	1.7071280000	1.2774600000
H	-2.2215330000	0.9561860000	0.3390330000
H	-2.0981870000	-0.4866720000	1.3481450000
H	2.9847550000	-0.7320770000	1.5096640000
H	3.3043700000	0.6935150000	0.5204540000
H	-3.1982370000	-1.8437780000	-0.4649640000
H	-3.2980110000	-0.4084740000	-1.4855310000
H	-0.2243800000	2.1989180000	-1.2874250000
H	0.6860780000	3.4146260000	-0.3692370000
H	1.5498190000	2.1233390000	-1.2270610000
H	4.4125200000	-0.7593840000	-1.2359740000
H	4.1100550000	-2.1930780000	-0.2294650000
H	5.2088350000	-0.9170120000	0.3476600000
H	-5.4920070000	-0.8280900000	-0.3351810000
H	-4.6985330000	-0.7317120000	1.2548950000
H	-4.8000520000	0.7131870000	0.2239170000

4-Isopropylheptane

0 1

C	0.1479880000	0.1020970000	-0.0743980000

C	0.1009250000	1.6491150000	-0.2526770000
C	-1.1984590000	-0.4757280000	0.4148070000
C	1.3081060000	-0.3632070000	0.8364650000
C	-2.3276380000	-0.4788460000	-0.6235040000
C	1.7617350000	-1.8202060000	0.6337660000
C	1.2208350000	2.1546150000	-1.1746070000
C	0.1016030000	2.4268440000	1.0729640000
C	-3.6292580000	-1.0816720000	-0.0881870000
C	2.5171850000	-2.0700360000	-0.6758060000
H	0.3266040000	-0.3120770000	-1.0844600000
H	-0.8519730000	1.8778380000	-0.7631160000
H	-1.0412410000	-1.5167380000	0.7486140000
H	-1.5297080000	0.0737560000	1.3158440000
H	1.0081790000	-0.2261180000	1.8906730000
H	2.1875930000	0.2879060000	0.6878890000
H	-1.9977250000	-1.0464340000	-1.5134780000
H	-2.5246500000	0.5487000000	-0.9766660000
H	0.8922210000	-2.4995210000	0.6925540000
H	2.4149730000	-2.1020230000	1.4784200000
H	2.2207280000	1.9751360000	-0.7444550000
H	1.1305060000	3.2401420000	-1.3453690000
H	1.1872070000	1.6568300000	-2.1578740000
H	-0.6984200000	2.0927240000	1.7528930000
H	1.0603370000	2.3198560000	1.6068080000
H	-0.0520840000	3.5030380000	0.8897100000
H	-3.4820680000	-2.1244690000	0.2416250000
H	-4.0062700000	-0.5131200000	0.7791750000
H	-4.4207770000	-1.0830360000	-0.8550330000
H	2.8404200000	-3.1208930000	-0.7547830000
H	1.8979860000	-1.8510130000	-1.5613720000
H	3.4200260000	-1.4383500000	-0.7405380000

4-Methylnonane

0 1

C	1.3077850000	0.9719530000	0.4068170000
C	-0.1576990000	0.8026850000	0.8684190000
C	1.9971730000	-0.3838890000	0.1521800000
C	-1.1229930000	0.1518940000	-0.1317360000
C	-2.5548850000	0.0427510000	0.4047020000
C	1.4285380000	1.9250940000	-0.7929550000
C	3.5244660000	-0.3220560000	0.0228160000
C	-3.5353040000	-0.6031420000	-0.5800150000
C	4.1639980000	-1.7010630000	-0.1592940000
C	-4.9626320000	-0.7047100000	-0.0371720000
H	1.8379780000	1.4467320000	1.2551090000
H	-0.1630360000	0.2094520000	1.8018000000
H	-0.5528730000	1.7983810000	1.1432650000
H	1.5791330000	-0.8477380000	-0.7608300000
H	1.7447870000	-1.0697930000	0.9828110000
H	-0.7637010000	-0.8578880000	-0.4005730000
H	-1.1379690000	0.7303960000	-1.0730900000
H	-2.5496960000	-0.5376070000	1.3471610000
H	-2.9219070000	1.0513030000	0.6751150000
H	2.4792090000	2.1696750000	-1.0142200000
H	0.9938990000	1.4866810000	-1.7073560000
H	0.9051320000	2.8759170000	-0.5975640000
H	3.8049830000	0.3218610000	-0.8290210000
H	3.9453770000	0.1636710000	0.9228970000
H	-3.1696550000	-1.6116990000	-0.8483980000
H	-3.5394200000	-0.0233740000	-1.5217570000
H	3.7880970000	-2.1991070000	-1.0694810000
H	5.2603310000	-1.6308770000	-0.2469660000
H	3.9389500000	-2.3631870000	0.6943870000
H	-5.3700330000	0.2915850000	0.2062510000
H	-5.6420680000	-1.1727560000	-0.7678260000
H	-4.9981980000	-1.3087340000	0.8857120000

4-n-Propylheptane

0 1

C	-0.0000050000	-0.4860210000	0.4462590000
C	0.0000040000	1.0375150000	0.7142210000
C	-1.2629250000	-0.9820030000	-0.2891590000
C	1.2629100000	-0.9820150000	-0.2891570000
C	0.0000220000	1.9482510000	-0.5212820000
C	-2.5963700000	-0.7044530000	0.4152590000
C	2.5963580000	-0.7044720000	0.4152600000

C	0.0000210000	3.4347190000	-0.1530860000
C	-3.7892850000	-1.3469450000	-0.2979200000
C	3.7892680000	-1.3469710000	-0.2979200000
H	-0.0000090000	-0.9718190000	1.4422460000
H	0.8762290000	1.2887520000	1.3377300000
H	-0.8762260000	1.2887670000	1.3377160000
H	-1.2941510000	-0.5546660000	-1.3090130000
H	-1.1655350000	-2.0742080000	-0.4324340000
H	1.1655130000	-2.0742210000	-0.4324280000
H	1.2941400000	-0.5546820000	-1.3090120000
H	0.8815690000	1.7304290000	-1.1498800000
H	-0.8815090000	1.7304340000	-1.1499030000
H	-2.7666900000	0.3836360000	0.4925570000
H	-2.5434830000	-1.0780280000	1.4549680000
H	2.5434690000	-1.0780460000	1.4549700000
H	2.7666830000	0.3836160000	0.4925570000
H	0.0000420000	4.0754480000	-1.0497940000
H	0.8886680000	3.6988400000	0.4456840000
H	-0.8886480000	3.6988490000	0.4456480000
H	-3.8856820000	-0.9731610000	-1.3318100000
H	-3.6795590000	-2.4435390000	-0.3543370000
H	-4.7365520000	-1.1326220000	0.2229120000
H	3.8856610000	-0.9731970000	-1.3318130000
H	3.6795400000	-2.4435660000	-0.3543240000
H	4.7365380000	-1.1326440000	0.2229070000

5-Methylnonane

0 1

C	0.0017330000	1.6236330000	0.3782690000
C	-1.1271760000	0.9648240000	-0.4409550000
C	1.0612140000	0.6295690000	0.9084320000
C	-1.9338890000	-0.1122370000	0.2922110000
C	1.8310830000	-0.1765050000	-0.1461280000
C	0.6385850000	2.7744440000	-0.4152830000
C	-3.1209400000	-0.6401990000	-0.5219990000
C	2.8766430000	-1.1179970000	0.4639990000
C	-3.9205730000	-1.7254180000	0.2023280000
C	3.6524970000	-1.9201420000	-0.5833250000
H	-0.4713340000	2.0702780000	1.2742120000
H	-1.8202500000	1.7623010000	-0.7673210000
H	-0.7103830000	0.5389710000	-1.3728440000
H	0.5778740000	-0.0750610000	1.6083900000
H	1.7871190000	1.1976060000	1.5194350000
H	-2.3042980000	0.2967650000	1.2518230000
H	-1.2797750000	-0.9622500000	0.5588290000
H	1.1271860000	-0.7737560000	-0.7540260000
H	2.3368420000	0.5071710000	-0.8517570000
H	-0.1141380000	3.5351070000	-0.6801400000
H	1.0902340000	2.4205040000	-1.3571330000
H	1.4299810000	3.2750990000	0.1669890000
H	-2.7520160000	-1.0358660000	-1.4865820000
H	-3.7898130000	0.2023150000	-0.7782890000
H	2.3767070000	-1.8117510000	1.1651720000
H	3.5841070000	-0.5285210000	1.0762450000
H	-4.7674740000	-2.0795600000	-0.4075260000
H	-3.2894440000	-2.6001000000	0.4357670000
H	-4.3300860000	-1.3515010000	1.1564010000
H	4.3941220000	-2.5879500000	-0.1158570000
H	4.1938810000	-1.2537560000	-1.2766930000
H	2.9758960000	-2.5458760000	-1.1903960000

n-Decane

0 1

C	0.0001450000	0.7661830000	0.0000000000
C	-0.0001450000	-0.7661830000	0.0000000000
C	-1.4022300000	1.3833970000	0.0000000000
C	1.4022300000	-1.3833970000	0.0000000000
C	-1.4022300000	2.9155310000	0.0000000000
C	1.4022300000	-2.9155310000	0.0000000000
C	-2.8044410000	3.5335290000	0.0000000000
C	2.8044410000	-3.5335290000	0.0000000000
C	-2.7924970000	5.0639500000	0.0000000000
C	2.7924970000	-5.0639500000	0.0000000000
H	0.5595000000	1.1308820000	0.8823490000
H	0.5595000000	1.1308820000	-0.8823490000
H	-0.5595000000	-1.1308820000	0.8823490000

H	-0.5595000000	-1.1308820000	-0.8823490000
H	-1.9614900000	1.0185260000	-0.8823780000
H	-1.9614900000	1.0185260000	0.8823780000
H	1.9614900000	-1.0185260000	-0.8823780000
H	1.9614900000	-1.0185260000	0.8823780000
H	-0.8431810000	3.2810770000	0.8824960000
H	-0.8431810000	3.2810770000	-0.8824960000
H	0.8431810000	-3.2810770000	0.8824960000
H	0.8431810000	-3.2810770000	-0.8824960000
H	-3.3625180000	3.1684560000	-0.8821120000
H	-3.3625180000	3.1684560000	0.8821120000
H	3.3625180000	-3.1684560000	-0.8821120000
H	3.3625180000	-3.1684560000	0.8821120000
H	-3.8137790000	5.4780130000	0.0000000000
H	-2.2719110000	5.4598000000	0.8888970000
H	-2.2719110000	5.4598000000	-0.8888970000
H	3.8137790000	-5.4780130000	0.0000000000
H	2.2719110000	-5.4598000000	0.8888970000
H	2.2719110000	-5.4598000000	-0.8888970000

Optimized Geometries at the UFF

Pentacontane

0 1

C	-0.632437	-0.433307	0.000000
C	-1.895566	0.435842	0.000000
C	-3.160407	-0.430821	0.000000
C	-4.423502	0.438353	0.000000
C	-5.688422	-0.428184	0.000000
C	-6.951399	0.441181	0.000000
C	-8.216457	-0.425167	0.000000
C	-9.479342	0.444354	0.000000
C	-10.744554	-0.421779	0.000000
C	-12.007304	0.447973	0.000000
C	-13.272596	-0.418013	0.000000
C	-14.535358	0.451716	0.000000
C	-15.800487	-0.414508	0.000000
C	-17.063485	0.454842	0.000000
C	-18.328337	-0.411794	0.000000
C	-19.591600	0.457176	0.000000
C	-20.856161	-0.409874	0.000000
C	-22.119780	0.458565	0.000000
C	-23.383912	-0.409140	0.000000
C	-24.647980	0.458614	0.000000
C	-25.911728	-0.409683	0.000000
C	-27.176103	0.457670	0.000000
C	-28.439712	-0.410725	0.000000
C	-29.703358	0.457076	0.000000
C	-30.963341	-0.403628	0.000000
C	0.632437	0.433307	0.000000
C	1.895566	-0.435842	0.000000
C	3.160407	0.430821	0.000000
C	4.423502	-0.438353	0.000000
C	5.688422	0.428184	0.000000
C	6.951399	-0.441181	0.000000
C	8.216457	0.425167	0.000000
C	9.479342	-0.444354	0.000000
C	10.744554	0.421779	0.000000
C	12.007304	-0.447973	0.000000
C	13.272596	0.418013	0.000000
C	14.535358	-0.451716	0.000000
C	15.800487	0.414508	-0.000000
C	17.063485	-0.454842	-0.000000
C	18.328337	0.411794	-0.000000
C	19.591600	-0.457176	-0.000000
C	20.856161	0.409874	0.000000
C	22.119780	-0.458565	0.000000
C	23.383912	0.409140	0.000000
C	24.647980	-0.458614	0.000000
C	25.911728	0.409683	0.000000
C	27.176103	-0.457670	0.000000
C	28.439712	0.410725	0.000000
C	29.703358	-0.457076	0.000000
C	30.963341	0.403628	0.000000
H	-0.633102	-1.083022	0.903030
H	-0.633102	-1.083022	-0.903030

H	-1.894937	1.085572	0.903024
H	-1.894937	1.085572	-0.903024
H	-3.161059	-1.080538	-0.903030
H	-3.161059	-1.080538	0.903030
H	-4.422821	1.088072	0.903028
H	-4.422821	1.088072	-0.903028
H	-5.689148	-1.077900	-0.903032
H	-5.689148	-1.077900	0.903032
H	-6.950604	1.090912	0.903022
H	-6.950604	1.090912	-0.903022
H	-8.217254	-1.074879	-0.903031
H	-8.217254	-1.074879	0.903031
H	-9.478470	1.094080	0.903020
H	-9.478470	1.094080	-0.903020
H	-10.745478	-1.071503	-0.903029
H	-10.745478	-1.071503	0.903029
H	-12.006355	1.097692	0.903026
H	-12.006355	1.097692	-0.903026
H	-13.273554	-1.067738	-0.903024
H	-13.273554	-1.067738	0.903024
H	-14.534474	1.101435	0.903028
H	-14.534474	1.101435	-0.903028
H	-15.801278	-1.064237	-0.903020
H	-15.801278	-1.064237	0.903020
H	-17.062800	1.104549	0.903035
H	-17.062800	1.104549	-0.903035
H	-18.328931	-1.061533	-0.903024
H	-18.328931	-1.061533	0.903024
H	-19.591113	1.106882	0.903032
H	-19.591113	1.106882	-0.903032
H	-20.856530	-1.059608	-0.903021
H	-20.856530	-1.059608	0.903021
H	-22.119580	1.108276	0.903033
H	-22.119580	1.108276	-0.903033
H	-23.383945	-1.058875	-0.903019
H	-23.383945	-1.058875	0.903019
H	-24.648100	1.108320	0.903036
H	-24.648100	1.108320	-0.903036
H	-25.911478	-1.059421	-0.903020
H	-25.911478	-1.059421	0.903020
H	-27.176412	1.107394	0.903031
H	-27.176412	1.107394	-0.903031
H	-28.439782	-1.060411	-0.903093
H	-28.439782	-1.060411	0.903093
H	-29.712068	1.106910	0.902216
H	-29.712068	1.106910	-0.902216
H	-30.992111	-1.045717	0.906079
H	-31.860432	0.250740	0.000000
H	-30.992111	-1.045717	-0.906079
H	0.633102	1.083022	-0.903030
H	0.633102	1.083022	0.903030
H	1.894937	-1.085572	0.903024
H	1.894937	-1.085572	-0.903024
H	3.161059	1.080538	-0.903030
H	3.161059	1.080538	0.903030
H	4.422821	-1.088072	0.903028
H	4.422821	-1.088072	-0.903028
H	5.689148	1.077900	-0.903032
H	5.689148	1.077900	0.903032
H	6.950604	-1.090912	0.903022
H	6.950604	-1.090912	-0.903022
H	8.217254	1.074879	-0.903031
H	8.217254	1.074879	0.903031
H	9.478470	-1.094080	0.903020
H	9.478470	-1.094080	-0.903020
H	10.745478	1.071503	-0.903029
H	10.745478	1.071503	0.903029
H	12.006355	-1.097692	0.903026
H	12.006355	-1.097692	-0.903026
H	13.273554	1.067738	-0.903024
H	13.273554	1.067738	0.903024
H	14.534474	-1.101435	0.903028
H	14.534474	-1.101435	-0.903028
H	15.801278	1.064237	-0.903020
H	15.801278	1.064237	0.903020
H	17.062800	-1.104549	0.903035
H	17.062800	-1.104549	-0.903035
H	18.328931	1.061533	-0.903024
H	18.328931	1.061533	0.903024

H	19.591113	-1.106882	0.903032
H	19.591113	-1.106882	-0.903032
H	20.856530	1.059608	-0.903021
H	20.856530	1.059608	0.903021
H	22.119580	-1.108276	0.903033
H	22.119580	-1.108276	-0.903033
H	23.383945	1.058875	-0.903019
H	23.383945	1.058875	0.903019
H	24.648100	-1.108320	0.903036
H	24.648100	-1.108320	-0.903036
H	25.911478	1.059421	-0.903020
H	25.911478	1.059421	0.903020
H	27.176412	-1.107394	0.903031
H	27.176412	-1.107394	-0.903031
H	28.439782	1.060411	-0.903093
H	28.439782	1.060411	0.903093
H	29.712068	-1.106910	0.902216
H	29.712068	-1.106910	-0.902216
H	30.992111	1.045717	0.906079
H	31.860432	-0.250740	0.000000
H	30.992111	1.045717	-0.906079

14-Butyl-13-decyl-13-octyloctacosane

0 1

C	-1.543991	0.040754	-2.239472
C	-2.483168	-0.819989	-3.170951
C	-2.502600	-2.345805	-2.921219
C	-3.654549	-3.009020	-3.687085
C	-3.658340	-4.527487	-3.473276
C	-4.813278	-5.187804	-4.235292
C	-4.817224	-6.706001	-4.021538
C	-5.972365	-7.365597	-4.783398
C	-5.979532	-8.876949	-4.573333
C	-1.819662	1.542778	-2.639981
C	-0.871700	2.612534	-2.072426
C	-1.447064	4.021226	-2.270218
C	-0.477202	5.089864	-1.751707
C	-1.052910	6.497347	-1.946656
C	-0.082609	7.565474	-1.428537
C	-0.657766	8.973285	-1.623813
C	0.312615	10.041389	-1.105865
C	-0.262927	11.448615	-1.301703
C	0.700067	12.514654	-0.787340
C	-1.943073	-0.182629	-0.729745
C	-3.447308	-0.106927	-0.380301
C	-3.667500	-0.321641	1.123071
C	-5.158380	-0.260312	1.476977
C	-5.377457	-0.475650	2.979024
C	-6.868336	-0.415144	3.331780
C	-7.088326	-0.630479	4.833788
C	-8.579371	-0.569795	5.185987
C	-8.799866	-0.784787	6.688017
C	-10.290879	-0.723484	7.040017
C	-10.510797	-0.938421	8.541787
C	-11.993750	-0.877393	8.896070
C	-0.024849	-0.412119	-2.476730
C	0.515055	-0.071380	-3.906713
C	1.034225	-1.312875	-4.651862
C	1.645858	-0.930935	-6.004605
C	2.146309	-2.164224	-6.750688
C	1.001543	0.018373	-1.374722
C	2.352138	-0.712350	-1.491606
C	3.274124	-0.363920	-0.315894
C	4.626157	-1.075123	-0.447038
C	5.548346	-0.725505	0.726810
C	6.901274	-1.434594	0.594226
C	7.824196	-1.083526	1.767160
C	9.177364	-1.792180	1.634472
C	10.100355	-1.440586	2.807218
C	11.453511	-2.149335	2.674840
C	12.376243	-1.797946	3.847892
C	13.729314	-2.506818	3.715884
C	14.651025	-2.155357	4.889264
C	15.998881	-2.859164	4.761605
H	-3.522939	-0.433468	-3.094043
H	-2.209313	-0.666225	-4.235616
H	-1.553398	-2.799670	-3.272305
H	-2.620810	-2.574010	-1.843355

H	-4.621636	-2.587583	-3.333801
H	-3.548467	-2.790295	-4.772729
H	-2.693229	-4.951549	-3.828695
H	-3.761925	-4.745638	-2.387314
H	-5.778434	-4.763878	-3.879783
H	-4.709774	-4.969499	-5.321219
H	-3.852282	-7.130231	-4.377311
H	-4.920662	-6.924496	-2.935615
H	-6.940919	-6.949072	-4.430160
H	-5.873691	-7.154340	-5.870593
H	-5.031018	-9.322342	-4.942165
H	-6.102779	-9.116195	-3.495528
H	-6.824979	-9.326377	-5.135661
H	-1.820704	1.659399	-3.743942
H	-2.856404	1.809173	-2.353037
H	-0.698292	2.454426	-0.989274
H	0.093040	2.561512	-2.618573
H	-1.639096	4.192409	-3.352633
H	-2.411413	4.105444	-1.721932
H	-0.284720	4.917756	-0.669604
H	0.487570	5.008123	-2.299559
H	-1.245602	6.669204	-3.028747
H	-2.017370	6.579448	-1.398322
H	0.109846	7.393614	-0.346394
H	0.881964	7.482995	-1.976626
H	-0.850287	9.145081	-2.705956
H	-1.622286	9.055844	-1.075639
H	0.504987	9.870075	-0.023592
H	1.277254	9.959061	-1.653916
H	-0.454433	11.628512	-2.382130
H	-1.225962	11.539616	-0.753395
H	0.888793	12.372784	0.298176
H	1.663726	12.462006	-1.337501
H	0.257459	13.521188	-0.942129
H	-1.565983	-1.173914	-0.396792
H	-1.434088	0.569019	-0.094496
H	-3.861180	0.884342	-0.653280
H	-4.014413	-0.889857	-0.923756
H	-3.261555	-1.315072	1.416274
H	-3.122876	0.464394	1.691517
H	-5.565251	0.732952	1.184656
H	-5.703784	-1.046617	0.909615
H	-4.970272	-1.468760	3.271466
H	-4.832841	0.310966	3.546656
H	-7.275458	0.577937	3.039120
H	-7.412805	-1.201705	2.763902
H	-6.681368	-1.623614	5.126467
H	-6.543906	0.156070	5.401714
H	-8.986307	0.423260	4.892987
H	-9.123700	-1.356437	4.618090
H	-8.393354	-1.778024	6.980985
H	-8.255301	0.001643	7.255983
H	-10.697555	0.269784	6.747282
H	-10.835692	-1.509975	6.472319
H	-10.110336	-1.931585	8.841141
H	-9.971564	-0.153615	9.115953
H	-12.122015	-1.036189	9.987536
H	-12.413504	0.116138	8.629999
H	-12.553005	-1.669398	8.353802
H	-0.032713	-1.515523	-2.372756
H	-0.259333	0.384965	-4.548475
H	1.323644	0.689037	-3.845064
H	1.800538	-1.833877	-4.040357
H	0.188421	-2.014442	-4.819763
H	0.883310	-0.417390	-6.629979
H	2.499054	-0.235654	-5.846798
H	2.929450	-2.682665	-6.157365
H	1.306741	-2.865837	-6.943025
H	2.583185	-1.857761	-7.724411
H	1.202712	1.103612	-1.407065
H	0.609920	-0.220860	-0.367510
H	2.180662	-1.811138	-1.504896
H	2.859654	-0.418181	-2.434528
H	3.438052	0.736137	-0.290908
H	2.789200	-0.670847	0.637057
H	4.462181	-2.175168	-0.471366
H	5.111988	-0.769028	-1.399879
H	5.710723	0.374777	0.751803
H	5.063574	-1.033103	1.679681

H	6.739031	-2.534913	0.570310
H	7.385443	-1.127794	-0.359224
H	7.986134	0.016843	1.791102
H	7.340176	-1.390487	2.720623
H	9.015505	-2.892561	1.610867
H	9.661251	-1.485449	0.680865
H	10.262300	-0.340213	2.830684
H	9.616366	-1.747126	3.760835
H	11.291560	-3.249702	2.651114
H	11.937648	-1.842582	1.721366
H	12.538336	-0.697589	3.871531
H	11.891979	-2.104533	4.801364
H	13.567502	-3.607233	3.692181
H	14.214083	-2.200010	2.762717
H	14.820445	-1.056775	4.917146
H	14.174369	-2.462587	5.845700
H	16.643729	-2.586373	5.623420
H	15.859824	-3.961310	4.756950
H	16.508737	-2.549465	3.824470

9-Tert-butyl-11-heptyl-10-hexyl-11-octyl-10-pentylicosane

0 1

C	-1.166446	-0.446781	-2.622454
C	-2.310099	-0.882665	-3.600801
C	0.098722	-1.050445	-3.282596
C	-1.043295	1.093387	-2.818287
C	-1.470946	-0.800308	-1.024858
C	-3.005859	-1.175505	-0.827918
C	-3.932264	0.058000	-0.718411
C	-5.405469	-0.357610	-0.618165
C	-6.315441	0.868672	-0.482099
C	-7.787180	0.452729	-0.375866
C	-8.697185	1.678962	-0.238888
C	-10.168619	1.262628	-0.132490
C	-11.077639	2.480595	0.003794
C	-0.418747	-1.600322	-0.006486
C	0.771372	-0.607754	0.651513
C	1.857349	-0.163735	-0.403183
C	3.137708	0.519587	0.145911
C	3.844960	1.332295	-0.946507
C	5.133907	1.968779	-0.413834
C	5.835744	2.785318	-1.505115
C	7.123864	3.423720	-0.972320
C	7.825724	4.240618	-2.063522
C	9.113268	4.879022	-1.530117
C	9.814972	5.692780	-2.613521
C	0.140601	0.721527	1.310781
C	0.207943	2.086015	0.576994
C	-0.509948	3.181354	1.379954
C	-0.466839	4.524866	0.641501
C	-1.191857	5.614980	1.439065
C	-1.148649	6.957845	0.700604
C	-1.874974	8.047294	1.497668
C	-1.833287	9.385823	0.766277
C	1.562592	-1.300749	1.859682
C	2.446618	-2.541008	1.583634
C	3.644732	-2.611650	2.543546
C	4.483251	-3.870127	2.288739
C	5.681311	-3.936020	3.242958
C	6.519187	-5.193903	2.987198
C	7.713028	-5.263152	3.934950
C	-1.172977	-2.329062	1.217776
C	-1.933340	-1.519370	2.301716
C	-2.829796	-2.434793	3.148333
C	-3.564485	-1.638167	4.233116
C	-4.462115	-2.544801	5.069812
C	0.211893	-2.848210	-0.769964
C	-0.767861	-3.834159	-1.457844
C	-0.014063	-4.861999	-2.317253
C	-0.990494	-5.793821	-3.044990
C	-0.238163	-6.822049	-3.897138
C	-1.207066	-7.750485	-4.623374
H	-2.455308	-1.981271	-3.609778
H	-3.274775	-0.385569	-3.390081
H	-2.072882	-0.607341	-4.652645
H	0.229008	-0.646105	-4.311319
H	1.034125	-0.789636	-2.784923

H	0.012828	-2.140573	-3.428029
H	-1.840203	1.646239	-2.281669
H	-0.057356	1.469118	-2.491446
H	-1.118084	1.379353	-3.890616
H	-1.458482	0.187227	-0.562866
H	-3.370989	-1.870486	-1.603628
H	-3.242702	-1.758603	0.060121
H	-3.658550	0.637623	0.190338
H	-3.809122	0.732269	-1.586224
H	-5.688260	-0.927129	-1.530854
H	-5.543548	-1.016216	0.267865
H	-6.028304	1.440327	0.428100
H	-6.181821	1.526156	-1.369490
H	-8.074417	-0.118829	-1.286043
H	-7.920352	-0.204985	0.511458
H	-8.409988	2.250523	0.671340
H	-8.564311	2.336789	-1.126172
H	-10.464027	0.693543	-1.040915
H	-10.309877	0.607641	0.754869
H	-12.134702	2.148968	0.078650
H	-10.819643	3.054148	0.919522
H	-10.974440	3.140422	-0.883937
H	1.385187	0.518610	-1.124544
H	2.244263	-1.019618	-0.973923
H	2.920561	1.181027	1.007937
H	3.841185	-0.260718	0.505921
H	4.088644	0.663390	-1.801365
H	3.163891	2.133915	-1.308356
H	4.888061	2.634616	0.442836
H	5.818490	1.168674	-0.054995
H	6.081978	2.119393	-2.361563
H	5.150449	3.584686	-1.864329
H	6.877363	4.089454	-0.115775
H	7.809203	2.624415	-0.613094
H	8.072737	3.574972	-2.920004
H	7.140485	5.039939	-2.422974
H	8.873611	5.549168	-0.675818
H	9.804943	4.085486	-1.172000
H	10.088752	5.040226	-3.469851
H	9.153358	6.510162	-2.971629
H	10.741936	6.142184	-2.199104
H	-0.922603	0.585177	1.511935
H	0.574151	0.911031	2.316949
H	1.248847	2.417886	0.416652
H	-0.284129	2.022361	-0.400923
H	-1.571208	2.887479	1.539486
H	-0.020444	3.291680	2.372688
H	0.592928	4.825444	0.486112
H	-0.952180	4.413382	-0.353438
H	-2.251718	5.314151	1.593502
H	-0.707212	5.726484	2.434187
H	-0.088853	7.259289	0.546874
H	-1.632562	6.846154	-0.294907
H	-2.936620	7.754676	1.651451
H	-1.394766	8.166728	2.493408
H	-2.330140	9.300337	-0.223629
H	-2.366095	10.153718	1.365790
H	-0.781883	9.714279	0.622212
H	2.221708	-0.539543	2.327331
H	0.893099	-1.545112	2.703343
H	1.847740	-3.458135	1.760244
H	2.829241	-2.548002	0.544437
H	4.286544	-1.714226	2.406054
H	3.273474	-2.621519	3.592290
H	3.847845	-4.771713	2.433411
H	4.849908	-3.860838	1.238435
H	6.317311	-3.034818	3.097924
H	5.314989	-3.945190	4.293367
H	5.890780	-6.099408	3.133938
H	6.891766	-5.189758	1.939563
H	8.300017	-6.182436	3.726797
H	8.372099	-4.380623	3.790528
H	7.366823	-5.294118	4.990066
H	-1.877515	-3.086443	0.836975
H	-0.465354	-2.982294	1.765498
H	-1.219713	-1.029952	2.993155
H	-2.577229	-0.738432	1.855731
H	-3.579242	-2.928546	2.490802
H	-2.205317	-3.221373	3.626807

H	-2.825782	-1.146200	4.902846
H	-4.190816	-0.850309	3.760183
H	-5.228076	-3.028814	4.427039
H	-3.856894	-3.328446	5.573464
H	-4.979359	-1.942037	5.845744
H	0.984281	-2.569876	-1.488430
H	0.764214	-3.487038	-0.070011
H	-1.339422	-4.413084	-0.705811
H	-1.493178	-3.295269	-2.086661
H	0.619481	-4.342962	-3.067835
H	0.652062	-5.465526	-1.661785
H	-1.619551	-6.325333	-2.297100
H	-1.655382	-5.188982	-3.700718
H	0.389676	-6.297636	-4.650255
H	0.426678	-7.432668	-3.247943
H	-0.635078	-8.482584	-5.231500
H	-1.830352	-8.306884	-3.891242
H	-1.867735	-7.166576	-5.299149

8,14-Diethyl-9-heptyl-13-hexyl-10,15-dimethyl-11-pentyl-12-propyltricosane

0 1

C	4.922036	-2.134565	-1.191328
C	5.889881	-3.253632	-0.783170
C	4.657592	-1.072994	-0.065477
C	5.578055	0.164561	-0.330417
C	7.081217	-0.147809	-0.226664
C	7.920321	1.134033	-0.314512
C	9.418874	0.817890	-0.240630
C	10.256673	2.099091	-0.325663
C	11.754731	1.782382	-0.252720
C	12.592059	3.055218	-0.337118
C	3.097410	-0.742559	0.041674
C	2.271013	-1.789893	0.930369
C	0.666871	-1.821426	0.774868
C	-0.147583	-1.153730	1.982255
C	-1.742264	-1.336505	2.012154
C	-2.671639	-0.281457	1.249482
C	-4.175939	-0.171781	1.770383
C	-5.104520	0.282489	0.605611
C	-6.348576	1.049529	1.085386
C	-7.172748	1.557035	-0.104465
C	-8.418027	2.315296	0.370241
C	-9.240805	2.822257	-0.820101
C	-10.486886	3.579271	-0.345891
C	-11.309238	4.085150	-1.536569
C	-12.550925	4.838596	-1.068717
C	-4.792920	-1.391246	2.477391
C	-2.103708	1.187340	1.285540
C	-1.283548	1.597908	0.061906
C	-2.128909	-2.855178	1.860276
C	-2.603569	-3.317157	0.468874
C	-3.049968	-4.783788	0.497735
C	-3.469153	-5.256624	-0.899659
C	-3.910695	-6.723969	-0.874835
C	-4.325267	-7.197693	-2.264797
C	0.411074	-1.646620	3.372766
C	1.044290	-0.505321	4.186941
C	1.644273	-1.019407	5.492215
C	0.190120	-1.381146	-0.637012
C	0.521183	-2.409608	-1.737805
C	-0.132405	-2.018236	-3.068167
C	0.196037	-3.038759	-4.163475
C	-0.457767	-2.654970	-5.487470
C	2.792829	-3.244707	0.889271
C	2.802581	0.691865	0.595329
C	2.707242	1.786932	-0.485303
C	2.381617	3.147988	0.143312
C	2.190754	4.221668	-0.933920
C	1.854467	5.578316	-0.304373
C	1.659961	6.651111	-1.381765
C	1.323853	8.002991	-0.759104
H	5.325371	-1.641583	-2.102836
H	3.987869	-2.610129	-1.544582
H	5.368525	-4.035863	-0.198293
H	6.297285	-3.738921	-1.695141
H	6.738243	-2.873827	-0.181377
H	4.976524	-1.469375	0.921885
H	5.380369	0.566881	-1.346541

H	5.385776	0.965224	0.406706
H	7.289572	-0.652321	0.742243
H	7.382795	-0.817844	-1.059343
H	7.700130	1.652272	-1.274001
H	7.647639	1.812290	0.524044
H	9.638067	0.297811	0.718049
H	9.694284	0.142183	-1.080376
H	10.037023	2.619440	-1.284126
H	9.982278	2.774713	0.514520
H	11.982546	1.265137	0.704837
H	12.037257	1.110061	-1.092077
H	13.670169	2.795240	-0.281862
H	12.401893	3.579248	-1.298010
H	12.346969	3.735048	0.506589
H	2.728667	-0.766231	-0.998768
H	2.526275	-1.478562	1.958501
H	0.363726	-2.884260	0.833282
H	0.050229	-0.079523	1.930634
H	-2.013888	-1.085526	3.060575
H	-2.735386	-0.592953	0.191802
H	-4.200234	0.613730	2.560650
H	-4.546923	0.951301	-0.085951
H	-5.415862	-0.602793	0.007925
H	-6.989614	0.389159	1.705289
H	-6.030111	1.917209	1.704004
H	-6.545603	2.234408	-0.725325
H	-7.484996	0.691971	-0.730394
H	-9.046788	1.638609	0.990188
H	-8.105542	3.180014	0.996497
H	-8.612488	3.499745	-1.439655
H	-9.552254	1.957468	-1.446813
H	-11.115302	2.901971	0.273802
H	-10.175724	4.444523	0.280349
H	-10.688568	4.766423	-2.158661
H	-11.626627	3.225015	-2.165697
H	-13.125392	5.192245	-1.950697
H	-13.202371	4.171554	-0.464757
H	-12.260338	5.719598	-0.457593
H	-4.238709	-1.627577	3.407300
H	-5.822216	-1.162043	2.817879
H	-4.868952	-2.267181	1.806010
H	-2.930348	1.931001	1.317241
H	-1.541125	1.381261	2.222793
H	-1.273466	2.705593	-0.025412
H	-0.235712	1.288834	0.175444
H	-1.712329	1.184529	-0.874863
H	-1.275400	-3.496475	2.163011
H	-2.888960	-3.138177	2.610364
H	-3.450171	-2.701327	0.107413
H	-1.780988	-3.231009	-0.255843
H	-2.211476	-5.417248	0.862987
H	-3.908213	-4.893746	1.196882
H	-4.309386	-4.625497	-1.264898
H	-2.611583	-5.142658	-1.599617
H	-3.074571	-7.361133	-0.512544
H	-4.771769	-6.844805	-0.181815
H	-4.639274	-8.261656	-2.216606
H	-5.177669	-6.592392	-2.640451
H	-3.473184	-7.110452	-2.972269
H	-0.379814	-2.102060	4.007305
H	1.129469	-2.483147	3.260408
H	1.843998	-0.006474	3.599980
H	0.265616	0.252761	4.421732
H	0.859298	-1.496283	6.116971
H	2.446463	-1.758557	5.281898
H	2.082827	-0.171300	6.059016
H	-0.896346	-1.244676	-0.638040
H	0.600094	-0.399834	-0.920415
H	1.615481	-2.479085	-1.886129
H	0.162897	-3.418425	-1.441561
H	-1.235873	-1.966619	-2.935022
H	0.234941	-1.014261	-3.376062
H	1.297241	-3.092523	-4.308033
H	-0.169418	-4.044372	-3.860521
H	-0.202533	-3.409946	-6.260633
H	-1.562552	-2.619970	-5.376251
H	-0.091055	-1.662291	-5.825388
H	2.688337	-3.690702	-0.118083
H	3.832216	-3.316040	1.255059

H	2.213818	-3.882955	1.590112
H	1.830259	0.702970	1.103644
H	3.510851	0.965749	1.403396
H	3.650075	1.880565	-1.051769
H	1.908117	1.520732	-1.209437
H	1.450661	3.067957	0.746445
H	3.213147	3.445702	0.819565
H	3.124470	4.313333	-1.531671
H	1.363581	3.917508	-1.612988
H	0.921134	5.484877	0.293973
H	2.681045	5.883969	0.374581
H	2.590483	6.753053	-1.981840
H	0.832669	6.353353	-2.062442
H	1.188856	8.758537	-1.561529
H	0.382310	7.934727	-0.173496
H	2.147562	8.336272	-0.092388

Hexacontane

0 1

C	-0.433783	0.632114	-0.000000
C	0.434408	1.895928	0.000000
C	-0.433234	3.160104	0.000000
C	0.434842	4.423987	0.000000
C	-0.432936	5.688073	0.000000
C	0.435004	6.952046	0.000000
C	-0.432899	8.216051	0.000000
C	0.434941	9.480096	0.000000
C	-0.433028	10.744048	0.000000
C	0.434735	12.008145	0.000000
C	-0.433367	13.272003	0.000000
C	0.434237	14.536195	0.000000
C	-0.434013	15.799957	0.000000
C	0.433501	17.064204	0.000000
C	-0.434736	18.327980	0.000000
C	0.432899	19.592149	0.000000
C	-0.435147	20.856048	0.000000
C	0.432722	22.120057	0.000000
C	-0.435048	23.384143	0.000000
C	0.433140	24.647930	0.000000
C	-0.434273	25.912263	0.000000
C	0.434291	27.175787	0.000000
C	-0.432720	28.440398	0.000000
C	0.436313	29.703605	0.000000
C	-0.430178	30.968565	0.000000
C	0.439384	32.231433	0.000000
C	-0.426621	33.496745	0.000000
C	0.443239	34.759350	0.000000
C	-0.422977	36.024089	0.000000
C	0.439385	37.282956	0.000000
C	0.433783	-0.632114	-0.000000
C	-0.434408	-1.895928	0.000000
C	0.433234	-3.160104	0.000000
C	-0.434842	-4.423987	0.000000
C	0.432936	-5.688073	0.000000
C	-0.435004	-6.952046	0.000000
C	0.432899	-8.216051	0.000000
C	-0.434941	-9.480096	0.000000
C	0.433028	-10.744048	0.000000
C	-0.434735	-12.008145	0.000000
C	0.433367	-13.272003	0.000000
C	-0.434237	-14.536195	0.000000
C	0.434013	-15.799957	0.000000
C	-0.433501	-17.064204	0.000000
C	0.434736	-18.327980	0.000000
C	-0.432899	-19.592149	0.000000
C	0.435147	-20.856048	0.000000
C	-0.432722	-22.120057	0.000000
C	0.435048	-23.384143	0.000000
C	-0.433140	-24.647930	0.000000
C	0.434273	-25.912263	0.000000
C	-0.434291	-27.175787	0.000000
C	0.432720	-28.440398	0.000000
C	-0.436313	-29.703605	0.000000
C	0.430178	-30.968565	0.000000
C	-0.439384	-32.231433	0.000000
C	0.426621	-33.496745	0.000000
C	-0.443239	-34.759350	0.000000
C	0.422977	-36.024089	0.000000

C	-0.439385	-37.282956	0.000000
H	-1.083506	0.632273	0.903025
H	-1.083506	0.632273	-0.903025
H	1.084133	1.895796	0.903025
H	1.084133	1.895796	-0.903025
H	-1.082962	3.160214	-0.903023
H	-1.082962	3.160214	0.903023
H	1.084562	4.423911	0.903028
H	1.084562	4.423911	-0.903028
H	-1.082663	5.688114	-0.903023
H	-1.082663	5.688114	0.903023
H	1.084725	6.952034	0.903027
H	1.084725	6.952034	-0.903027
H	-1.082629	8.216031	-0.903023
H	-1.082629	8.216031	0.903023
H	1.084657	9.480132	0.903028
H	1.084657	9.480132	-0.903028
H	-1.082759	10.744001	-0.903020
H	-1.082759	10.744001	0.903020
H	1.084457	12.008233	0.903027
H	1.084457	12.008233	-0.903027
H	-1.083089	13.271882	-0.903028
H	-1.083089	13.271882	0.903028
H	1.083962	14.536360	0.903024
H	1.083962	14.536360	-0.903024
H	-1.083737	15.799772	-0.903022
H	-1.083737	15.799772	0.903022
H	1.083223	17.064382	0.903028
H	1.083223	17.064382	-0.903028
H	-1.084456	18.327833	-0.903028
H	-1.084456	18.327833	0.903028
H	1.082630	19.592248	0.903020
H	1.082630	19.592248	-0.903020
H	-1.084859	20.856008	-0.903032
H	-1.084859	20.856008	0.903032
H	1.082459	22.120031	0.903018
H	1.082459	22.120031	-0.903018
H	-1.084755	23.384258	-0.903034
H	-1.084755	23.384258	0.903034
H	1.082882	24.647720	0.903013
H	1.082882	24.647720	-0.903013
H	-1.083976	25.912575	-0.903035
H	-1.083976	25.912575	0.903035
H	1.084033	27.175375	0.903015
H	1.084033	27.175375	-0.903015
H	-1.082428	28.440924	-0.903035
H	-1.082428	28.440924	0.903035
H	1.086050	29.702947	0.903020
H	1.086050	29.702947	-0.903020
H	-1.079887	30.969346	-0.903036
H	-1.079887	30.969346	0.903036
H	1.089121	32.230510	0.903018
H	1.089121	32.230510	-0.903018
H	-1.076339	33.497781	-0.903037
H	-1.076339	33.497781	0.903037
H	1.092940	34.758627	0.903080
H	1.092940	34.758627	-0.903080
H	-1.072801	36.033635	-0.902217
H	-1.072801	36.033635	0.902217
H	1.081529	37.310858	0.906068
H	1.081529	37.310858	-0.906068
H	-0.213746	38.180953	0.000000
H	1.083506	-0.632273	-0.903025
H	1.083506	-0.632273	0.903025
H	-1.084133	-1.895796	0.903025
H	-1.084133	-1.895796	-0.903025
H	1.082962	-3.160214	-0.903023
H	1.082962	-3.160214	0.903023
H	-1.084562	-4.423911	0.903028
H	-1.084562	-4.423911	-0.903028
H	1.082663	-5.688114	-0.903023
H	1.082663	-5.688114	0.903023
H	-1.084725	-6.952034	0.903027
H	-1.084725	-6.952034	-0.903027
H	1.082629	-8.216031	-0.903023
H	1.082629	-8.216031	0.903023
H	-1.084657	-9.480132	0.903028
H	-1.084657	-9.480132	-0.903028
H	1.082759	-10.744001	-0.903020

H	1.082759	-10.744001	0.903020
H	-1.084457	-12.008233	0.903027
H	-1.084457	-12.008233	-0.903027
H	1.083089	-13.271882	-0.903028
H	1.083089	-13.271882	0.903028
H	-1.083962	-14.536360	0.903024
H	-1.083962	-14.536360	-0.903024
H	1.083737	-15.799772	-0.903022
H	1.083737	-15.799772	0.903022
H	-1.083223	-17.064382	0.903028
H	-1.083223	-17.064382	-0.903028
H	1.084456	-18.327833	-0.903028
H	1.084456	-18.327833	0.903028
H	-1.082630	-19.592248	0.903020
H	-1.082630	-19.592248	-0.903020
H	1.084859	-20.856008	-0.903032
H	1.084859	-20.856008	0.903032
H	-1.082459	-22.120031	0.903018
H	-1.082459	-22.120031	-0.903018
H	1.084755	-23.384258	-0.903034
H	1.084755	-23.384258	0.903034
H	-1.082882	-24.647720	0.903013
H	-1.082882	-24.647720	-0.903013
H	1.083976	-25.912575	-0.903035
H	1.083976	-25.912575	0.903035
H	-1.084033	-27.175375	0.903015
H	-1.084033	-27.175375	-0.903015
H	1.082428	-28.440924	-0.903035
H	1.082428	-28.440924	0.903035
H	-1.086050	-29.702947	0.903020
H	-1.086050	-29.702947	-0.903020
H	1.079887	-30.969346	-0.903036
H	1.079887	-30.969346	0.903036
H	-1.089121	-32.230510	0.903018
H	-1.089121	-32.230510	-0.903018
H	1.076339	-33.497781	-0.903037
H	1.076339	-33.497781	0.903037
H	-1.092940	-34.758627	0.903080
H	-1.092940	-34.758627	-0.903080
H	1.072801	-36.033635	-0.902217
H	1.072801	-36.033635	0.902217
H	-1.081529	-37.310858	0.906068
H	-1.081529	-37.310858	-0.906068
H	0.213746	-38.180953	0.000000

13-Methyl-11,13,15,17-tetraoctylheptacosane

0 1

C	0.853046	0.003166	-0.080193
C	0.023507	-0.621725	1.068717
C	1.616665	1.247820	0.488568
C	2.613691	1.928198	-0.475592
C	2.779665	3.417948	-0.129957
C	3.483204	4.194251	-1.247911
C	3.578332	5.683827	-0.888981
C	4.073093	6.516265	-2.076047
C	4.187989	7.996667	-1.692764
C	4.571027	8.851400	-2.896690
C	1.846650	-1.073590	-0.648143
C	2.848209	-1.722654	0.365744
C	2.377968	-3.134585	0.822489
C	2.566894	-4.250307	-0.224206
C	1.892456	-5.549420	0.233017
C	2.101972	-6.670326	-0.792031
C	1.423909	-7.967446	-0.335510
C	1.632984	-9.088360	-1.360424
C	0.954449	-10.384771	-0.903534
C	1.160255	-11.503009	-1.921141
C	4.292336	-1.718245	-0.201471
C	5.343884	-2.170687	0.825057
C	6.761232	-2.029609	0.257299
C	7.810998	-2.490311	1.275388
C	9.228013	-2.348546	0.707369
C	10.277971	-2.809239	1.725287
C	11.695036	-2.667642	1.157122
C	12.745142	-3.127336	2.175300
C	14.161575	-2.985811	1.606450
C	15.209738	-3.442253	2.617086
C	-0.133830	0.385018	-1.248859

C	-1.132616	1.570522	-0.995794
C	-0.690010	2.835800	-1.782243
C	-1.088479	4.146754	-1.087439
C	-0.540827	5.351857	-1.866592
C	-0.654892	6.648436	-1.058969
C	-0.085942	7.831650	-1.852610
C	-0.059974	9.107852	-1.004930
C	0.493297	10.289463	-1.810147
C	0.555481	11.554782	-0.960104
C	-2.582541	1.193129	-1.412840
C	-3.316042	0.241030	-0.426565
C	-3.714449	0.933613	0.910883
C	-4.797286	2.021385	0.787150
C	-5.123581	2.622968	2.159632
C	-6.195883	3.712812	2.043211
C	-6.519819	4.315202	3.415364
C	-7.591724	5.405206	3.299001
C	-7.915157	6.007104	4.671210
C	-8.982147	7.092205	4.559644
C	-4.511936	-0.433709	-1.146647
C	-5.237042	-1.476055	-0.279661
C	-6.267955	-2.256906	-1.103675
C	-7.006546	-3.280881	-0.233649
C	-8.035953	-4.063226	-1.057606
C	-8.774354	-5.087060	-0.187312
C	-9.803738	-5.869735	-1.011143
C	-10.542259	-6.893422	-0.140851
C	-11.570962	-7.675766	-0.965229
C	-12.308637	-8.695388	-0.102340
H	-0.725911	0.092365	1.462686
H	-0.513364	-1.529507	0.719688
H	0.665939	-0.904202	1.927705
H	0.876543	1.987925	0.850739
H	2.176853	0.973512	1.406459
H	3.603142	1.433500	-0.391677
H	2.293101	1.815856	-1.528427
H	1.781693	3.879427	0.030008
H	3.357923	3.511348	0.815467
H	4.503807	3.782060	-1.405932
H	2.903712	4.078205	-2.190388
H	2.575691	6.058823	-0.585271
H	4.273325	5.807970	-0.029432
H	5.067521	6.141575	-2.404521
H	3.356167	6.408191	-2.920034
H	3.217684	8.360323	-1.290750
H	4.960362	8.118158	-0.902354
H	5.548080	8.521165	-3.309313
H	3.793109	8.775013	-3.686158
H	4.656188	9.913943	-2.585652
H	1.247268	-1.873282	-1.132492
H	2.409277	-0.616327	-1.490552
H	2.884023	-1.101239	1.281510
H	2.923650	-3.418249	1.748348
H	1.306195	-3.086457	1.104931
H	2.136496	-3.944292	-1.200399
H	3.650274	-4.447814	-0.368119
H	2.320095	-5.860762	1.211714
H	0.802132	-5.371414	0.363834
H	1.676251	-6.357586	-1.771093
H	3.191945	-6.851103	-0.921932
H	1.849684	-8.280151	0.643545
H	0.333935	-7.786643	-0.205520
H	1.207302	-8.775804	-2.339591
H	2.722902	-9.269696	-1.490273
H	1.377675	-10.705486	0.073392
H	-0.136436	-10.212033	-0.775092
H	0.658840	-12.427229	-1.564259
H	0.723024	-11.217746	-2.901723
H	2.243453	-11.713508	-2.049422
H	4.352594	-2.349054	-1.114100
H	4.548269	-0.683152	-0.511376
H	5.253545	-1.549859	1.743428
H	5.174113	-3.233230	1.098808
H	6.849492	-2.643916	-0.665998
H	6.948304	-0.965243	-0.006748
H	7.721658	-1.876580	2.198934
H	7.625386	-3.555042	1.539048
H	9.317058	-2.962236	-0.216244
H	9.413726	-1.283839	0.443716

H 10.188933 -2.195434 2.648818
H 10.092187 -3.873898 1.989088
H 11.784224 -3.282007 0.233964
H 11.880601 -1.603109 0.892640
H 12.656456 -2.512755 3.098388
H 12.559815 -4.191878 2.440003
H 14.258936 -3.600719 0.685162
H 14.355434 -1.923154 1.342734
H 15.150950 -2.825578 3.539225
H 15.053690 -4.510448 2.879187
H 16.223396 -3.328834 2.178219
H 0.445845 0.581193 -2.176896
H -0.707769 -0.531445 -1.509342
H -1.143537 1.826849 0.075714
H 0.407149 2.851674 -1.896107
H -1.105832 2.815449 -2.814312
H -2.194754 4.222847 -1.016640
H -0.671142 4.151762 -0.056801
H 0.531490 5.183294 -2.109720
H -1.101712 5.454076 -2.821588
H -1.722367 6.843097 -0.814982
H -0.087623 6.535868 -0.108510
H 0.949759 7.594173 -2.181315
H -0.711068 7.998913 -2.757354
H -1.091623 9.345134 -0.663125
H 0.580499 8.940372 -0.110731
H 1.516449 10.052067 -2.174559
H -0.157602 10.479182 -2.691358
H 0.954924 12.392607 -1.569563
H -0.459400 11.828707 -0.600904
H 1.224973 11.400212 -0.087164
H -3.193741 2.106419 -1.556506
H -2.530746 0.721534 -2.419634
H -2.614051 -0.577171 -0.163479
H -4.062327 0.157801 1.625695
H -2.820106 1.379537 1.388279
H -4.445556 2.836301 0.121731
H -5.726267 1.587386 0.362087
H -5.488498 1.819009 2.836483
H -4.200154 3.062281 2.597867
H -5.831474 4.516470 1.365705
H -7.120217 3.274178 1.606409
H -6.884313 3.511721 4.092985
H -5.595336 4.753704 3.852036
H -7.227304 6.208903 2.621543
H -8.516379 4.966901 2.862466
H -8.283389 5.209790 5.353166
H -6.995953 6.450928 5.112174
H -8.628577 7.914976 3.902284
H -9.921493 6.668556 4.144625
H -9.195188 7.508937 5.566579
H -5.235032 0.332836 -1.498945
H -4.123761 -0.952454 -2.051410
H -4.493959 -2.185676 0.146205
H -5.764561 -0.966758 0.554786
H -7.003621 -1.545769 -1.540426
H -5.751368 -2.786334 -1.934544
H -6.270076 -3.990299 0.204467
H -7.525275 -2.752323 0.596516
H -8.772303 -3.353658 -1.495673
H -7.517429 -4.592010 -1.887734
H -8.037906 -5.796438 0.250901
H -9.292927 -4.558234 0.642768
H -10.540130 -5.160359 -1.449472
H -9.285121 -6.398698 -1.841110
H -9.806092 -7.602947 0.297668
H -11.061357 -6.364625 0.688951
H -12.312455 -6.973075 -1.404289
H -11.058543 -8.210535 -1.794574
H -11.591944 -9.426360 0.329072
H -12.851321 -8.183640 0.720882
H -13.045262 -9.245580 -0.724949

12-(2-ethylhexyl)-11-heptyl-10-hexyl-12-nonyl-11-octyldocosane

0 1
C -2.930599 2.383416 3.922842
C -3.279775 3.664213 3.154392
C -2.821245 1.131783 2.998585

C	-1.427330	1.174857	2.249763
C	-0.934761	0.307307	0.980371
C	-1.628006	0.820959	-0.468660
C	-1.033292	2.254906	-1.013343
C	0.429539	2.313125	-1.598684
C	1.141213	3.632295	-1.238940
C	2.562124	3.672915	-1.815001
C	3.266738	4.984433	-1.449679
C	4.689159	5.022507	-2.020378
C	5.394302	6.333650	-1.653716
C	6.816704	6.372463	-2.224583
C	7.520938	7.683582	-1.857282
C	8.937027	7.726387	-2.424019
C	-1.927883	3.032146	-2.063077
C	-2.786079	4.156516	-1.446773
C	-3.645208	4.844288	-2.515021
C	-4.494741	5.963805	-1.901821
C	-5.353785	6.650022	-2.969778
C	-6.202514	7.763257	-2.362617
C	-1.618335	-0.268596	-1.627235
C	-0.372115	-0.528074	-2.502271
C	-0.490002	-1.886474	-3.220295
C	0.849704	-2.341827	-3.809136
C	0.713375	-3.729027	-4.450956
C	2.075266	-4.276075	-4.890998
C	1.928546	-5.669495	-5.513454
C	3.285329	-6.242307	-5.911199
C	-3.185334	1.082952	-0.240334
C	-4.143811	-0.086193	0.111469
C	-5.599175	0.401685	0.193296
C	-6.526495	-0.713331	0.694334
C	-7.967580	-0.211589	0.835065
C	-8.884395	-1.319974	1.364729
C	-10.319649	-0.824051	1.513096
C	0.632720	0.550415	0.974675
C	1.460467	0.177950	2.238921
C	2.926527	-0.105131	1.880000
C	3.756648	-0.406824	3.133079
C	5.217414	-0.694984	2.767712
C	6.048906	-0.997546	4.019732
C	7.509802	-1.285386	3.653940
C	8.341623	-1.588155	4.905759
C	9.802031	-1.875840	4.539259
C	10.633360	-2.177438	5.782753
C	-1.141659	-1.243121	1.279979
C	-0.163301	-2.262873	0.635195
C	-0.786298	-3.654309	0.461496
C	0.221975	-4.615350	-0.187081
C	-0.448167	-5.916768	-0.638475
C	0.570977	-6.849658	-1.305044
C	-0.106389	-8.112812	-1.847334
C	0.916810	-9.042270	-2.510262
C	0.244992	-10.293652	-3.067456
C	-3.035160	-0.171708	3.819460
C	-4.428996	-0.271258	4.476678
C	-4.791072	-1.724551	4.811225
C	-6.171323	-1.814343	5.455809
H	-1.983792	2.535118	4.485774
H	-3.731157	2.259237	4.676655
H	-4.216821	3.524970	2.574170
H	-3.437982	4.493580	3.875706
H	-2.467639	3.968244	2.464732
H	-3.679602	1.205923	2.331401
H	-1.219923	2.226338	1.985023
H	-0.737223	0.982328	3.090188
H	-1.015193	2.894729	-0.108334
H	1.085603	1.494659	-1.309327
H	0.417072	2.235923	-2.706157
H	0.560218	4.491708	-1.638080
H	1.196915	3.738553	-0.133939
H	3.147197	2.817350	-1.410509
H	2.513294	3.576743	-2.922122
H	2.683936	5.840517	-1.855928
H	3.312301	5.081271	-0.342337
H	5.271359	4.165818	-1.614442
H	4.643818	4.926218	-3.127745
H	4.811825	7.190362	-2.059160
H	5.439772	6.429544	-0.546300
H	7.399470	5.515880	-1.819226

H	6.771498	6.277004	-3.332060
H	6.945612	8.544600	-2.262272
H	7.573336	7.784074	-0.751153
H	8.911559	7.653310	-3.532216
H	9.421193	8.685201	-2.142532
H	9.541993	6.889416	-2.014660
H	-1.304453	3.554616	-2.821661
H	-2.525641	2.342477	-2.691056
H	-3.455142	3.773781	-0.656090
H	-2.111169	4.908659	-0.981795
H	-2.985079	5.272747	-3.301222
H	-4.314791	4.091281	-2.986470
H	-5.156941	5.535677	-1.116943
H	-3.825699	6.715995	-1.428295
H	-4.698424	7.085372	-3.755473
H	-6.026873	5.904013	-3.446008
H	-6.812396	8.241458	-3.157804
H	-6.886303	7.349901	-1.590811
H	-5.551968	8.536253	-1.900709
H	-1.950253	-1.231531	-1.202716
H	-2.413664	-0.023355	-2.365716
H	-0.300109	0.250406	-3.287616
H	0.557669	-0.516863	-1.916087
H	-0.833475	-2.666173	-2.506818
H	-1.248783	-1.808522	-4.029861
H	1.191632	-1.608174	-4.571637
H	1.607136	-2.388427	-2.995456
H	0.262806	-4.434092	-3.717691
H	0.037278	-3.660078	-5.331498
H	2.528942	-3.583905	-5.634214
H	2.747940	-4.338809	-4.006985
H	1.449086	-6.358522	-4.784568
H	1.283171	-5.609748	-6.416831
H	3.778658	-5.583494	-6.657248
H	3.938954	-6.340676	-5.018320
H	3.146421	-7.247866	-6.361246
H	-3.310678	1.926946	0.453237
H	-3.646420	1.457520	-1.168163
H	-4.092857	-0.884677	-0.656945
H	-3.912600	-0.527667	1.086756
H	-5.661793	1.266516	0.890139
H	-5.935121	0.737596	-0.812432
H	-6.498180	-1.564181	-0.021560
H	-6.169505	-1.073840	1.684561
H	-7.990096	0.649785	1.538881
H	-8.336384	0.131351	-0.156828
H	-8.872439	-2.183240	0.664062
H	-8.519822	-1.666252	2.356691
H	-10.362792	0.023375	2.230115
H	-10.717409	-0.493776	0.529851
H	-10.959270	-1.646592	1.896832
H	1.081713	0.002195	0.134783
H	0.828492	1.629921	0.846500
H	1.449845	1.032456	2.951302
H	1.047449	-0.699549	2.771142
H	2.974934	-0.978120	1.192167
H	3.353137	0.780098	1.357989
H	3.714867	0.465973	3.821502
H	3.326632	-1.290241	3.654872
H	5.258960	-1.567665	2.078970
H	5.646897	0.188390	2.245370
H	6.007232	-0.125036	4.708629
H	5.619433	-1.881103	4.541734
H	7.551403	-2.157804	2.964894
H	7.939193	-0.401783	3.131926
H	8.300383	-0.715759	5.594877
H	7.912552	-2.471884	5.427852
H	9.851473	-2.749550	3.853291
H	10.238886	-0.995094	4.019830
H	10.621627	-1.308573	6.474865
H	10.232333	-3.070512	6.307810
H	11.683995	-2.381511	5.486970
H	-1.030691	-1.418591	2.367396
H	-2.154566	-1.565429	1.024752
H	0.164268	-1.932474	-0.353235
H	0.748617	-2.365860	1.259337
H	-1.098682	-4.053230	1.451255
H	-1.686563	-3.569554	-0.186250
H	0.684620	-4.129419	-1.074354

H 1.029543 -4.843808 0.542831
H -0.899532 -6.426313 0.241081
H -1.256261 -5.677826 -1.364941
H 1.068886 -6.315452 -2.144208
H 1.346948 -7.135628 -0.561092
H -0.606394 -8.650537 -1.011670
H -0.877909 -7.823434 -2.594849
H 1.426785 -8.508678 -3.341709
H 1.683957 -9.347003 -1.765365
H 1.009184 -10.947389 -3.538189
H -0.253342 -10.859806 -2.251868
H -0.507988 -10.017386 -3.836095
H -2.967120 -1.032940 3.139096
H -2.245314 -0.276168 4.594941
H -4.441426 0.314056 5.420501
H -5.201547 0.140401 3.790268
H -4.794925 -2.335800 3.882159
H -4.035411 -2.146702 5.509003
H -6.405250 -2.874863 5.686959
H -6.195145 -1.230960 6.400807
H -6.946416 -1.422989 4.762410

11-Decyl-10-heptyl-12,12-dimethyl-11-nonyl-10-octyltetracosane

0 1
C 0.639406 -0.670046 1.067887
C 1.381263 0.582727 1.623047
C 0.572974 1.303546 2.716431
C 1.311599 2.549220 3.220419
C 0.500803 3.268730 4.304414
C 1.236980 4.517044 4.804685
C 0.424918 5.238540 5.886720
C 1.161009 6.486980 6.386987
C 0.348374 7.208179 7.468359
C 1.078186 8.451013 7.969468
C 0.575702 -0.977315 -0.504734
C 2.084670 -1.444010 -1.114564
C 3.258197 -0.549031 -0.542824
C 4.672789 -0.767839 -1.144639
C 5.732486 -0.003654 -0.339726
C 7.127436 -0.184709 -0.950057
C 8.184449 0.577345 -0.142413
C 9.579036 0.398281 -0.753795
C 10.636333 1.160082 0.053926
C 12.030144 0.981963 -0.558780
C 13.085483 1.738327 0.242794
C 2.483176 -2.960491 -0.772793
C 3.108252 -3.314903 0.605005
C 3.271603 -4.834643 0.757962
C 3.912751 -5.190287 2.105087
C 4.072456 -6.707634 2.255358
C 4.713149 -7.062972 3.602140
C 4.872121 -8.580203 3.752225
C 5.509734 -8.937865 5.091582
C 2.185357 -1.341183 -2.722366
C 2.323836 0.033840 -3.442919
C 3.043829 -0.106744 -4.794440
C 3.121069 1.239918 -5.524721
C 3.845756 1.096998 -6.868085
C 3.922825 2.443276 -7.597005
C 4.643496 2.305336 -8.934874
C -0.658905 -2.091060 -0.711297
C -2.101791 -1.443932 -0.549600
C -3.334080 -2.372437 -0.702463
C -4.620406 -1.642353 -0.287761
C -5.853028 -2.531892 -0.489283
C -7.133837 -1.798011 -0.074903
C -8.367860 -2.684163 -0.281033
C -9.648460 -1.948995 0.131832
C -10.883142 -2.833767 -0.076868
C -12.163767 -2.098153 0.335251
C -13.398684 -2.982198 0.125034
C -14.678604 -2.245970 0.536944
C -15.910180 -3.122422 0.328565
C -0.681430 -2.750026 -2.123788
C -0.607155 -3.235090 0.359609
C 0.161976 0.373417 -1.243613
C -0.775851 1.420370 -0.568287
C -1.595785 2.198062 -1.609654

C	-2.507388	3.230644	-0.935359
C	-3.329245	4.001706	-1.974528
C	-4.244465	5.029754	-1.299156
C	-5.068558	5.799659	-2.337732
C	-5.983682	6.826974	-1.661697
C	-6.805893	7.595330	-2.692205
H	1.030179	-1.531257	1.618244
H	-0.389804	-0.616196	1.457391
H	2.328994	0.265678	2.106177
H	1.641434	1.306863	0.833194
H	-0.418333	1.607696	2.318243
H	0.405871	0.604281	3.565418
H	2.298788	2.249259	3.636249
H	1.482667	3.244049	2.368554
H	-0.486820	3.566721	3.887829
H	0.330268	2.575183	5.157322
H	2.223982	4.219130	5.222616
H	1.408881	5.209422	3.951049
H	-0.562026	5.536394	5.468560
H	0.252832	4.546244	6.740370
H	2.147789	6.189304	6.805693
H	1.333324	7.179386	5.533437
H	-0.638167	7.512672	7.055578
H	0.174728	6.522902	8.326614
H	1.242272	9.165157	7.134509
H	0.467075	8.951574	8.749820
H	2.058310	8.171166	8.411204
H	3.034905	0.525291	-0.653008
H	3.356964	-0.735575	0.536655
H	4.946767	-1.840714	-1.155876
H	4.713731	-0.406243	-2.190284
H	5.474299	1.078373	-0.326542
H	5.739828	-0.378194	0.707782
H	7.384927	-1.266880	-0.964367
H	7.122695	0.191651	-1.996952
H	7.925855	1.659239	-0.126747
H	8.190265	0.199972	0.904089
H	9.837508	-0.683644	-0.769772
H	9.573076	0.775871	-1.800230
H	10.377615	2.241954	0.070842
H	10.643339	0.781705	1.100084
H	12.297161	-0.097280	-0.576103
H	12.031832	1.362015	-1.603732
H	12.855495	2.825091	0.254769
H	13.122411	1.359332	1.286391
H	14.082396	1.591560	-0.223680
H	1.670289	-3.660422	-0.976312
H	3.248372	-3.317854	-1.492282
H	4.115013	-2.860397	0.702953
H	2.503756	-2.956933	1.447933
H	2.274032	-5.321873	0.688420
H	3.911128	-5.221533	-0.065953
H	4.911954	-4.706450	2.176229
H	3.273564	-4.804555	2.929864
H	3.073184	-7.191411	2.184384
H	4.711623	-7.093641	1.430702
H	5.712660	-6.579639	3.673089
H	4.074198	-6.676789	4.426925
H	3.876098	-9.070033	3.686137
H	5.512890	-8.973080	2.932822
H	6.518989	-8.480816	5.172820
H	4.875214	-8.578007	5.929421
H	5.611906	-10.040620	5.171921
H	3.074301	-1.920381	-3.048139
H	1.372974	-1.860395	-3.235632
H	1.321268	0.447001	-3.667785
H	2.876396	0.777856	-2.839690
H	4.075371	-0.487781	-4.627574
H	2.495283	-0.838747	-5.427671
H	2.091697	1.622955	-5.701337
H	3.666500	1.971982	-4.888930
H	4.875589	0.714785	-6.691891
H	3.300678	0.364889	-7.504115
H	2.896525	2.830125	-7.779824
H	4.469883	3.179375	-6.968308
H	5.682051	1.943443	-8.778259
H	4.101888	1.593157	-9.593326
H	4.684248	3.293976	-9.438760
H	-2.203491	-0.997659	0.455289

H	-2.247616	-0.653538	-1.307848
H	-3.452406	-2.678108	-1.763027
H	-3.229671	-3.280278	-0.073880
H	-4.550845	-1.352202	0.783951
H	-4.731212	-0.717574	-0.896582
H	-5.926261	-2.822267	-1.560651
H	-5.743875	-3.455583	0.120951
H	-7.062394	-1.510358	0.997361
H	-7.240275	-0.872383	-0.682818
H	-8.438642	-2.972360	-1.353197
H	-8.262201	-3.609351	0.327611
H	-9.578372	-1.662280	1.204439
H	-9.752901	-1.022831	-0.475565
H	-10.952824	-3.120737	-1.149434
H	-10.779048	-3.759741	0.530854
H	-12.094543	-1.812076	1.408092
H	-12.267245	-1.171605	-0.271731
H	-13.468115	-3.268321	-0.947802
H	-13.295727	-3.908691	0.732211
H	-14.618178	-1.960254	1.609826
H	-14.789981	-1.319949	-0.068399
H	-16.008411	-3.401736	-0.742146
H	-15.835979	-4.044746	0.943292
H	-16.819842	-2.564033	0.634645
H	0.193978	-3.365248	-2.362448
H	-1.496681	-3.492873	-2.216873
H	-0.846754	-1.994986	-2.919438
H	-1.131653	-2.938042	1.293372
H	-1.091630	-4.166780	-0.002598
H	0.397725	-3.514729	0.689658
H	1.061781	0.968300	-1.456630
H	-0.271008	0.099620	-2.224463
H	-1.458604	0.985966	0.176526
H	-0.168648	2.173042	-0.025763
H	-0.900911	2.716853	-2.306608
H	-2.221042	1.495004	-2.200879
H	-3.197258	2.709268	-0.235290
H	-1.886105	3.945974	-0.352341
H	-2.640145	4.525065	-2.673656
H	-3.948820	3.285504	-2.558361
H	-4.932273	4.505928	-0.599013
H	-3.625033	5.746649	-0.716072
H	-4.381066	6.323930	-3.037837
H	-5.688071	5.082790	-2.920815
H	-6.676462	6.309522	-0.962645
H	-5.370870	7.549555	-1.079712
H	-7.457727	8.330245	-2.174533
H	-6.136501	8.143335	-3.389124
H	-7.447562	6.897843	-3.271742

Heptacontane

0 1

C	-0.632323	-0.433492	0.000000
C	-1.895772	0.435262	0.000000
C	-3.160349	-0.431824	-0.000000
C	-4.423894	0.436769	-0.000000
C	-5.688357	-0.430490	-0.000000
C	-6.952004	0.437949	-0.000000
C	-8.216388	-0.429437	-0.000000
C	-9.480095	0.438913	0.000000
C	-10.744453	-0.428515	0.000000
C	-12.008145	0.439857	0.000000
C	-13.272567	-0.427473	0.000000
C	-14.536146	0.441061	-0.000000
C	-15.800694	-0.426069	-0.000000
C	-17.064115	0.442695	-0.000000
C	-18.328819	-0.424194	-0.000000
C	-19.592078	0.444812	-0.000001
C	-20.856909	-0.421872	-0.000001
C	-22.120079	0.447262	-0.000000
C	-23.384924	-0.419395	0.000000
C	-24.648129	0.449684	0.000001
C	-25.912880	-0.417107	0.000001
C	-27.176222	0.451760	0.000001
C	-28.440769	-0.415320	0.000001
C	-29.704336	0.453201	0.000001
C	-30.968607	-0.414281	0.000000

C	-32.232467	0.453805	-0.000000
C	-33.496420	-0.414138	-0.000000
C	-34.760597	0.453476	-0.000000
C	-36.024211	-0.414961	-0.000000
C	-37.288723	0.452158	-0.000000
C	-38.552044	-0.416735	0.000000
C	-39.816841	0.450001	0.000000
C	-41.079967	-0.419085	0.000000
C	-42.344149	0.447935	0.000000
C	-43.603556	-0.413624	-0.000000
C	0.632323	0.433492	0.000001
C	1.895772	-0.435262	0.000001
C	3.160349	0.431824	0.000001
C	4.423894	-0.436769	0.000001
C	5.688357	0.430490	0.000000
C	6.952004	-0.437949	-0.000000
C	8.216388	0.429437	-0.000000
C	9.480095	-0.438913	-0.000000
C	10.744453	0.428515	-0.000000
C	12.008145	-0.439857	0.000000
C	13.272567	0.427473	0.000000
C	14.536146	-0.441061	-0.000000
C	15.800694	0.426069	-0.000000
C	17.064115	-0.442695	-0.000000
C	18.328819	0.424194	0.000000
C	19.592078	-0.444812	0.000000
C	20.856909	0.421872	0.000000
C	22.120079	-0.447262	-0.000000
C	23.384924	0.419395	-0.000000
C	24.648129	-0.449684	-0.000001
C	25.912880	0.417107	-0.000001
C	27.176222	-0.451760	-0.000000
C	28.440769	0.415320	-0.000000
C	29.704336	-0.453201	0.000000
C	30.968607	0.414281	0.000000
C	32.232467	-0.453805	0.000000
C	33.496420	0.414138	0.000000
C	34.760597	-0.453476	-0.000000
C	36.024211	0.414961	-0.000000
C	37.288723	-0.452158	-0.000000
C	38.552044	0.416735	-0.000000
C	39.816841	-0.450001	-0.000000
C	41.079967	0.419085	-0.000000
C	42.344149	-0.447935	-0.000000
C	43.603556	0.413624	-0.000000
H	-0.632772	-1.083220	0.903019
H	-0.632772	-1.083220	-0.903019
H	-1.895350	1.084989	0.903019
H	-1.895350	1.084989	-0.903019
H	-3.160737	-1.081555	-0.903017
H	-3.160737	-1.081555	0.903018
H	-4.423556	1.086494	0.903023
H	-4.423556	1.086494	-0.903023
H	-5.688667	-1.080222	-0.903017
H	-5.688667	-1.080223	0.903017
H	-6.951732	1.087675	0.903022
H	-6.951732	1.087675	-0.903022
H	-8.216641	-1.079167	-0.903020
H	-8.216641	-1.079167	0.903020
H	-9.479856	1.088640	0.903020
H	-9.479856	1.088640	-0.903020
H	-10.744709	-1.078241	-0.903022
H	-10.744709	-1.078241	0.903022
H	-12.007877	1.089593	0.903020
H	-12.007877	1.089593	-0.903020
H	-13.272881	-1.077197	-0.903025
H	-13.272881	-1.077197	0.903025
H	-14.535784	1.090795	0.903018
H	-14.535784	1.090795	-0.903018
H	-15.801118	-1.075790	-0.903026
H	-15.801118	-1.075790	0.903026
H	-17.063637	1.092431	0.903019
H	-17.063637	1.092431	-0.903019
H	-18.329362	-1.073916	-0.903028
H	-18.329362	-1.073916	0.903028
H	-19.591494	1.094542	0.903021
H	-19.591494	1.094542	-0.903020
H	-20.857536	-1.071598	-0.903022
H	-20.857536	-1.071599	0.903022

H	-22.119452	1.096990	0.903025
H	-22.119452	1.096990	-0.903025
H	-23.385540	-1.069124	-0.903025
H	-23.385540	-1.069124	0.903025
H	-24.647552	1.099405	0.903027
H	-24.647552	1.099406	-0.903027
H	-25.913405	-1.066844	-0.903020
H	-25.913405	-1.066843	0.903019
H	-27.175772	1.101471	0.903030
H	-27.175772	1.101472	-0.903031
H	-28.441138	-1.065061	-0.903013
H	-28.441138	-1.065060	0.903012
H	-29.704076	1.102909	0.903034
H	-29.704076	1.102910	-0.903035
H	-30.968765	-1.064026	-0.903017
H	-30.968764	-1.064025	0.903016
H	-32.232431	1.103511	0.903038
H	-32.232431	1.103511	-0.903038
H	-33.496339	-1.063880	-0.903014
H	-33.496339	-1.063880	0.903015
H	-34.760807	1.103183	0.903036
H	-34.760807	1.103183	-0.903036
H	-36.023876	-1.064699	-0.903015
H	-36.023876	-1.064699	0.903015
H	-37.289169	1.101870	0.903033
H	-37.289169	1.101870	-0.903033
H	-38.551483	-1.066474	-0.903016
H	-38.551483	-1.066474	0.903016
H	-39.817486	1.099721	0.903037
H	-39.817486	1.099721	-0.903037
H	-41.079651	-1.068786	-0.903078
H	-41.079651	-1.068786	0.903078
H	-42.353281	1.097768	0.902213
H	-42.353281	1.097768	-0.902214
H	-43.631875	-1.055744	0.906074
H	-44.501123	0.240099	-0.000000
H	-43.631875	-1.055744	-0.906074
H	0.632772	1.083220	-0.903019
H	0.632772	1.083220	0.903018
H	1.895350	-1.084989	0.903019
H	1.895350	-1.084990	-0.903020
H	3.160737	1.081555	-0.903018
H	3.160737	1.081554	0.903017
H	4.423556	-1.086494	0.903022
H	4.423556	-1.086494	-0.903023
H	5.688667	1.080223	-0.903017
H	5.688667	1.080223	0.903017
H	6.951732	-1.087675	0.903022
H	6.951732	-1.087675	-0.903022
H	8.216641	1.079167	-0.903020
H	8.216641	1.079167	0.903020
H	9.479856	-1.088640	0.903020
H	9.479856	-1.088640	-0.903020
H	10.744709	1.078241	-0.903022
H	10.744709	1.078241	0.903022
H	12.007877	-1.089593	0.903020
H	12.007877	-1.089593	-0.903020
H	13.272881	1.077197	-0.903025
H	13.272881	1.077197	0.903025
H	14.535784	-1.090795	0.903018
H	14.535784	-1.090795	-0.903018
H	15.801118	1.075790	-0.903026
H	15.801118	1.075790	0.903026
H	17.063637	-1.092431	0.903019
H	17.063637	-1.092431	-0.903019
H	18.329362	1.073916	-0.903028
H	18.329362	1.073916	0.903028
H	19.591494	-1.094542	0.903020
H	19.591494	-1.094542	-0.903021
H	20.857536	1.071599	-0.903022
H	20.857536	1.071599	0.903022
H	22.119452	-1.096990	0.903025
H	22.119452	-1.096990	-0.903025
H	23.385540	1.069123	-0.903025
H	23.385540	1.069124	0.903025
H	24.647552	-1.099406	0.903027
H	24.647552	-1.099405	-0.903027
H	25.913405	1.066844	-0.903019
H	25.913405	1.066844	0.903019

H 27.175772 -1.101471 0.903031
H 27.175772 -1.101471 -0.903031
H 28.441138 1.065060 -0.903013
H 28.441138 1.065061 0.903013
H 29.704076 -1.102909 0.903034
H 29.704076 -1.102909 -0.903034
H 30.968765 1.064026 -0.903017
H 30.968765 1.064025 0.903016
H 32.232431 -1.103511 0.903037
H 32.232431 -1.103511 -0.903038
H 33.496339 1.063880 -0.903015
H 33.496339 1.063880 0.903014
H 34.760806 -1.103183 0.903036
H 34.760807 -1.103183 -0.903036
H 36.023876 1.064699 -0.903015
H 36.023876 1.064699 0.903015
H 37.289169 -1.101870 0.903033
H 37.289169 -1.101870 -0.903033
H 38.551483 1.066474 -0.903016
H 38.551483 1.066474 0.903016
H 39.817486 -1.099721 0.903037
H 39.817486 -1.099721 -0.903037
H 41.079651 1.068786 -0.903078
H 41.079651 1.068786 0.903078
H 42.353281 -1.097768 0.902213
H 42.353281 -1.097768 -0.902214
H 43.631875 1.055744 -0.906074
H 43.631875 1.055744 0.906074
H 44.501123 -0.240099 -0.000000

16-Decyl-16-pentadecyl-15-tridecyldotriacontane

0 1
C -0.584260 -2.758141 -1.743725
C -1.092930 -3.858109 -0.783316
C -1.368458 -5.161315 -1.544186
C -1.883552 -6.253806 -0.599436
C -2.164630 -7.553731 -1.361995
C -2.683837 -8.644320 -0.417642
C -2.966960 -9.944088 -1.180057
C -3.487760 -11.034040 -0.235851
C -3.770843 -12.333230 -0.998630
C -4.289985 -13.420403 -0.062185
C -0.276742 -1.347791 -1.096747
C -1.622874 -0.738494 -0.476431
C -1.444758 0.620795 0.267272
C -2.634460 0.956071 1.185818
C -2.381423 2.251877 1.966564
C -3.571680 2.589844 2.872095
C -3.318838 3.883892 3.654454
C -4.508089 4.219242 4.562214
C -4.255784 5.512873 5.345587
C -5.444151 5.845953 6.255384
C -5.192355 7.139311 7.039390
C -6.380160 7.470808 7.950541
C -6.128712 8.764046 8.734927
C -7.316182 9.094864 9.646674
C -7.064219 10.387873 10.430599
C -8.243990 10.720409 11.339419
C -2.806987 -0.610753 -1.504374
C -4.059190 -1.392488 -1.072880
C -5.246225 -1.093171 -1.996337
C -6.481935 -1.901061 -1.582899
C -7.668160 -1.604632 -2.507783
C -8.902557 -2.416037 -2.097165
C -10.088112 -2.121746 -3.023797
C -11.321787 -2.935248 -2.615006
C -12.506917 -2.641904 -3.542536
C -13.740173 -3.456703 -3.134948
C -14.925144 -3.163640 -4.062697
C -16.157518 -3.979127 -3.655112
C -17.339158 -3.690201 -4.576318
C 0.802480 -1.547863 0.037072
C 2.064870 -2.339792 -0.377570
C 3.175169 -2.202237 0.672049
C 4.411425 -3.018804 0.277200
C 5.523141 -2.876014 1.323216
C 6.758353 -3.693929 0.928207
C 7.870693 -3.552083 1.973833

C	9.104874	-4.371696	1.578985
C	10.217250	-4.231200	2.624783
C	11.450579	-5.052254	2.230236
C	12.562800	-4.913013	3.276390
C	13.795540	-5.735116	2.882145
C	14.907687	-5.596706	3.928536
C	16.139995	-6.419470	3.534497
C	17.251566	-6.280808	4.580952
C	18.479646	-7.098902	4.192443
C	0.283100	-0.421042	-2.249912
C	1.225539	0.735759	-1.843206
C	1.488331	1.673990	-3.028282
C	2.455277	2.798522	-2.638403
C	2.714796	3.736649	-3.822882
C	3.682272	4.860515	-3.433430
C	3.942129	5.798431	-4.618174
C	4.909981	6.922152	-4.229108
C	5.169951	7.859795	-5.414077
C	6.138038	8.983418	-5.025264
C	6.397995	9.920992	-6.210308
C	7.366398	11.044445	-5.821672
C	7.626124	11.982193	-7.006563
C	8.594706	13.104834	-6.617410
C	8.855574	14.040556	-7.794167
H	0.331999	-3.146412	-2.238035
H	-1.305784	-2.664001	-2.577312
H	-0.342141	-4.065607	0.005269
H	-2.028392	-3.546160	-0.284427
H	-2.128874	-4.972117	-2.333947
H	-0.430638	-5.508523	-2.031481
H	-1.123047	-6.446778	0.189208
H	-2.819926	-5.905215	-0.110129
H	-2.923355	-7.359833	-2.152163
H	-1.228005	-7.904134	-1.849359
H	-1.924822	-8.838910	0.372063
H	-3.619777	-8.293023	0.070453
H	-3.725264	-9.749190	-1.970381
H	-2.030837	-10.296039	-1.667307
H	-2.729569	-11.229355	0.554501
H	-4.423979	-10.682259	0.251401
H	-4.530735	-12.146430	-1.788588
H	-2.838145	-12.693304	-1.485225
H	-3.537389	-13.644640	0.723564
H	-5.237212	-13.095494	0.418733
H	-4.485643	-14.347441	-0.641223
H	-1.929655	-1.446372	0.321531
H	-0.547661	0.607395	0.911371
H	-1.331931	1.442937	-0.470917
H	-3.557520	1.082257	0.581570
H	-2.793247	0.122739	1.905096
H	-1.467765	2.134464	2.590220
H	-2.215414	3.086268	1.249755
H	-4.485661	2.709453	2.249221
H	-3.738265	1.754145	3.587265
H	-2.403816	3.765185	4.275929
H	-3.154549	4.719972	2.939220
H	-5.423122	4.338147	3.940776
H	-4.672409	3.382685	5.276911
H	-3.339733	5.394686	5.965652
H	-4.093517	6.349898	4.630991
H	-6.360233	5.964168	5.635372
H	-5.606346	5.008646	6.969676
H	-4.275582	7.021596	7.658476
H	-5.031557	7.976939	6.325172
H	-7.296943	7.588516	7.331466
H	-6.540912	6.632997	8.664571
H	-5.211631	8.646569	9.353612
H	-5.968602	9.602019	8.020945
H	-8.233424	9.212569	9.028228
H	-7.476334	8.257093	10.360925
H	-6.150433	10.276380	11.054241
H	-6.906935	11.230950	9.722898
H	-8.033487	11.659600	11.893116
H	-9.166852	10.862230	10.737505
H	-8.407029	9.903667	12.074566
H	-3.087444	0.455892	-1.646383
H	-2.535123	-0.943645	-2.521704
H	-3.843276	-2.479777	-1.112055
H	-4.332686	-1.125641	-0.029491

H	-5.484413	-0.007565	-1.949218
H	-4.971824	-1.350620	-3.043262
H	-6.243307	-2.986776	-1.627578
H	-6.757313	-1.642331	-0.536556
H	-7.908807	-0.519510	-2.461056
H	-7.391317	-1.860705	-3.554395
H	-8.661085	-3.501059	-2.142304
H	-9.180504	-2.158887	-1.051122
H	-10.330766	-1.037054	-2.977413
H	-9.809376	-2.377254	-4.070042
H	-11.078731	-4.019885	-2.660707
H	-11.601131	-2.679282	-1.569043
H	-12.750683	-1.557465	-3.496025
H	-12.227081	-2.896816	-4.588630
H	-13.496281	-4.541131	-3.181342
H	-14.020227	-3.201731	-2.088922
H	-15.169723	-2.079360	-4.015979
H	-14.645243	-3.418309	-5.108867
H	-15.921572	-5.064670	-3.702984
H	-16.445632	-3.727039	-2.611177
H	-17.085927	-3.957880	-5.624320
H	-18.215156	-4.293537	-4.257595
H	-17.612244	-2.614485	-4.527888
H	1.146057	-0.570354	0.422967
H	0.340393	-2.057898	0.909463
H	1.820175	-3.416908	-0.483278
H	2.446731	-1.979619	-1.355188
H	3.460200	-1.131188	0.768178
H	2.796451	-2.557964	1.655695
H	4.129815	-4.090996	0.183989
H	4.786304	-2.664691	-0.708557
H	5.805872	-1.804037	1.415574
H	5.147956	-3.229063	2.309206
H	6.475314	-4.765802	0.835519
H	7.133357	-3.340798	-0.057835
H	8.154720	-2.480412	2.065698
H	7.495433	-3.904261	2.960109
H	8.820448	-5.443210	1.486400
H	9.480453	-4.019167	0.592949
H	10.502533	-3.159867	2.716700
H	9.841389	-4.582903	3.611003
H	11.164950	-6.123437	2.137545
H	11.826824	-4.700124	1.244311
H	12.849028	-3.841954	3.368584
H	12.186305	-5.264535	4.262431
H	13.509066	-6.806069	2.789455
H	14.172271	-5.383341	1.896281
H	15.194589	-4.525835	4.020906
H	14.530836	-5.948088	4.914503
H	15.853310	-7.490438	3.442267
H	16.517192	-6.068150	2.548618
H	17.546393	-5.212888	4.675699
H	16.882756	-6.633343	5.568907
H	19.266263	-6.979216	4.966961
H	18.217588	-8.175727	4.115847
H	18.883986	-6.749290	3.218602
H	0.853138	-1.036845	-2.980022
H	-0.557547	0.007819	-2.830718
H	0.804664	1.328893	-1.012095
H	2.199660	0.324757	-1.505161
H	1.922466	1.090760	-3.870276
H	0.526009	2.119096	-3.364989
H	2.021223	3.381181	-1.795932
H	3.418745	2.355340	-2.302361
H	3.148164	3.153772	-4.665523
H	1.751450	4.180432	-4.158464
H	3.248763	5.443509	-2.590938
H	4.645485	4.416663	-3.097539
H	4.375314	5.215290	-5.460729
H	2.978923	6.242435	-4.953877
H	4.476759	7.505437	-3.386678
H	5.873130	6.478137	-3.893265
H	5.602975	7.276413	-6.256548
H	4.206819	8.303920	-5.749823
H	5.705026	9.566833	-4.182814
H	7.101162	8.539274	-4.689522
H	6.830751	9.337515	-7.052849
H	5.434888	10.365299	-6.545883
H	6.933784	11.627825	-4.978983

H	8.329589	10.600115	-5.486336
H	8.058687	11.399059	-7.849477
H	6.663114	12.427083	-7.341747
H	8.167253	13.694553	-5.777244
H	9.561431	12.667607	-6.284790
H	9.307278	13.479989	-8.640227
H	7.907251	14.511439	-8.130377
H	9.558931	14.841561	-7.483277

17,17,18-Tridodecyltetratriacontane

0 1

C	0.966696	0.452873	2.875962
C	1.375700	-1.038609	2.867114
C	1.399696	-1.611364	4.290142
C	1.845079	-3.078726	4.286830
C	1.866579	-3.650990	5.708931
C	2.313189	-5.117704	5.705086
C	2.335758	-5.690314	7.127180
C	2.783168	-7.156834	7.123121
C	2.806610	-7.729489	8.545244
C	3.254104	-9.195940	8.541090
C	3.277999	-9.767696	9.963201
C	3.722388	-11.227453	9.963110
C	0.668570	1.137260	1.481362
C	1.622860	0.568596	0.360680
C	3.123289	0.904757	0.530122
C	3.991455	0.046938	-0.399248
C	5.475424	0.404456	-0.254438
C	6.342269	-0.461909	-1.175529
C	7.826723	-0.107644	-1.028619
C	8.693457	-0.977295	-1.946913
C	10.178285	-0.624722	-1.799312
C	11.044709	-1.496786	-2.715646
C	12.529777	-1.145210	-2.568015
C	13.395835	-2.018929	-3.483139
C	14.881060	-1.667860	-3.335797
C	15.746953	-2.542911	-4.249856
C	17.232183	-2.191822	-4.103052
C	18.097173	-3.067743	-5.016541
C	19.576290	-2.721150	-4.873551
C	-0.863457	0.929873	1.059367
C	-1.272385	-0.549953	0.784921
C	-2.590527	-0.666492	-0.002795
C	-2.911999	-2.130872	-0.326859
C	-4.230977	-2.246907	-1.099973
C	-4.551929	-3.710471	-1.424912
C	-5.869801	-3.825960	-2.199943
C	-6.190891	-5.289415	-2.525523
C	-7.507296	-5.404431	-3.303167
C	-7.828117	-6.867805	-3.629461
C	-9.143118	-6.982587	-4.409505
C	-9.463904	-8.445964	-4.735885
C	-10.778200	-8.560574	-5.517157
C	-11.099166	-10.023942	-5.843491
C	-12.412832	-10.138386	-6.625758
C	-12.733380	-11.601648	-6.951344
C	-14.039996	-11.719598	-7.730591
C	-1.899346	1.573271	2.053431
C	-2.814266	2.606944	1.375434
C	-3.928121	3.068928	2.322666
C	-4.816819	4.123662	1.653055
C	-5.930129	4.586973	2.599769
C	-6.816782	5.644077	1.931095
C	-7.929924	6.108030	2.877915
C	-8.815726	7.166209	2.209709
C	-9.928692	7.630492	3.156654
C	-10.813856	8.689437	2.488918
C	-11.926357	9.153047	3.436179
C	-12.809122	10.207798	2.775428
C	0.966575	2.681571	1.649578
C	0.798943	3.555254	0.384833
C	1.090438	5.028442	0.697791
C	0.915946	5.903069	-0.549576
C	1.200367	7.375969	-0.233341
C	1.021210	8.250933	-1.479550
C	1.303583	9.724351	-1.163198
C	1.122751	10.599489	-2.409122

C	1.404648	12.073054	-2.092744
C	1.223071	12.948342	-3.338397
C	1.504706	14.421440	-3.021270
C	1.324832	15.295930	-4.258721
H	0.091405	0.565803	3.546149
H	1.793248	0.993167	3.387921
H	0.684307	-1.645322	2.255908
H	2.392032	-1.146419	2.434131
H	2.100907	-1.015291	4.915102
H	0.381676	-1.538756	4.732754
H	1.144043	-3.674408	3.661232
H	2.863422	-3.153108	3.845170
H	2.566983	-3.054802	6.334693
H	0.848069	-3.577694	6.150352
H	1.612503	-5.713872	5.079599
H	3.331422	-5.190891	5.262979
H	3.036112	-5.093851	7.752747
H	1.317434	-5.617629	7.569138
H	2.082528	-7.753335	6.497911
H	3.801256	-7.229505	6.680602
H	3.507251	-7.132959	9.170439
H	1.788533	-7.656920	8.987809
H	2.553199	-9.792796	7.916467
H	4.272096	-9.268822	8.098316
H	3.980043	-9.178352	10.592619
H	2.262562	-9.701185	10.411340
H	3.730450	-11.613605	11.004160
H	3.021544	-11.844162	9.360970
H	4.746441	-11.319100	9.542396
H	1.542086	-0.532632	0.304413
H	1.292676	0.934855	-0.635101
H	3.303747	1.971951	0.285554
H	3.444015	0.736954	1.579035
H	3.848659	-1.027396	-0.148102
H	3.673422	0.209962	-1.452716
H	5.622702	1.476656	-0.511831
H	5.789934	0.247639	0.801091
H	6.193156	-1.534149	-0.919218
H	6.028950	-0.303846	-2.231195
H	7.976582	0.963868	-1.287443
H	8.139205	-0.263361	0.027657
H	8.542542	-2.048770	-1.688480
H	8.381584	-0.821127	-3.003295
H	10.329692	0.446201	-2.059662
H	10.489727	-0.779168	-0.742547
H	10.892731	-2.567672	-2.455406
H	10.733483	-1.342188	-3.772450
H	12.682081	-0.074697	-2.829537
H	12.840788	-1.298683	-1.510982
H	13.243274	-3.089393	-3.221538
H	13.084799	-1.865532	-4.540174
H	15.033809	-0.597697	-3.598496
H	15.191892	-1.820263	-2.278561
H	15.594325	-3.613035	-3.986875
H	15.435905	-2.390846	-5.307089
H	17.385219	-1.121934	-4.366825
H	17.543459	-2.343471	-3.045806
H	17.952265	-4.138948	-4.755989
H	17.793942	-2.918829	-6.075898
H	19.752926	-1.660616	-5.153099
H	19.911902	-2.885895	-3.827459
H	20.176732	-3.370084	-5.545356
H	-0.970935	1.428520	0.073593
H	-0.504378	-1.070528	0.185785
H	-1.391757	-1.092794	1.746540
H	-3.427775	-0.244237	0.591993
H	-2.506414	-0.093292	-0.952206
H	-2.089131	-2.562633	-0.938397
H	-2.988720	-2.708504	0.620798
H	-5.054949	-1.816834	-0.488635
H	-4.154156	-1.668052	-2.046870
H	-3.727451	-4.141092	-2.035159
H	-4.630130	-4.288872	-0.477846
H	-6.694377	-3.395498	-1.589715
H	-5.791398	-3.247216	-3.146786
H	-5.365425	-5.720441	-3.134146
H	-6.271235	-5.867787	-1.578621
H	-8.332932	-4.973781	-2.694507
H	-7.426915	-4.825648	-4.249822

H	-7.001634	-7.298978	-4.236594
H	-7.910302	-7.446192	-2.682729
H	-9.969703	-6.551406	-3.802516
H	-9.060780	-6.404175	-5.356217
H	-8.636908	-8.877391	-5.342144
H	-9.547132	-9.024212	-3.789154
H	-11.605212	-8.129013	-4.911021
H	-10.694816	-7.982389	-6.463912
H	-10.271824	-10.455784	-6.448995
H	-11.183355	-10.601962	-4.896696
H	-13.240392	-9.706519	-6.020535
H	-12.328639	-9.560835	-7.572865
H	-11.911658	-12.039351	-7.559249
H	-12.823178	-12.184621	-6.008772
H	-14.245840	-12.788138	-7.951543
H	-14.883434	-11.311449	-7.133839
H	-13.967929	-11.165850	-8.690935
H	-2.535746	0.787861	2.516754
H	-1.421346	2.062201	2.920707
H	-2.211746	3.489356	1.077675
H	-3.270990	2.168425	0.462285
H	-4.551051	2.193993	2.612400
H	-3.473869	3.500495	3.241915
H	-4.193947	4.998360	1.362092
H	-5.271216	3.691838	0.733993
H	-6.554300	3.712755	2.889152
H	-5.475445	5.016846	3.519593
H	-6.192302	6.518026	1.641506
H	-7.271616	5.214254	1.011328
H	-8.554952	5.234257	3.166805
H	-7.475044	6.537015	3.798057
H	-8.190571	8.039838	1.920640
H	-9.270783	6.737207	1.289667
H	-10.554292	6.756992	3.445162
H	-9.473642	8.058851	4.077013
H	-10.188480	9.563196	2.200624
H	-11.269196	8.261353	1.568553
H	-12.558510	8.285243	3.725420
H	-11.478535	9.585212	4.357620
H	-13.604056	10.523723	3.483413
H	-12.205264	11.098337	2.499045
H	-13.289767	9.792821	1.863915
H	0.351232	3.118959	2.458493
H	2.007360	2.820365	2.013115
H	1.492691	3.223195	-0.413369
H	-0.229972	3.484397	-0.012052
H	0.394598	5.380177	1.491428
H	2.133303	5.125905	1.072555
H	1.613732	5.555508	-1.343204
H	-0.126141	5.802838	-0.926062
H	0.504136	7.722342	0.562221
H	2.243181	7.477140	0.140658
H	1.718050	7.905314	-2.274882
H	-0.021361	8.148524	-1.853958
H	0.607347	10.069566	-0.367149
H	2.346451	9.827007	-0.789730
H	1.819094	10.254459	-3.205150
H	0.079921	10.496516	-2.782641
H	0.708617	12.417917	-1.296363
H	2.447635	12.176128	-1.719695
H	1.919327	12.603985	-4.134831
H	0.180113	12.845439	-3.711643
H	0.809737	14.773871	-2.228061
H	2.547244	14.532727	-2.651007
H	1.534889	16.354939	-3.999215
H	2.027117	14.980502	-5.059586
H	0.282143	15.222549	-4.634892

15-(2,4,4,6,6-pentamethylheptan-2-yl)-16-tetradecyl-16-tridecylhentriacontane

0 1

C	-2.538142	-1.969358	-3.922999
C	-2.984545	-2.209968	-2.469157
C	-4.434819	-1.672916	-2.306377
C	-3.082582	-3.777456	-2.121467
C	-3.619122	-4.951814	-3.057480
C	-3.810597	-6.199820	-2.091946
C	-3.898389	-7.701953	-2.566528

C	-2.522046	-8.257537	-3.009323
C	-4.343233	-8.534774	-1.337232
C	-4.939809	-7.940755	-3.676754
C	-4.975566	-4.616937	-3.717036
C	-2.580587	-5.266702	-4.157351
C	-1.968698	-1.453159	-1.428696
C	-0.644023	-2.288946	-1.205908
C	-0.631848	-3.056650	0.131428
C	0.529838	-4.054981	0.185273
C	0.544195	-4.810970	1.518624
C	1.700379	-5.816381	1.568641
C	1.713016	-6.574177	2.901357
C	2.869493	-7.579475	2.952421
C	2.882088	-8.336583	4.285623
C	4.039738	-9.340519	4.338066
C	4.052567	-10.096779	5.671782
C	5.210804	-11.100053	5.725000
C	5.224152	-11.855454	7.059148
C	6.382753	-12.857808	7.112044
C	6.399846	-13.611204	8.438874
C	-1.557370	0.124442	-1.562552
C	-0.943903	0.569748	-0.160653
C	-0.665132	2.083131	0.068481
C	0.630528	2.311467	0.861345
C	0.887792	3.806082	1.086476
C	2.190370	4.027856	1.863946
C	2.452867	5.522095	2.084807
C	3.758262	5.742693	2.858170
C	4.022838	7.236819	3.077979
C	5.329336	7.456662	3.849770
C	5.594839	8.950707	4.069267
C	6.901868	9.170197	4.840278
C	7.167455	10.664204	5.060204
C	8.474682	10.883590	5.830800
C	8.739364	12.377393	6.050782
C	10.039715	12.599850	6.817588
C	-0.518553	0.305401	-2.764539
C	0.904825	0.836036	-2.471217
C	1.814916	0.684173	-3.698858
C	3.212417	1.252421	-3.421875
C	4.121945	1.100617	-4.646540
C	5.517525	1.671755	-4.369173
C	6.427164	1.522890	-5.594368
C	7.821942	2.096252	-5.317344
C	8.731452	1.948891	-6.542888
C	10.125735	2.523708	-6.266193
C	11.035068	2.377413	-7.491952
C	12.428652	2.952702	-7.214654
C	13.336668	2.809386	-8.432579
C	-2.771640	1.078461	-1.861590
C	-3.859745	1.177865	-0.765739
C	-5.098449	1.926183	-1.277081
C	-6.184218	1.996969	-0.196784
C	-7.420877	2.745842	-0.706915
C	-8.506979	2.814650	0.373056
C	-9.744717	3.561705	-0.137544
C	-10.830828	3.630557	0.942505
C	-12.068897	4.377044	0.431770
C	-13.155090	4.445690	1.511787
C	-14.393376	5.191792	1.000924
C	-15.479625	5.260310	2.080803
C	-16.717364	6.006134	1.569284
C	-17.800912	6.076398	2.641366
H	-2.494813	-0.889215	-4.153805
H	-1.541540	-2.417025	-4.111644
H	-3.260885	-2.374402	-4.651705
H	-5.167957	-2.235313	-2.894525
H	-4.771966	-1.744467	-1.252076
H	-4.582836	-0.660211	-2.687044
H	-2.097281	-4.161748	-1.839444
H	-3.649298	-3.831496	-1.163939
H	-2.986402	-6.184957	-1.342881
H	-4.721018	-5.987050	-1.486536
H	-1.725628	-7.963308	-2.292641
H	-2.543999	-9.368101	-3.055274
H	-2.231402	-7.924683	-4.021770
H	-5.345415	-8.211591	-0.980398
H	-4.407325	-9.617257	-1.583561
H	-3.621548	-8.417398	-0.499512

H	-4.610326	-7.526834	-4.648624
H	-5.090595	-9.030072	-3.839965
H	-5.920703	-7.502748	-3.393294
H	-4.897397	-3.771257	-4.422075
H	-5.350826	-5.446596	-4.337147
H	-5.747548	-4.403168	-2.947767
H	-1.619917	-5.600454	-3.710708
H	-2.957230	-6.036252	-4.854577
H	-2.370854	-4.402538	-4.804559
H	-2.525197	-1.508305	-0.468044
H	-0.431817	-2.953012	-2.068106
H	0.267892	-1.669625	-1.210236
H	-0.529602	-2.326190	0.963520
H	-1.587157	-3.600890	0.284608
H	0.424332	-4.784313	-0.648273
H	1.490723	-3.508768	0.059386
H	0.652449	-4.083042	2.352866
H	-0.419355	-5.352770	1.644414
H	1.592138	-6.543209	0.733361
H	2.664176	-5.274994	1.443819
H	1.820407	-5.847292	3.736650
H	0.749212	-7.115729	3.025626
H	2.761932	-8.306678	2.117432
H	3.833254	-7.037968	2.827803
H	2.988106	-7.609165	5.120627
H	1.918813	-8.879119	4.409559
H	3.933526	-10.068359	3.503466
H	5.002935	-8.797955	4.213644
H	4.158047	-9.368749	6.506319
H	3.089601	-10.639811	5.795935
H	5.105016	-11.828559	4.890913
H	6.173735	-10.557092	5.600206
H	5.329683	-11.127087	7.893418
H	4.261570	-12.399079	7.183917
H	6.281644	-13.592004	6.283135
H	7.348969	-12.320990	6.991163
H	6.525559	-12.901392	9.284128
H	5.453911	-14.178031	8.573033
H	7.247712	-14.328144	8.448314
H	-1.634111	0.252437	0.650957
H	-0.022697	0.004284	0.065864
H	-0.612453	2.650667	-0.877373
H	-1.496569	2.529631	0.651805
H	0.552269	1.797296	1.844721
H	1.488157	1.876142	0.303836
H	0.956136	4.319440	0.101850
H	0.038878	4.244368	1.656264
H	2.121996	3.516472	2.849532
H	3.037981	3.586212	1.294504
H	2.518520	6.033618	1.099085
H	1.606994	5.963598	2.656774
H	3.692359	5.231867	3.844226
H	4.603645	5.299866	2.286448
H	4.087731	7.747808	2.091941
H	3.178172	7.679555	3.650782
H	5.264319	6.945914	4.835931
H	6.173807	7.013378	3.277074
H	5.659348	9.461572	3.083141
H	4.750723	9.393903	4.642528
H	6.837511	8.659059	5.826281
H	7.745946	8.727138	4.266837
H	7.231586	11.175443	4.074228
H	6.323548	11.107207	5.633955
H	8.410902	10.372332	6.816808
H	9.318847	10.441150	5.256953
H	8.808129	12.894713	5.068940
H	7.901000	12.825939	6.627247
H	10.898689	12.182708	6.249921
H	10.204602	13.688418	6.961853
H	9.987699	12.113293	7.814908
H	-0.367564	-0.663032	-3.272539
H	-0.943311	0.961000	-3.556694
H	0.867292	1.913378	-2.226735
H	1.373985	0.283825	-1.633766
H	1.904530	-0.393208	-3.961477
H	1.363316	1.222137	-4.561593
H	3.126262	2.330105	-3.159484
H	3.663957	0.714208	-2.559265
H	4.210570	0.022875	-4.907667

H	3.669495	1.637601	-5.509392
H	5.428371	2.749021	-4.106133
H	5.970457	1.133736	-3.507199
H	6.517662	0.445560	-5.856580
H	5.973479	2.059779	-6.456642
H	7.731145	3.173347	-5.054220
H	8.276008	1.558875	-4.455573
H	8.823110	0.871745	-6.805477
H	8.276872	2.485515	-7.404859
H	10.033940	3.600693	-6.002933
H	10.580615	1.986689	-5.404618
H	11.127479	1.300464	-7.755199
H	10.580370	2.914388	-8.353681
H	12.344504	4.030361	-6.954000
H	12.891262	2.417882	-6.356563
H	14.336709	3.233647	-8.202600
H	13.458262	1.737883	-8.699364
H	12.909202	3.357320	-9.299255
H	-2.398224	2.110603	-2.022505
H	-3.202839	0.850623	-2.850446
H	-4.148599	0.181872	-0.391001
H	-3.472397	1.734169	0.107764
H	-4.804465	2.957533	-1.572633
H	-5.509277	1.412142	-2.172098
H	-6.475656	0.964718	0.098636
H	-5.780043	2.520249	0.697855
H	-7.129822	3.778081	-1.002433
H	-7.824652	2.222357	-1.601653
H	-8.796832	1.782293	0.669437
H	-8.103619	3.339147	1.267371
H	-9.454927	4.594016	-0.434097
H	-10.148041	3.037020	-1.031769
H	-11.120291	2.598227	1.239327
H	-10.427629	4.155566	1.836589
H	-11.779506	5.409426	0.135062
H	-12.472007	3.852078	-0.462388
H	-13.444259	3.413297	1.808692
H	-12.752074	4.970896	2.405845
H	-14.104281	6.224233	0.704102
H	-14.796355	4.666622	0.106821
H	-15.768991	4.227960	2.377758
H	-15.077044	5.785789	2.974925
H	-16.436466	7.040634	1.273885
H	-17.127708	5.484299	0.677281
H	-18.682950	6.620904	2.243251
H	-18.118487	5.053649	2.936695
H	-17.424191	6.616547	3.536023
